# Supplementary material for: PPTC7 antagonizes mitophagy by promoting BNIP3 and NIX degradation via SCFFBXL4
Source: EMBO Rep. 2024 Jul 11;25(8):3324–47. doi: 10.1038/s44319-024-00181-y (PMC11316107; doi:10.1038/s44319-024-00181-y)
Supplement: Supplementary file 1 — Appendix [file 44319_2024_181_MOESM1_ESM.pdf]

Appendix

Appendix Figure S1 Page 2
Appendix Table S1 Page 3

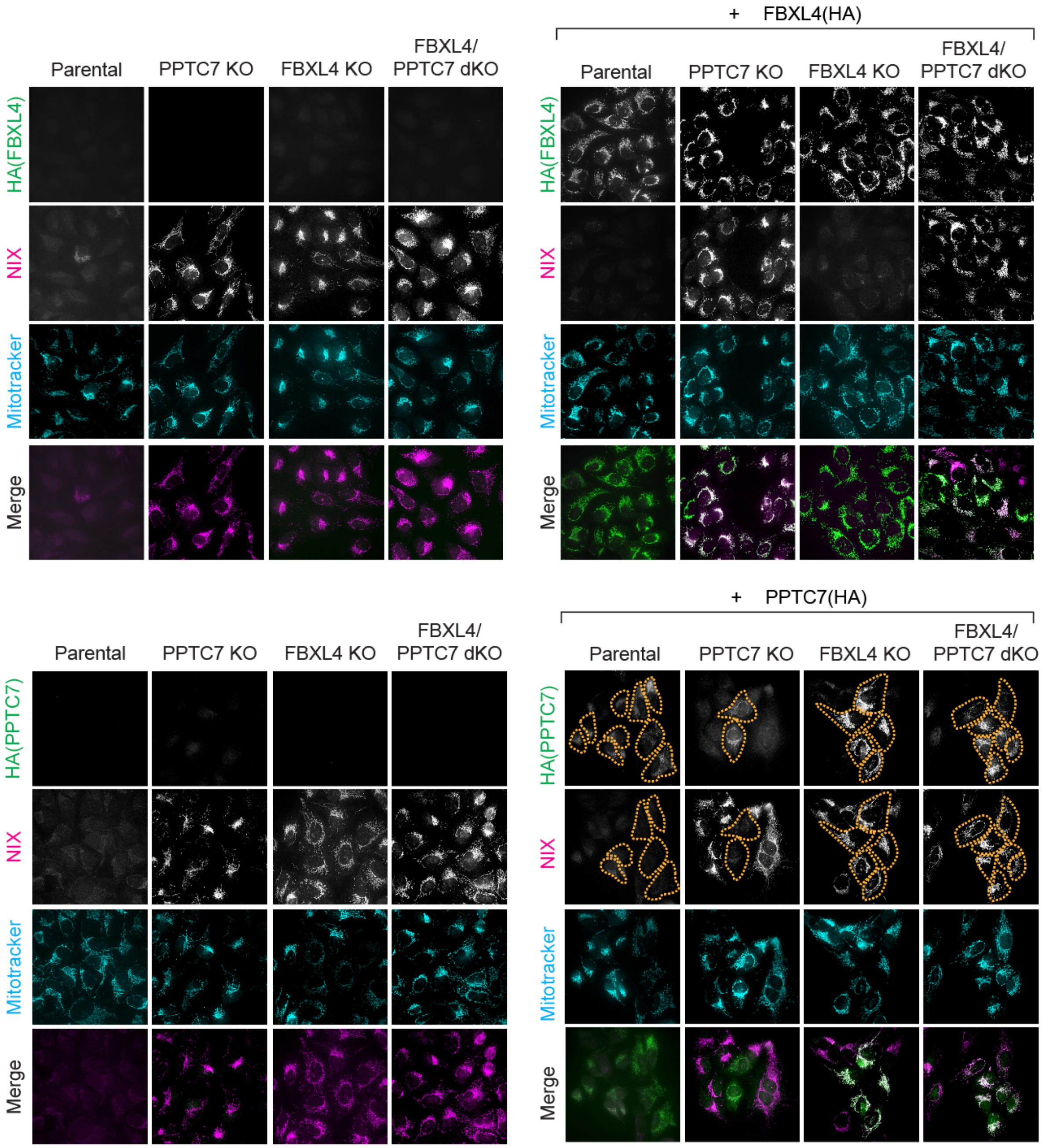

Appendix Figure S1. Full figures for 1E and 1F. FBXL4-HA or PPTC7-HA (green) were transduced in parental, FBXL4 KO, PPTC7 KO, and FBXL4/PPTC7 dKO cells. NIX protein levels (magenta) were monitored.

Syringe sample	Cell sample	Kd (nM)	N	ΔH (kcal/mol)	-TΔS (kcal/mol)	ΔG (kcal/mol)	Figure #
Mn^{2+}	PPTC7	6.21	1.14	-10.9	-0.46	-11.4	EV2D
Mg^{2+}	PPTC7	237	0.83	-2.03	-7.03	-9.06	EV2D
**Syringe sample**	**Cell sample**	**Kd (μM)**	**N**	**ΔH (kcal/mol)**	**-TΔS (kcal/mol)**	**ΔG (kcal/mol)**	**Figure #**
BNIP3	PPTC7 + Mn^{2+}	20.1	1	-3.22	-3.19	-6.41	EV2E
NIX	PPTC7 + Mn^{2+}	37.5	1	-5.22	-0.82	-6.04	EV2E
**Syringe sample**	**Cell sample**	**Kd (μM)**	**N**	**ΔH (kcal/mol)**	**-TΔS (kcal/mol)**	**ΔG (kcal/mol)**	**ΔG (kcal/mol)**
NIX	PPTC7 D290N	35.9±1.09	1	-4.12±0.55	-1.94±0.52	-6.07±0.02	EV4B
NIX	PPTC7 D290N Y179D	NB					EV4B

Appendix Table S1. Thermodynamic binding parameters from ITC measurements.
