# Supplementary material for: PPTC7 antagonizes mitophagy by promoting BNIP3 and NIX degradation via SCFFBXL4
Source: EMBO Rep. 2024 Jul 11;25(8):3324–47. doi: 10.1038/s44319-024-00181-y (PMC11316107; doi:10.1038/s44319-024-00181-y)
Supplement: Supplementary file 3 — Source data Fig. 1 [file 44319_2024_181_MOESM3_ESM.zip › Figure 1/Figure 1B/Annotations Figure 1B.pdf]

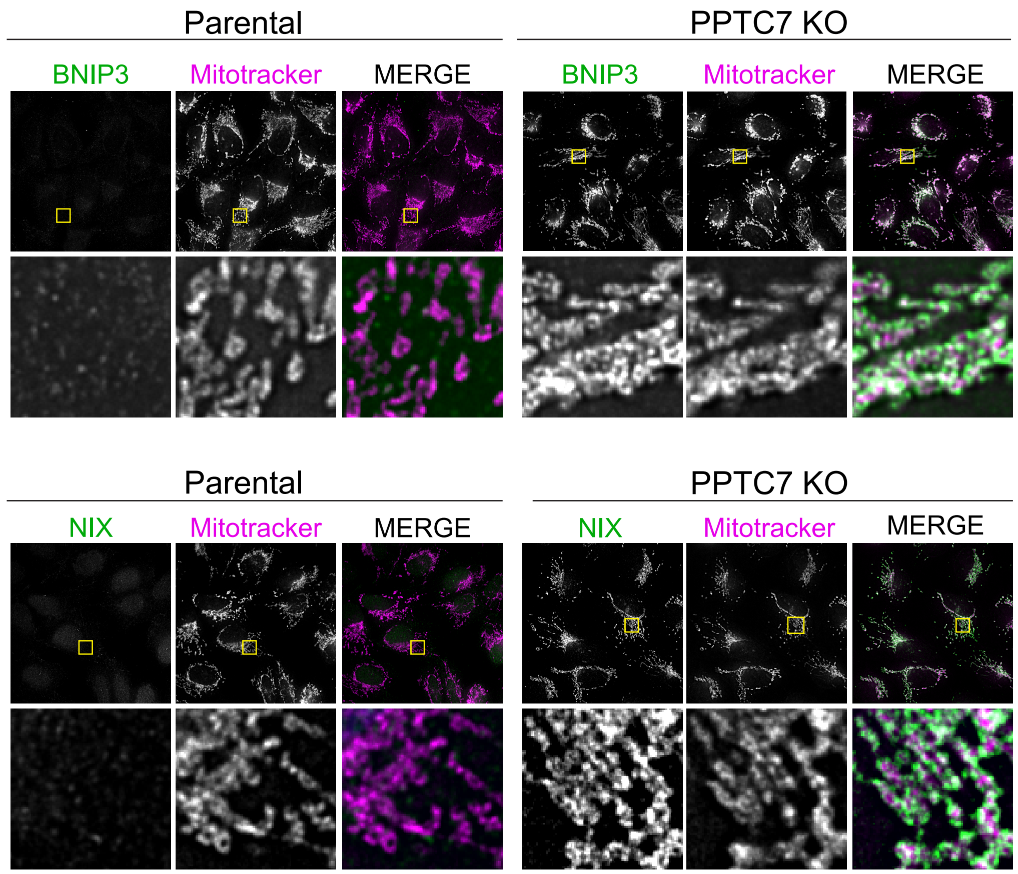

Parental
PPTC7 KO
BNIP3
Mitotracker
MERGE
BNIP3
Mitotracker
MERGE
Parental
PPTC7 KO
NIX
Mitotracker
MERGE
NIX
Mitotracker
MERGE

PPTC7KO_488flag_555nix_mitotracker_04_P2_R3D_D3D-1-1.tif
U2OS_488flag_555nix_mitotracker_07_P4_R3D_D3D-1-1.tif

pptc7KO_BNIP3488_FLAGm555_mrdr_11_P5_R3D_D3D-2.tif
U2OS_BNIP3488_FLAGm555_mrdr_12_P3_R3D_D3D-1.tif

Prepared with Brendan
Stained with Taylor

120 micro m
