# Supplementary figures and images for: PPTC7 antagonizes mitophagy by promoting BNIP3 and NIX degradation via SCFFBXL4 (part 1 of 2)
Source: EMBO Rep. 2024 Jul 11;25(8):3324–47. doi: 10.1038/s44319-024-00181-y (PMC11316107; doi:10.1038/s44319-024-00181-y)

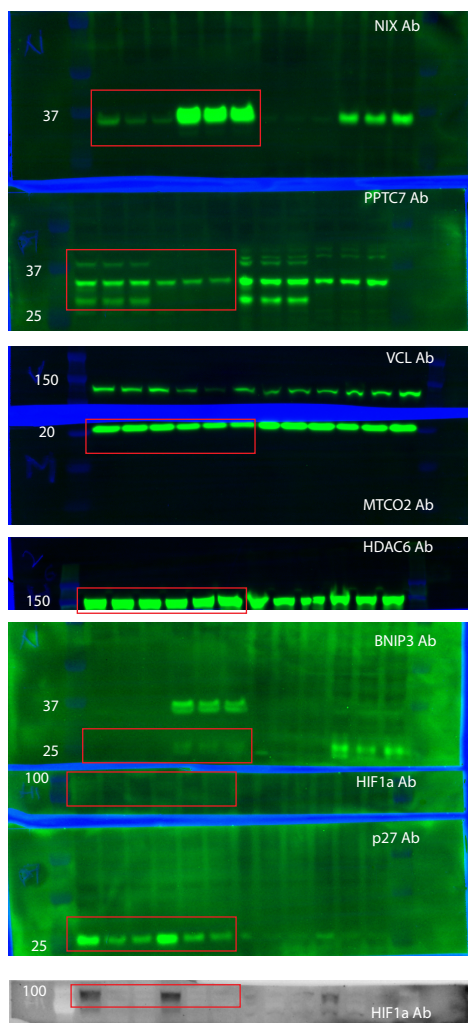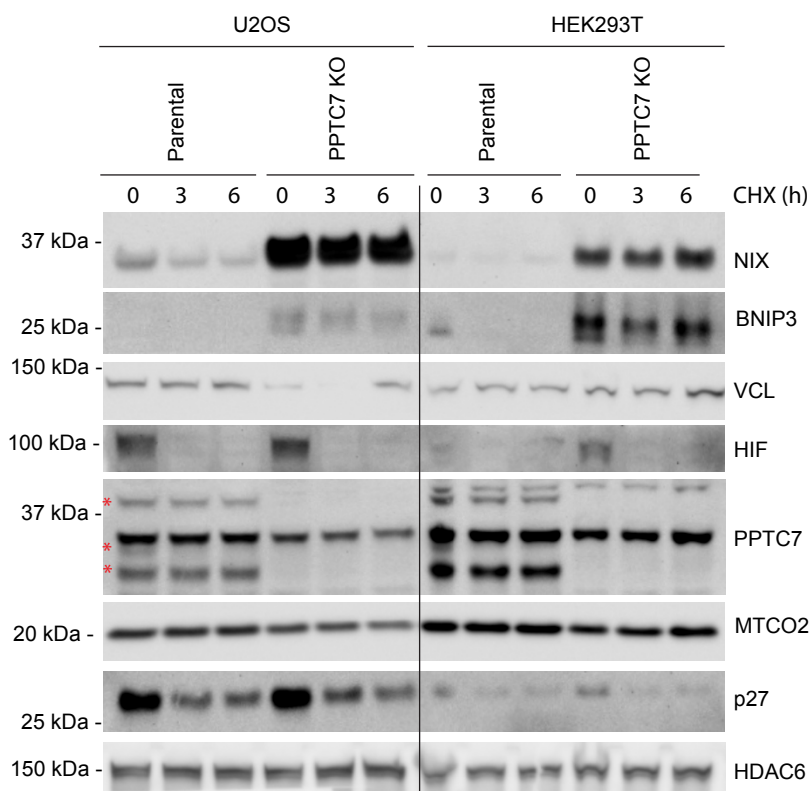

Supplement: Supplementary file 3 — Source data Fig. 1 [file 44319_2024_181_MOESM3_ESM.zip › Figure 1/Figure 1A/Annotation Figure 1A.pdf]

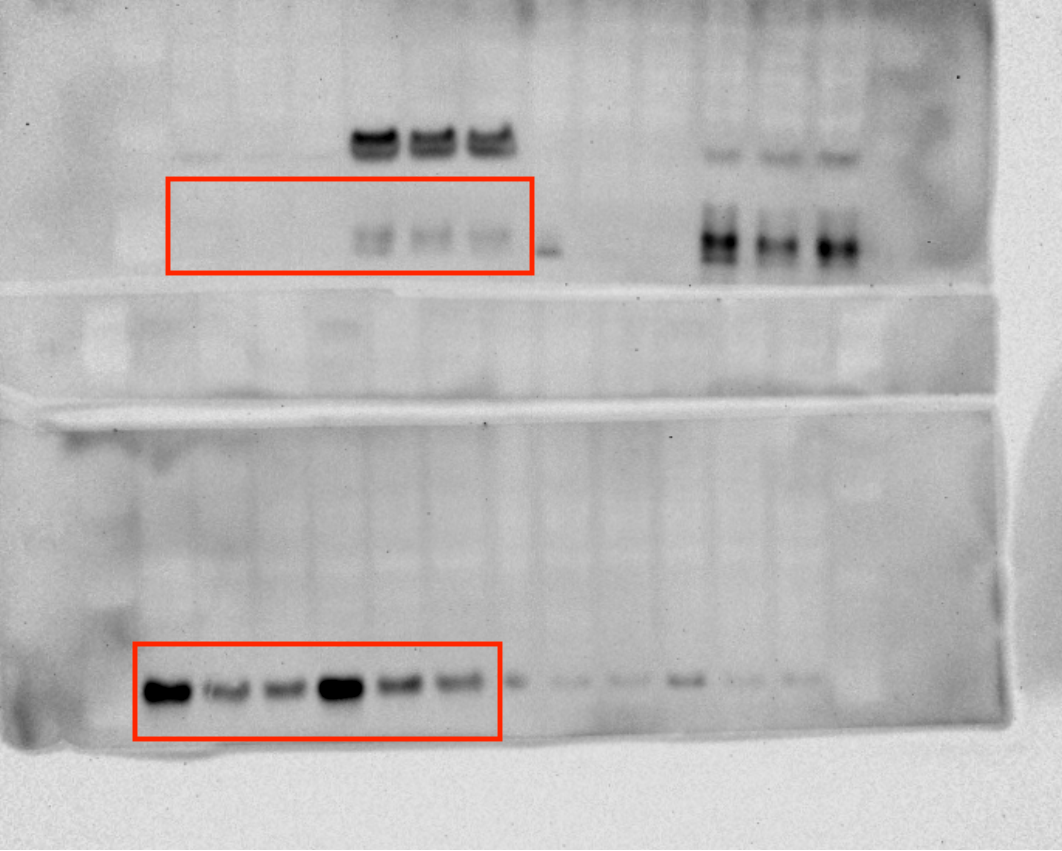

Supplement: Supplementary file 3 — Source data Fig. 1 [file 44319_2024_181_MOESM3_ESM.zip › Figure 1/Figure 1A/BNIP3-top_HIF-middle_p27-bottom.tif]

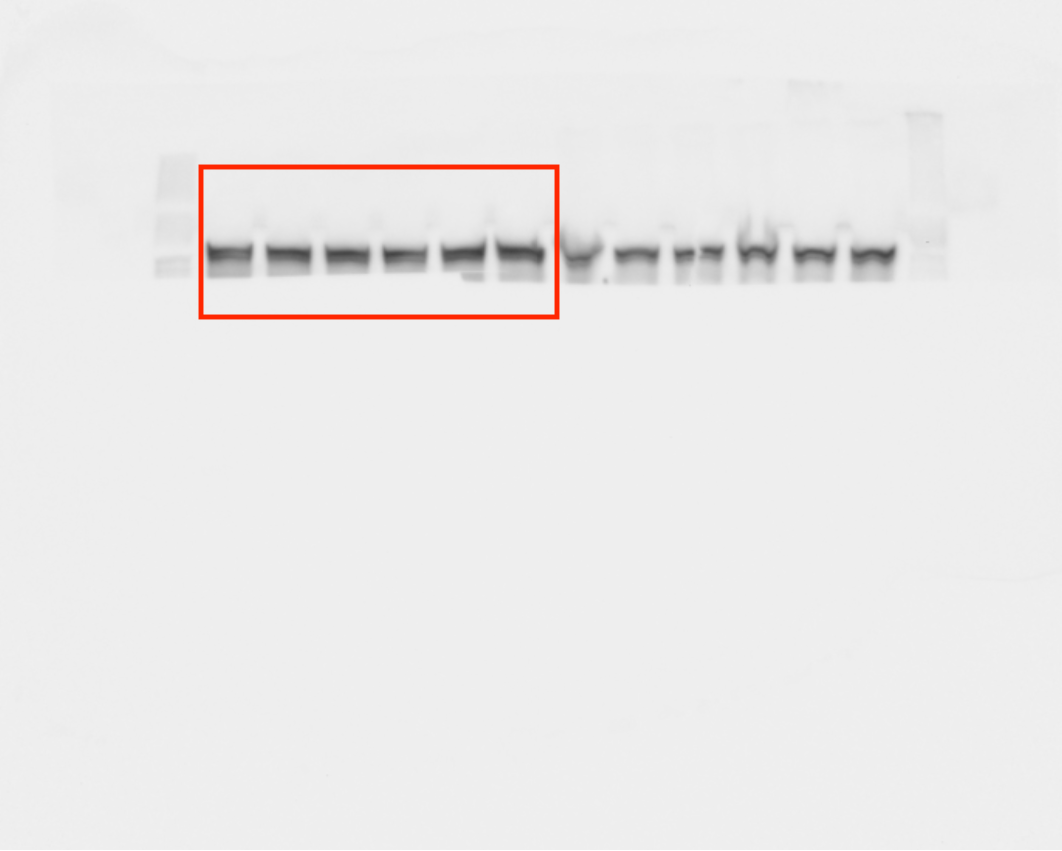

Supplement: Supplementary file 3 — Source data Fig. 1 [file 44319_2024_181_MOESM3_ESM.zip › Figure 1/Figure 1A/HDAC6.tif]

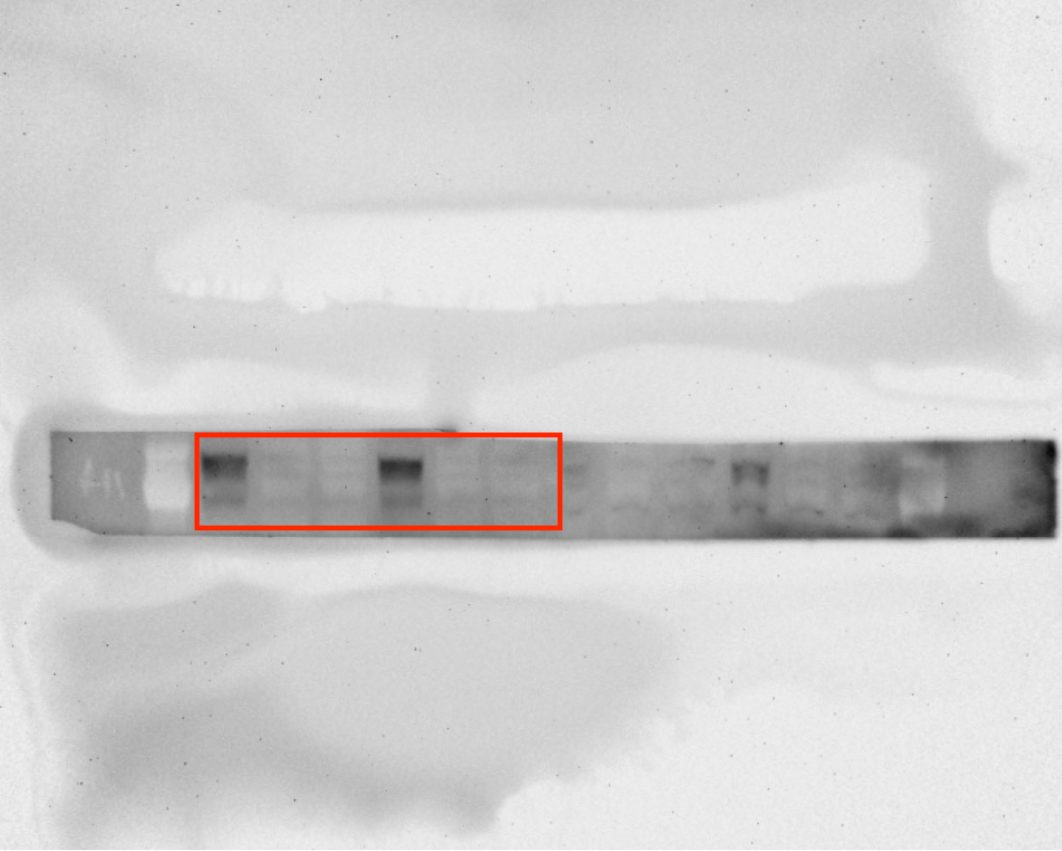

Supplement: Supplementary file 3 — Source data Fig. 1 [file 44319_2024_181_MOESM3_ESM.zip › Figure 1/Figure 1A/HIF.tif]

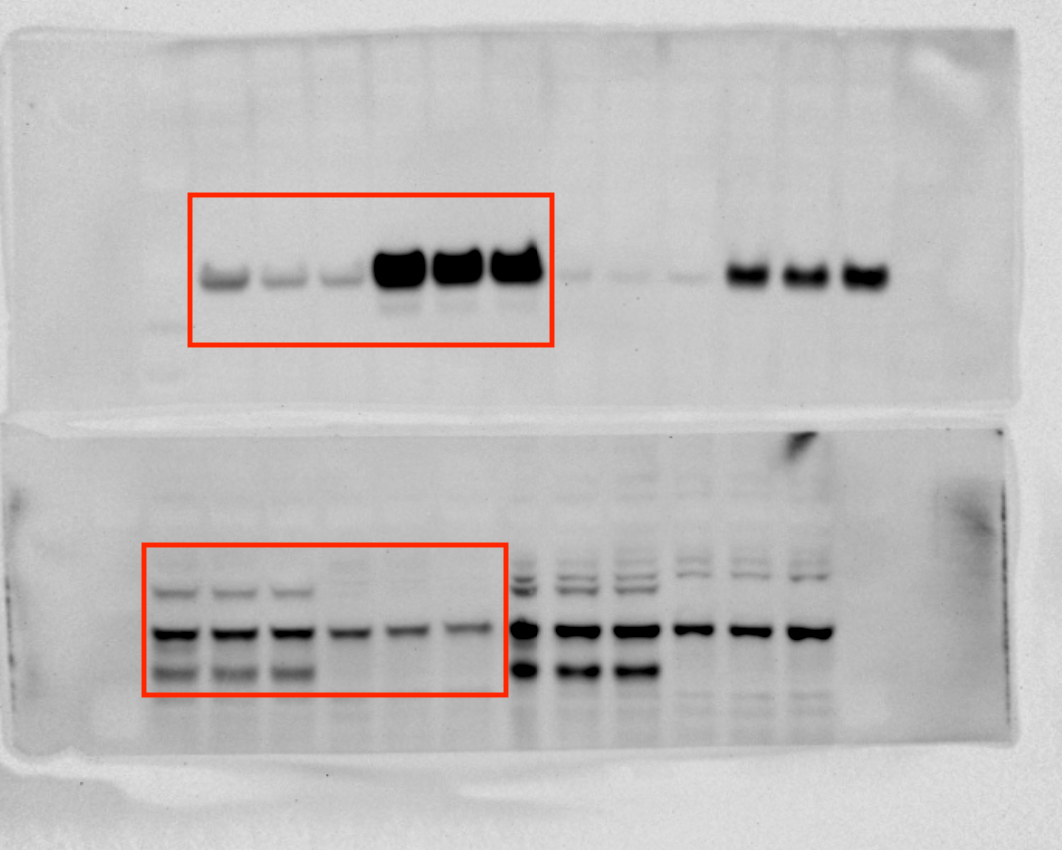

Supplement: Supplementary file 3 — Source data Fig. 1 [file 44319_2024_181_MOESM3_ESM.zip › Figure 1/Figure 1A/NIX-top_PPTC7-bottom.tif]

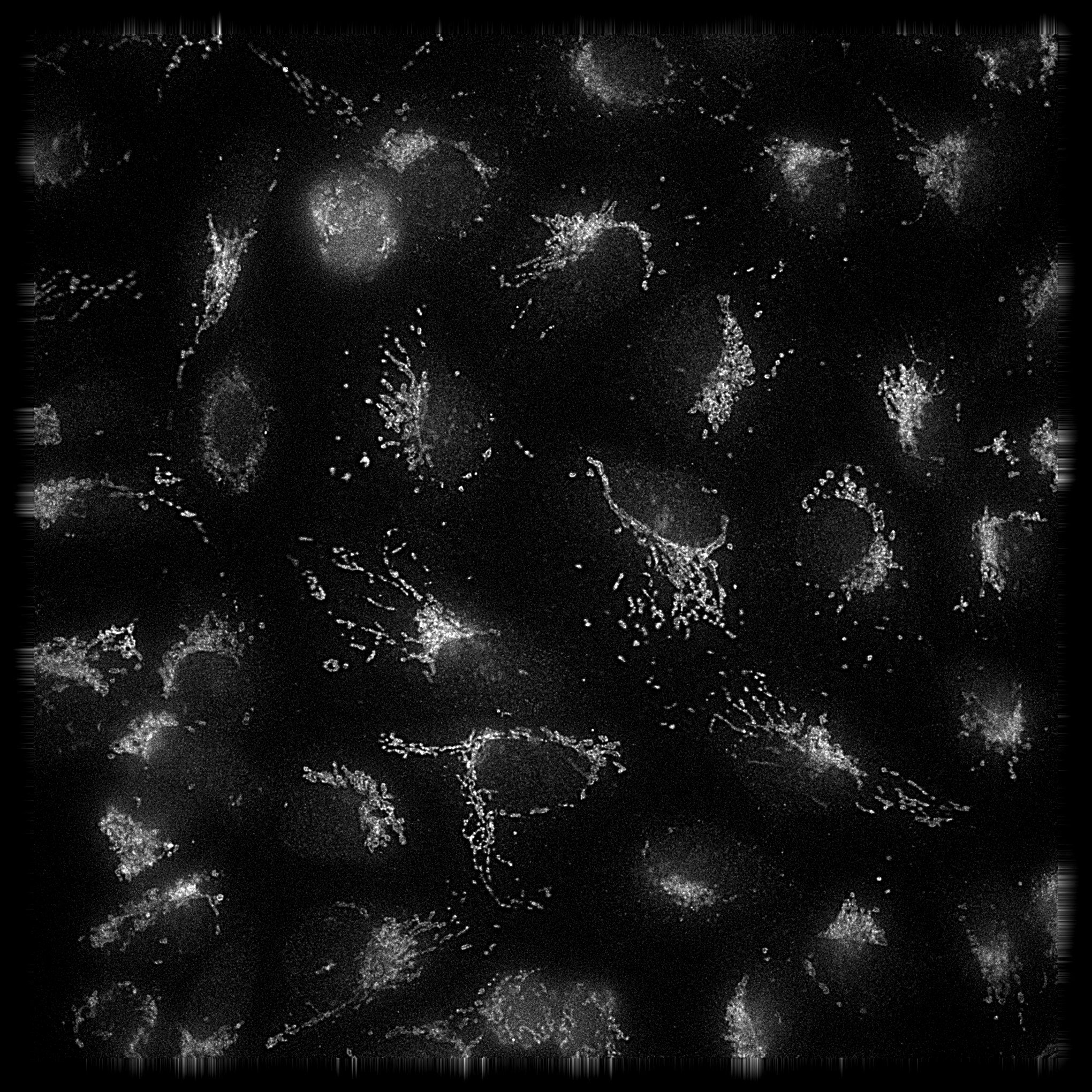

Supplement: Supplementary file 3 — Source data Fig. 1 [file 44319_2024_181_MOESM3_ESM.zip › Figure 1/Figure 1B/PPTC7 KO NIX.tif]

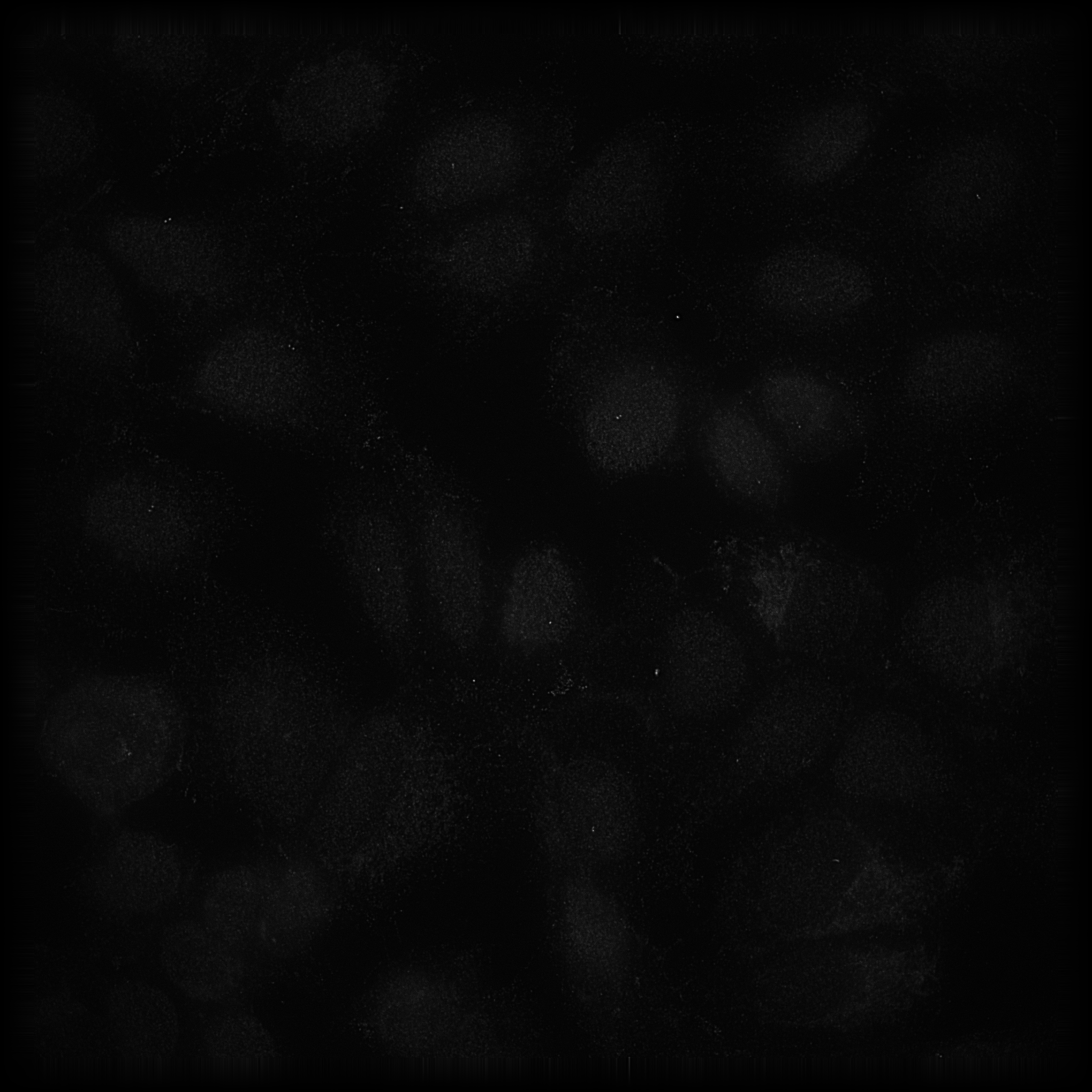

Supplement: Supplementary file 3 — Source data Fig. 1 [file 44319_2024_181_MOESM3_ESM.zip › Figure 1/Figure 1B/U2OS-NIX.tif]

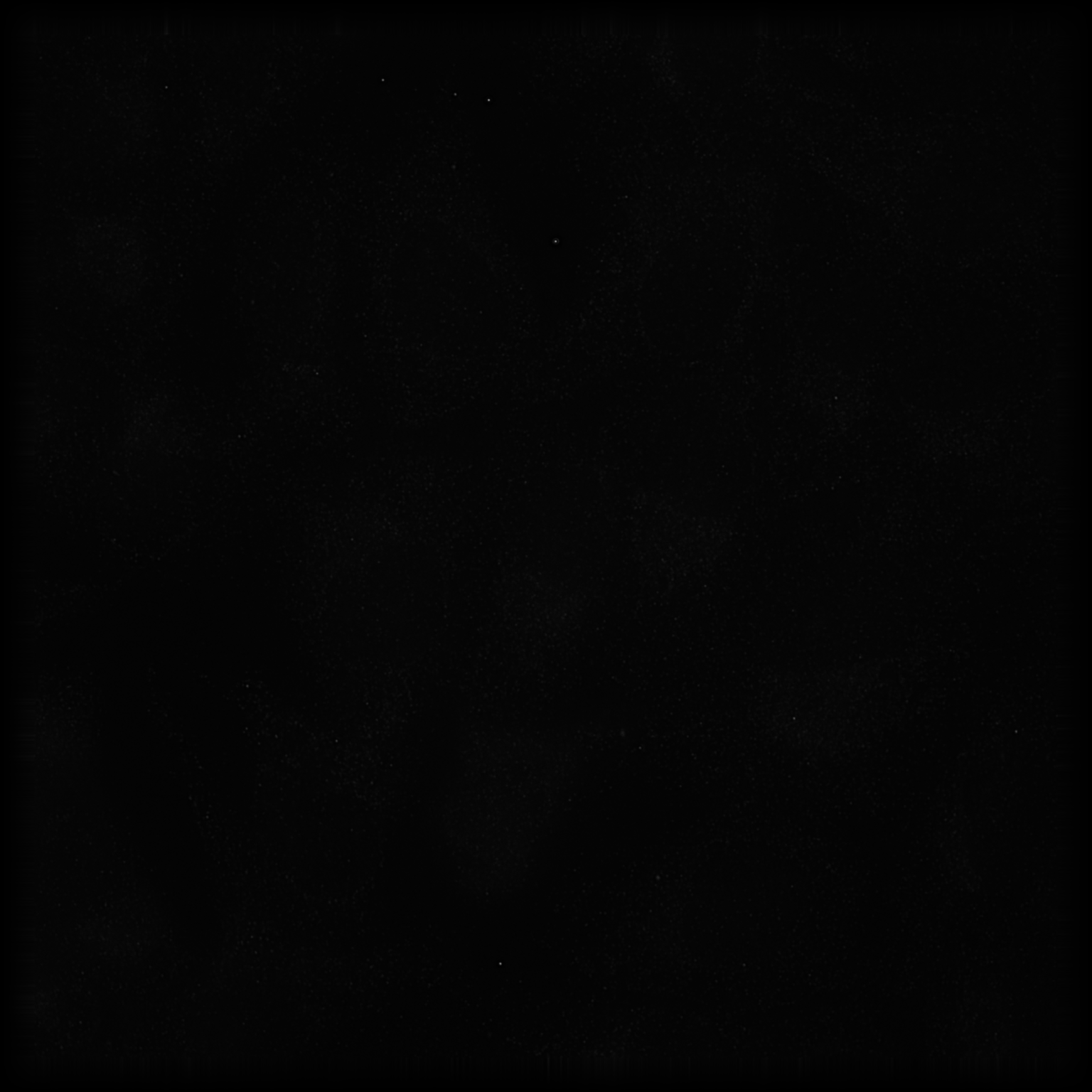

Supplement: Supplementary file 3 — Source data Fig. 1 [file 44319_2024_181_MOESM3_ESM.zip › Figure 1/Figure 1B/U2OS_BNIP3.tif]

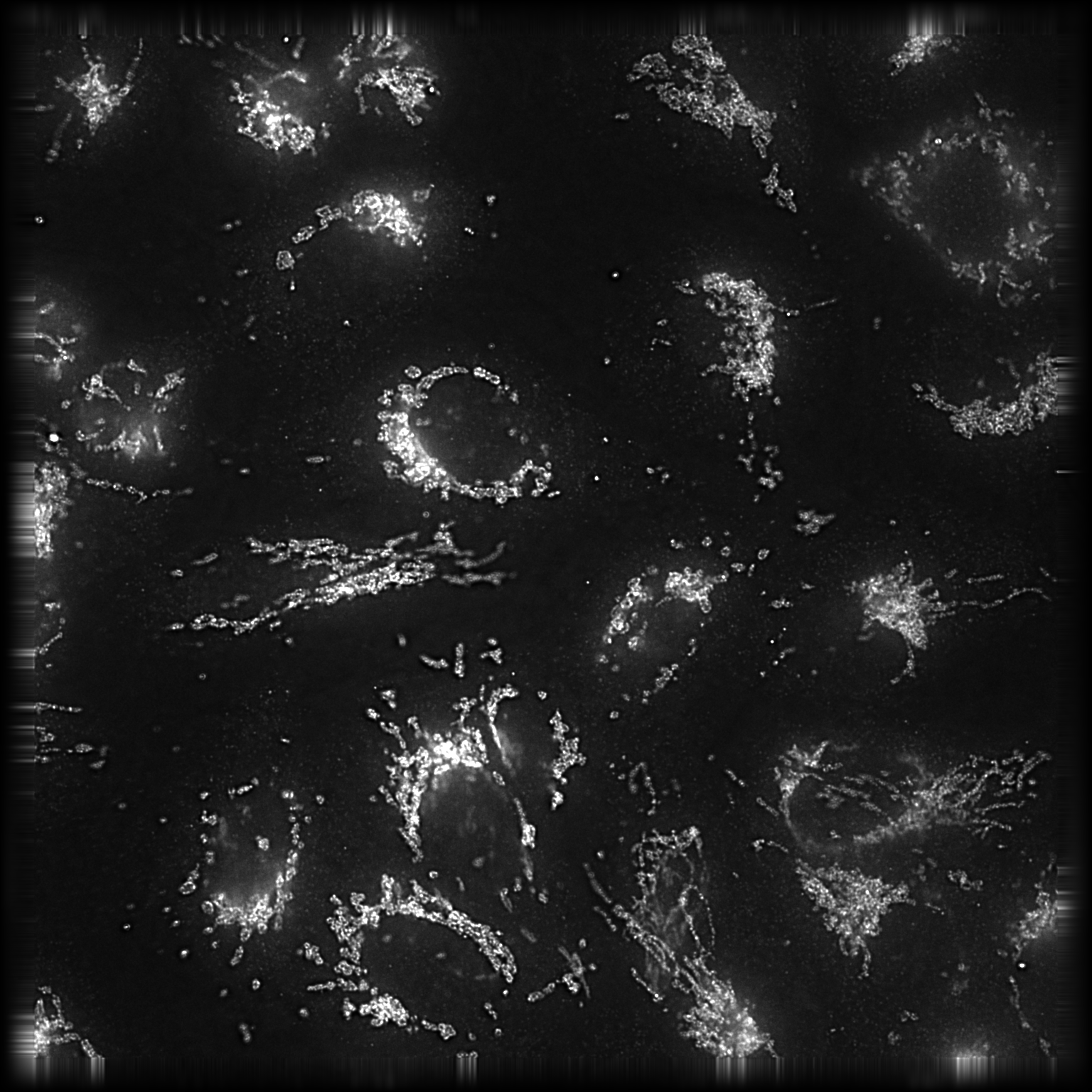

Supplement: Supplementary file 3 — Source data Fig. 1 [file 44319_2024_181_MOESM3_ESM.zip › Figure 1/Figure 1B/pptc7KO_BNIP3.tif]

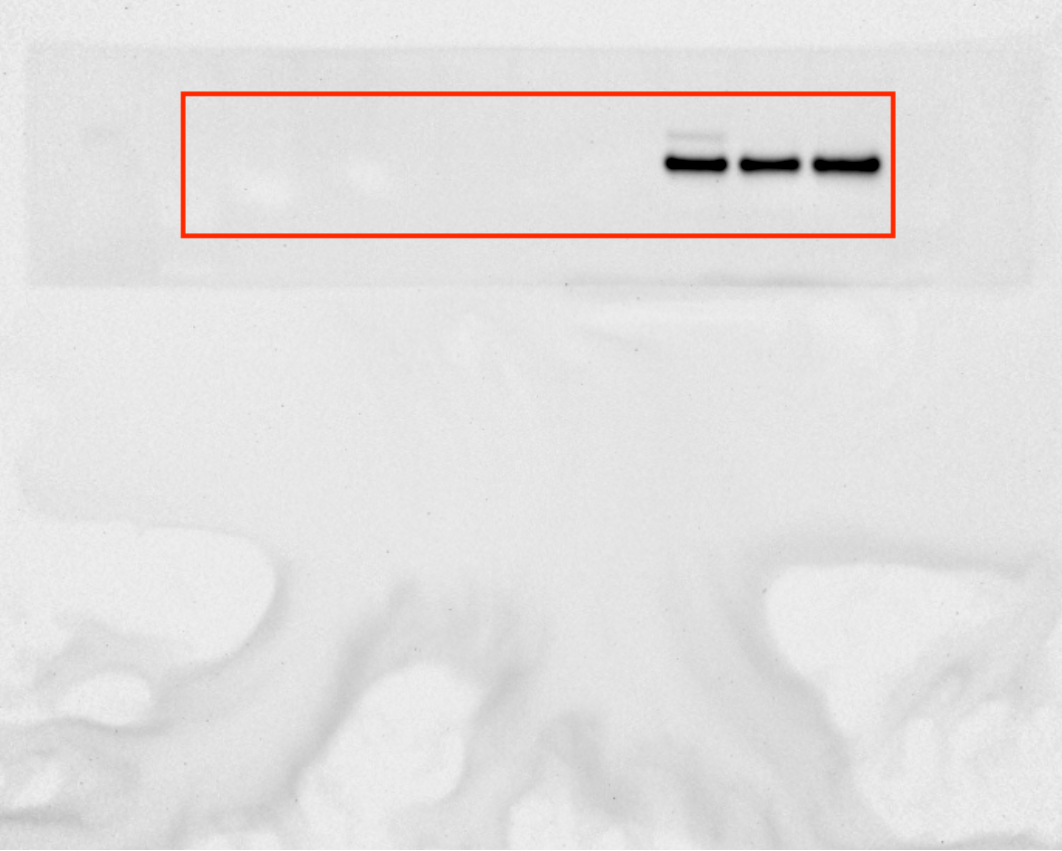

Supplement: Supplementary file 3 — Source data Fig. 1 [file 44319_2024_181_MOESM3_ESM.zip › Figure 1/Figure 1C/HA-PPTC7.tif]

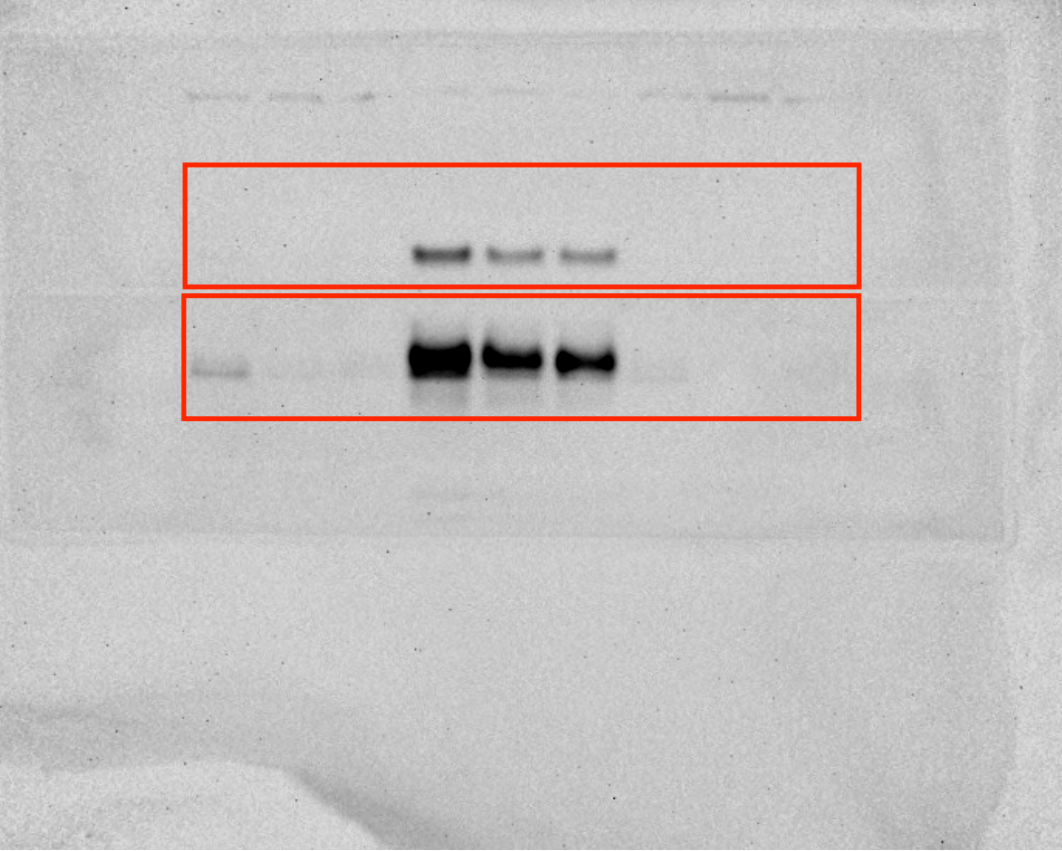

Supplement: Supplementary file 3 — Source data Fig. 1 [file 44319_2024_181_MOESM3_ESM.zip › Figure 1/Figure 1C/NIX-top_BNIP3-bottom.tif]

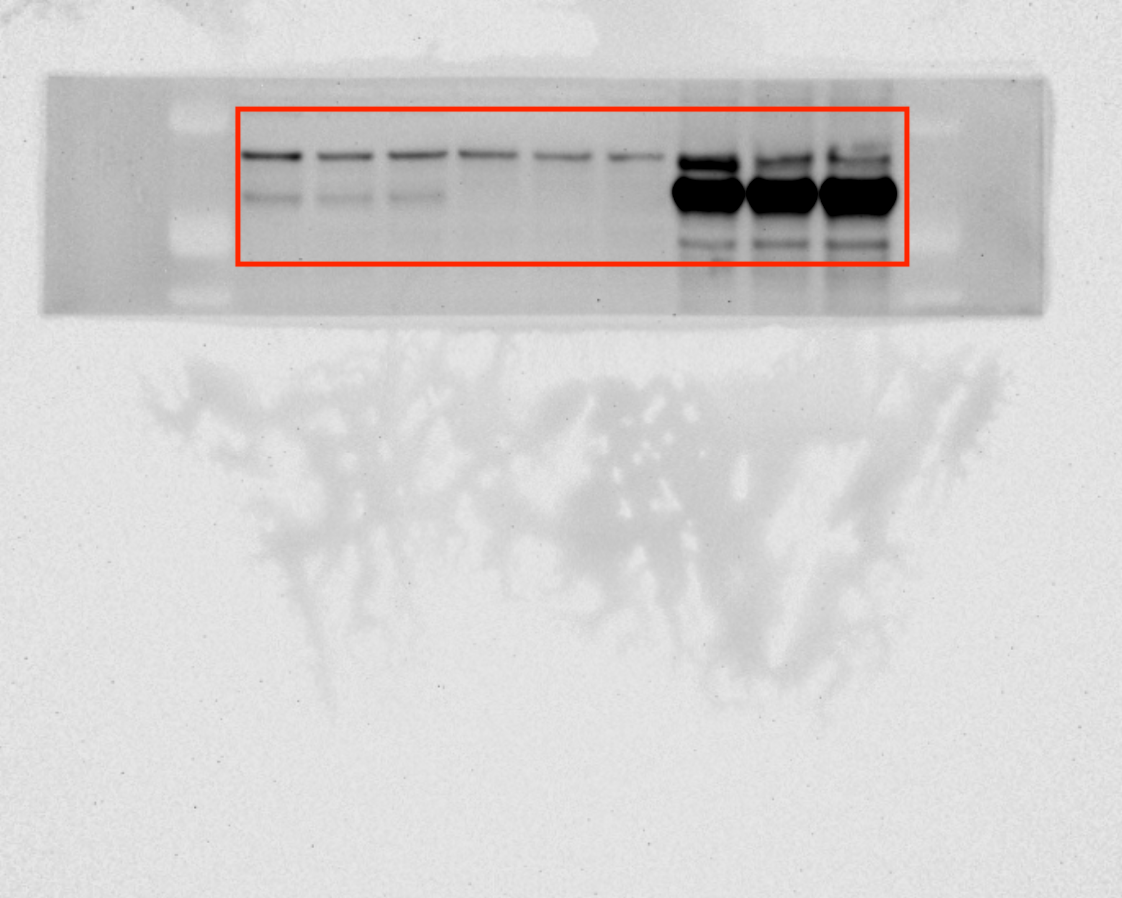

Supplement: Supplementary file 3 — Source data Fig. 1 [file 44319_2024_181_MOESM3_ESM.zip › Figure 1/Figure 1C/PPTC7.tif]

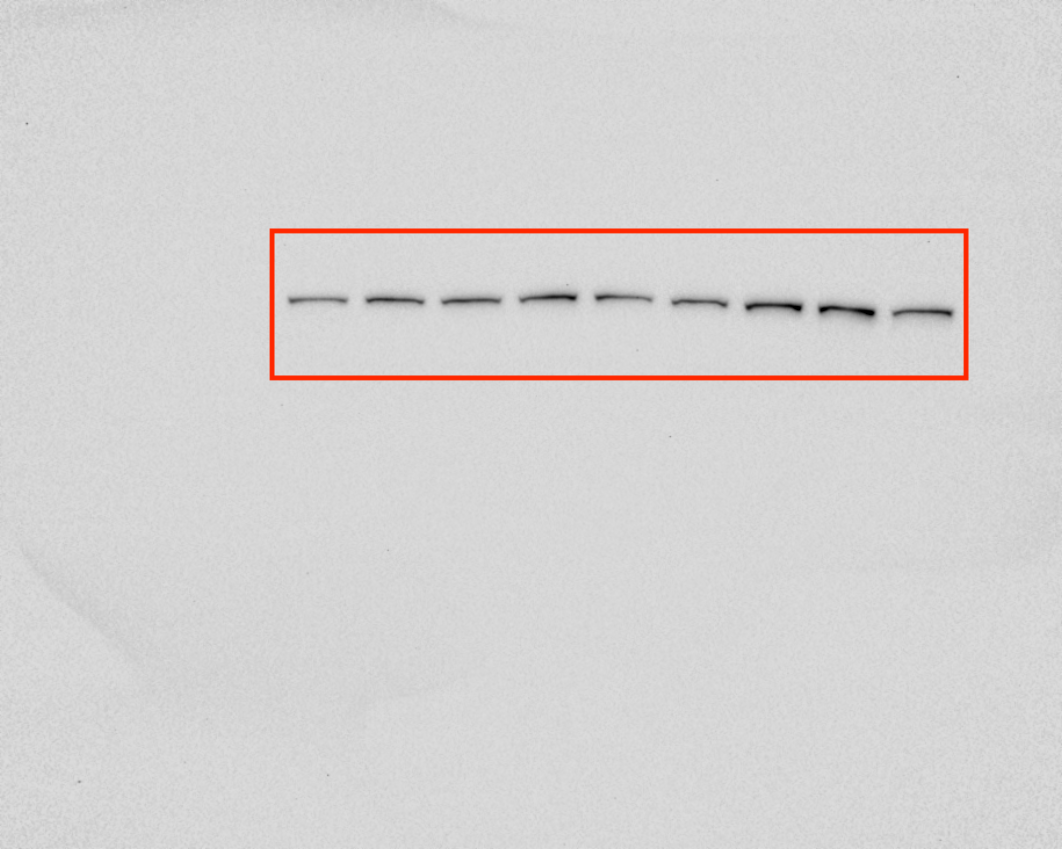

Supplement: Supplementary file 3 — Source data Fig. 1 [file 44319_2024_181_MOESM3_ESM.zip › Figure 1/Figure 1C/VCL.tif]

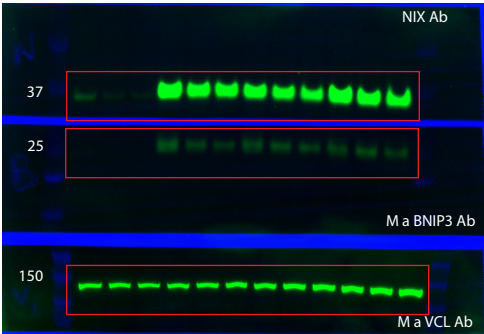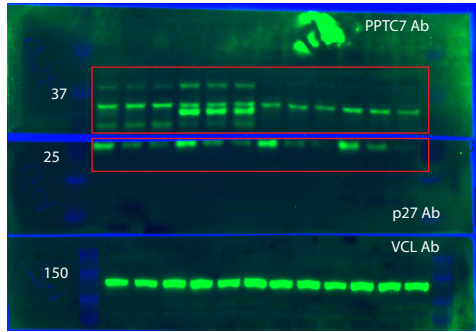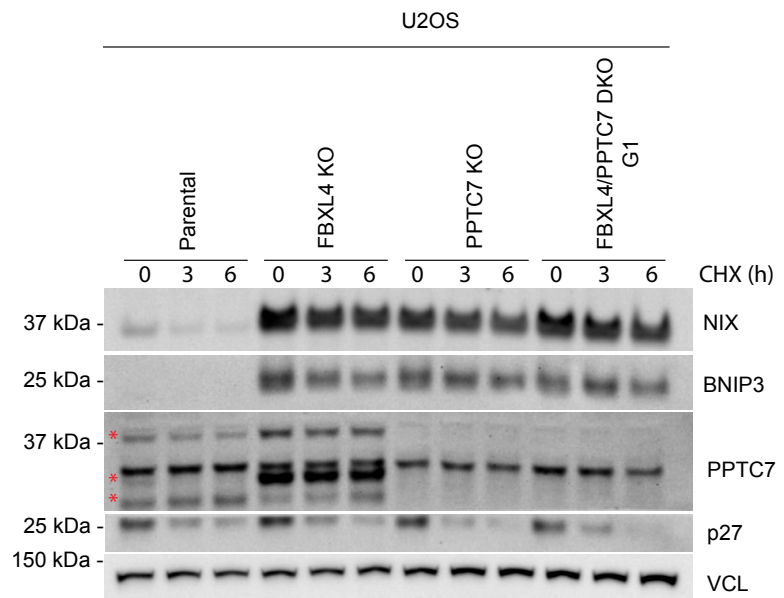

Supplement: Supplementary file 3 — Source data Fig. 1 [file 44319_2024_181_MOESM3_ESM.zip › Figure 1/Figure 1D/Annotation Figure 1D.pdf]

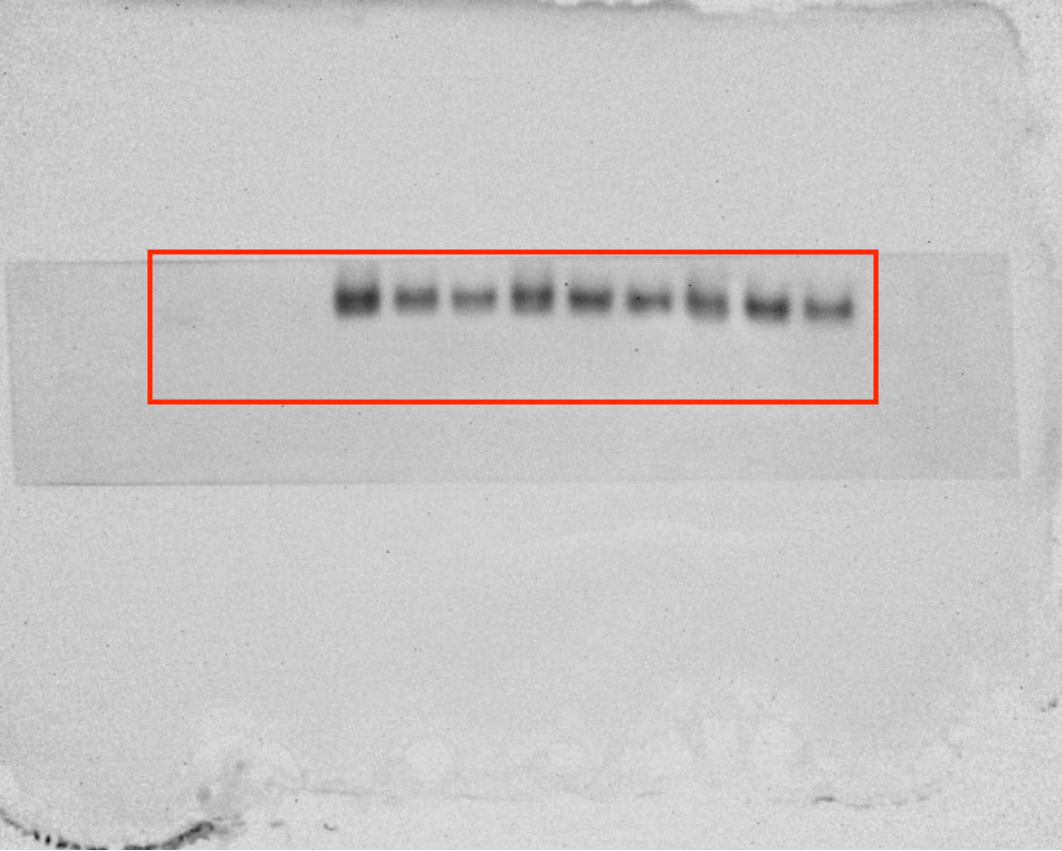

Supplement: Supplementary file 3 — Source data Fig. 1 [file 44319_2024_181_MOESM3_ESM.zip › Figure 1/Figure 1D/BNIP3.tif]

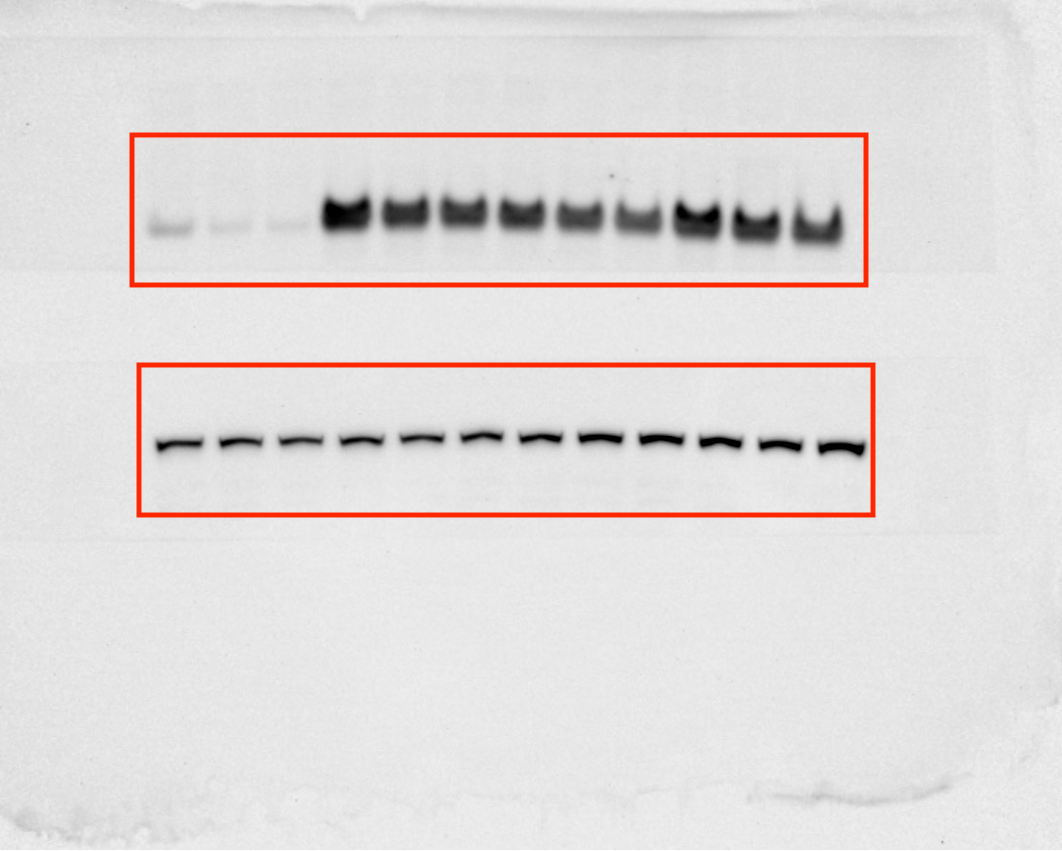

Supplement: Supplementary file 3 — Source data Fig. 1 [file 44319_2024_181_MOESM3_ESM.zip › Figure 1/Figure 1D/NIX-top_VCL-bottom.tif]

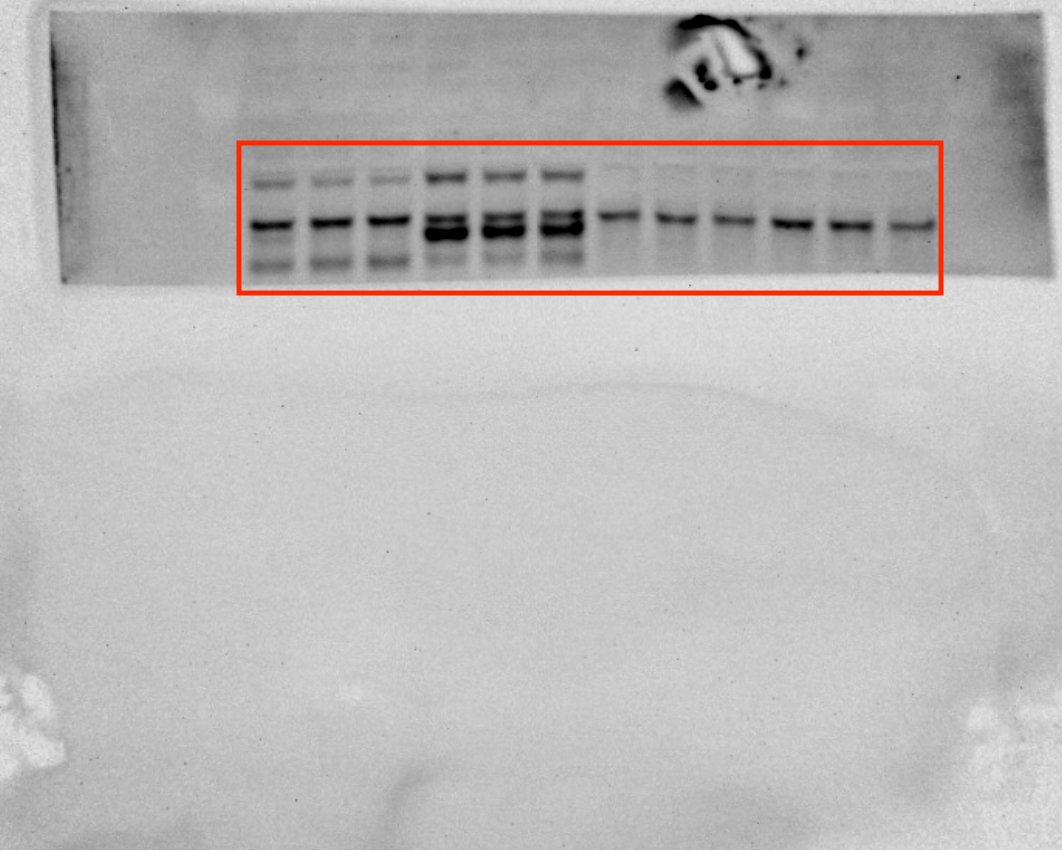

Supplement: Supplementary file 3 — Source data Fig. 1 [file 44319_2024_181_MOESM3_ESM.zip › Figure 1/Figure 1D/PPTC7.tif]

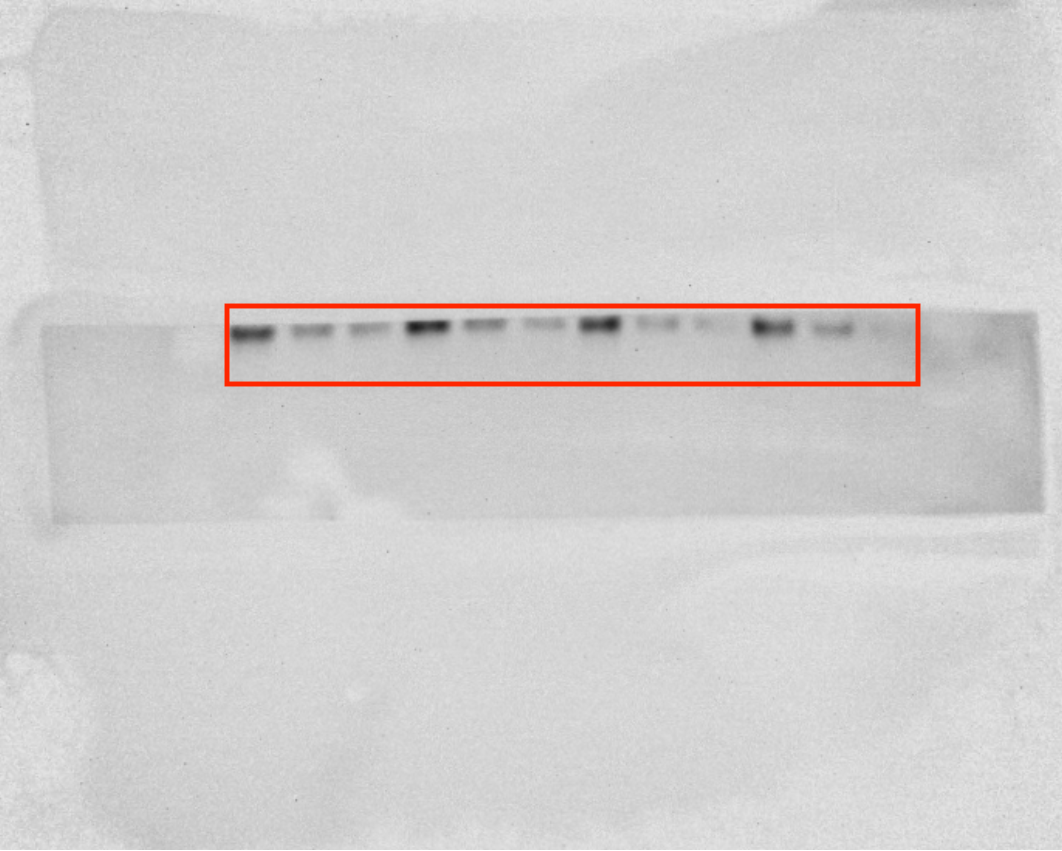

Supplement: Supplementary file 3 — Source data Fig. 1 [file 44319_2024_181_MOESM3_ESM.zip › Figure 1/Figure 1D/p27.tif]

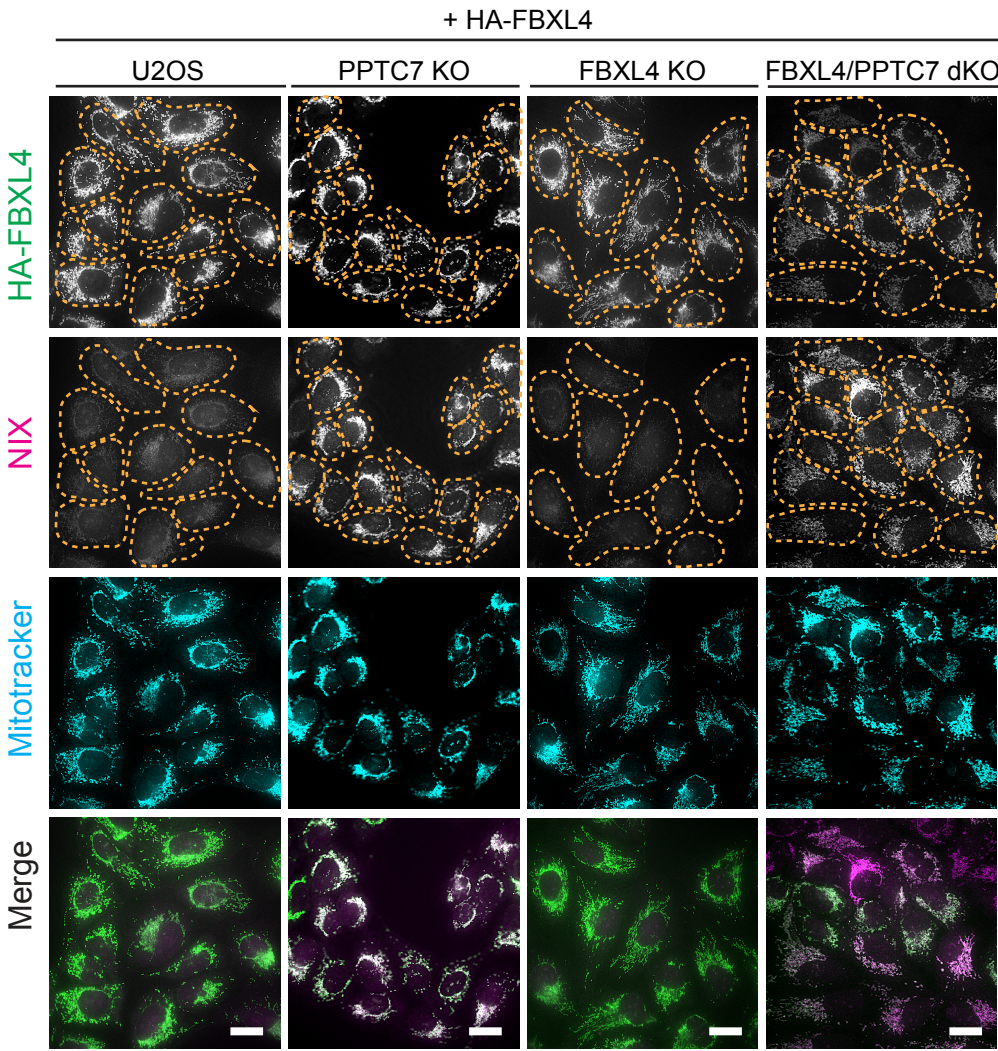

Supplement: Supplementary file 3 — Source data Fig. 1 [file 44319_2024_181_MOESM3_ESM.zip › Figure 1/Figure 1E/Annotation Figure 1E.pdf]

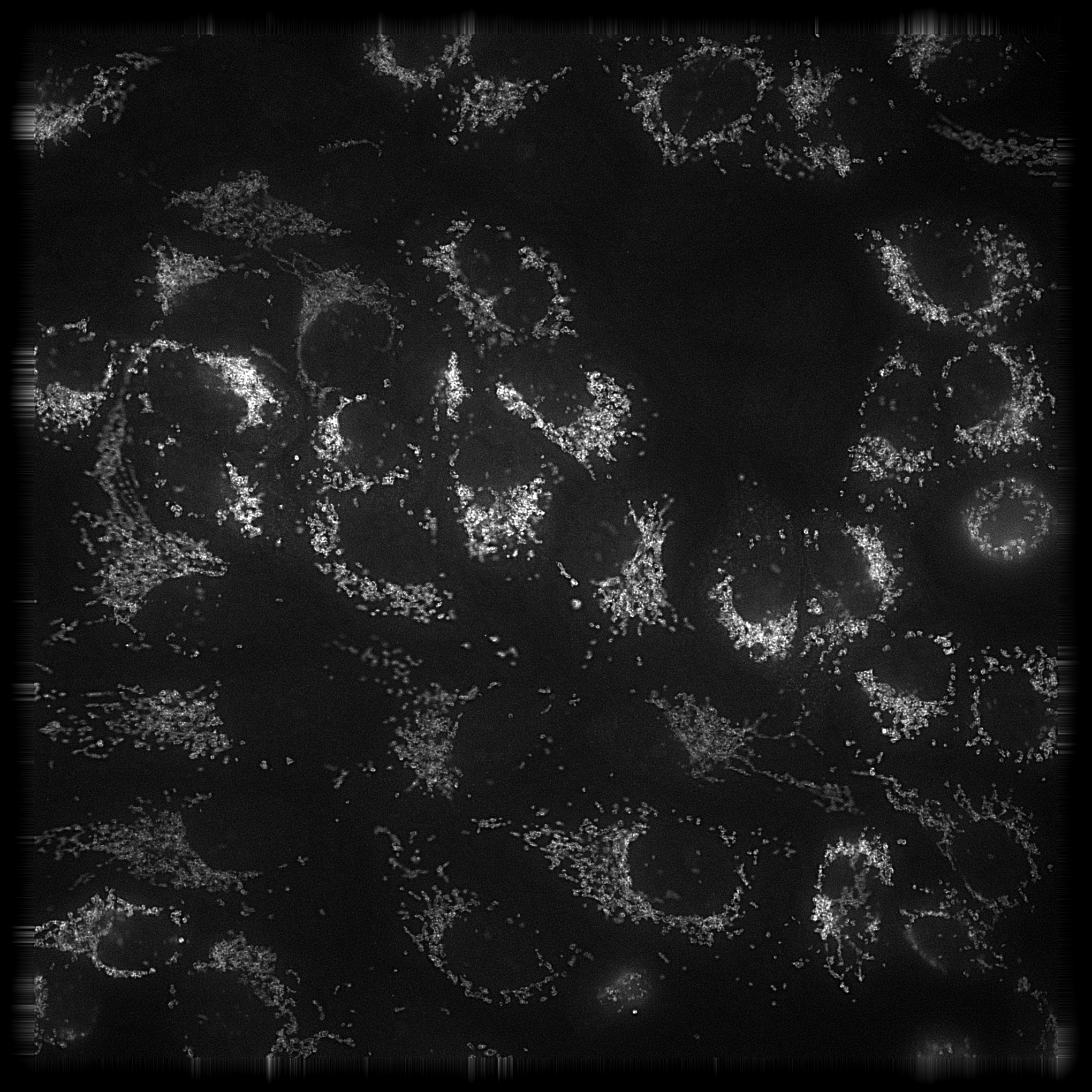

Supplement: Supplementary file 3 — Source data Fig. 1 [file 44319_2024_181_MOESM3_ESM.zip › Figure 1/Figure 1E/DKO_fbxl4ha_HA488_NIX555_deepMR.tif]

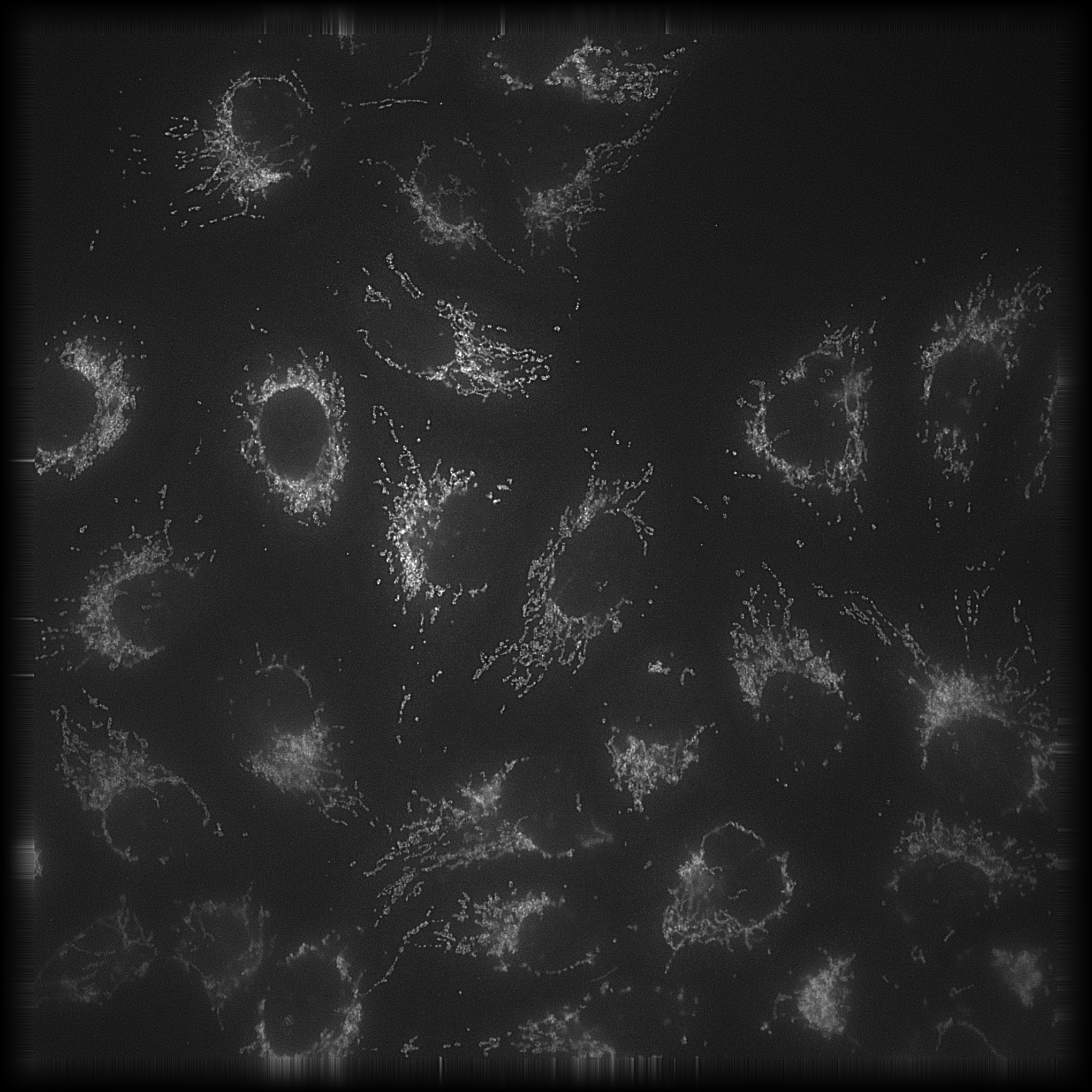

Supplement: Supplementary file 3 — Source data Fig. 1 [file 44319_2024_181_MOESM3_ESM.zip › Figure 1/Figure 1E/FBXL4KO_fbxl4ha_HA488_NIX555_deepMR.tif]

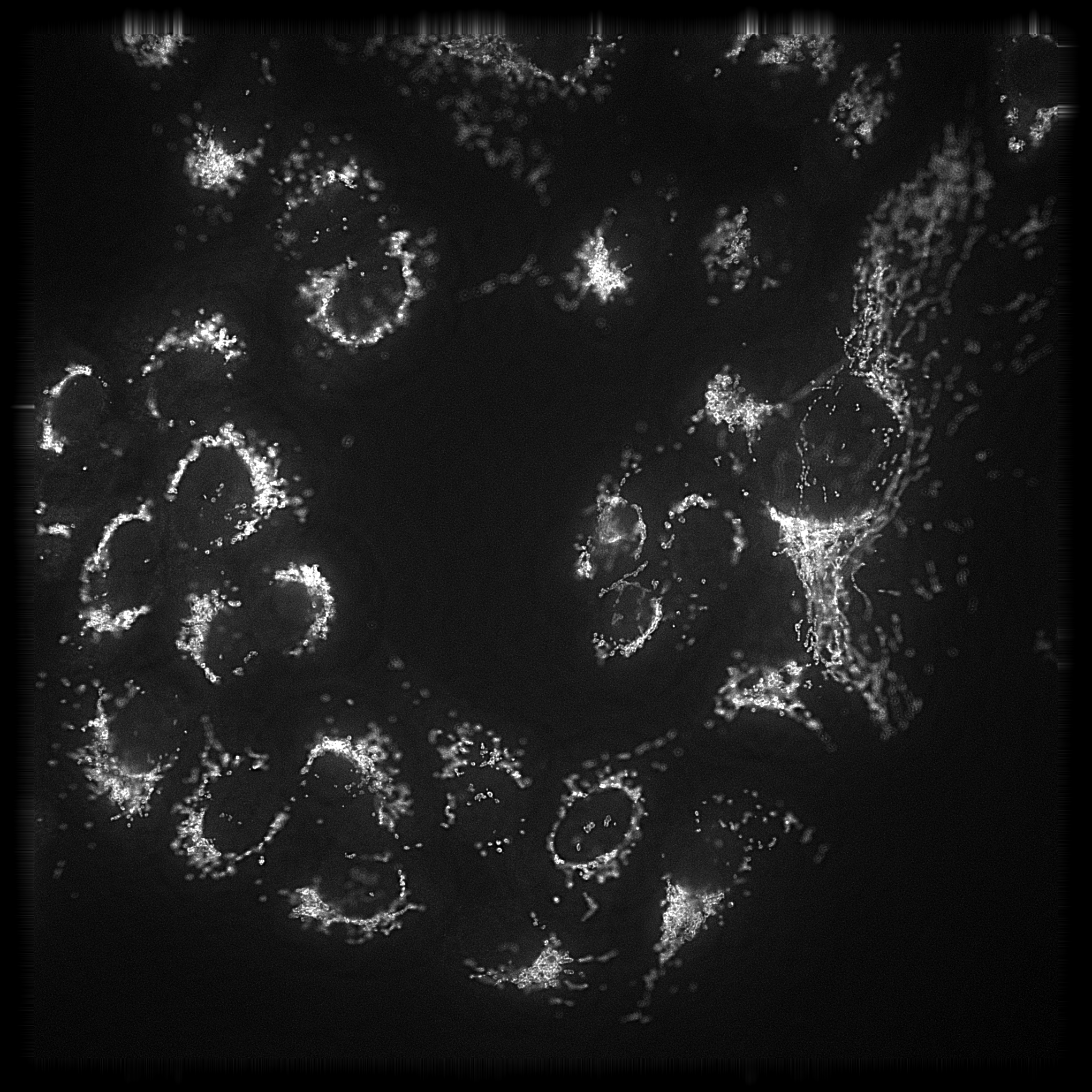

Supplement: Supplementary file 3 — Source data Fig. 1 [file 44319_2024_181_MOESM3_ESM.zip › Figure 1/Figure 1E/pptc7KO_fbxl4ha_HA488_NIX555_deepMR.tif]

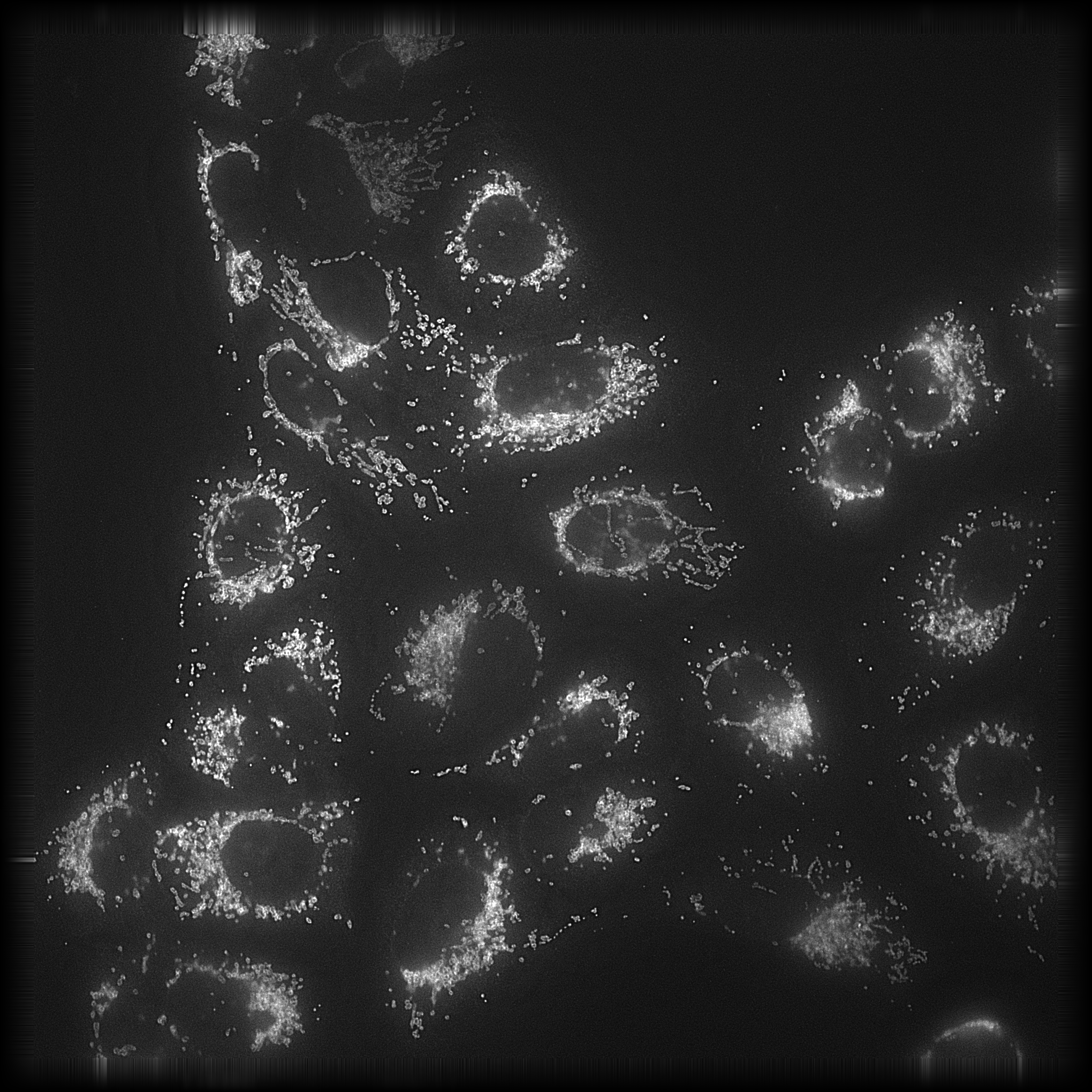

Supplement: Supplementary file 3 — Source data Fig. 1 [file 44319_2024_181_MOESM3_ESM.zip › Figure 1/Figure 1E/u2os_fbxl4ha_HA488_NIX555_deepMR.tif]

Scale bar = 20um

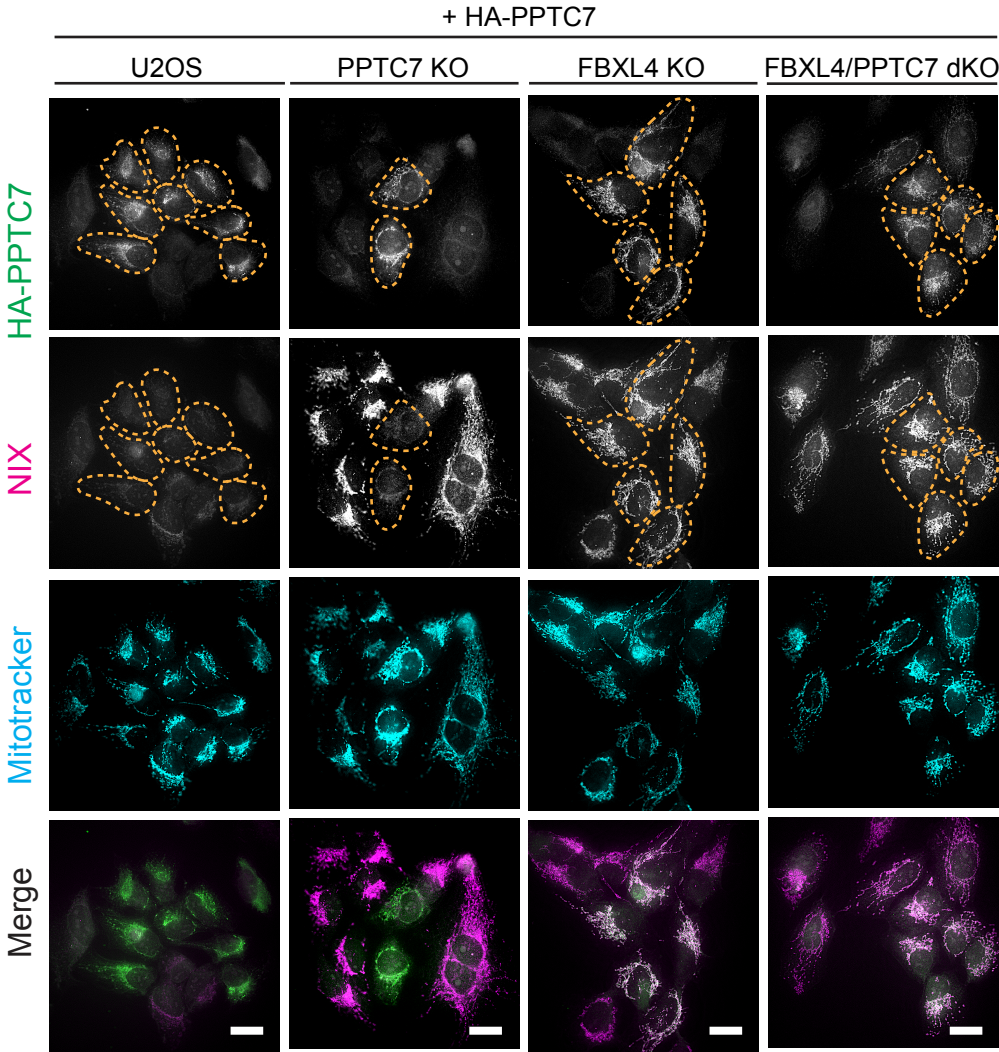

Supplement: Supplementary file 3 — Source data Fig. 1 [file 44319_2024_181_MOESM3_ESM.zip › Figure 1/Figure 1F/Annotation Figure 1F.pdf]

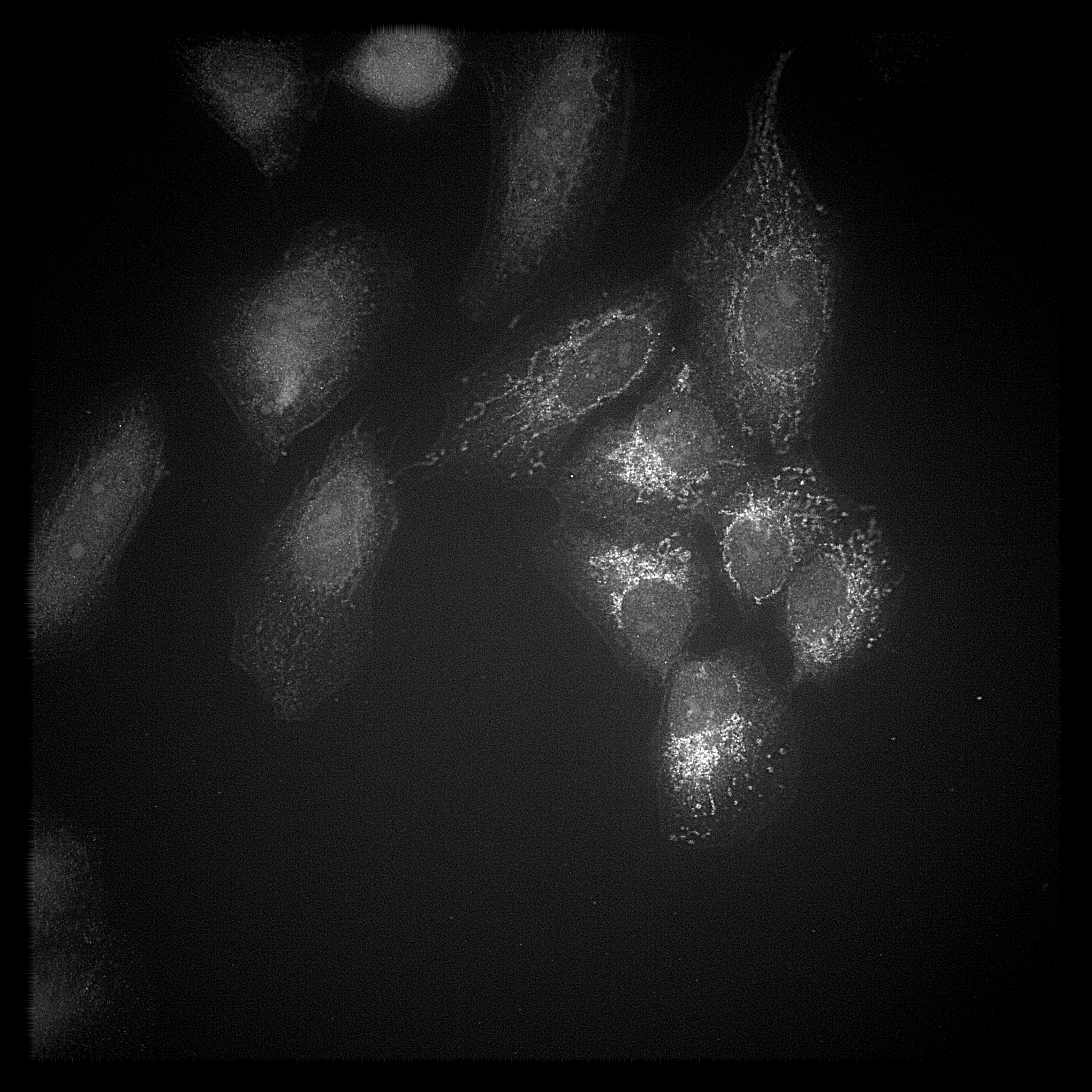

Supplement: Supplementary file 3 — Source data Fig. 1 [file 44319_2024_181_MOESM3_ESM.zip › Figure 1/Figure 1F/DKO_pptc7ha_HA488_NIX555_deepMR.tif]

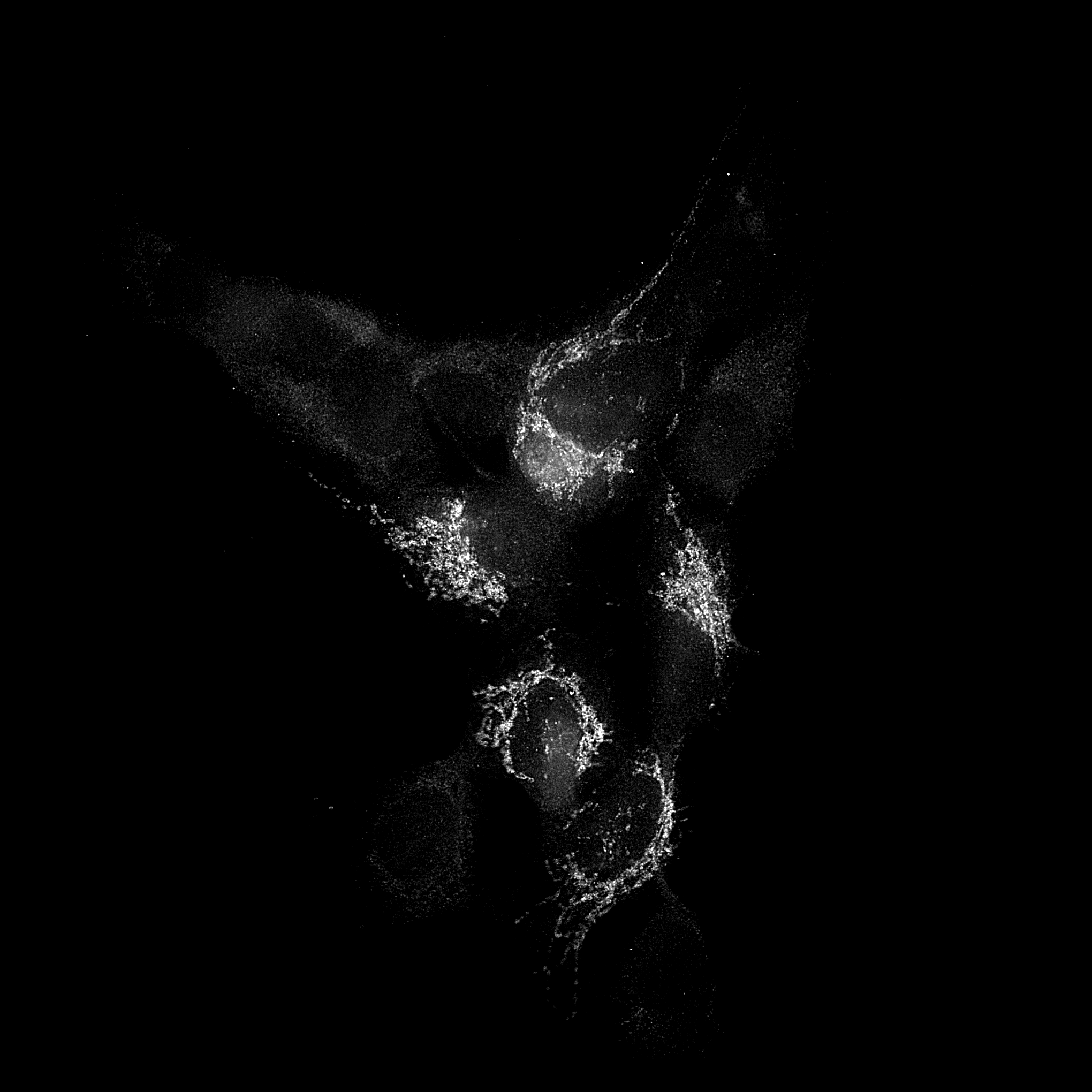

Supplement: Supplementary file 3 — Source data Fig. 1 [file 44319_2024_181_MOESM3_ESM.zip › Figure 1/Figure 1F/fbxl4ko_pptc7ha_HA488_NIX555_deepMR.tif]

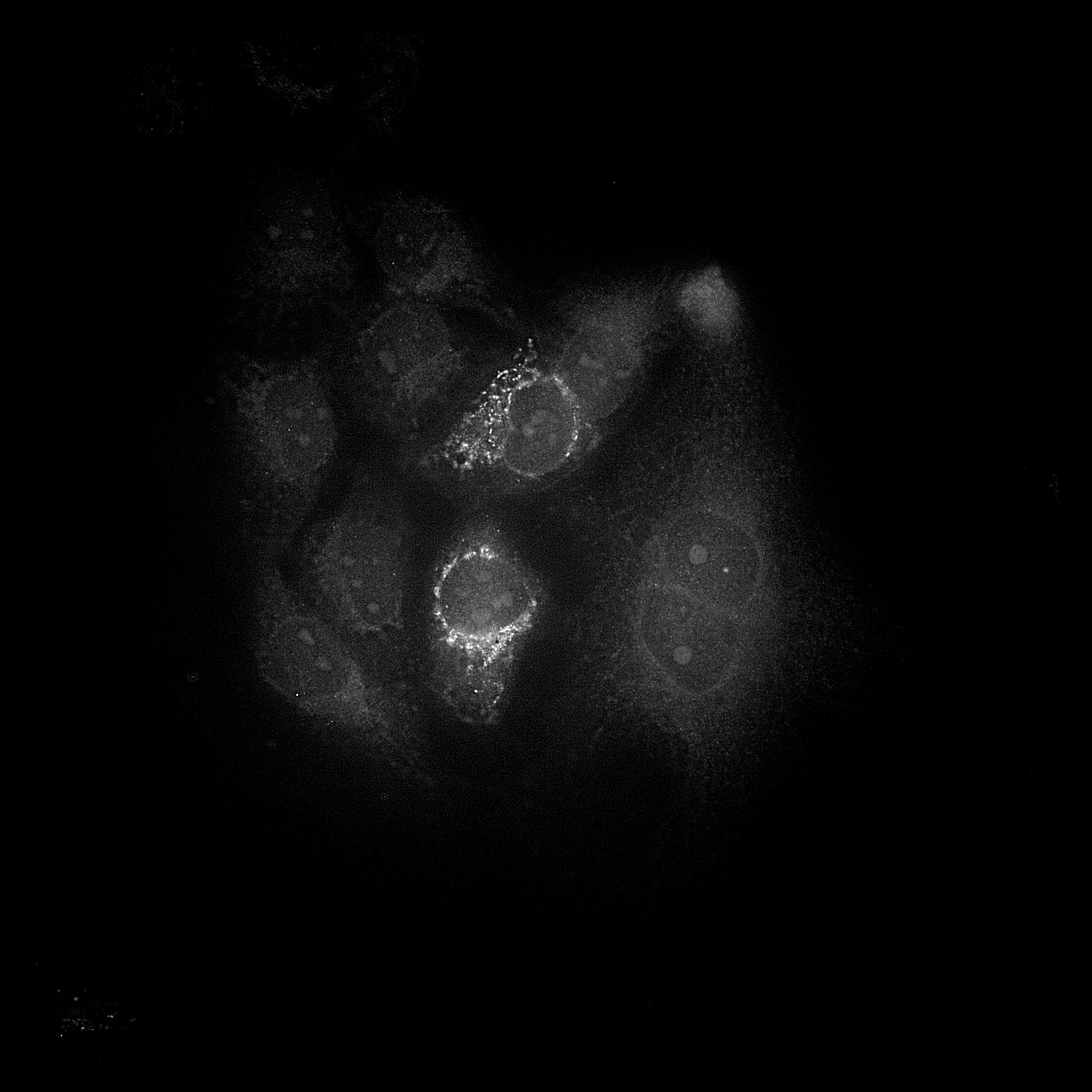

Supplement: Supplementary file 3 — Source data Fig. 1 [file 44319_2024_181_MOESM3_ESM.zip › Figure 1/Figure 1F/pptc7ko_pptc7ha_HA488_NIX555_deepMR.tif]

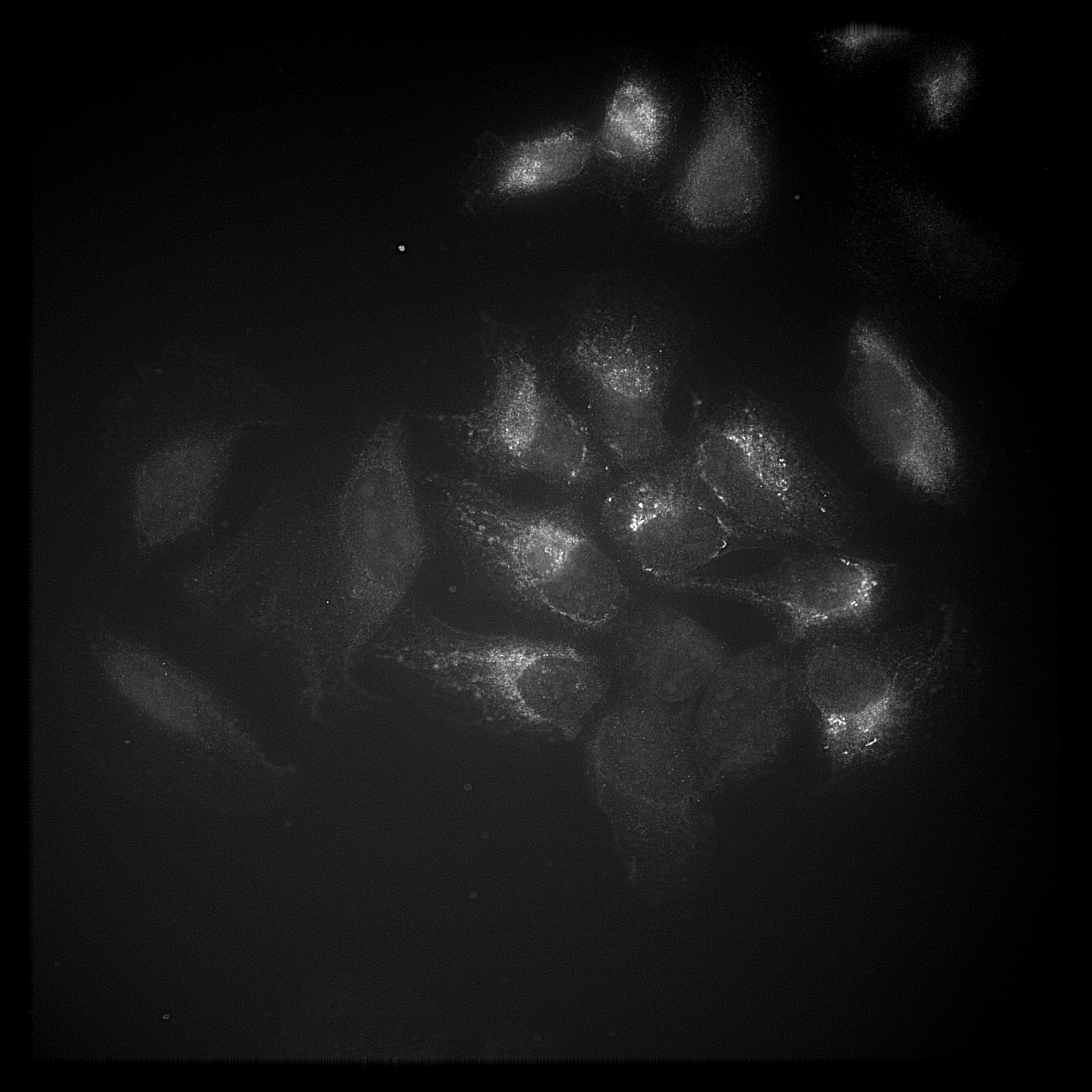

Supplement: Supplementary file 3 — Source data Fig. 1 [file 44319_2024_181_MOESM3_ESM.zip › Figure 1/Figure 1F/u2os_pptc7ha_HA488_NIX555_deepMR.tif]

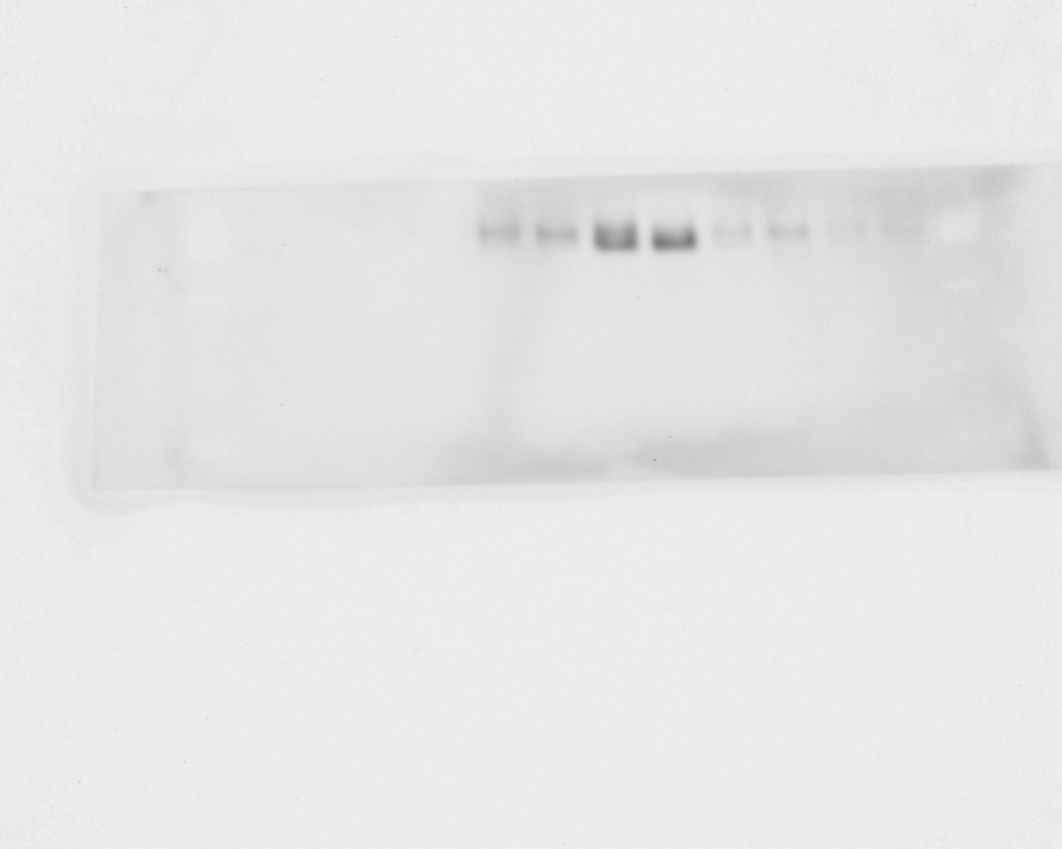

Supplement: Supplementary file 3 — Source data Fig. 1 [file 44319_2024_181_MOESM3_ESM.zip › Figure 1/Figure 1G/(BNIP3) CL.tif]

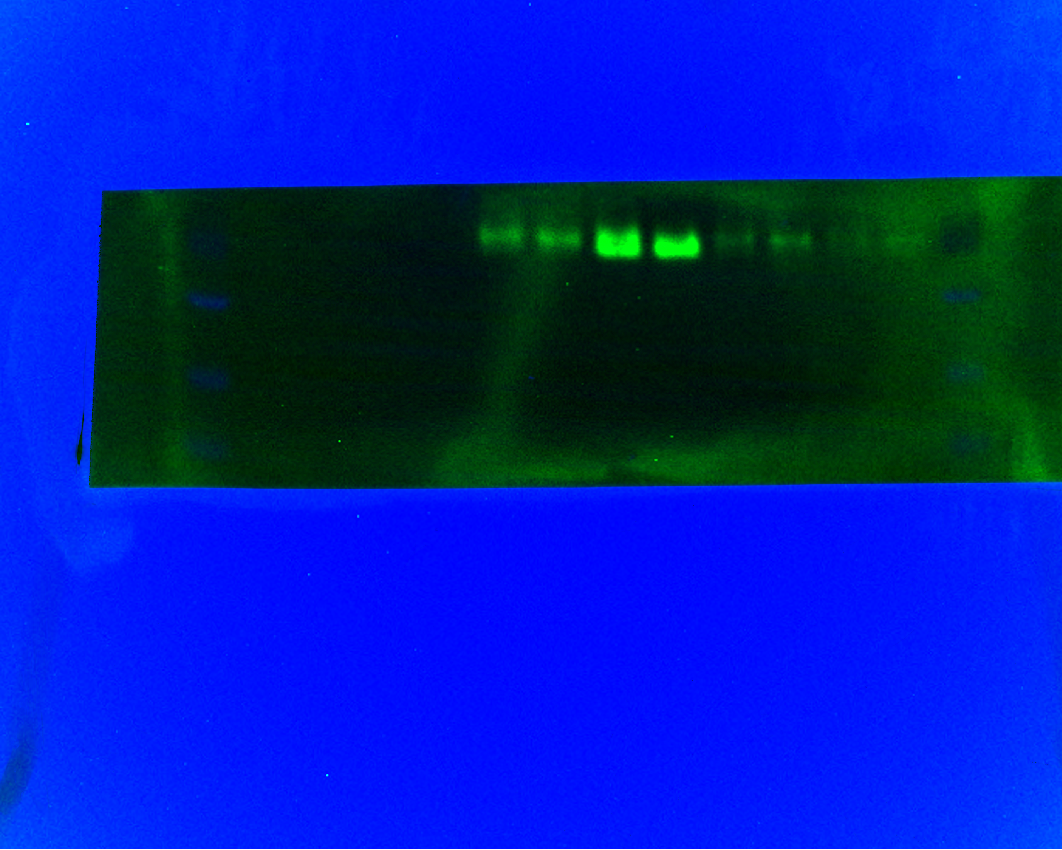

Supplement: Supplementary file 3 — Source data Fig. 1 [file 44319_2024_181_MOESM3_ESM.zip › Figure 1/Figure 1G/(BNIP3).tif]

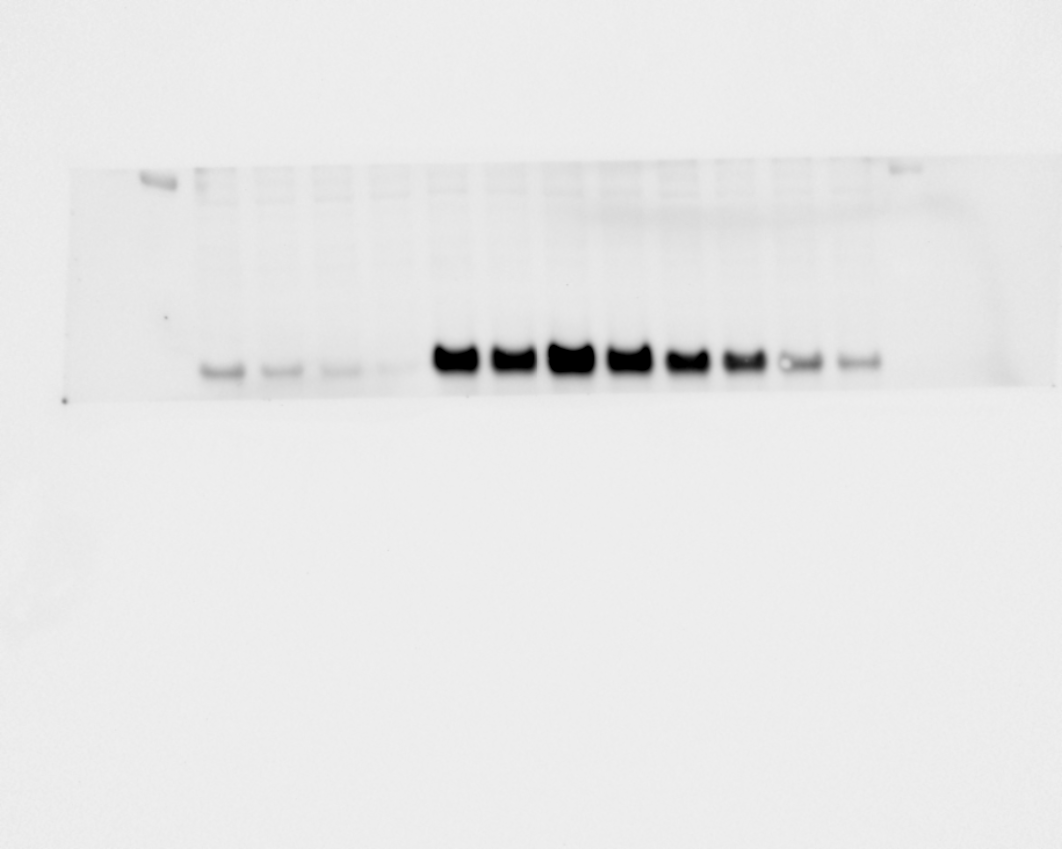

Supplement: Supplementary file 3 — Source data Fig. 1 [file 44319_2024_181_MOESM3_ESM.zip › Figure 1/Figure 1G/(NIX) (Chemiluminescence).tif]

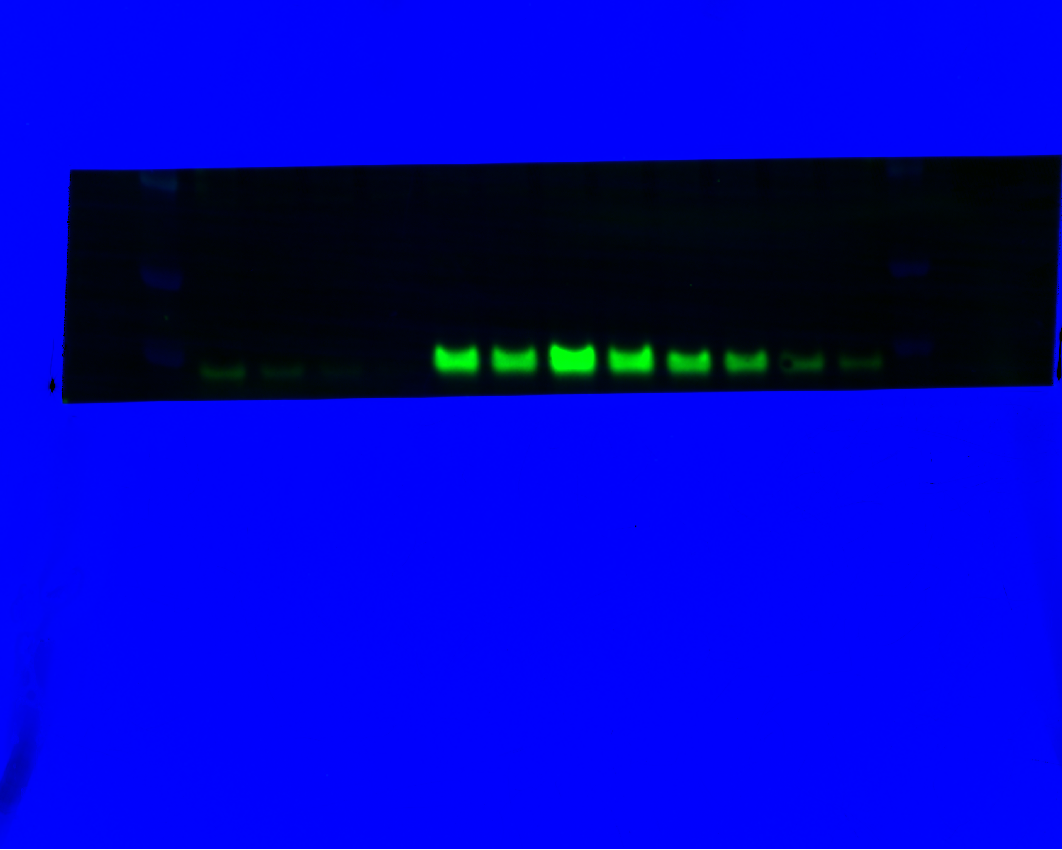

Supplement: Supplementary file 3 — Source data Fig. 1 [file 44319_2024_181_MOESM3_ESM.zip › Figure 1/Figure 1G/(NIX)(Composite).tif]

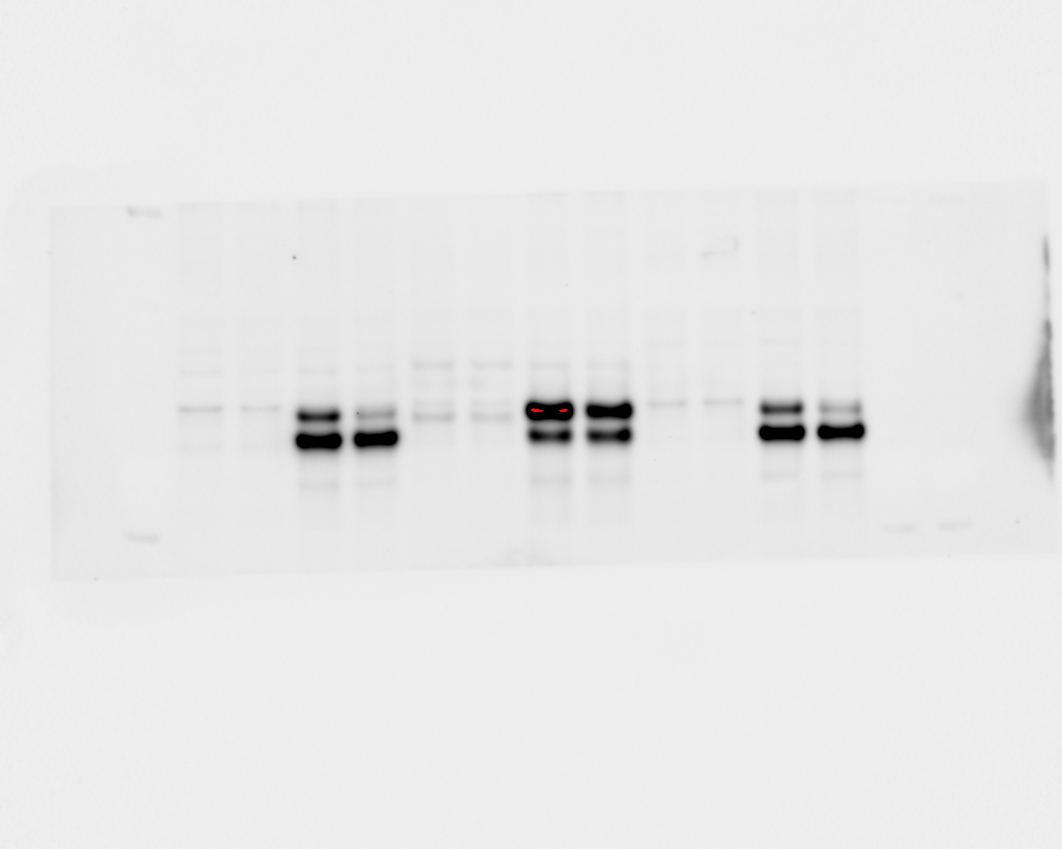

Supplement: Supplementary file 3 — Source data Fig. 1 [file 44319_2024_181_MOESM3_ESM.zip › Figure 1/Figure 1G/(PPTC7) (Chemiluminescence).tif]

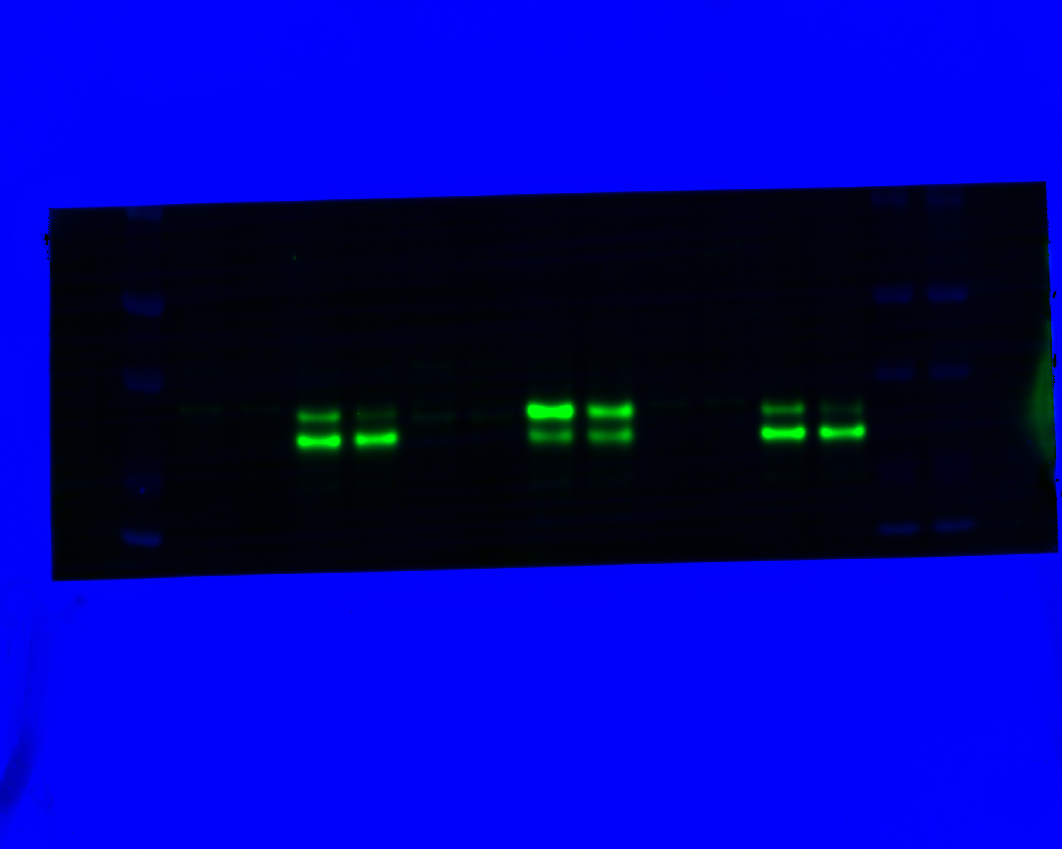

Supplement: Supplementary file 3 — Source data Fig. 1 [file 44319_2024_181_MOESM3_ESM.zip › Figure 1/Figure 1G/(PPTC7) (Composite).tif]

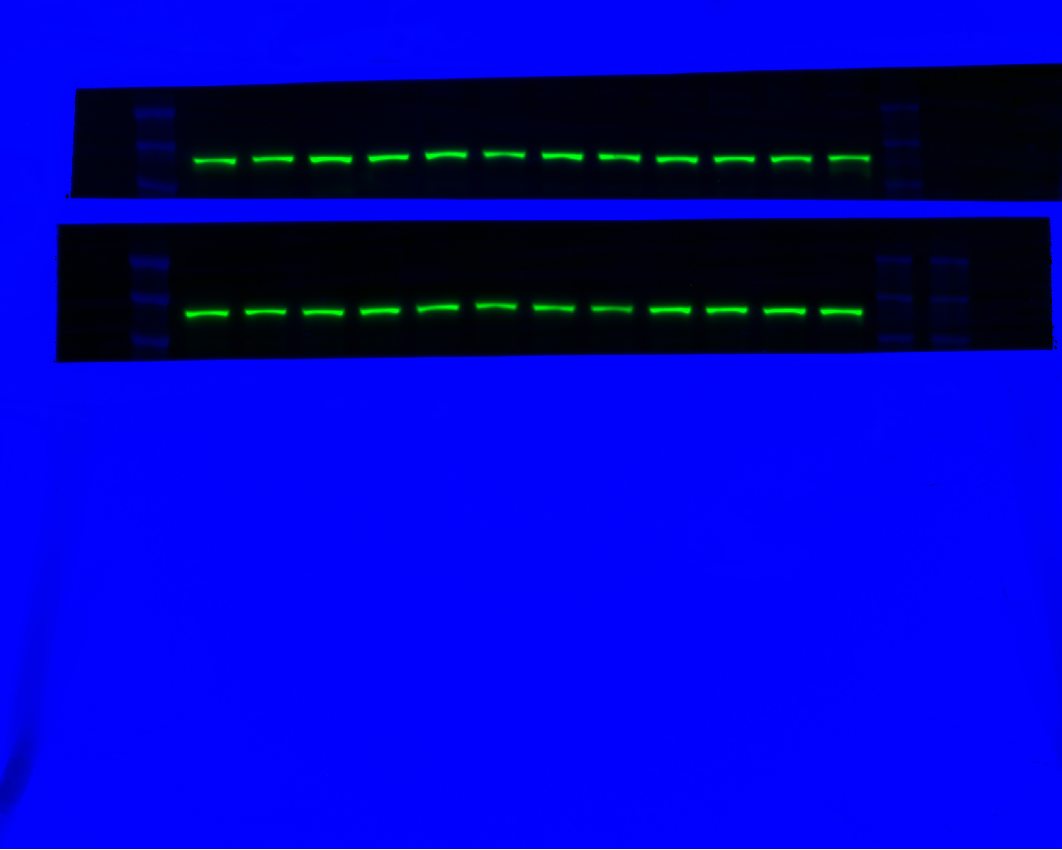

Supplement: Supplementary file 3 — Source data Fig. 1 [file 44319_2024_181_MOESM3_ESM.zip › Figure 1/Figure 1G/(Vinculin) (Composite).tif]

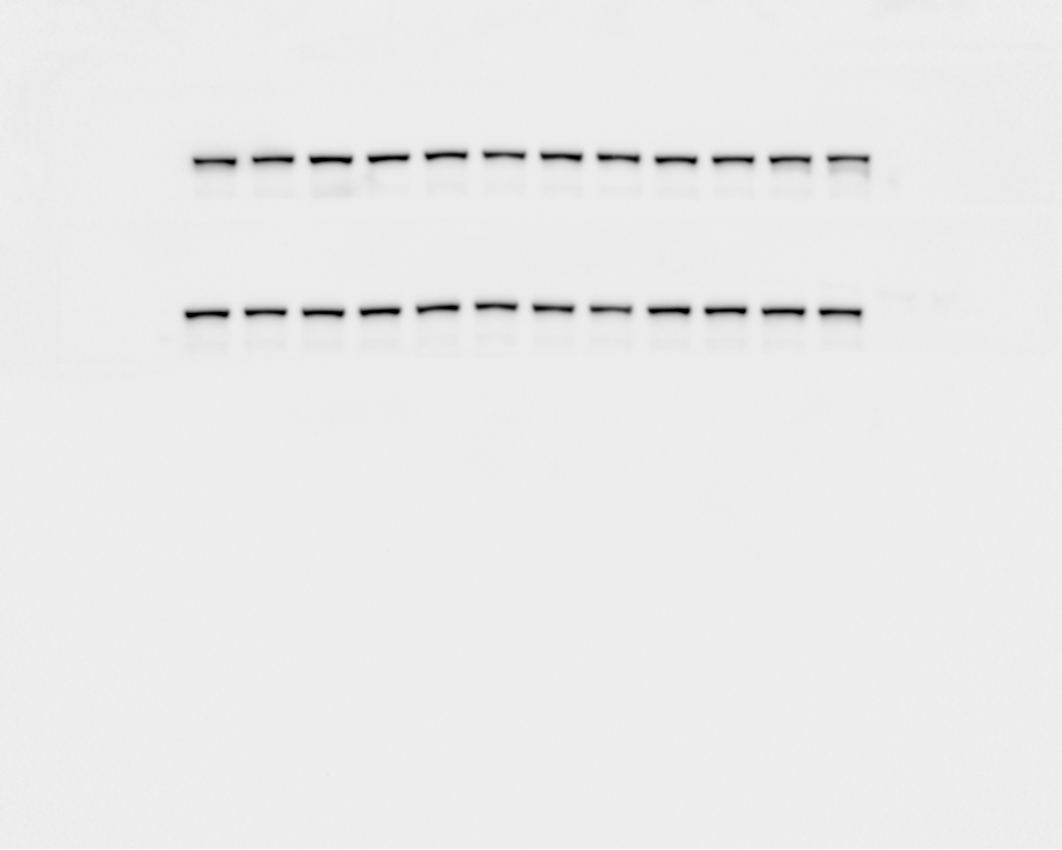

Supplement: Supplementary file 3 — Source data Fig. 1 [file 44319_2024_181_MOESM3_ESM.zip › Figure 1/Figure 1G/(Vinculin)(Chemiluminescence).tif]

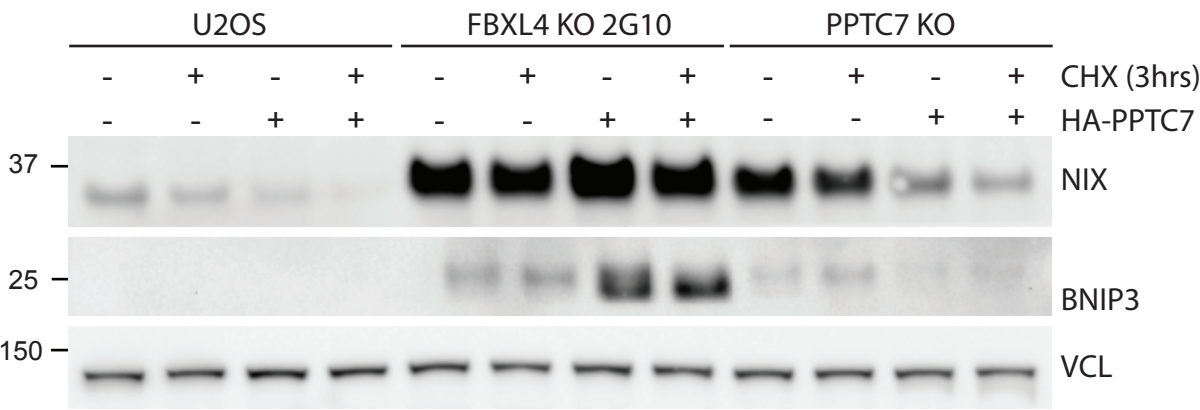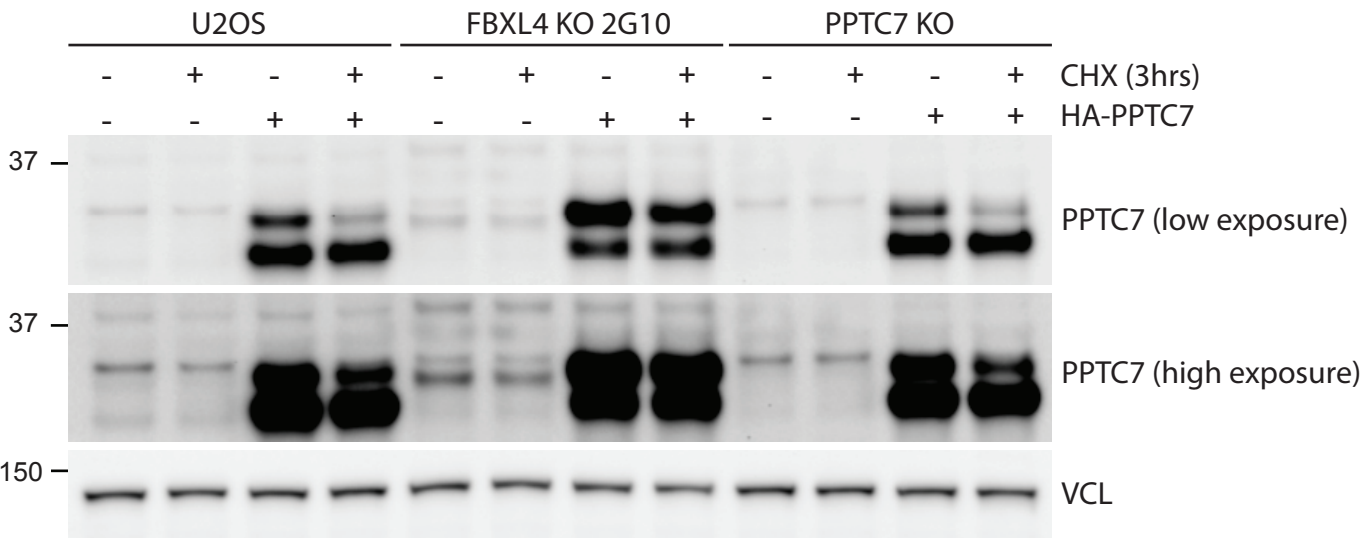

Supplement: Supplementary file 3 — Source data Fig. 1 [file 44319_2024_181_MOESM3_ESM.zip › Figure 1/Figure 1G/Annotation Figure 1G.pdf]

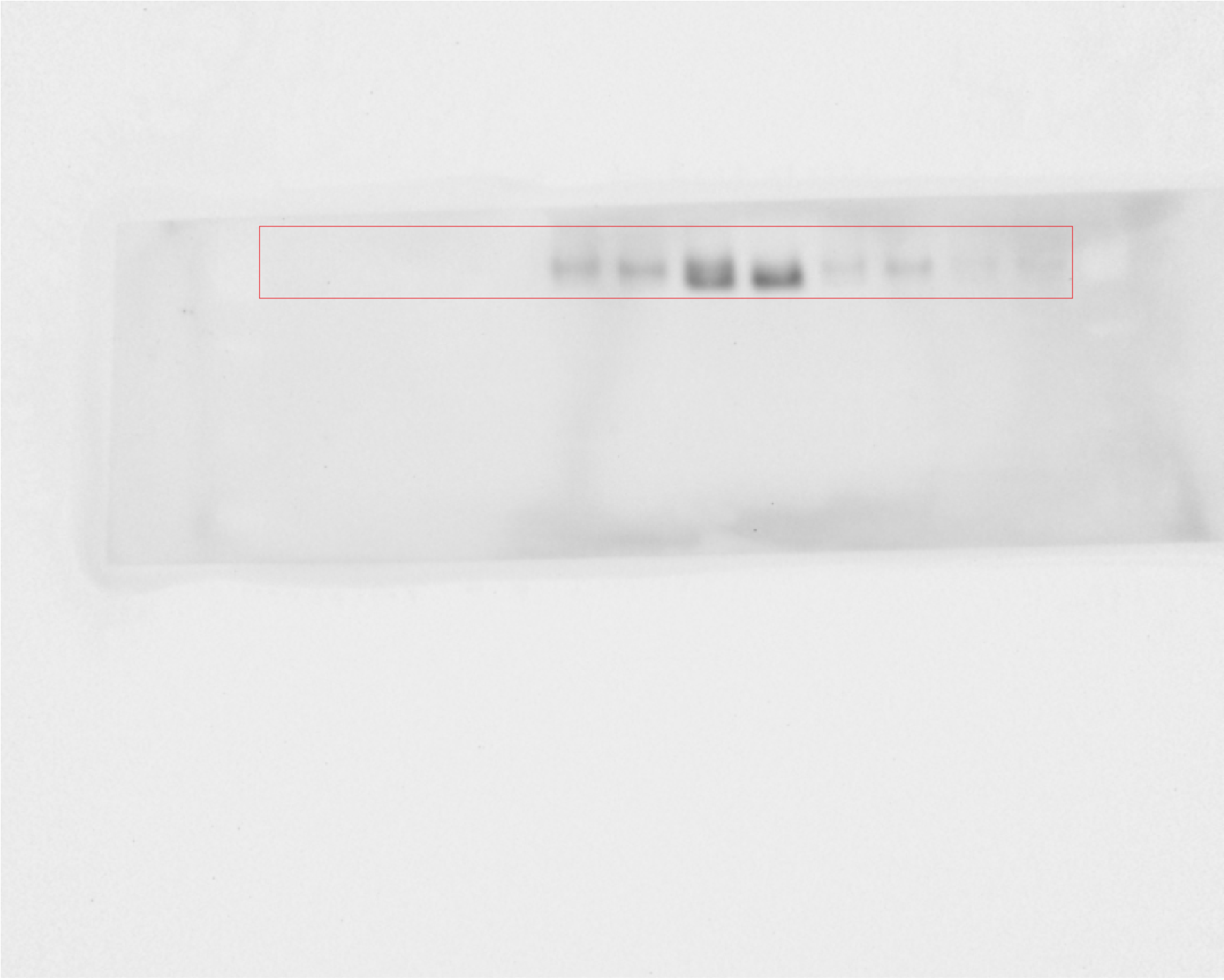

Supplement: Supplementary file 3 — Source data Fig. 1 [file 44319_2024_181_MOESM3_ESM.zip › Figure 1/Figure 1G/BNIP3.png]

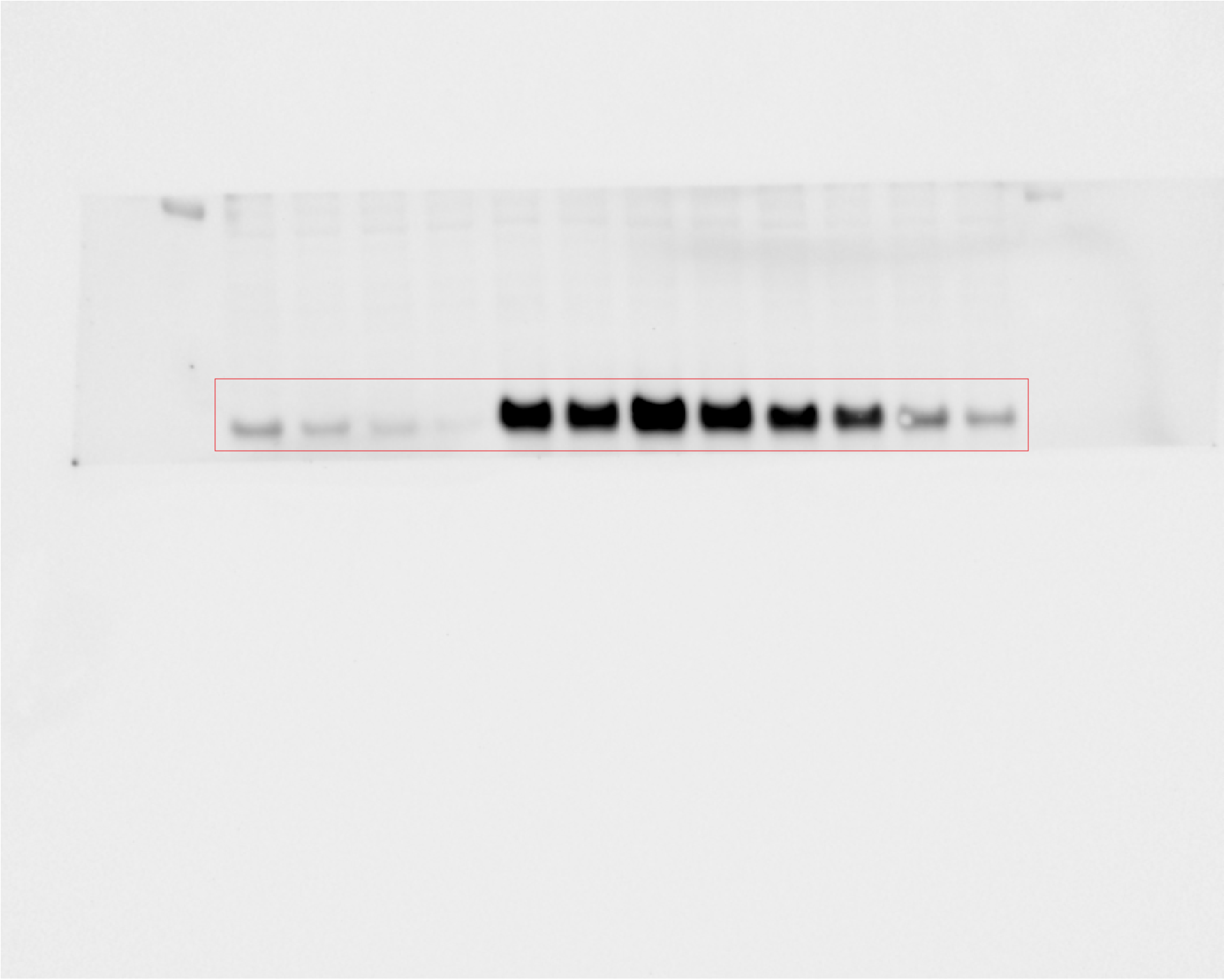

Supplement: Supplementary file 3 — Source data Fig. 1 [file 44319_2024_181_MOESM3_ESM.zip › Figure 1/Figure 1G/NIX.png]

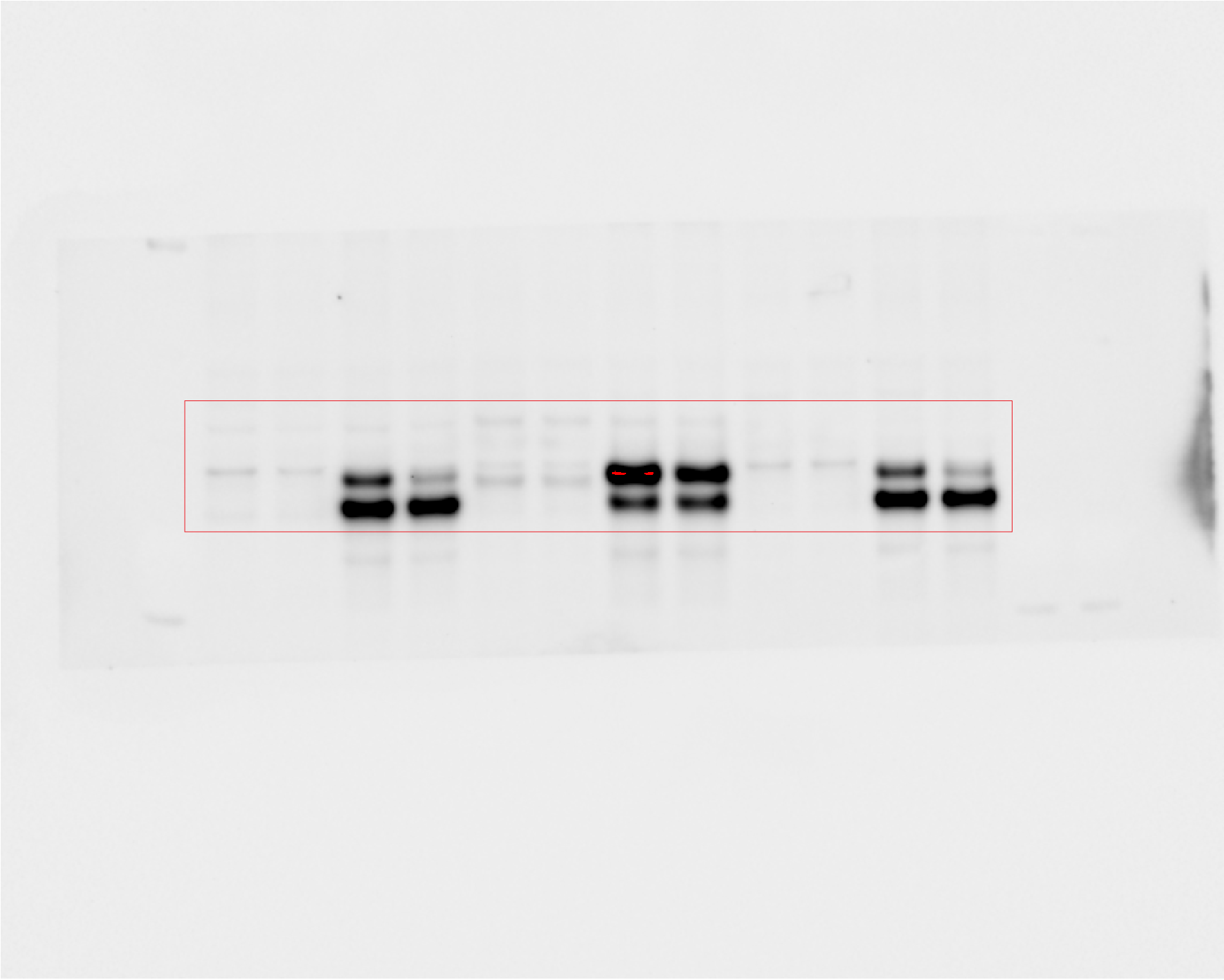

Supplement: Supplementary file 3 — Source data Fig. 1 [file 44319_2024_181_MOESM3_ESM.zip › Figure 1/Figure 1G/PPTC7 2.png]

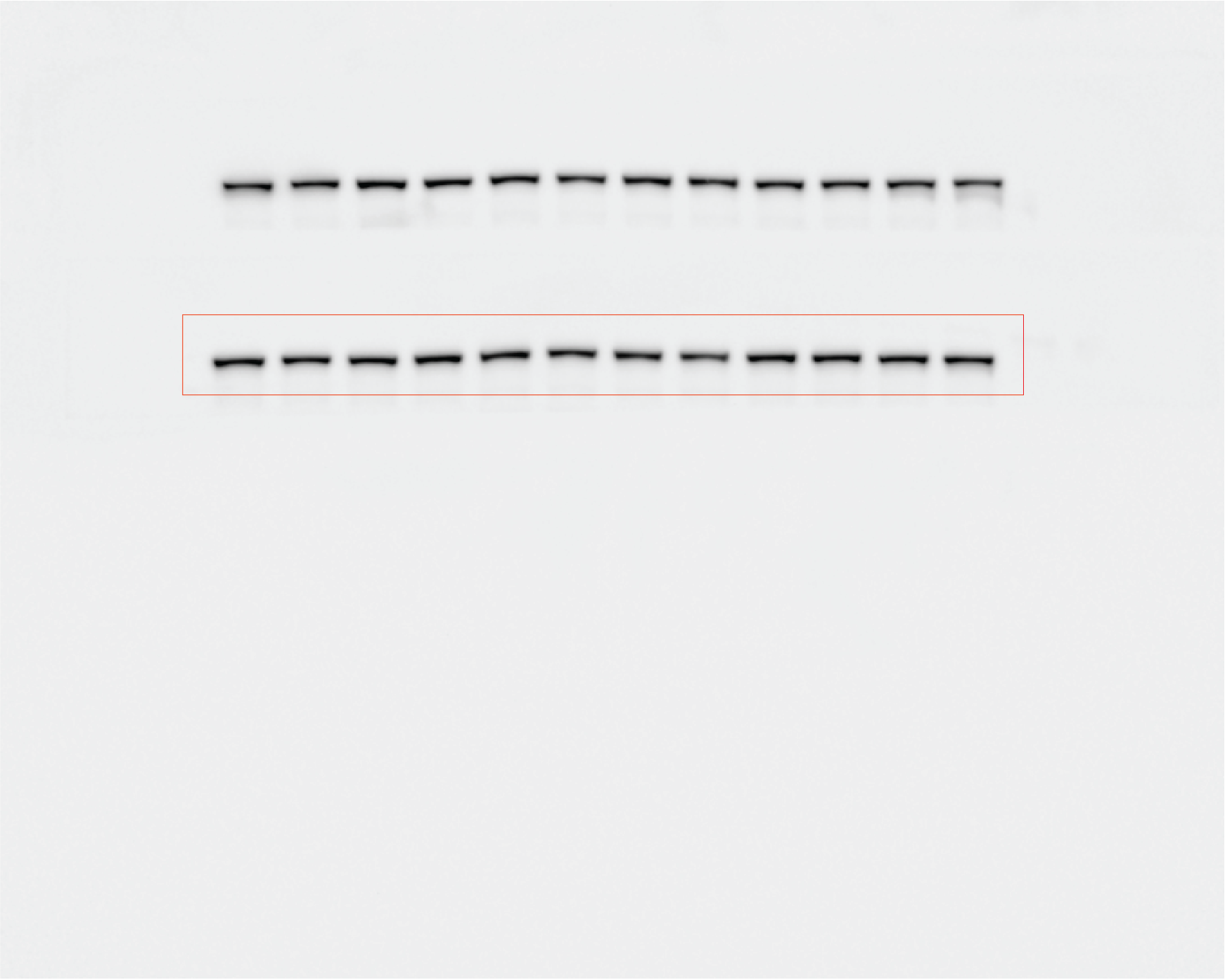

Supplement: Supplementary file 3 — Source data Fig. 1 [file 44319_2024_181_MOESM3_ESM.zip › Figure 1/Figure 1G/Vinculin.png]

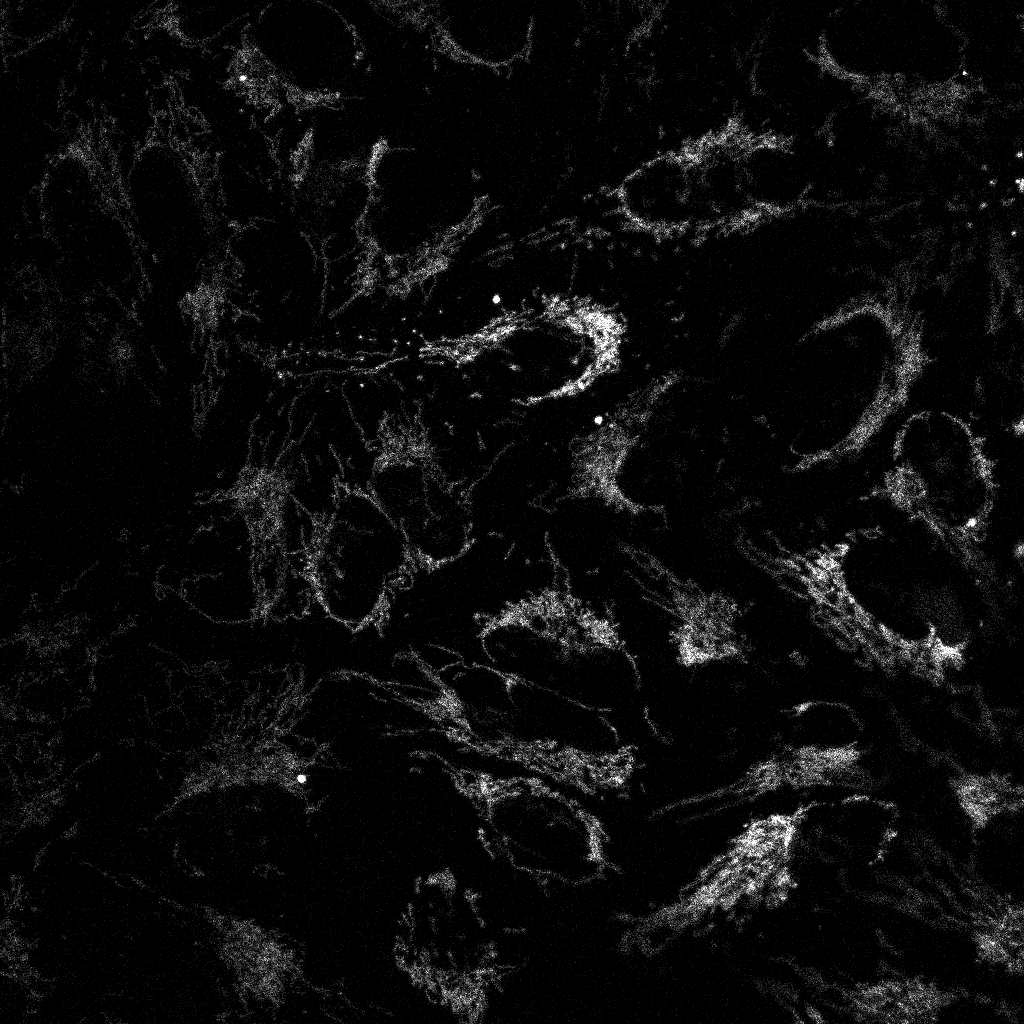

Supplement: Supplementary file 3 — Source data Fig. 1 [file 44319_2024_181_MOESM3_ESM.zip › Figure 1/Figure 1H/ U2OS.tif]

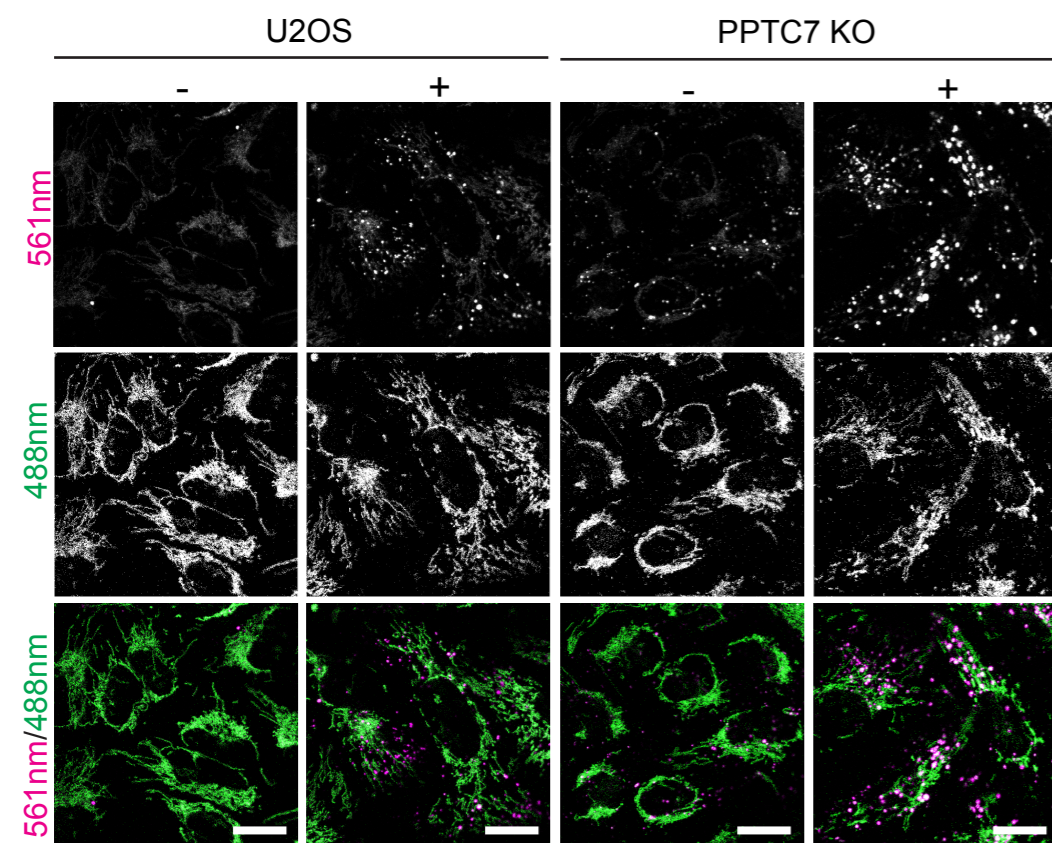

DFP (24hrs)

Scale bar = 20um

Supplement: Supplementary file 3 — Source data Fig. 1 [file 44319_2024_181_MOESM3_ESM.zip › Figure 1/Figure 1H/Annotation Figure 1H.pdf]

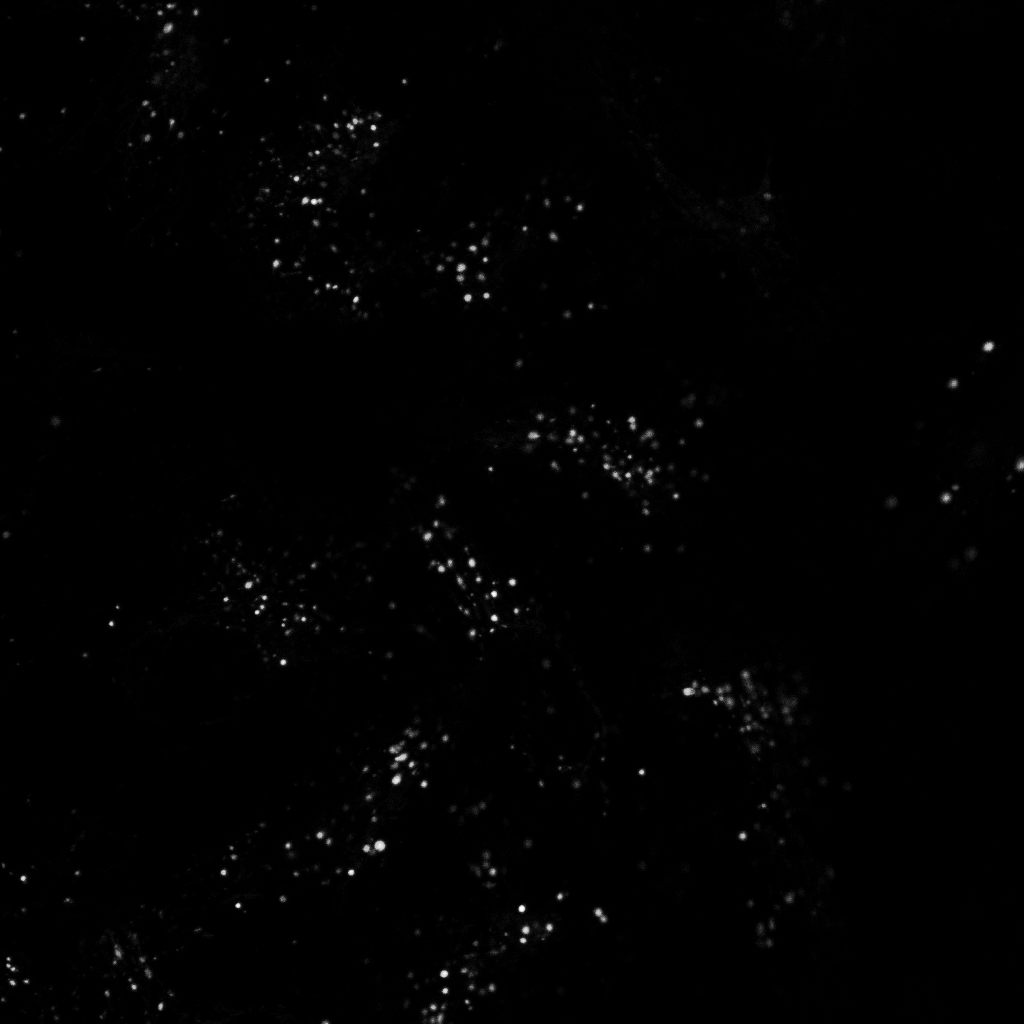

Supplement: Supplementary file 3 — Source data Fig. 1 [file 44319_2024_181_MOESM3_ESM.zip › Figure 1/Figure 1H/PPTC7 KO + 24hrs DFP.tif]

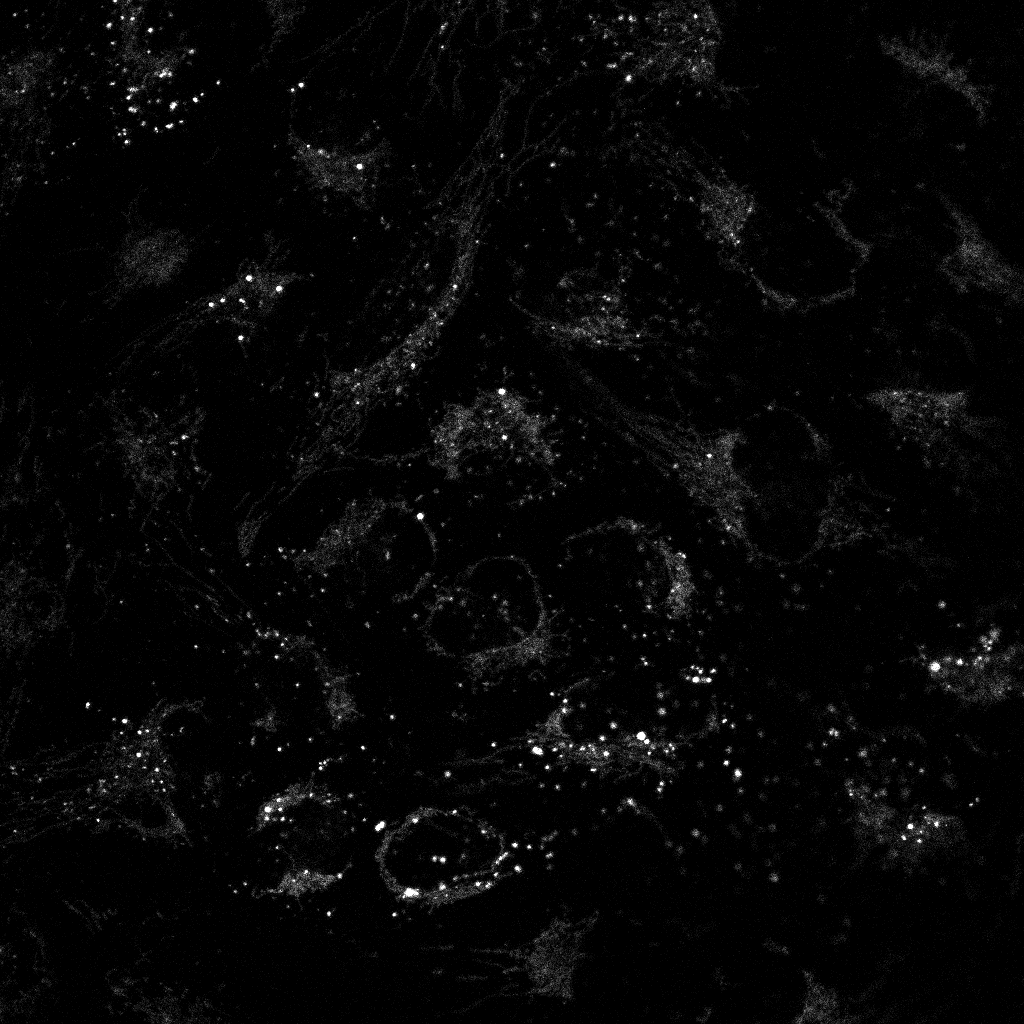

Supplement: Supplementary file 3 — Source data Fig. 1 [file 44319_2024_181_MOESM3_ESM.zip › Figure 1/Figure 1H/PPTC7 KO.tif]

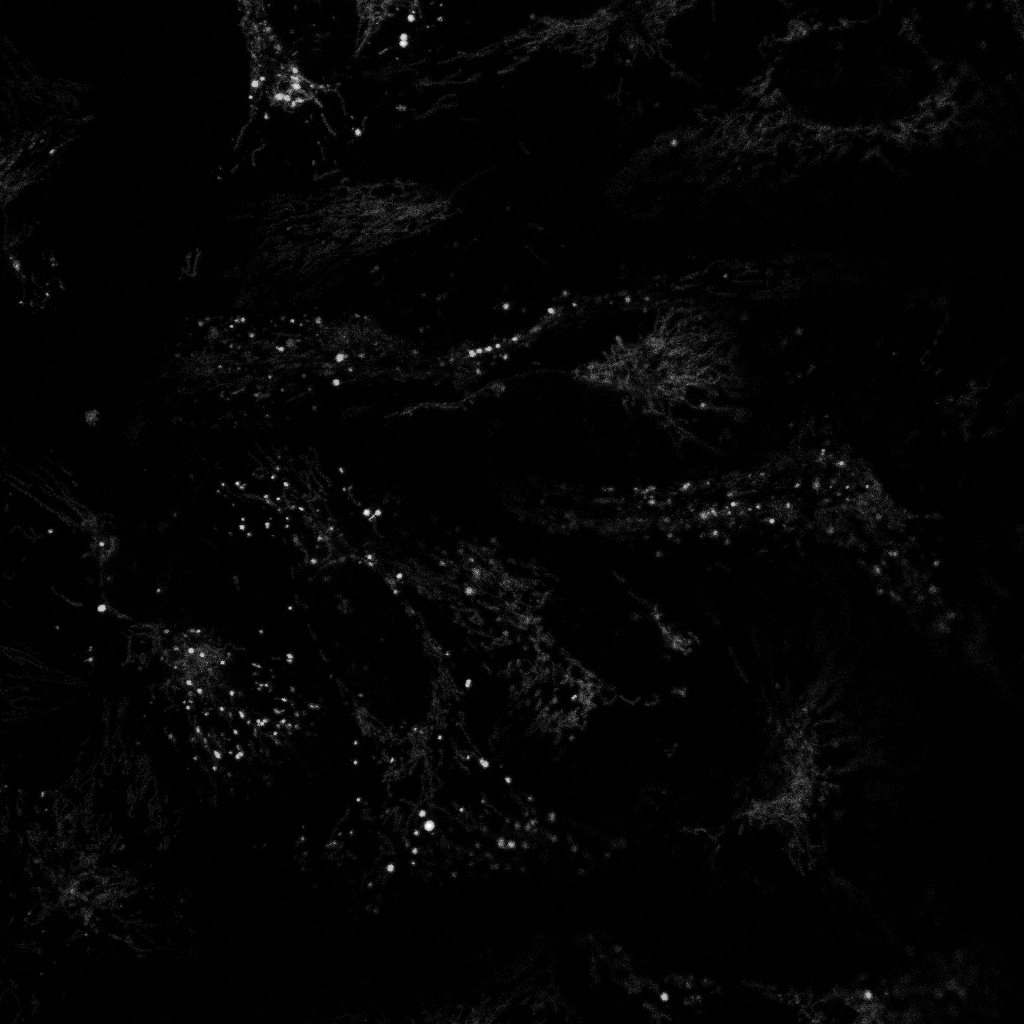

Supplement: Supplementary file 3 — Source data Fig. 1 [file 44319_2024_181_MOESM3_ESM.zip › Figure 1/Figure 1H/U2OS + 24hrs DFP.tif]

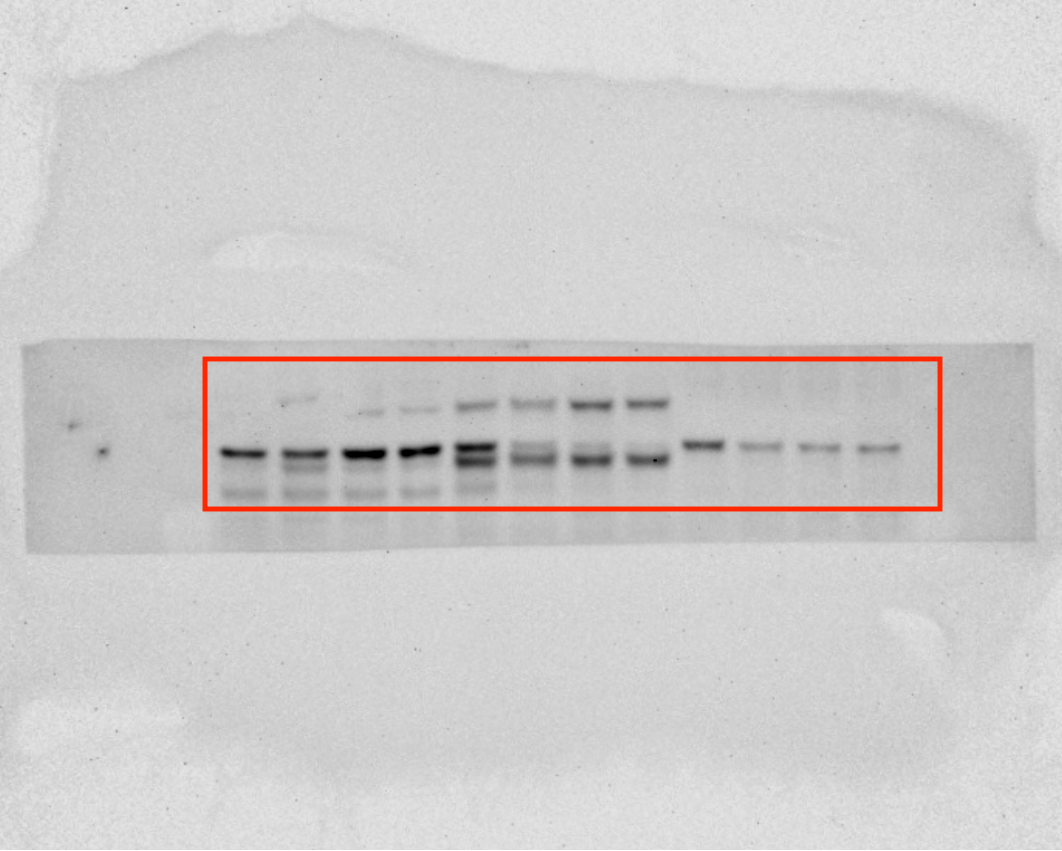

Supplement: Supplementary file 4 — Source data Fig. 2 [file 44319_2024_181_MOESM4_ESM.zip › Figure 2/Figure 2B/PPTC7.tif]

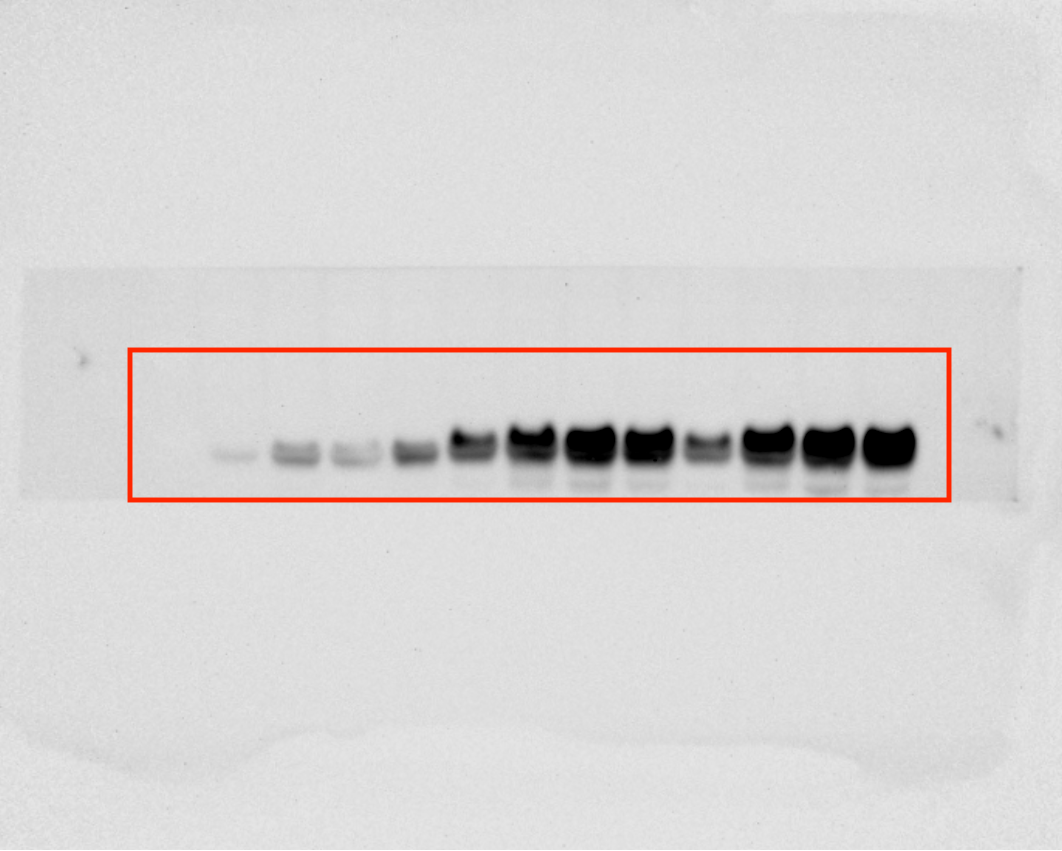

Supplement: Supplementary file 4 — Source data Fig. 2 [file 44319_2024_181_MOESM4_ESM.zip › Figure 2/Figure 2B/NIX.tif]

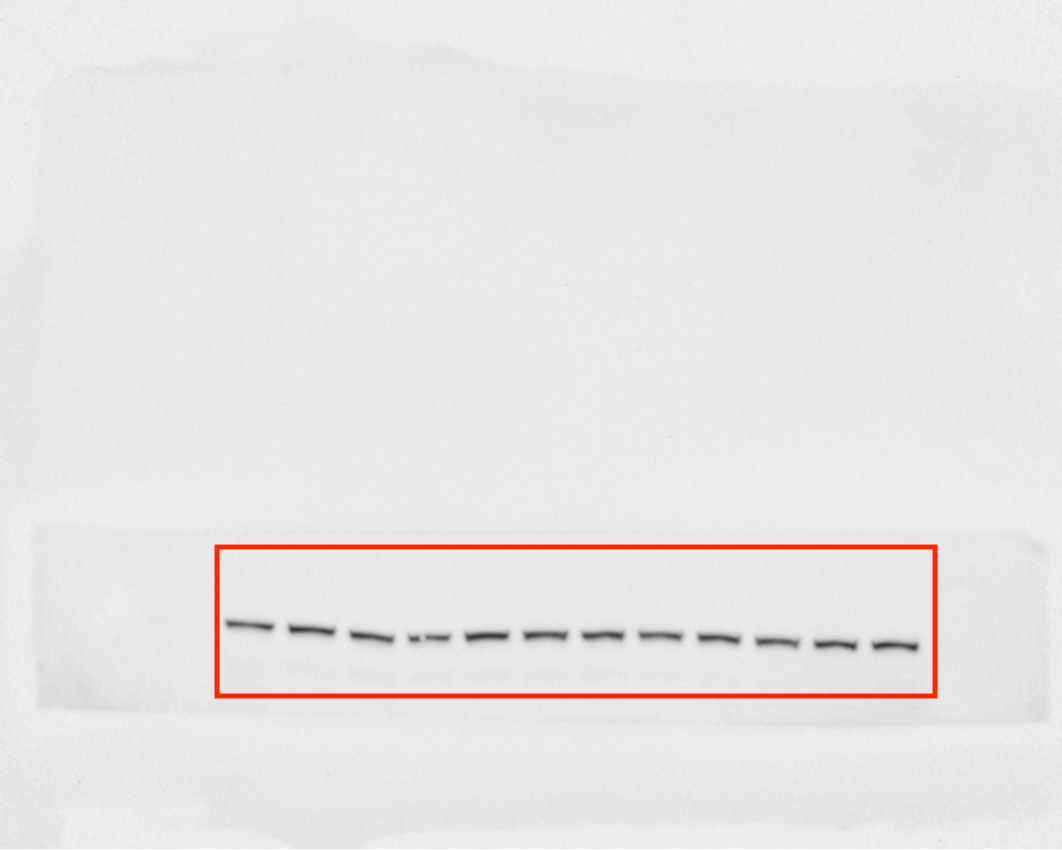

Supplement: Supplementary file 4 — Source data Fig. 2 [file 44319_2024_181_MOESM4_ESM.zip › Figure 2/Figure 2B/VCL.tif]

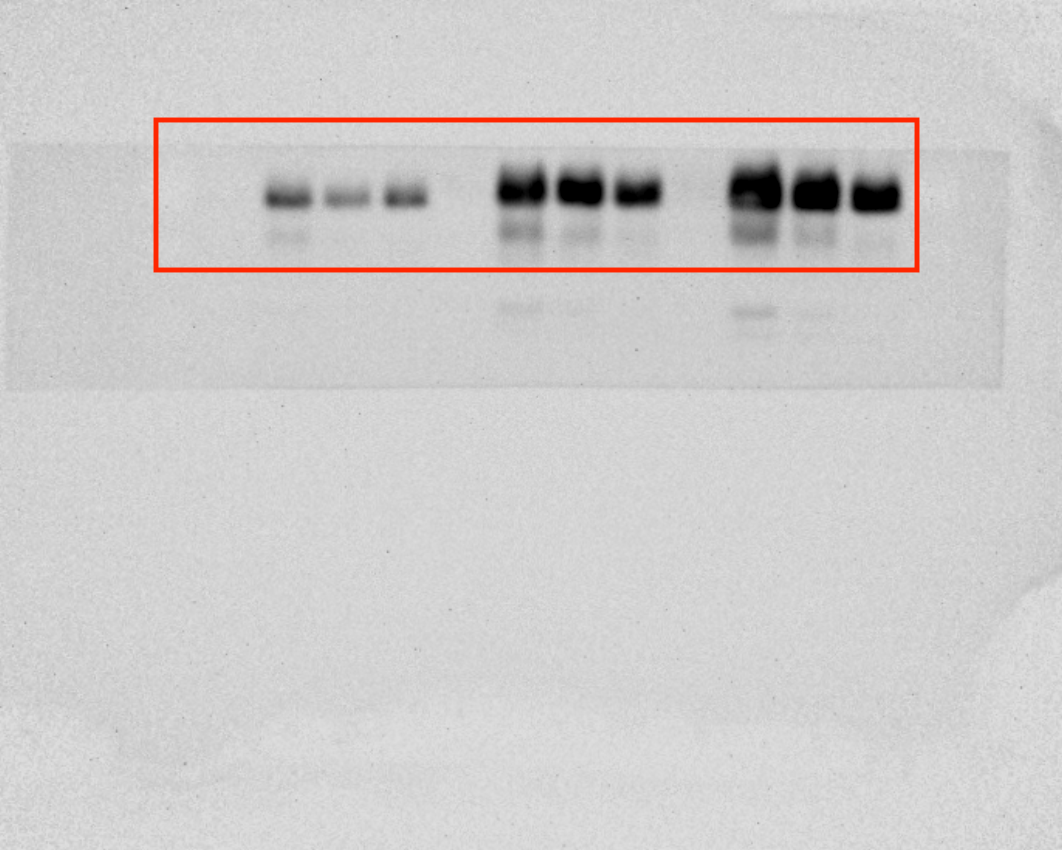

Supplement: Supplementary file 4 — Source data Fig. 2 [file 44319_2024_181_MOESM4_ESM.zip › Figure 2/Figure 2B/BNIP3.tif]

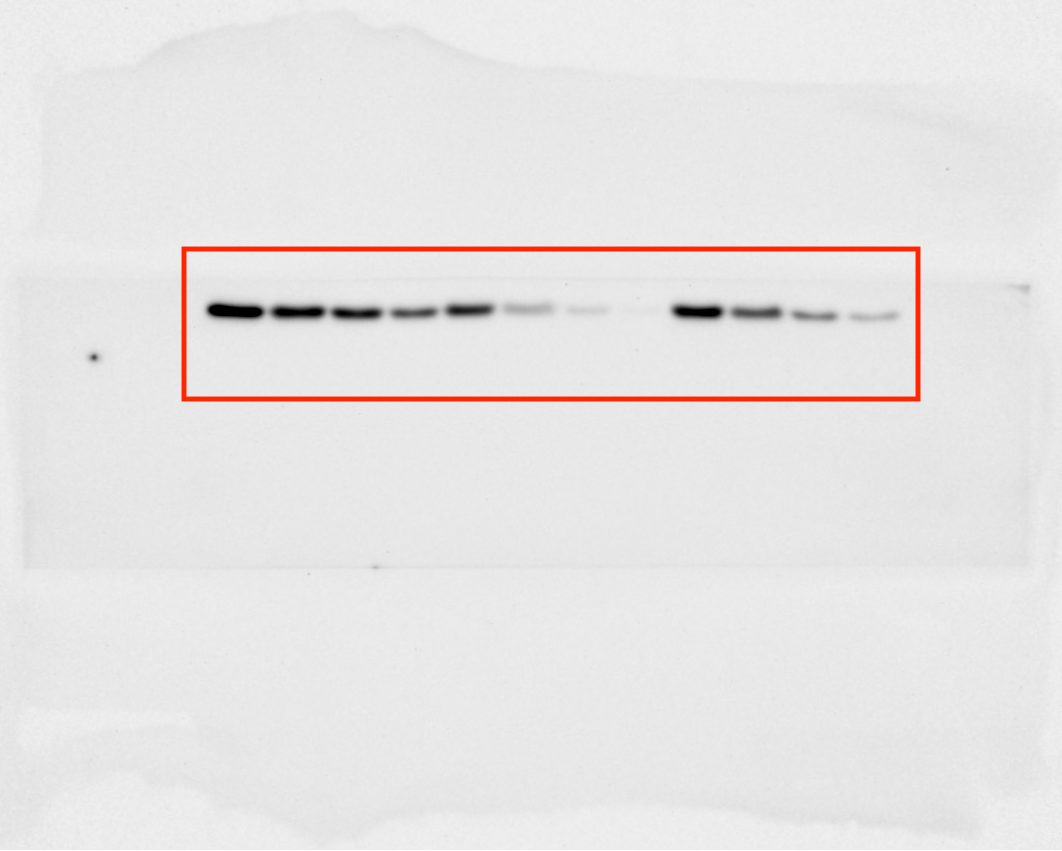

Supplement: Supplementary file 4 — Source data Fig. 2 [file 44319_2024_181_MOESM4_ESM.zip › Figure 2/Figure 2B/MTCO2.tif]

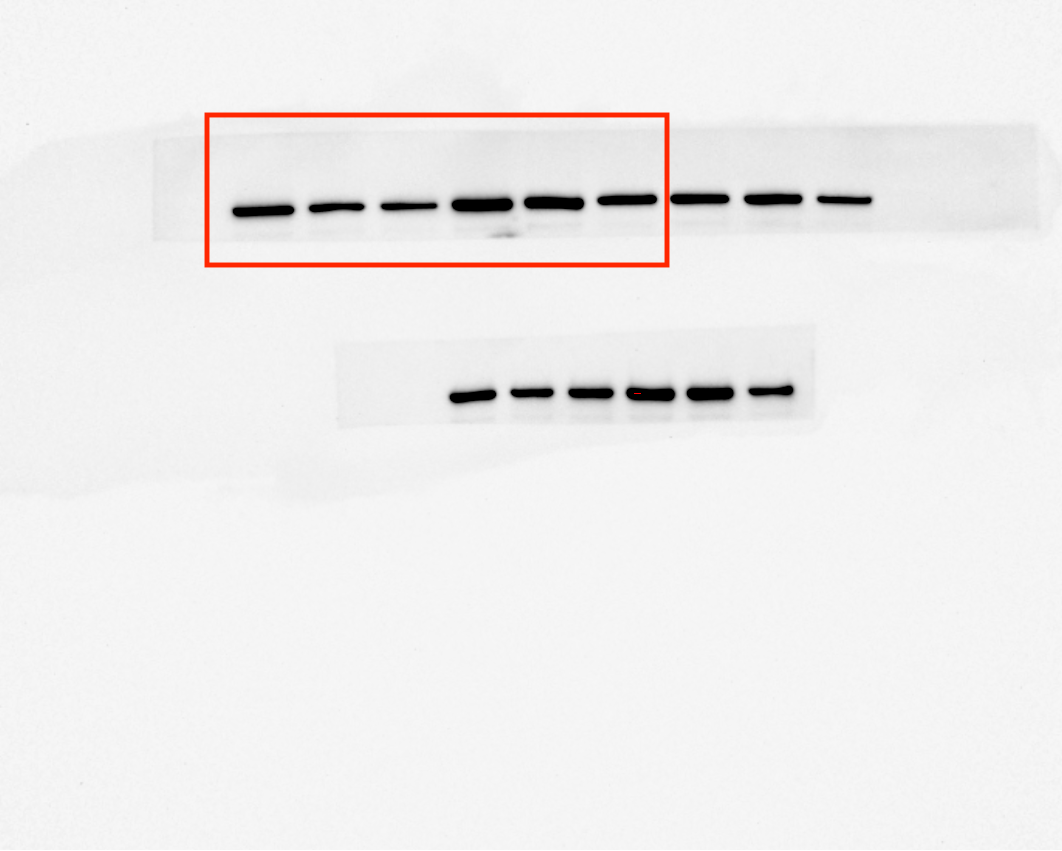

Supplement: Supplementary file 4 — Source data Fig. 2 [file 44319_2024_181_MOESM4_ESM.zip › Figure 2/Figure 2D/wcl vcl .tif]

WHOLE CELL LYSATE

VCL M 1:1000

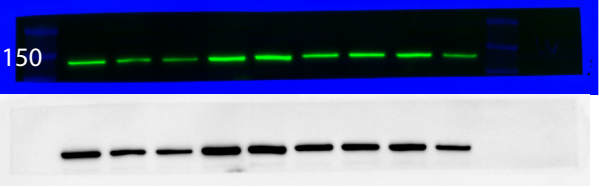

NIX M 1:1000

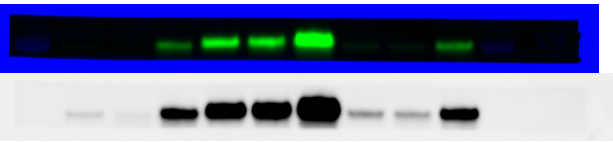

BNIP3 M 1:500

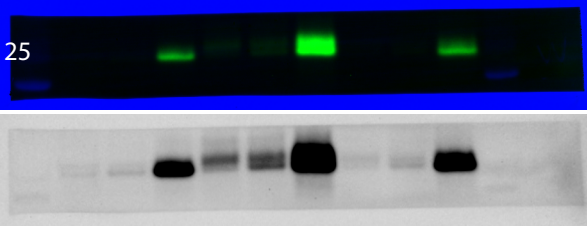

FLAG Rb 1:1000

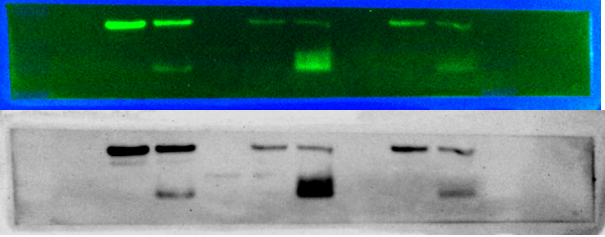

IP SAMPLES

FLAG Rb 1:1000

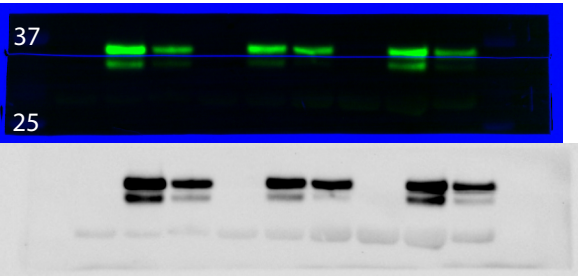

NIX Rb 1:1000

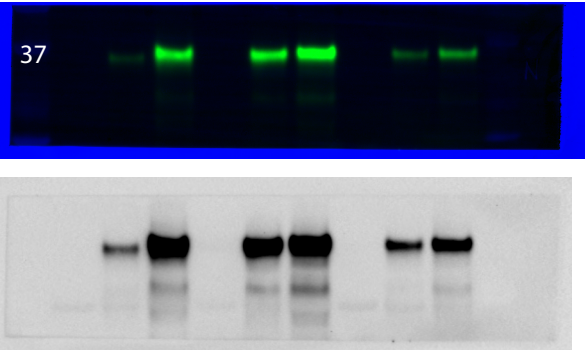

BNIP3 Rb 1:1000

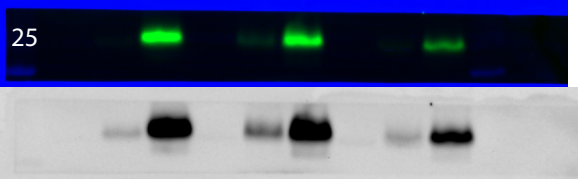

Supplement: Supplementary file 4 — Source data Fig. 2 [file 44319_2024_181_MOESM4_ESM.zip › Figure 2/Figure 2D/Annotation Figure 2D.pdf]

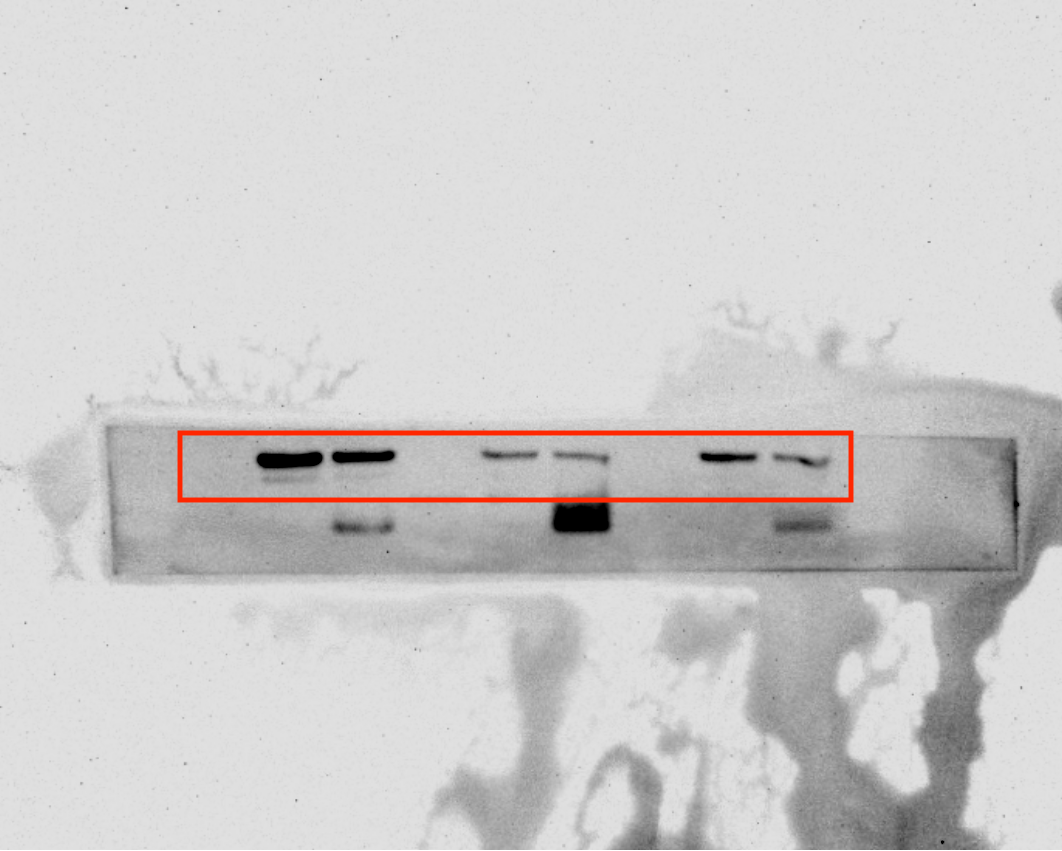

Supplement: Supplementary file 4 — Source data Fig. 2 [file 44319_2024_181_MOESM4_ESM.zip › Figure 2/Figure 2D/flag WCL.tif]

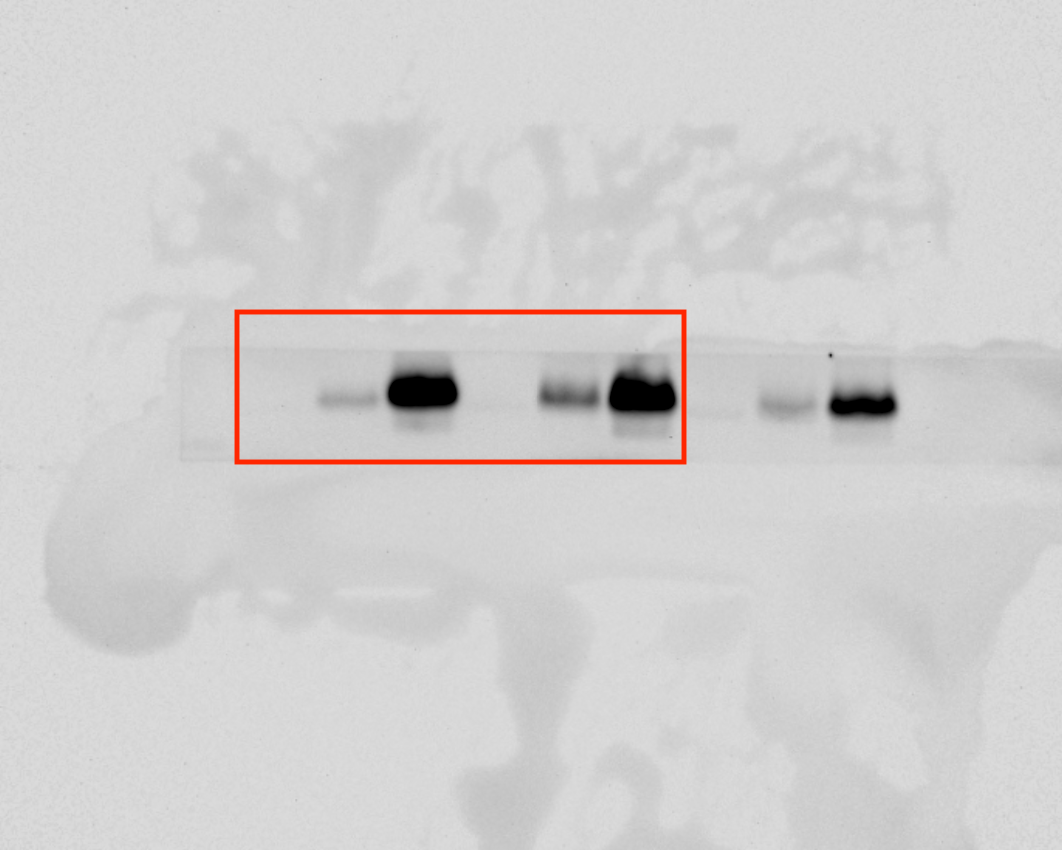

Supplement: Supplementary file 4 — Source data Fig. 2 [file 44319_2024_181_MOESM4_ESM.zip › Figure 2/Figure 2D/bnip3 IP.tif]

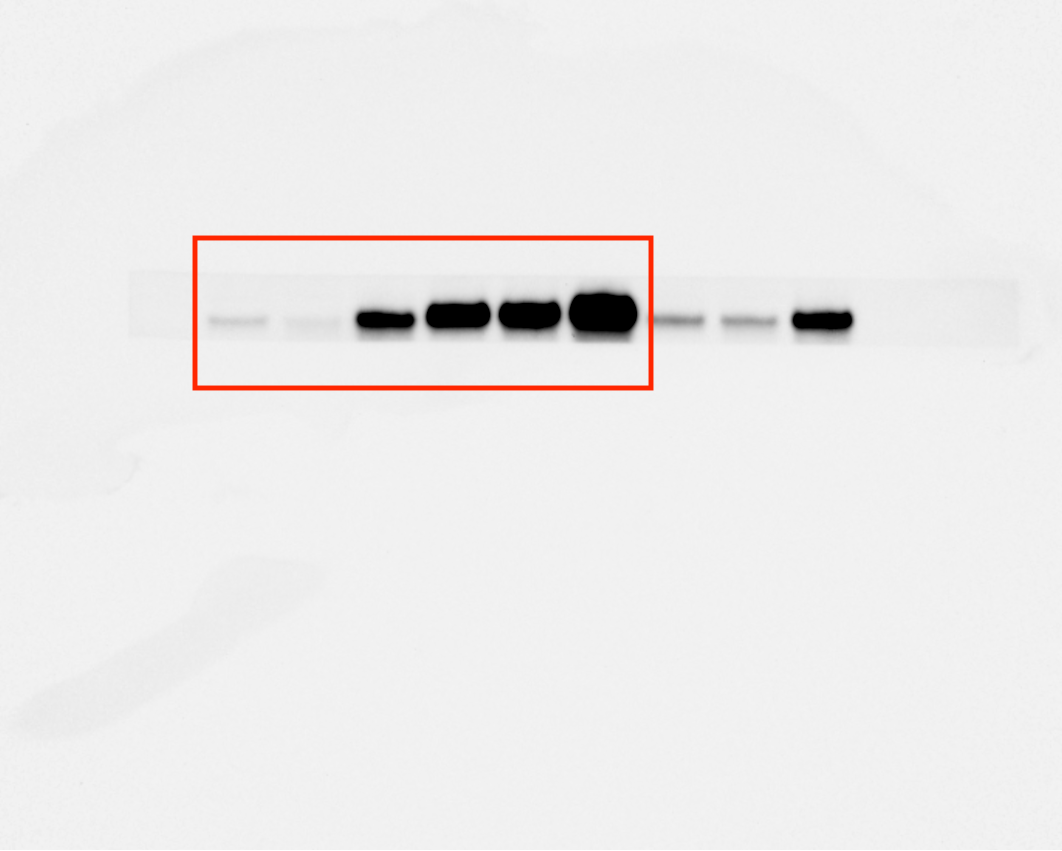

Supplement: Supplementary file 4 — Source data Fig. 2 [file 44319_2024_181_MOESM4_ESM.zip › Figure 2/Figure 2D/wcl nix.tif]

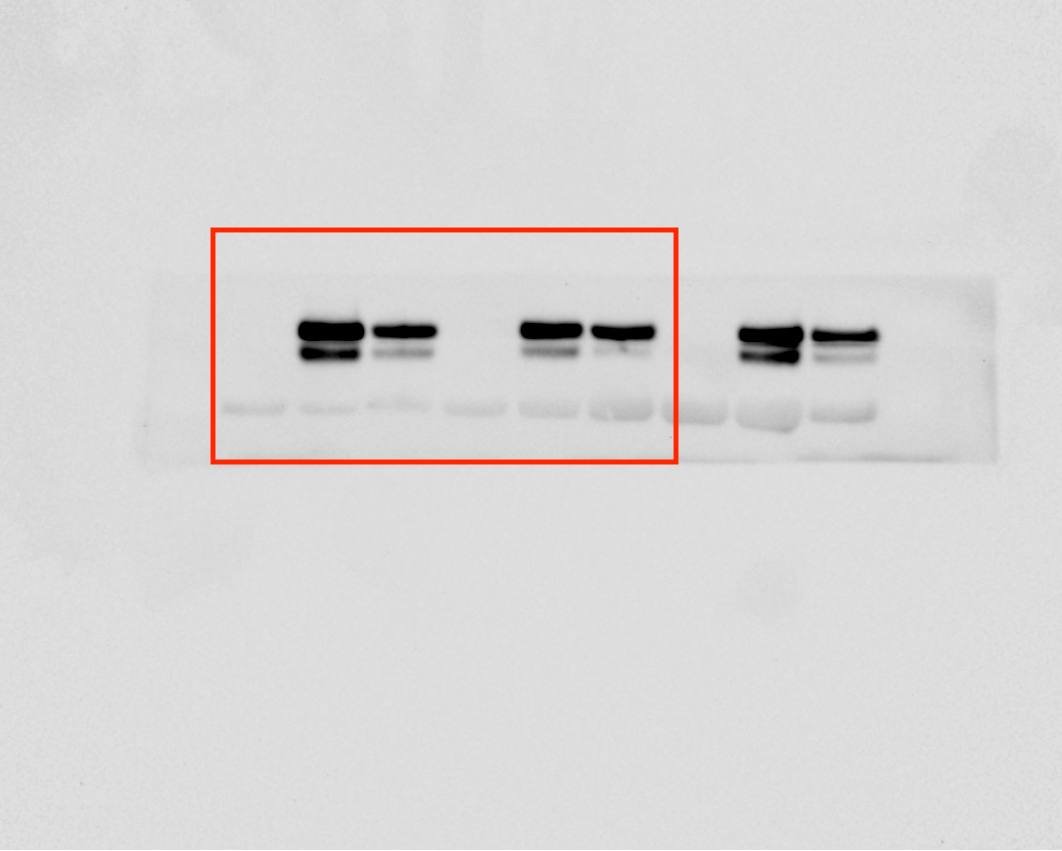

Supplement: Supplementary file 4 — Source data Fig. 2 [file 44319_2024_181_MOESM4_ESM.zip › Figure 2/Figure 2D/FLAG IP.tif]

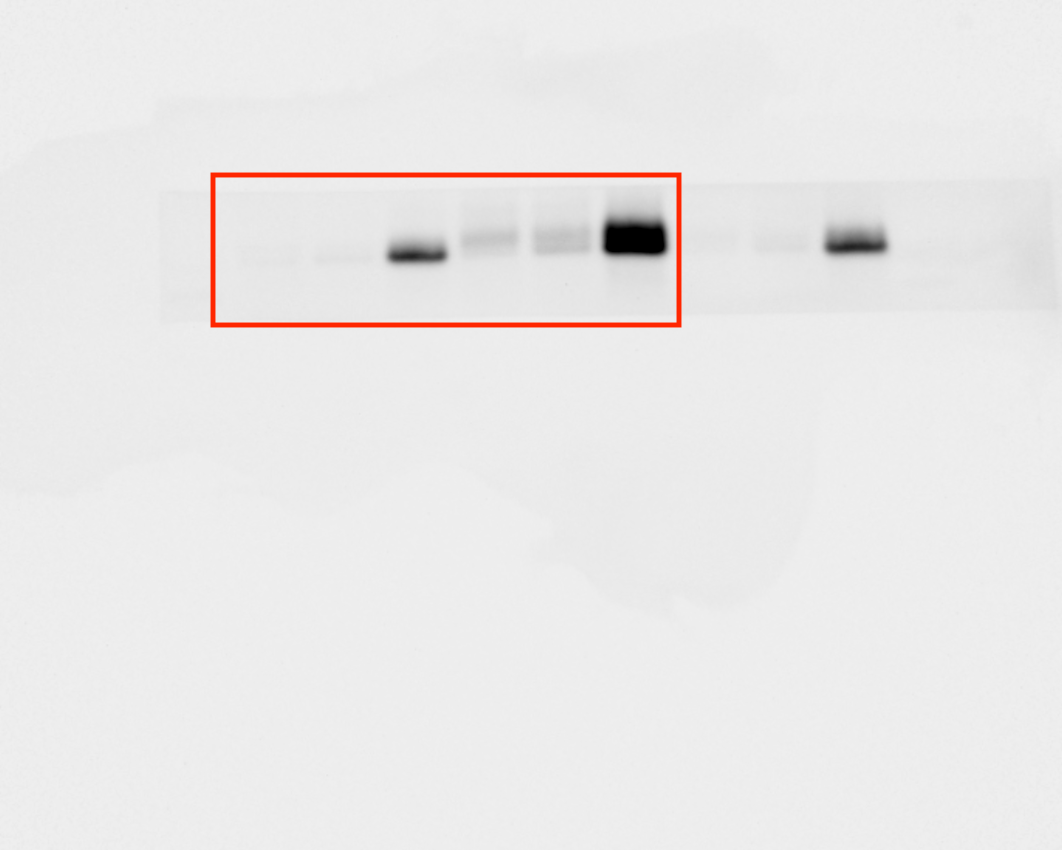

Supplement: Supplementary file 4 — Source data Fig. 2 [file 44319_2024_181_MOESM4_ESM.zip › Figure 2/Figure 2D/wcl bnip3 .tif]

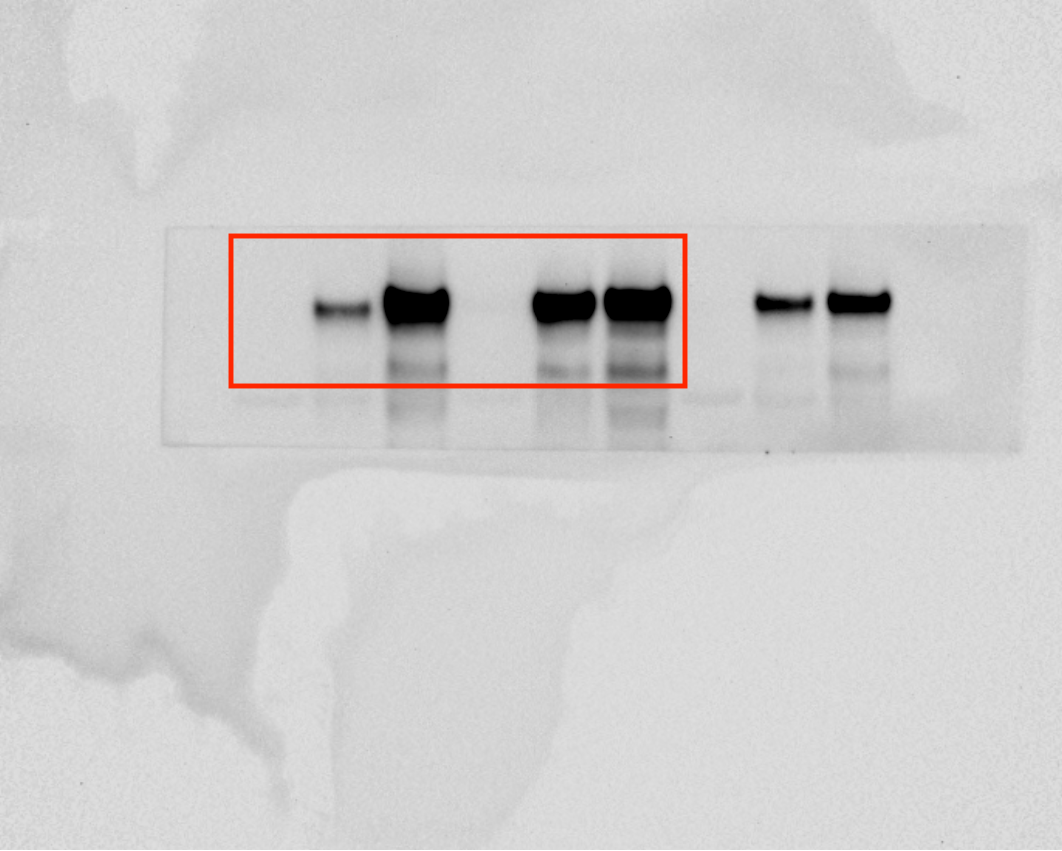

Supplement: Supplementary file 4 — Source data Fig. 2 [file 44319_2024_181_MOESM4_ESM.zip › Figure 2/Figure 2D/NIX IP.tif]

[illegible]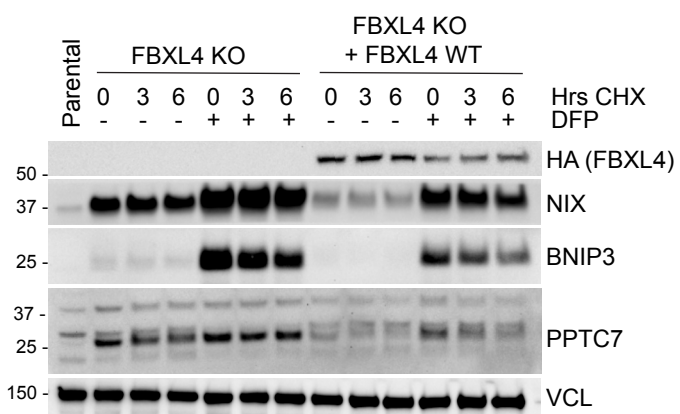

Supplement: Supplementary file 4 — Source data Fig. 2 [file 44319_2024_181_MOESM4_ESM.zip › Figure 2/Figure 2C/Annotation Figure 2C.pdf]

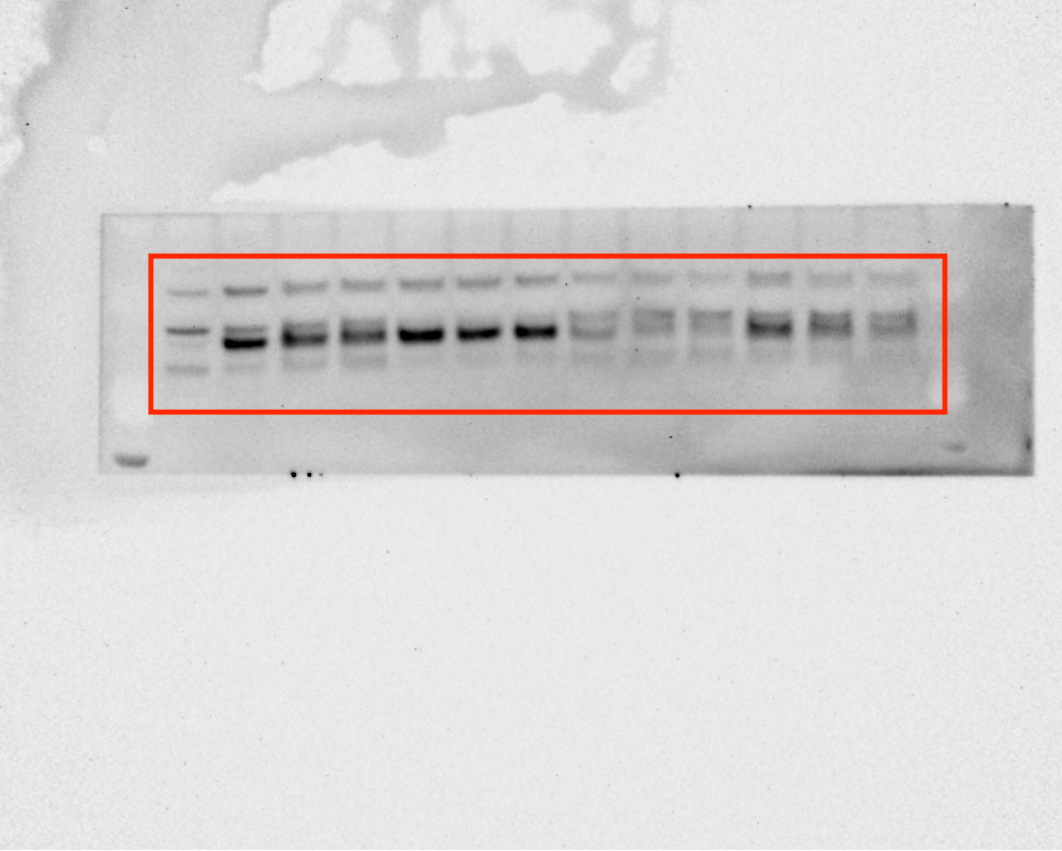

Supplement: Supplementary file 4 — Source data Fig. 2 [file 44319_2024_181_MOESM4_ESM.zip › Figure 2/Figure 2C/pptc7.tif]

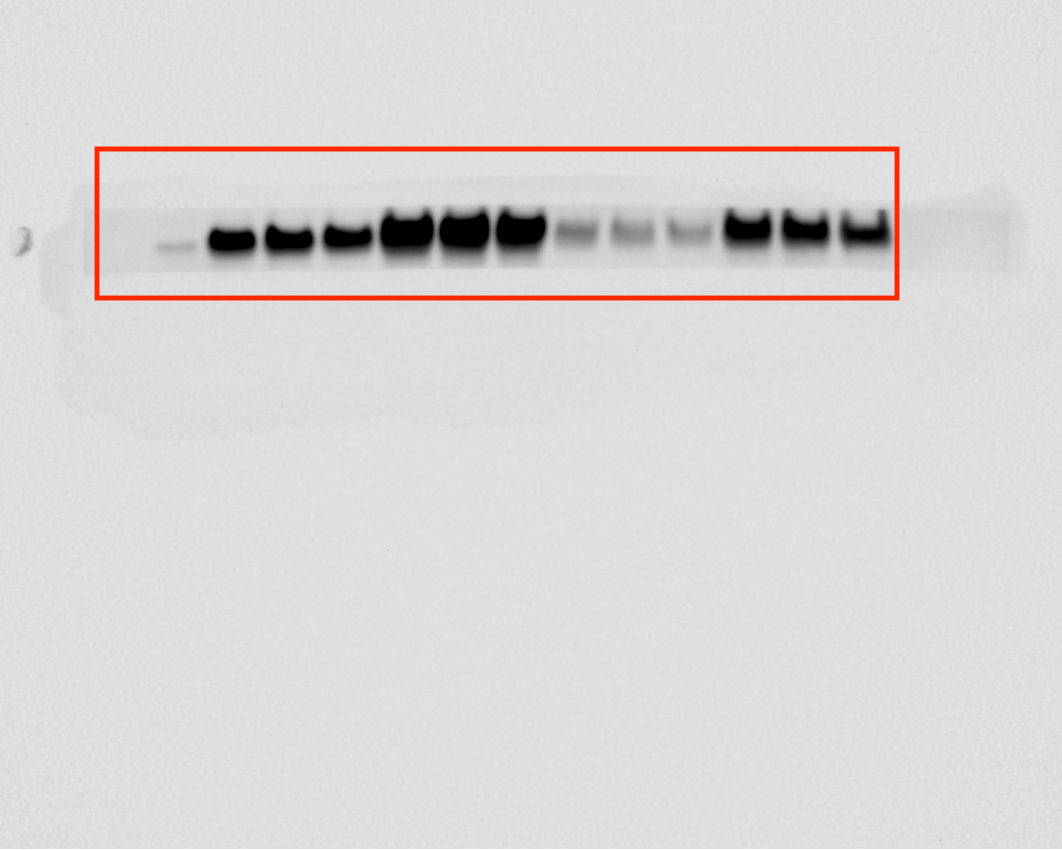

Supplement: Supplementary file 4 — Source data Fig. 2 [file 44319_2024_181_MOESM4_ESM.zip › Figure 2/Figure 2C/nix.tif]

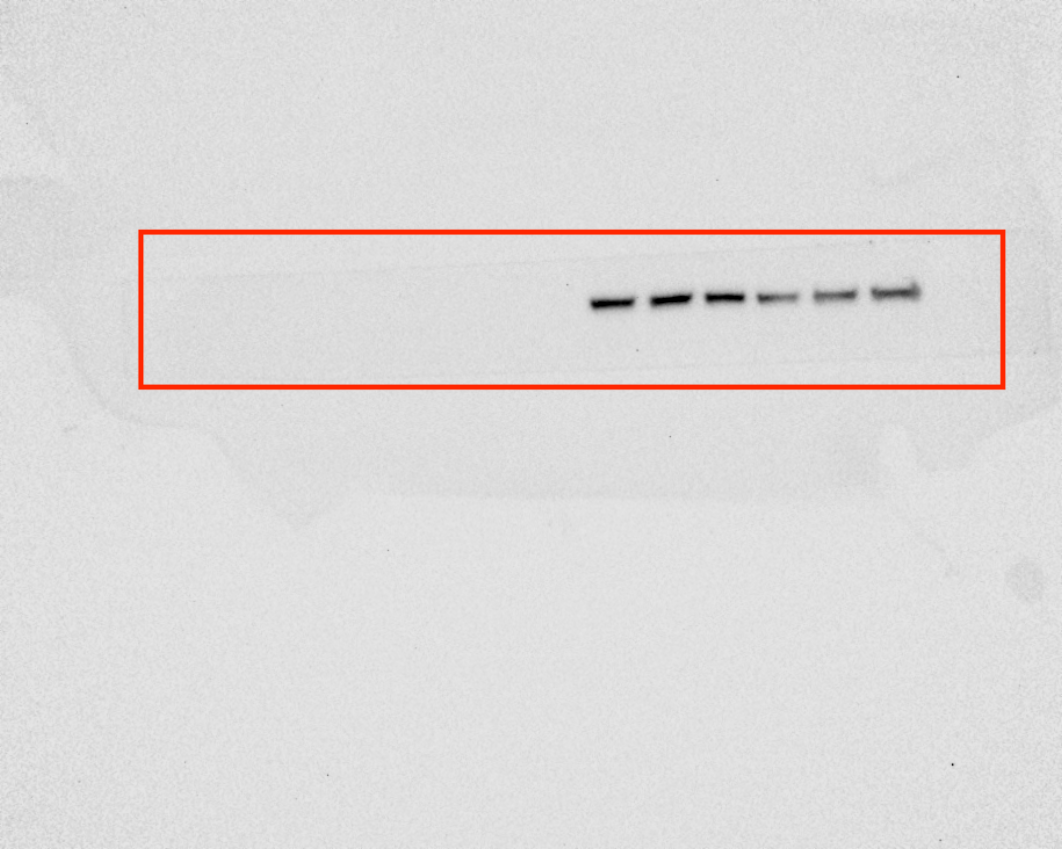

Supplement: Supplementary file 4 — Source data Fig. 2 [file 44319_2024_181_MOESM4_ESM.zip › Figure 2/Figure 2C/ha.tif]

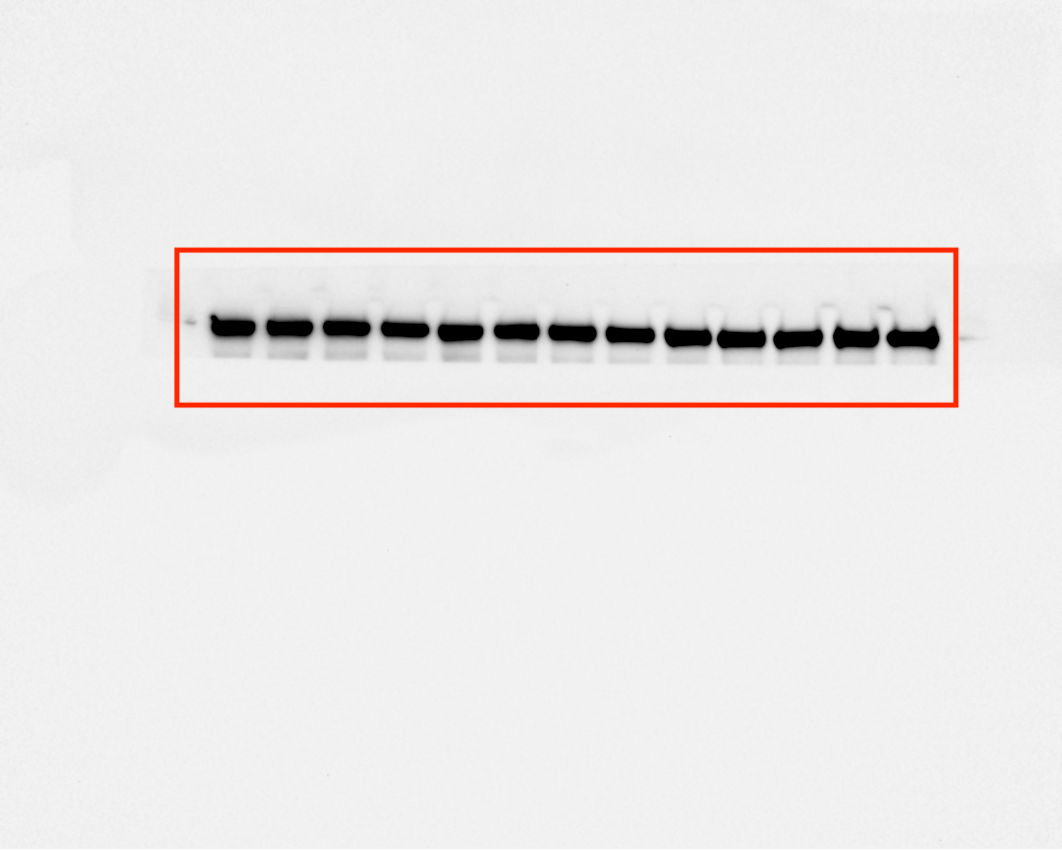

Supplement: Supplementary file 4 — Source data Fig. 2 [file 44319_2024_181_MOESM4_ESM.zip › Figure 2/Figure 2C/vcl.tif]

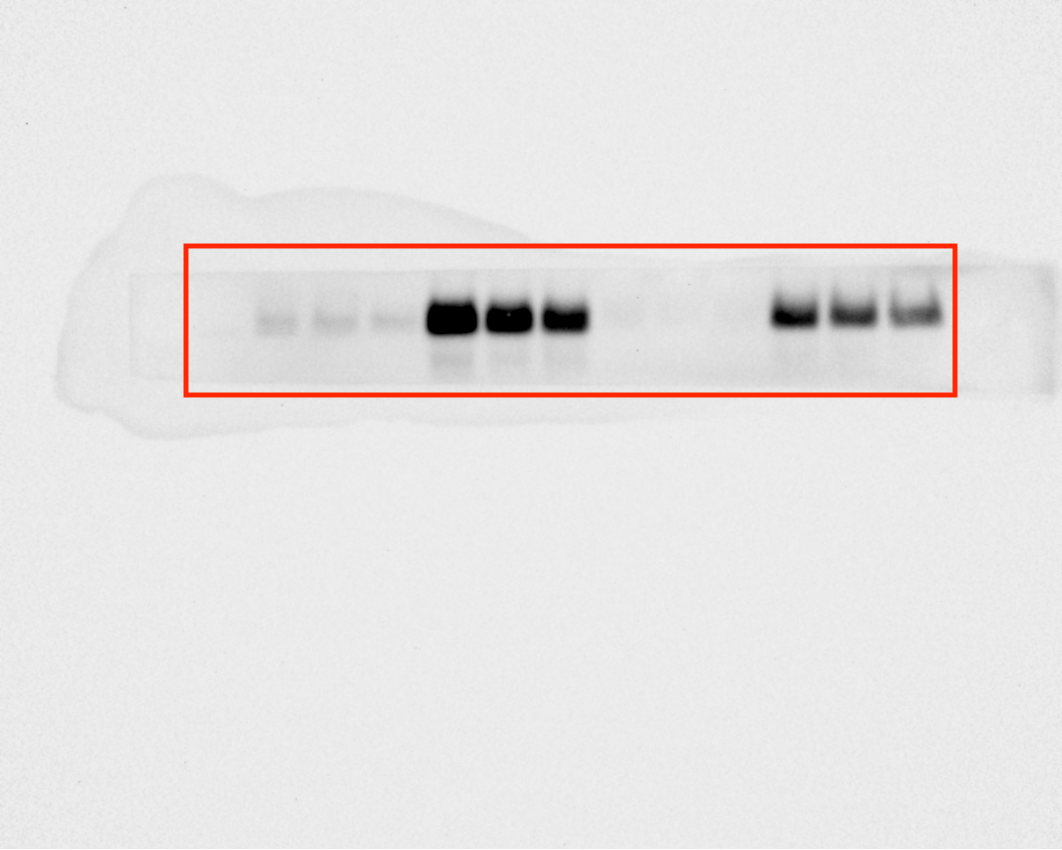

Supplement: Supplementary file 4 — Source data Fig. 2 [file 44319_2024_181_MOESM4_ESM.zip › Figure 2/Figure 2C/bnip3.tif]

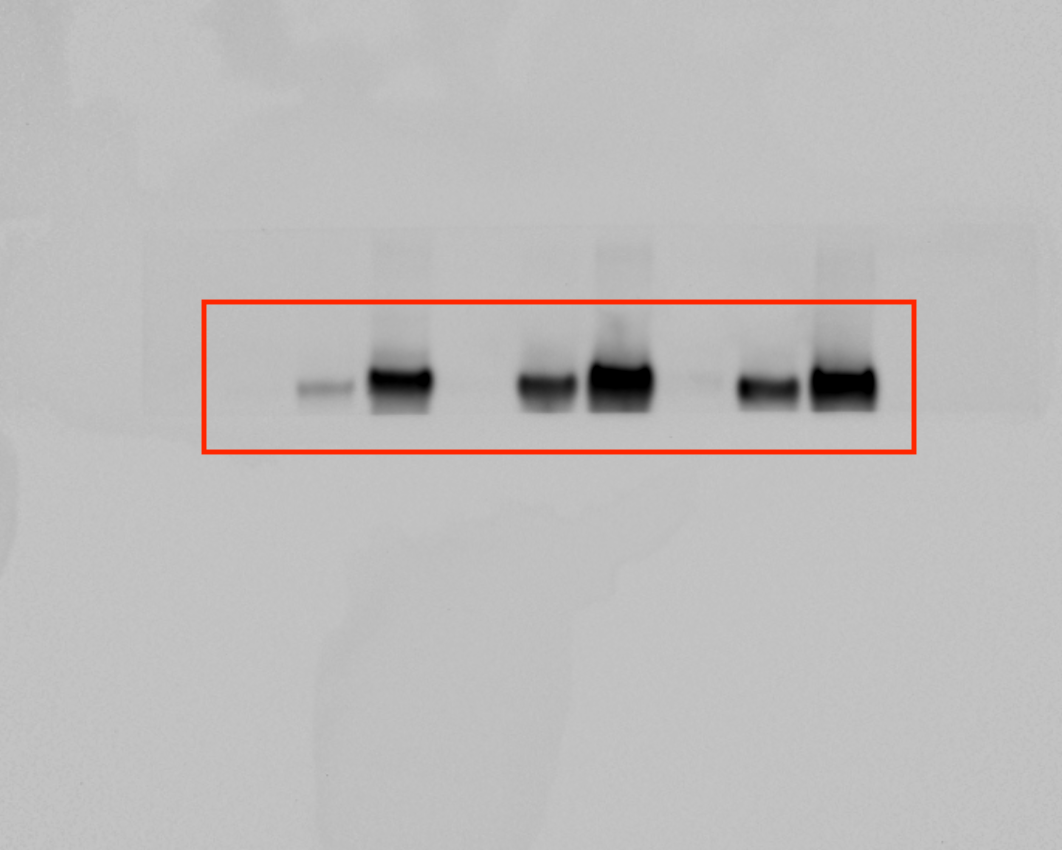

Supplement: Supplementary file 4 — Source data Fig. 2 [file 44319_2024_181_MOESM4_ESM.zip › Figure 2/Figure 2E/ip nix.tif]

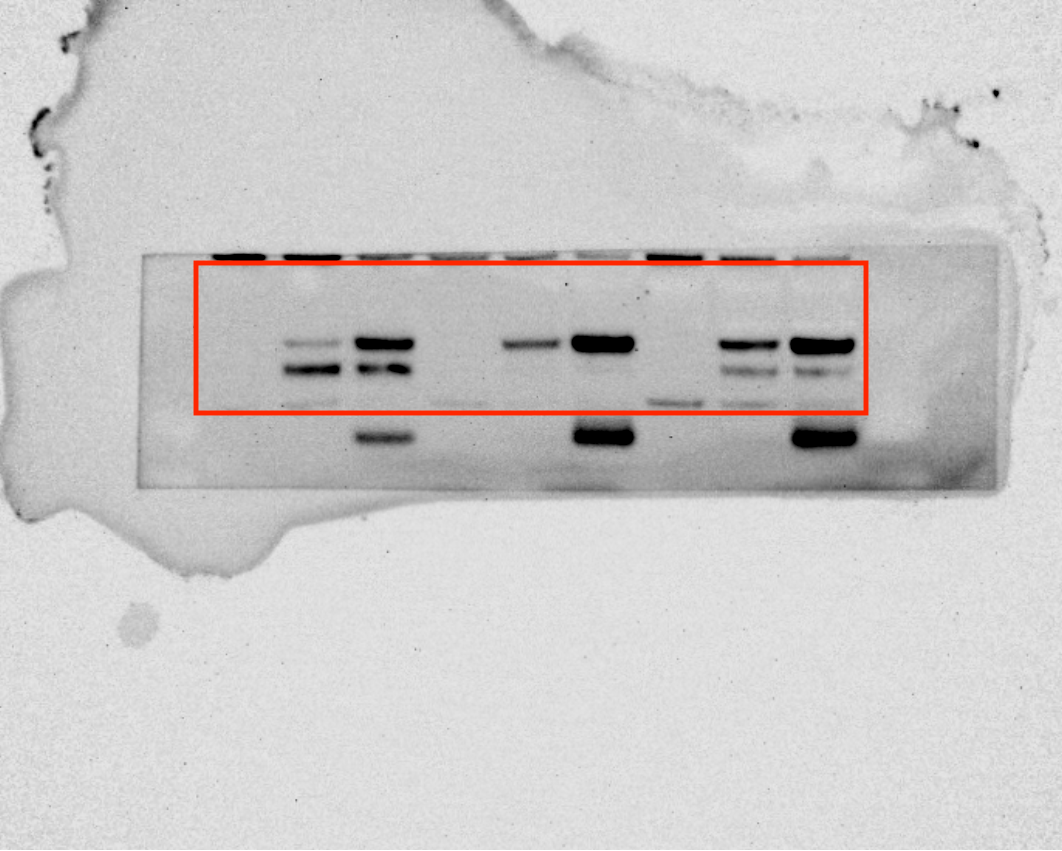

Supplement: Supplementary file 4 — Source data Fig. 2 [file 44319_2024_181_MOESM4_ESM.zip › Figure 2/Figure 2E/wcl flag.tif]

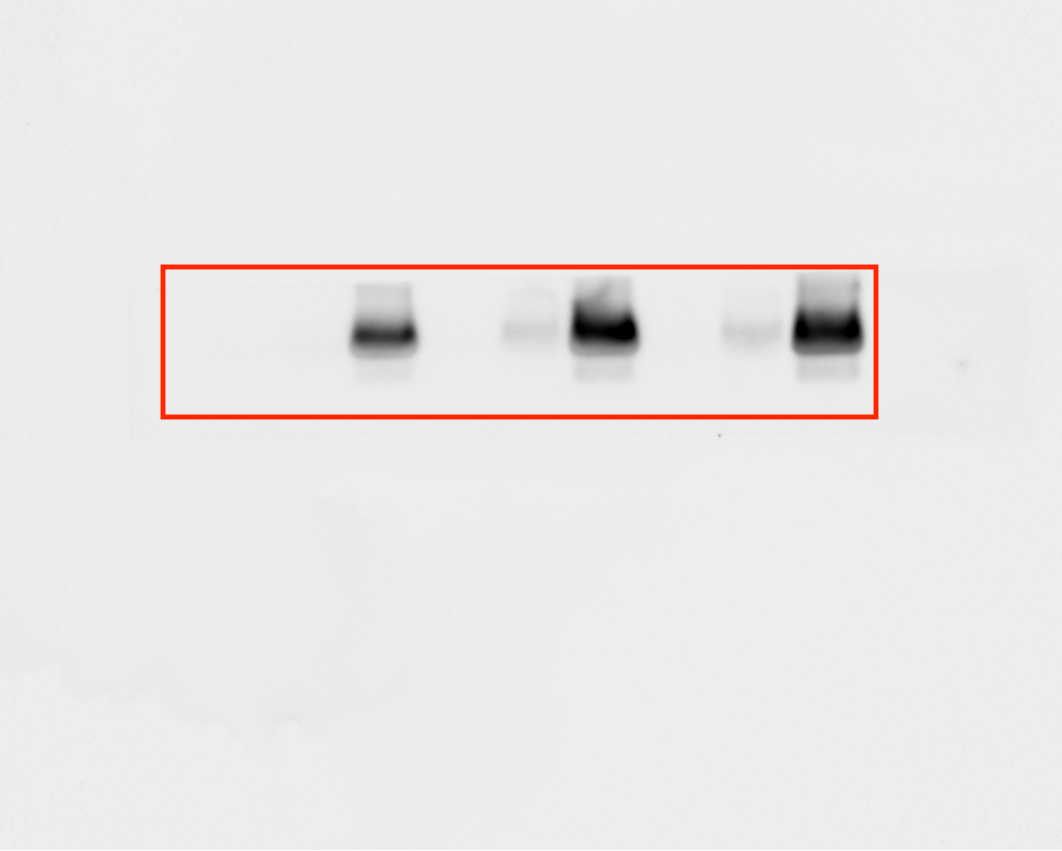

Supplement: Supplementary file 4 — Source data Fig. 2 [file 44319_2024_181_MOESM4_ESM.zip › Figure 2/Figure 2E/ip bnip3.tif]

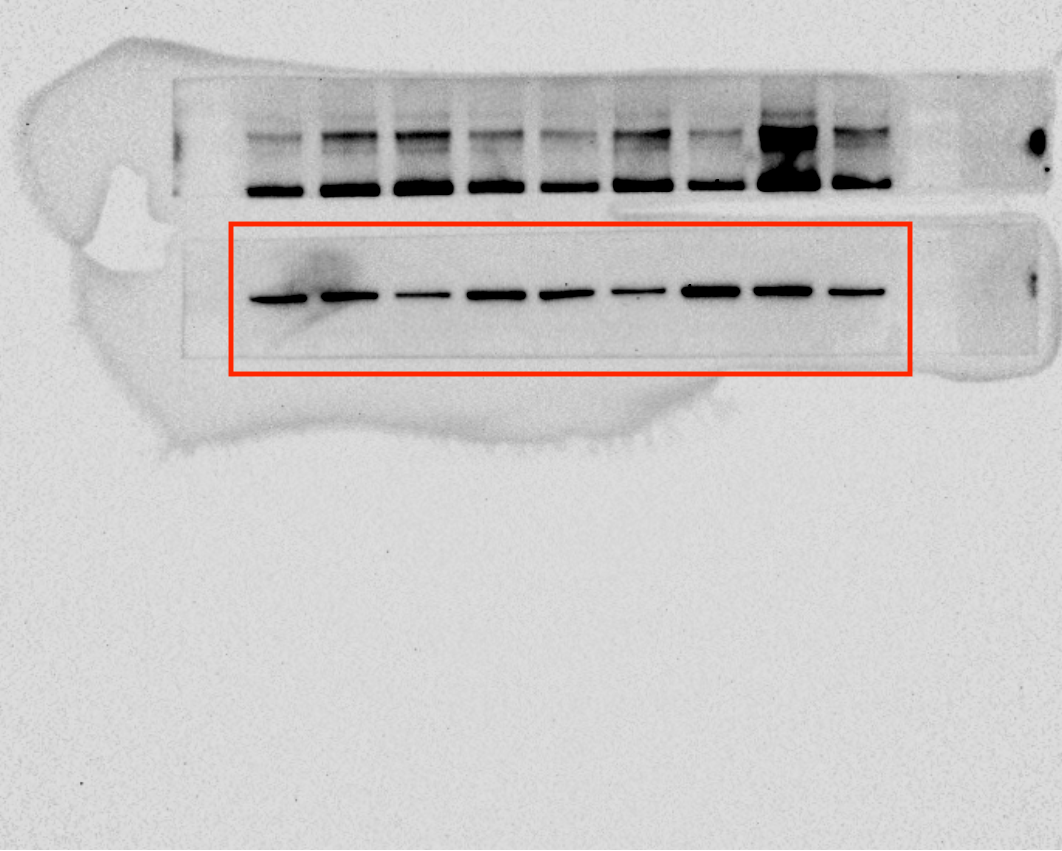

Supplement: Supplementary file 4 — Source data Fig. 2 [file 44319_2024_181_MOESM4_ESM.zip › Figure 2/Figure 2E/wcl vcl.tif]

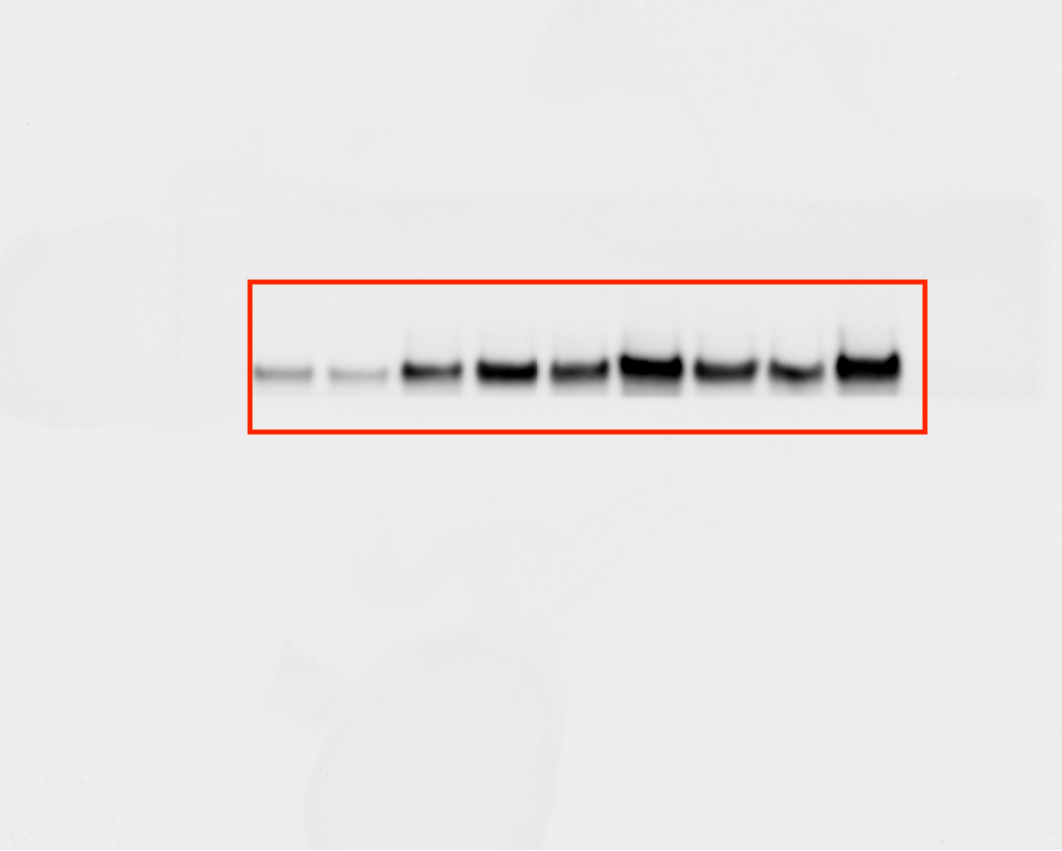

Supplement: Supplementary file 4 — Source data Fig. 2 [file 44319_2024_181_MOESM4_ESM.zip › Figure 2/Figure 2E/wcl nix.tif]

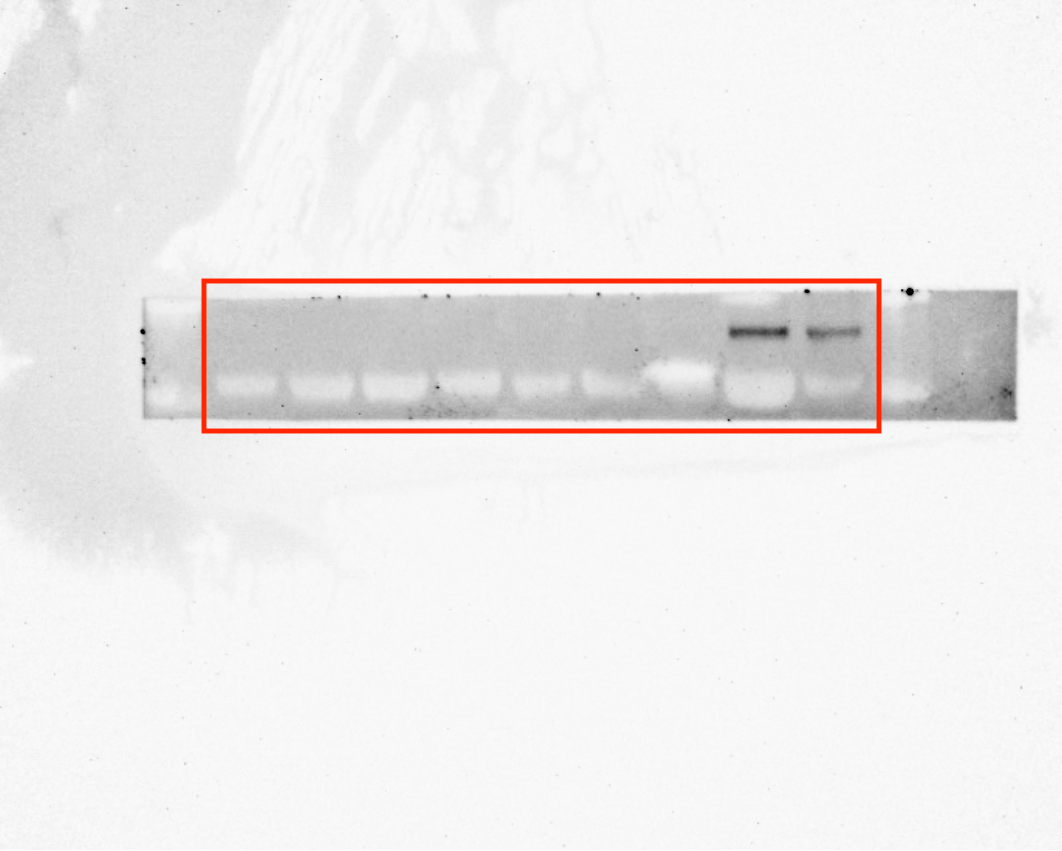

Supplement: Supplementary file 4 — Source data Fig. 2 [file 44319_2024_181_MOESM4_ESM.zip › Figure 2/Figure 2E/ip ha.tif]

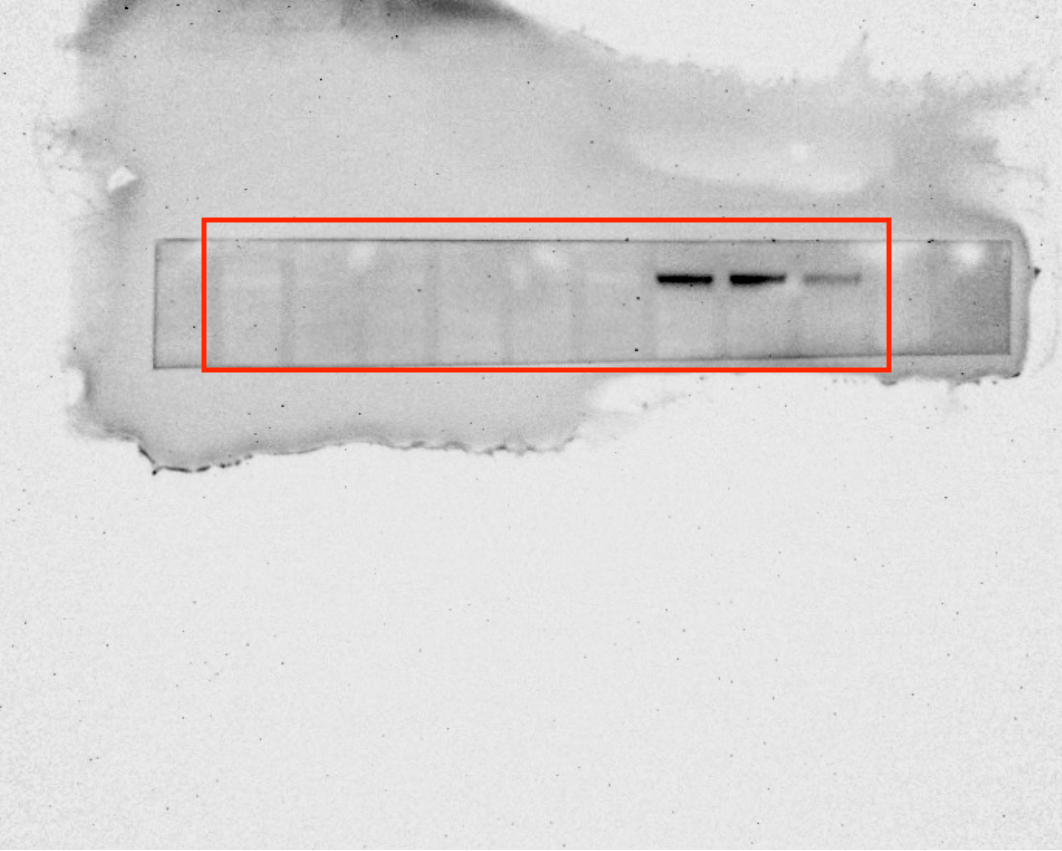

Supplement: Supplementary file 4 — Source data Fig. 2 [file 44319_2024_181_MOESM4_ESM.zip › Figure 2/Figure 2E/wcl ha 2823.tif]

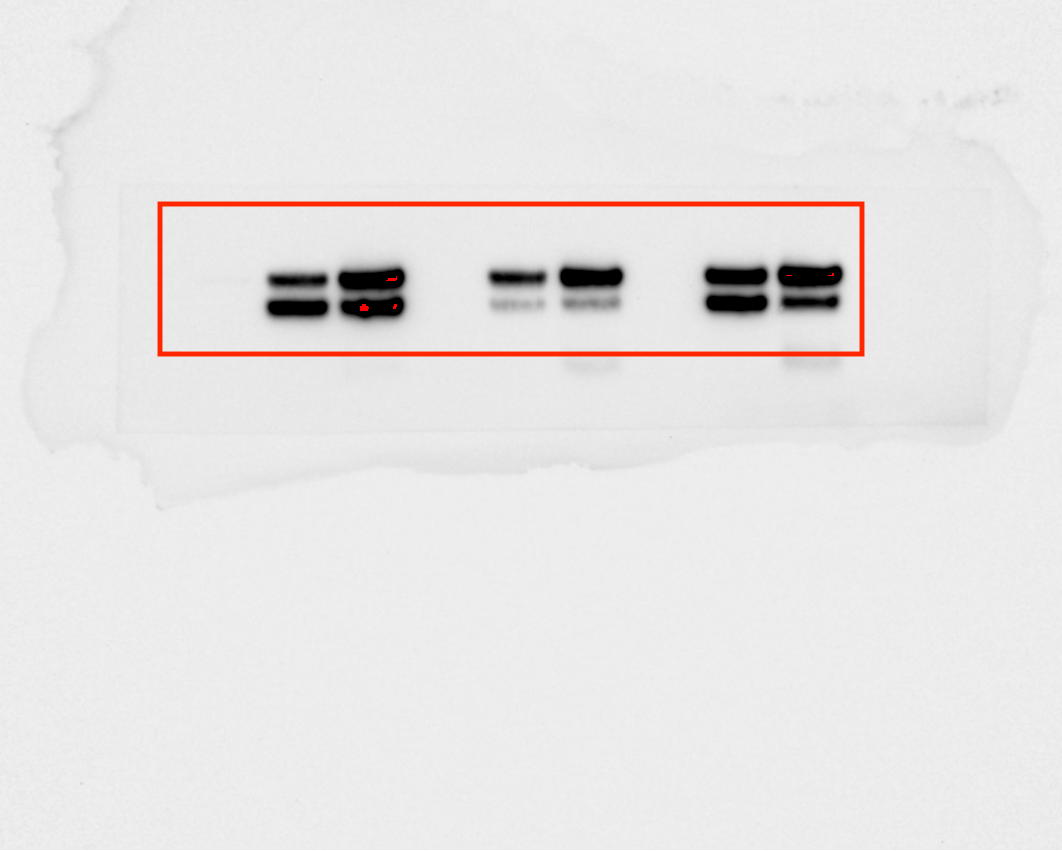

Supplement: Supplementary file 4 — Source data Fig. 2 [file 44319_2024_181_MOESM4_ESM.zip › Figure 2/Figure 2E/ip flag.tif]

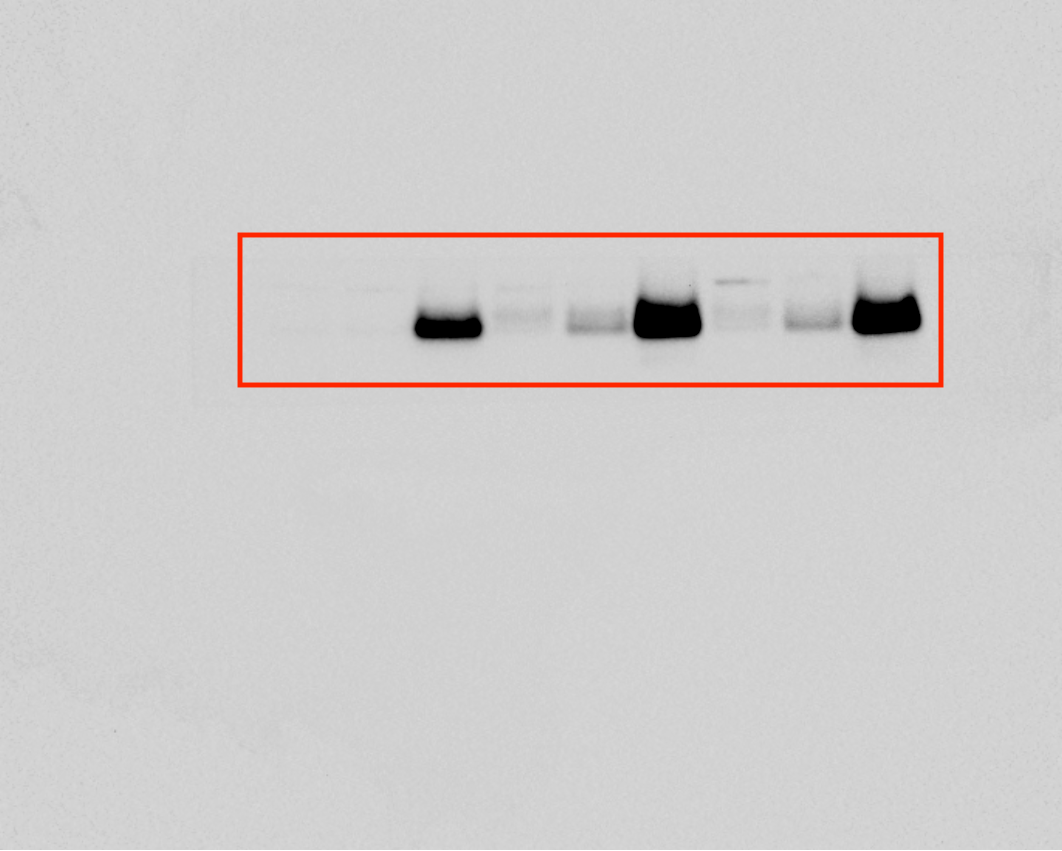

Supplement: Supplementary file 4 — Source data Fig. 2 [file 44319_2024_181_MOESM4_ESM.zip › Figure 2/Figure 2E/wcl bnip3.tif]

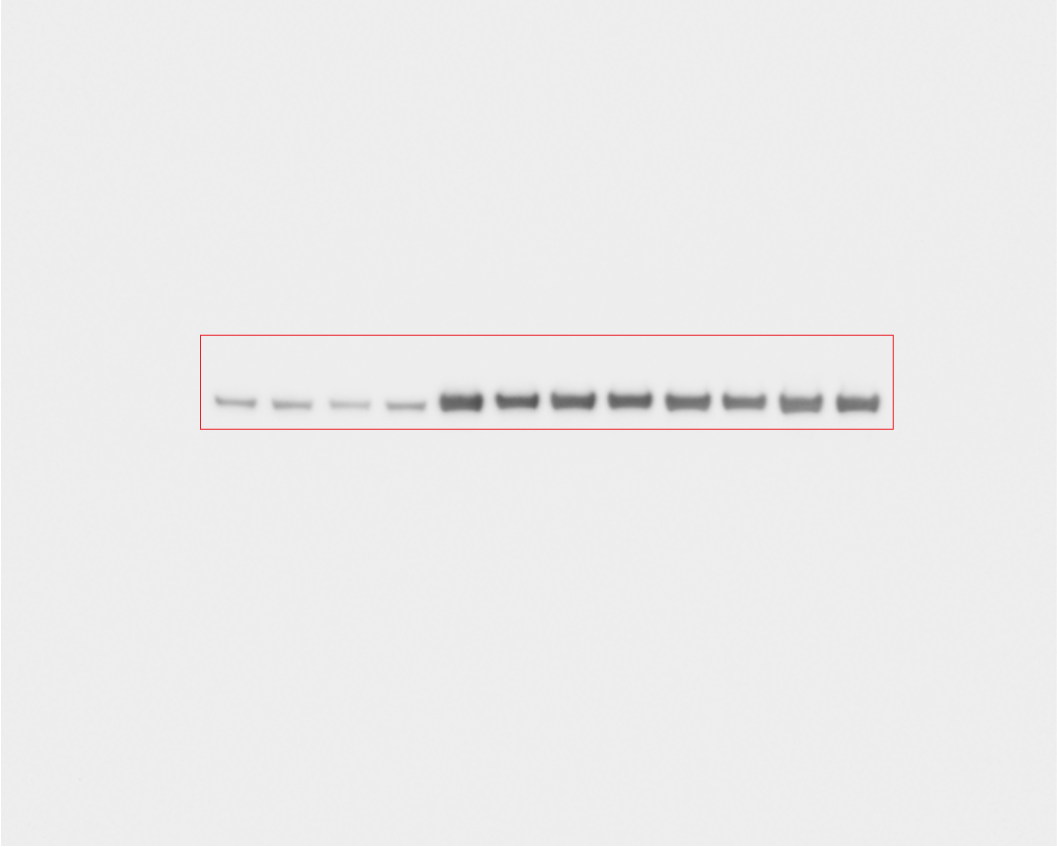

Supplement: Supplementary file 4 — Source data Fig. 2 [file 44319_2024_181_MOESM4_ESM.zip › Figure 2/Figure 2A/HSP60.png]

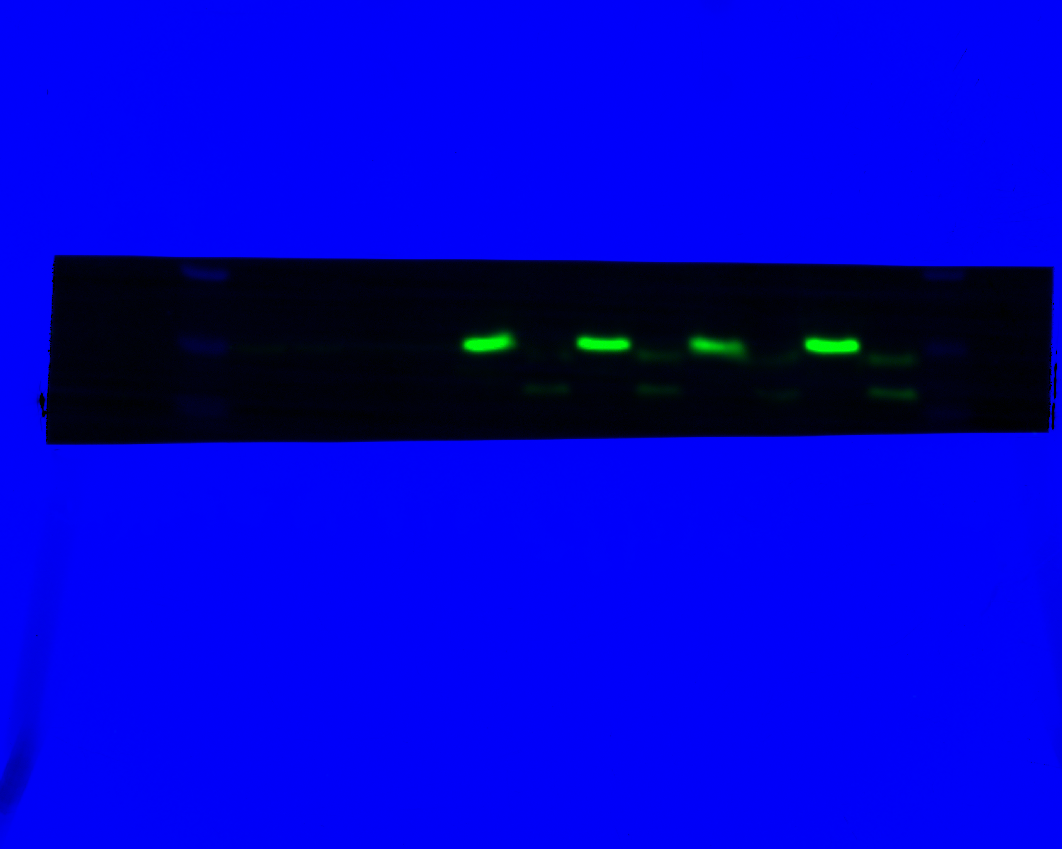

Supplement: Supplementary file 4 — Source data Fig. 2 [file 44319_2024_181_MOESM4_ESM.zip › Figure 2/Figure 2A/(TOM20) (Composite).tif]

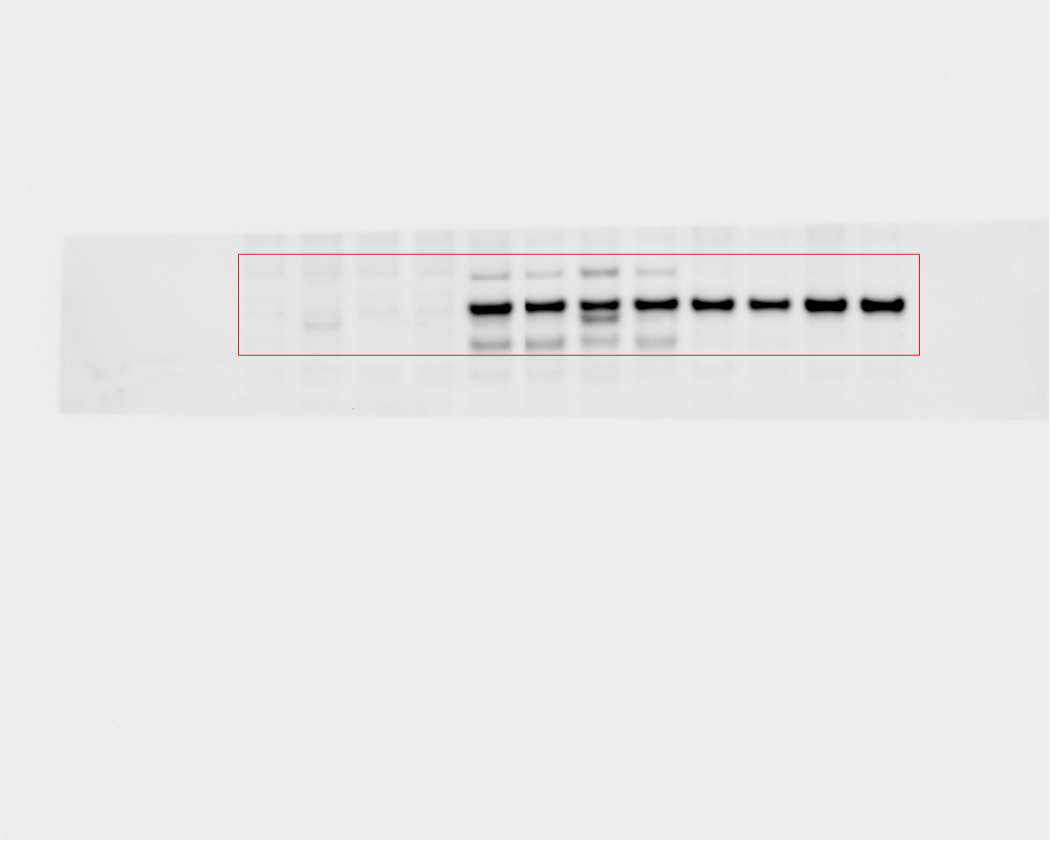

Supplement: Supplementary file 4 — Source data Fig. 2 [file 44319_2024_181_MOESM4_ESM.zip › Figure 2/Figure 2A/PPTC7.png]

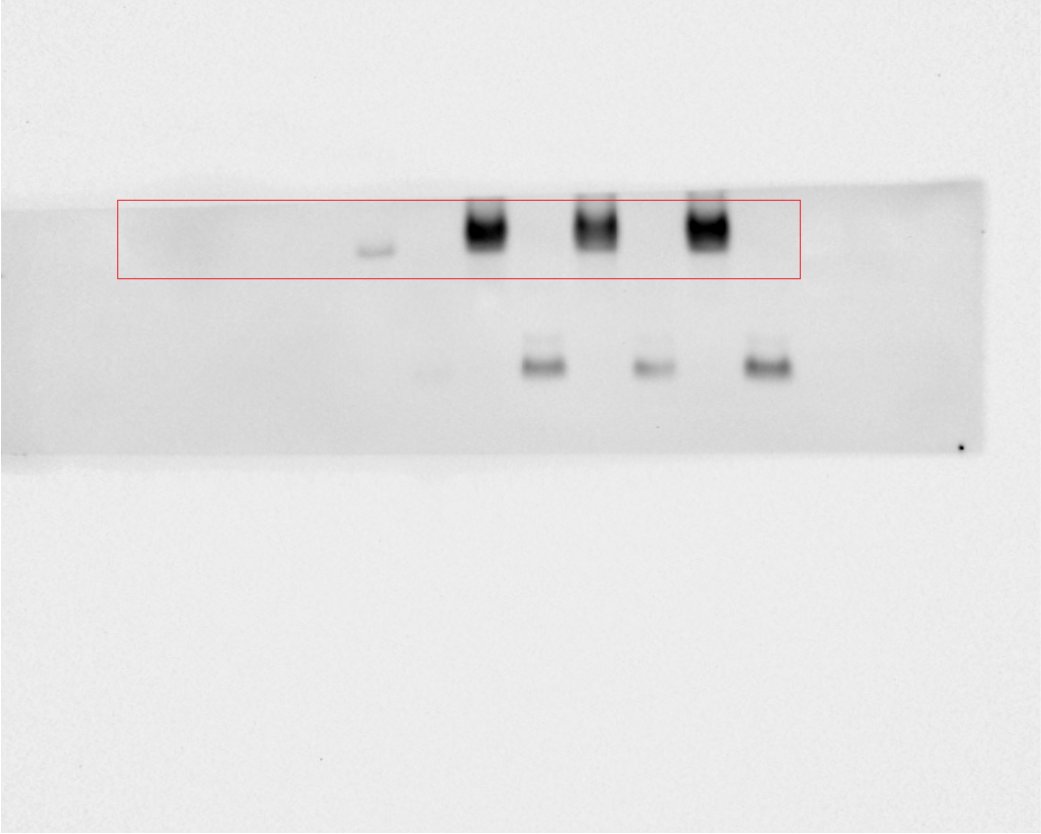

Supplement: Supplementary file 4 — Source data Fig. 2 [file 44319_2024_181_MOESM4_ESM.zip › Figure 2/Figure 2A/BNIP3.png]

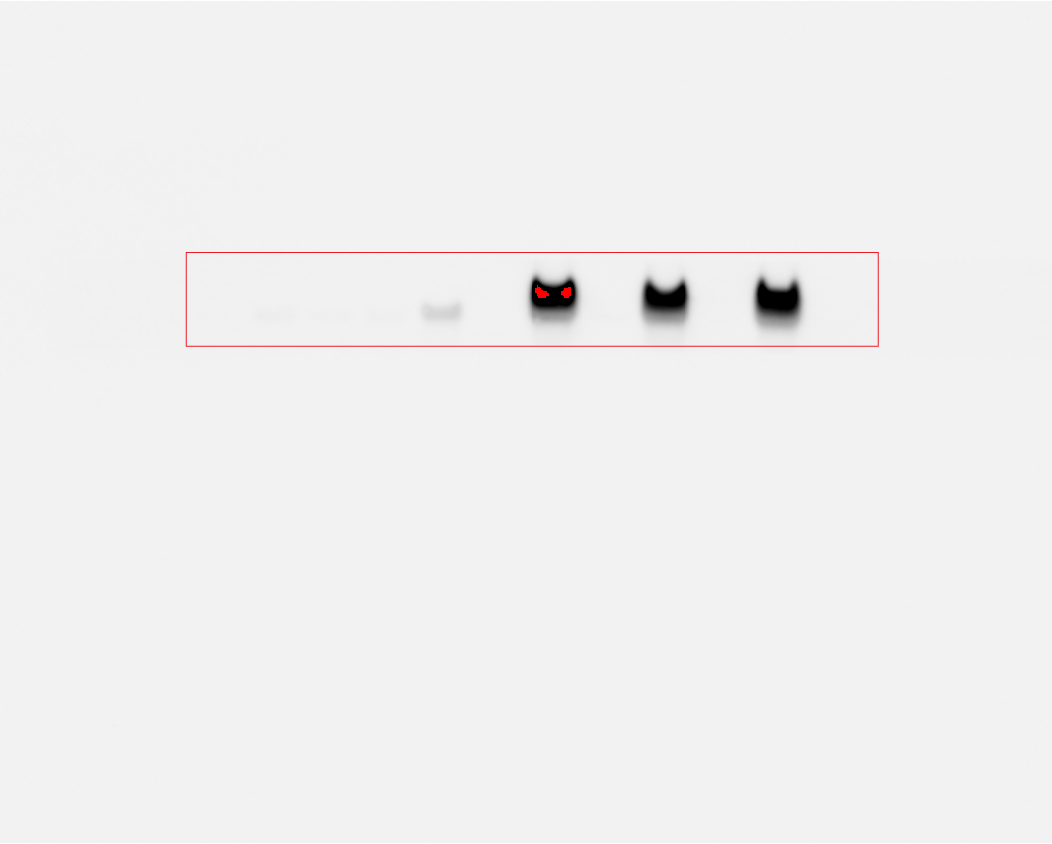

Supplement: Supplementary file 4 — Source data Fig. 2 [file 44319_2024_181_MOESM4_ESM.zip › Figure 2/Figure 2A/NIX.png]

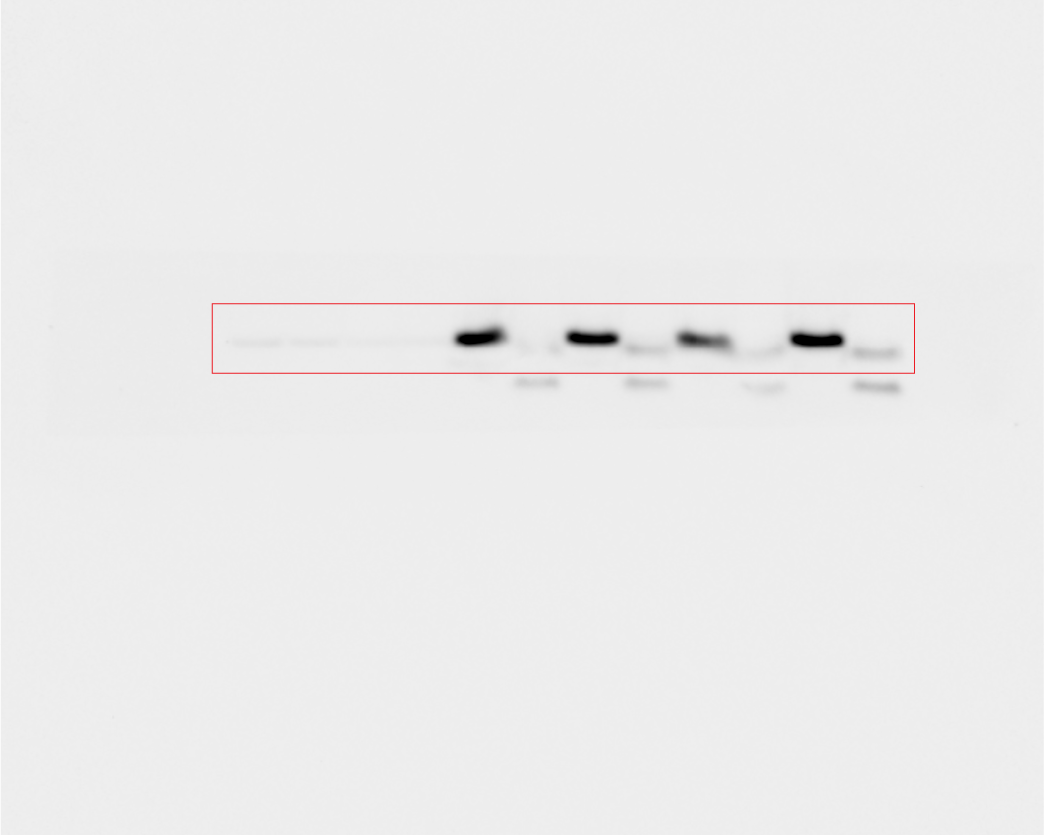

Supplement: Supplementary file 4 — Source data Fig. 2 [file 44319_2024_181_MOESM4_ESM.zip › Figure 2/Figure 2A/TOM20.png]

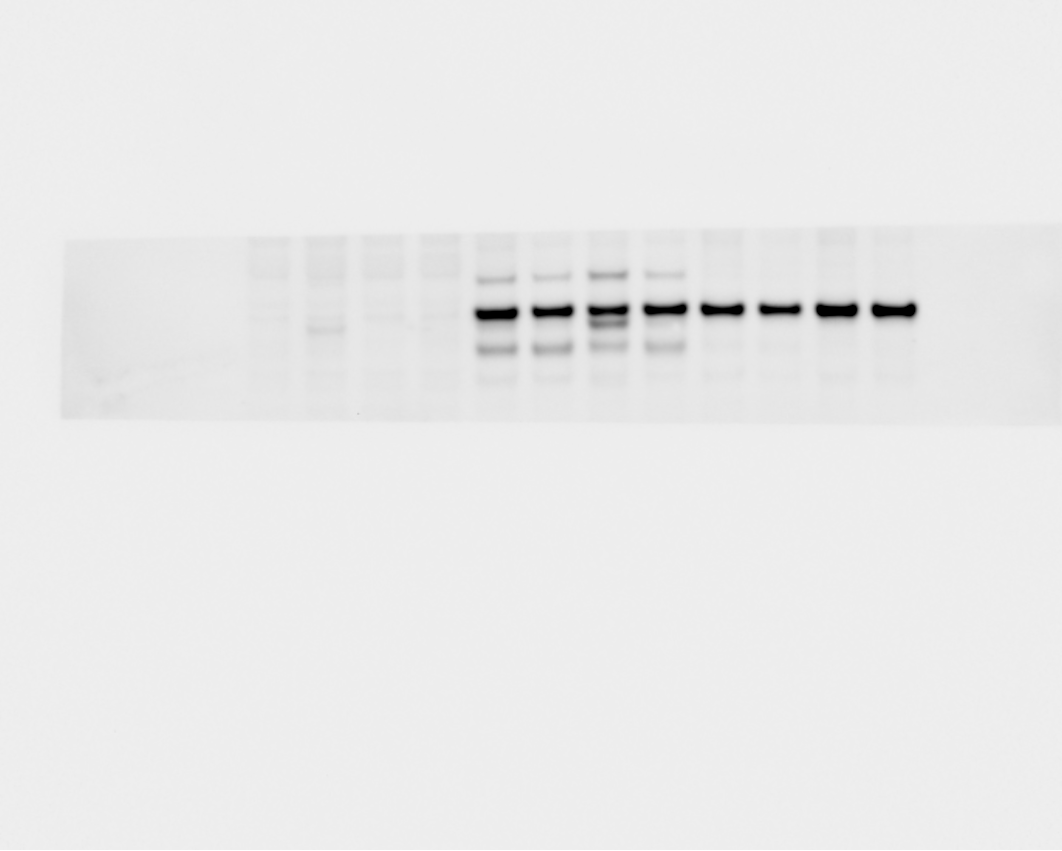

Supplement: Supplementary file 4 — Source data Fig. 2 [file 44319_2024_181_MOESM4_ESM.zip › Figure 2/Figure 2A/(PPTC7) Pagan Lab 2023-09-06 15h51m12s(Chemiluminescence).tif]

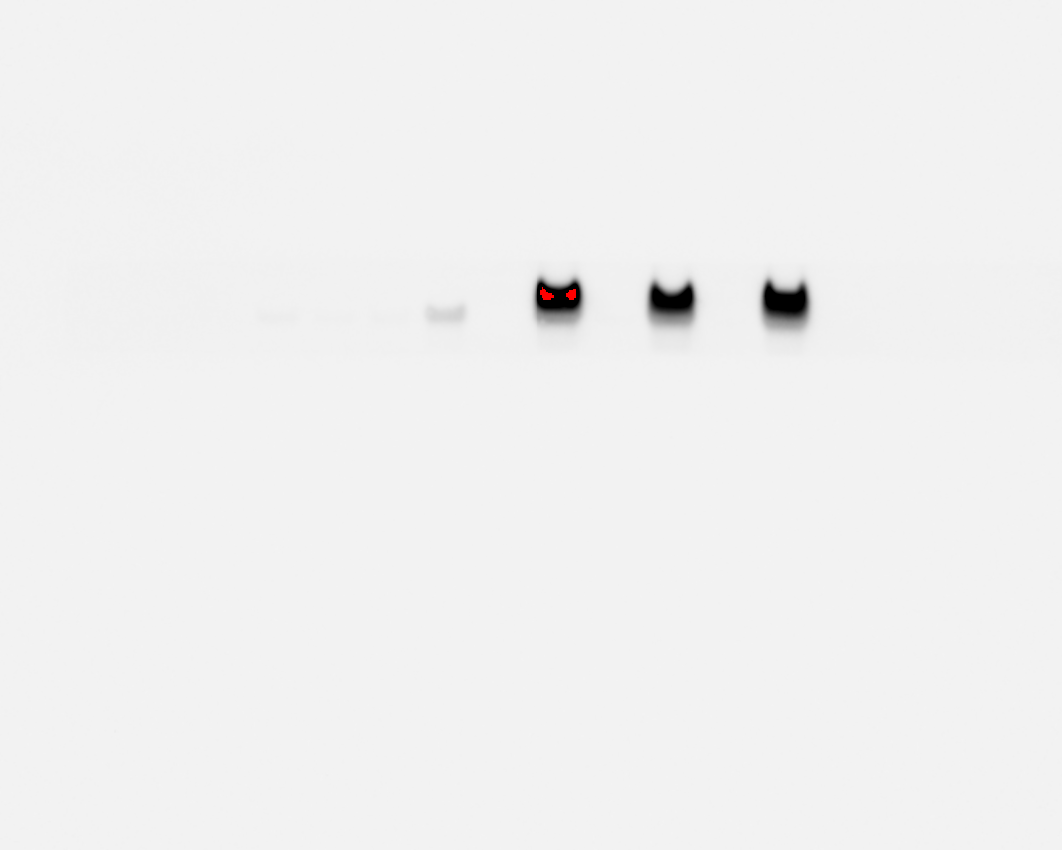

Supplement: Supplementary file 4 — Source data Fig. 2 [file 44319_2024_181_MOESM4_ESM.zip › Figure 2/Figure 2A/(NIX) Pagan Lab 2023-09-06 16h07m37s(Chemiluminescence).tif]

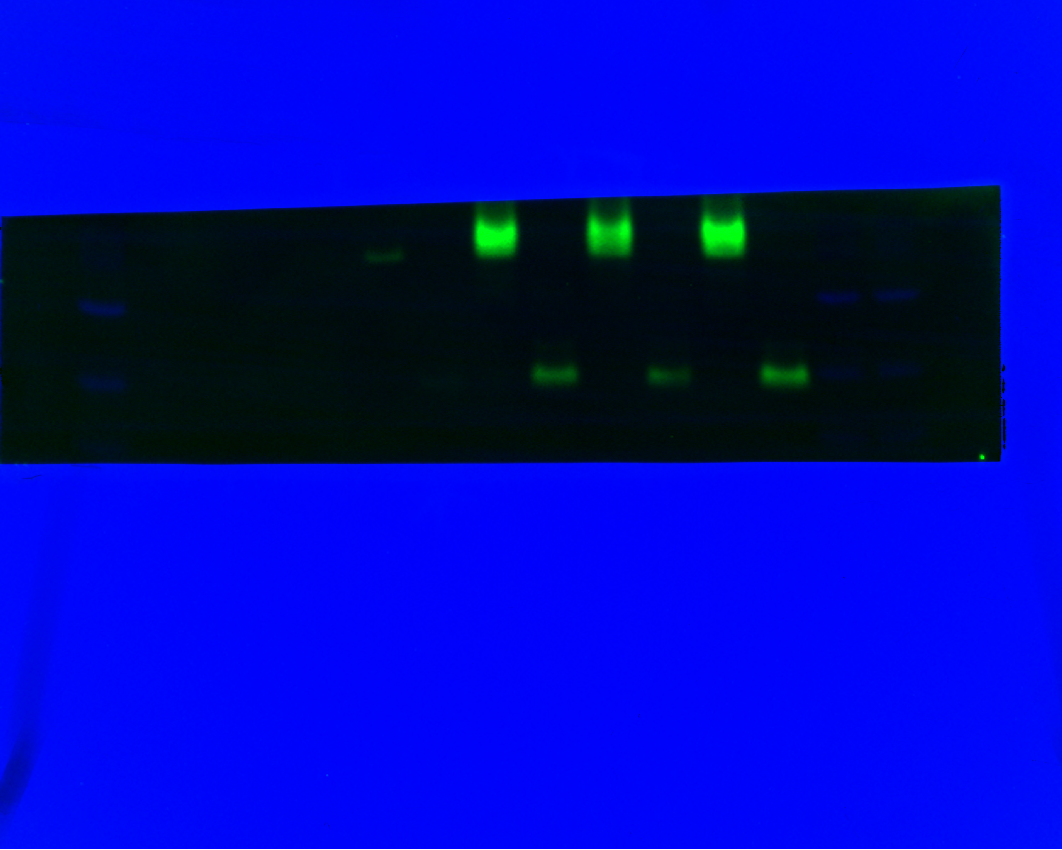

Supplement: Supplementary file 4 — Source data Fig. 2 [file 44319_2024_181_MOESM4_ESM.zip › Figure 2/Figure 2A/(BNIP3) (Composite).tif]

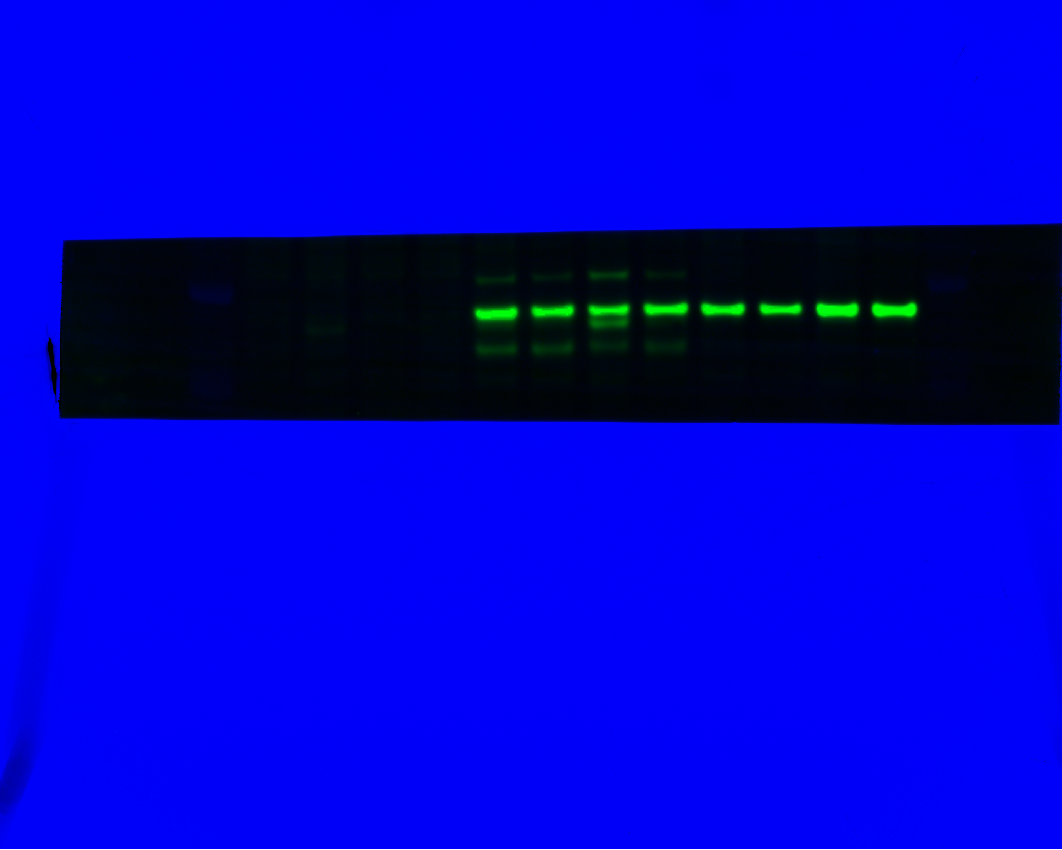

Supplement: Supplementary file 4 — Source data Fig. 2 [file 44319_2024_181_MOESM4_ESM.zip › Figure 2/Figure 2A/(PPTC7)(Composite).tif]

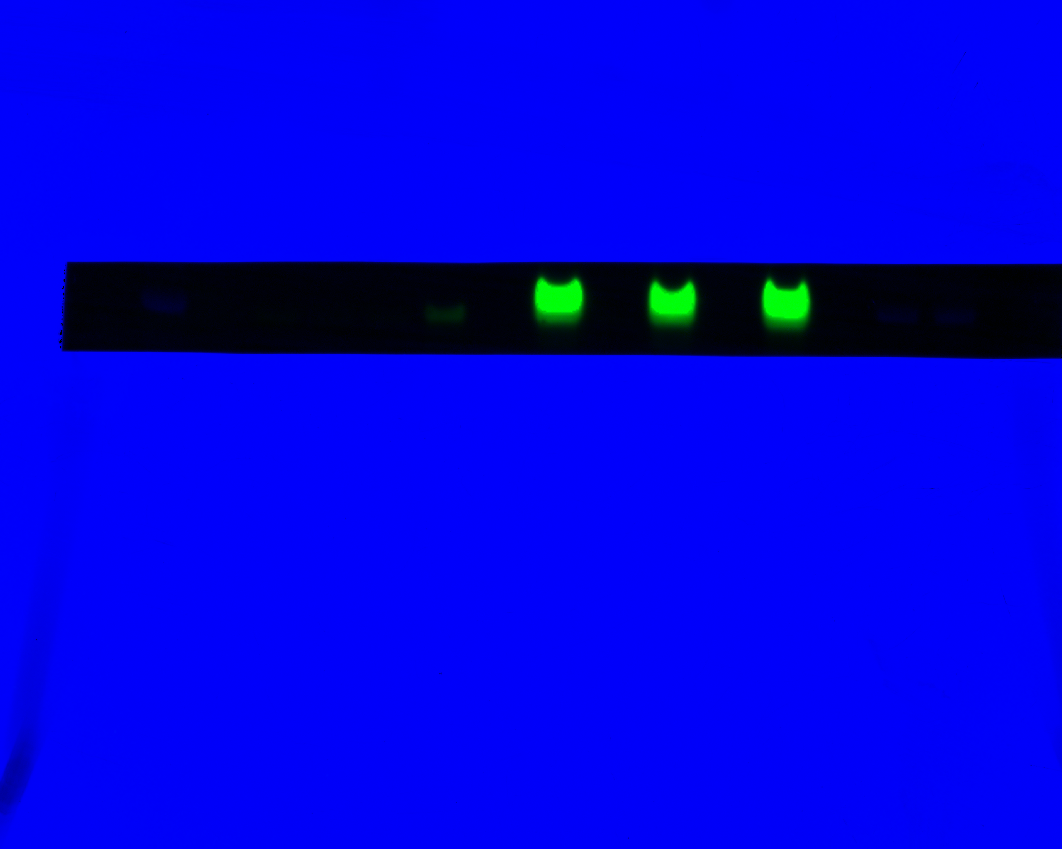

Supplement: Supplementary file 4 — Source data Fig. 2 [file 44319_2024_181_MOESM4_ESM.zip › Figure 2/Figure 2A/(NIX)(Composite).tif]

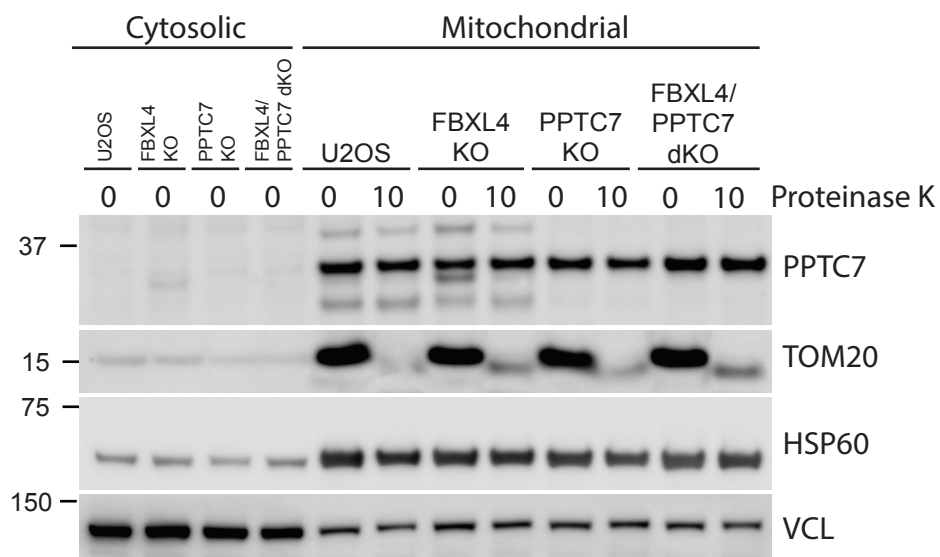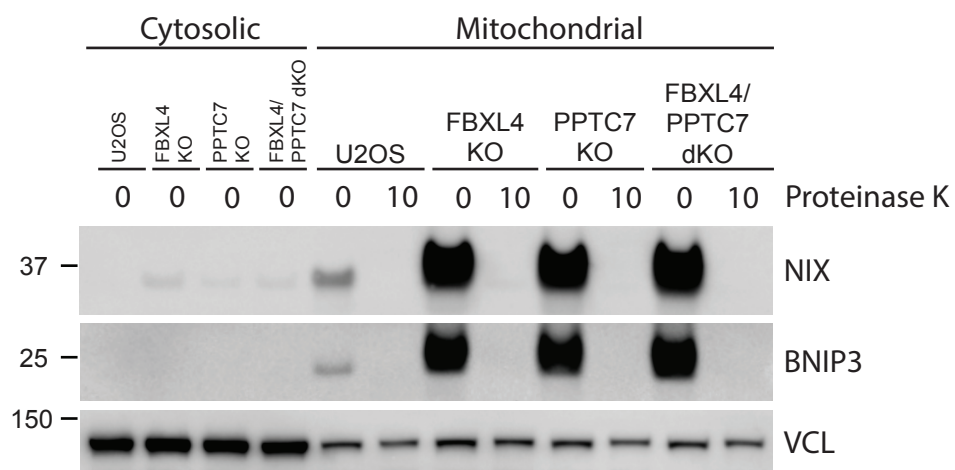

Supplement: Supplementary file 4 — Source data Fig. 2 [file 44319_2024_181_MOESM4_ESM.zip › Figure 2/Figure 2A/Annotation Figure 2A.pdf]

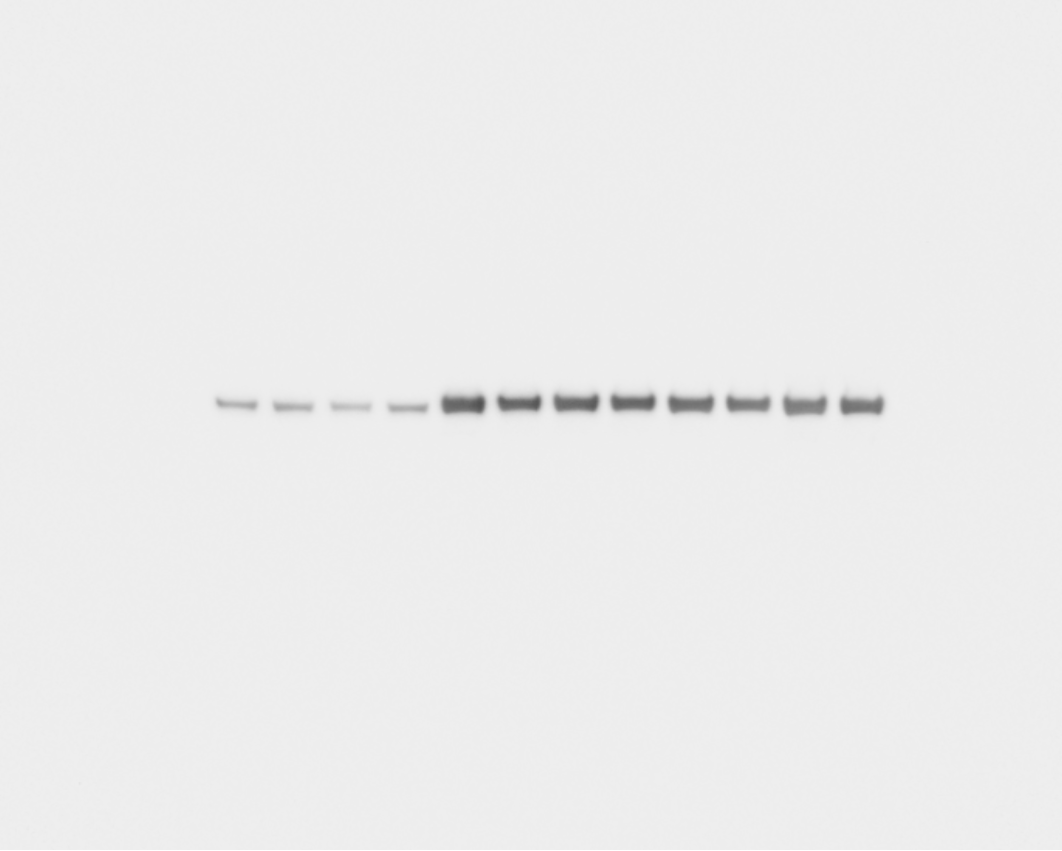

Supplement: Supplementary file 4 — Source data Fig. 2 [file 44319_2024_181_MOESM4_ESM.zip › Figure 2/Figure 2A/(HSP60)(Chemiluminescence).tif]

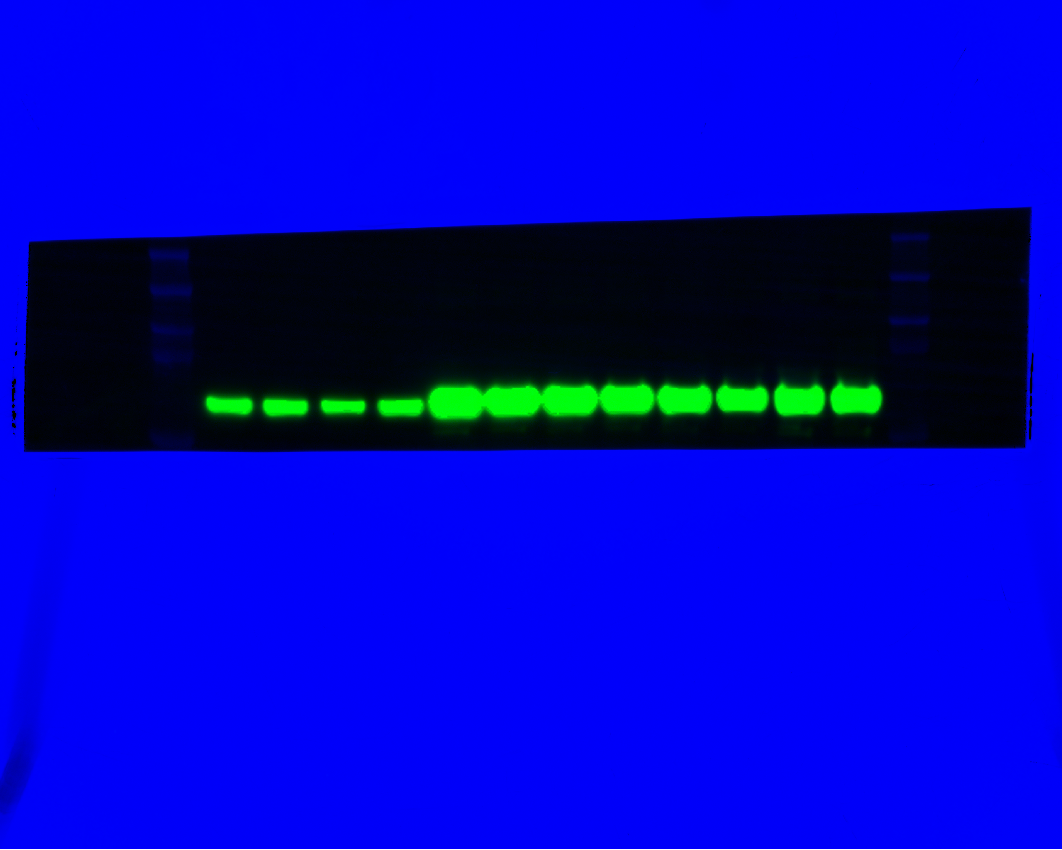

Supplement: Supplementary file 4 — Source data Fig. 2 [file 44319_2024_181_MOESM4_ESM.zip › Figure 2/Figure 2A/(HSP60) (Composite).tif]

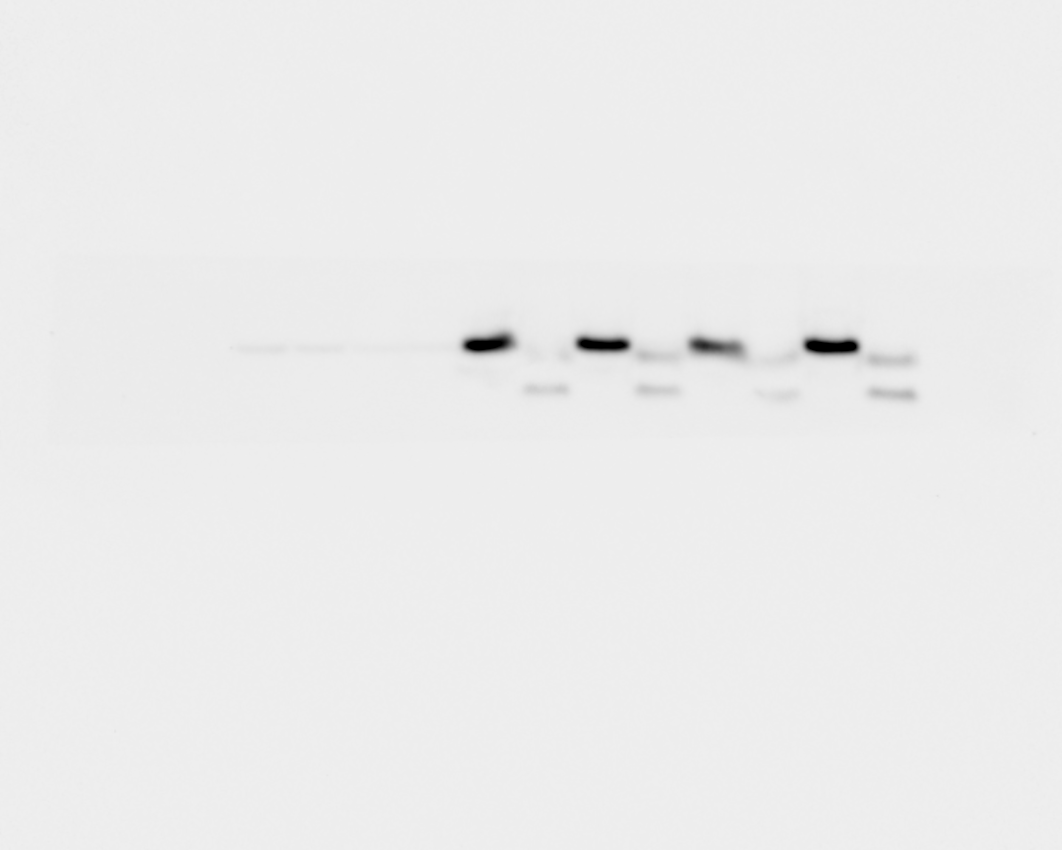

Supplement: Supplementary file 4 — Source data Fig. 2 [file 44319_2024_181_MOESM4_ESM.zip › Figure 2/Figure 2A/(TOM20)(Chemiluminescence).tif]

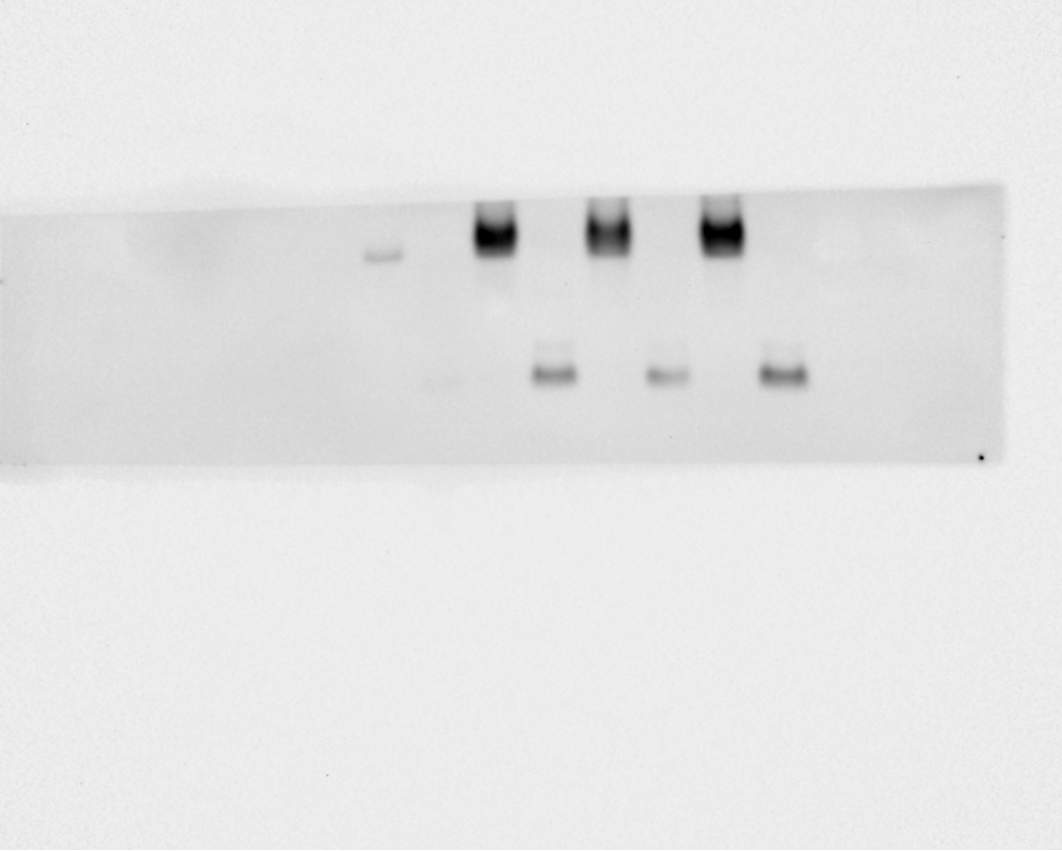

Supplement: Supplementary file 4 — Source data Fig. 2 [file 44319_2024_181_MOESM4_ESM.zip › Figure 2/Figure 2A/(BNIP3)(Chemiluminescence).tif]

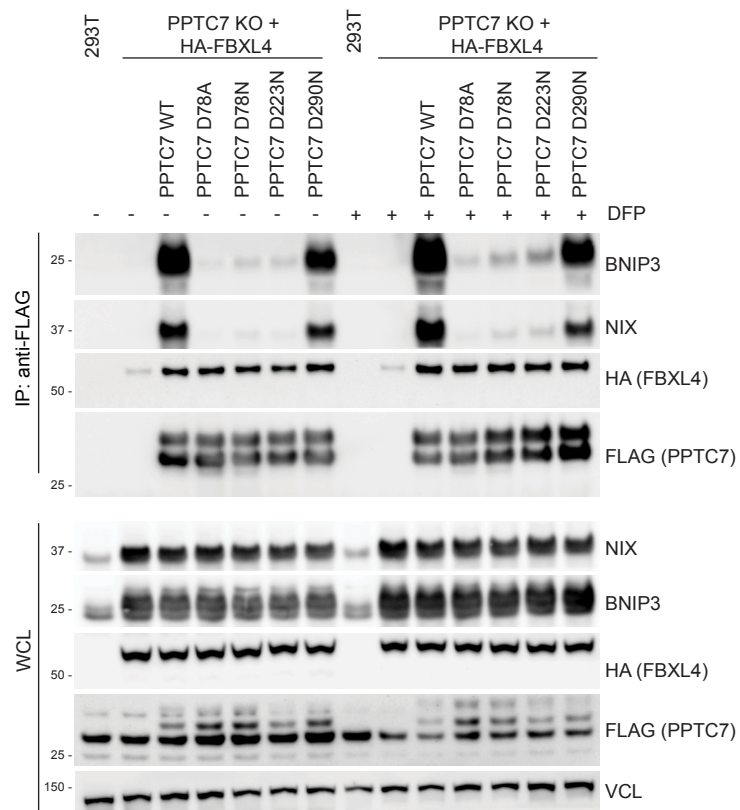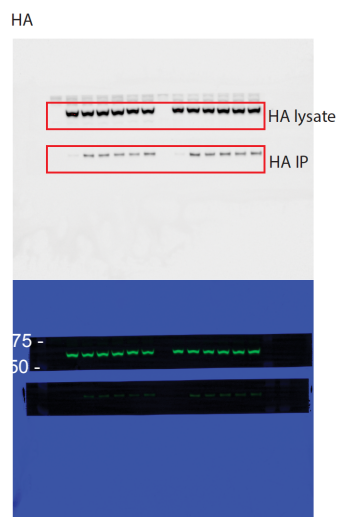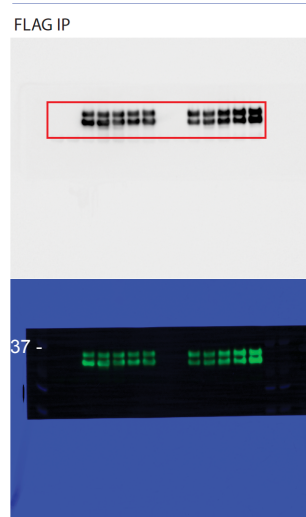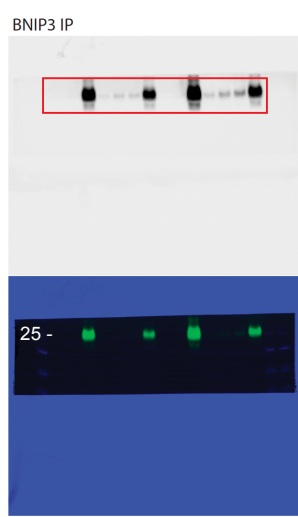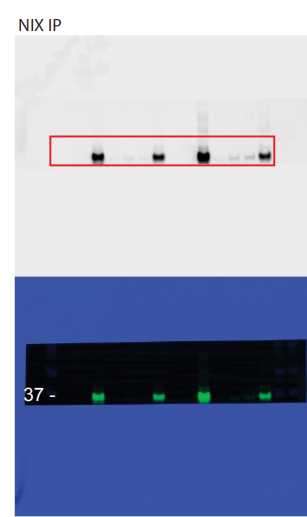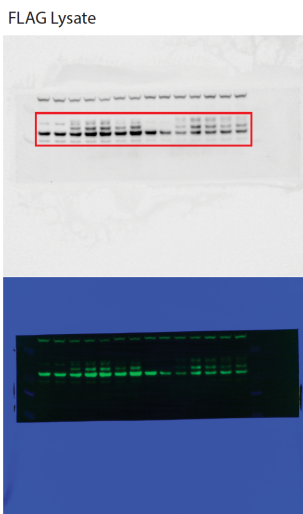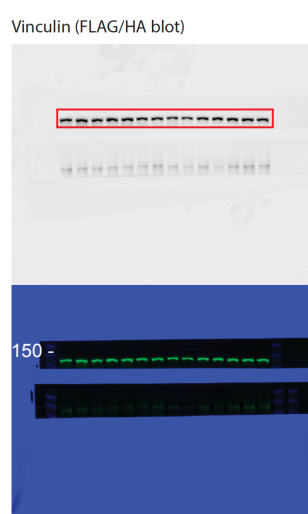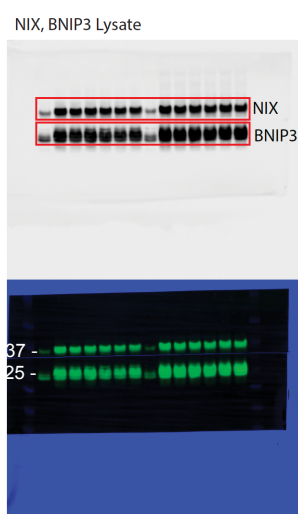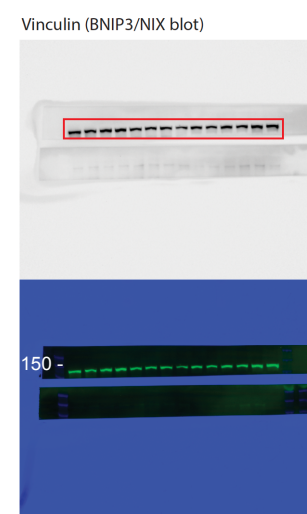

Supplement: Supplementary file 5 — Source data Fig. 3 [file 44319_2024_181_MOESM5_ESM.zip › Figure 3/Figure 3B/Annotated Figure 3B.pdf]

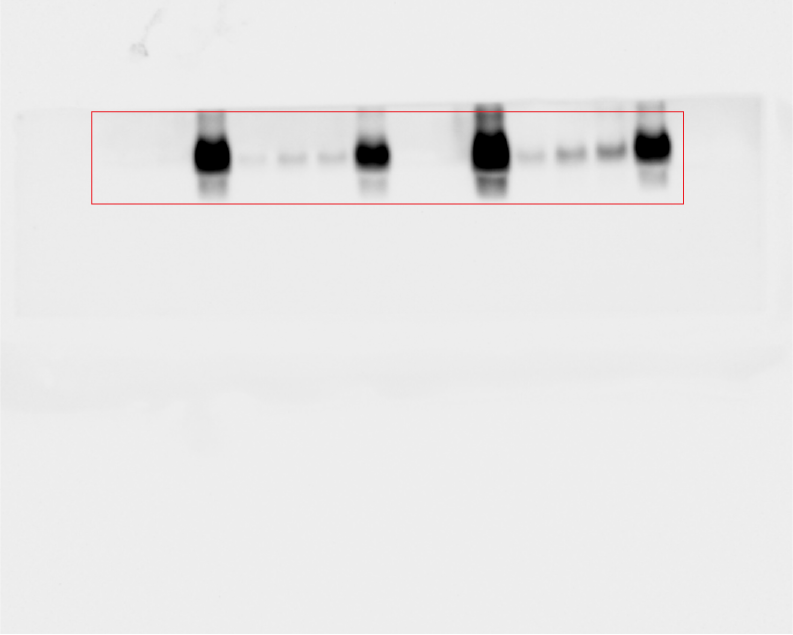

Supplement: Supplementary file 5 — Source data Fig. 3 [file 44319_2024_181_MOESM5_ESM.zip › Figure 3/Figure 3B/BNIP3 IP.png]

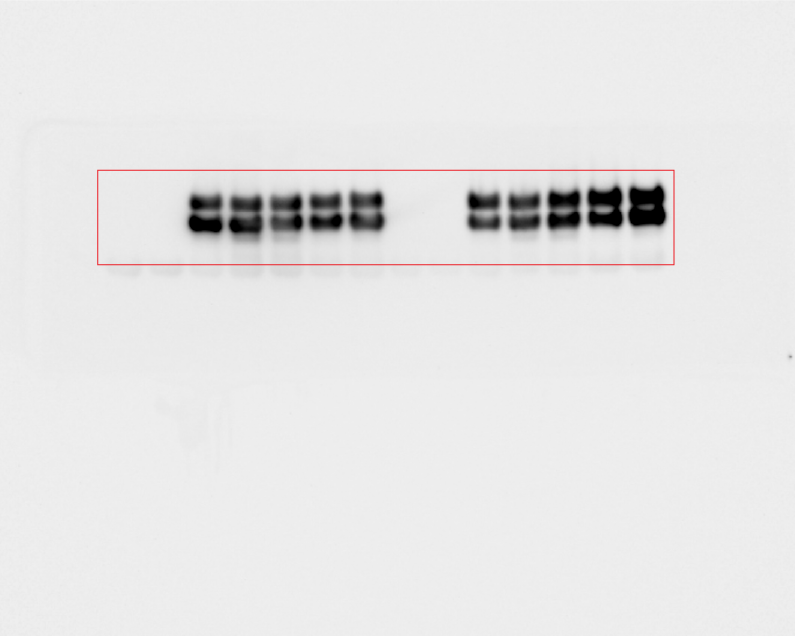

Supplement: Supplementary file 5 — Source data Fig. 3 [file 44319_2024_181_MOESM5_ESM.zip › Figure 3/Figure 3B/FLAG IP.png]

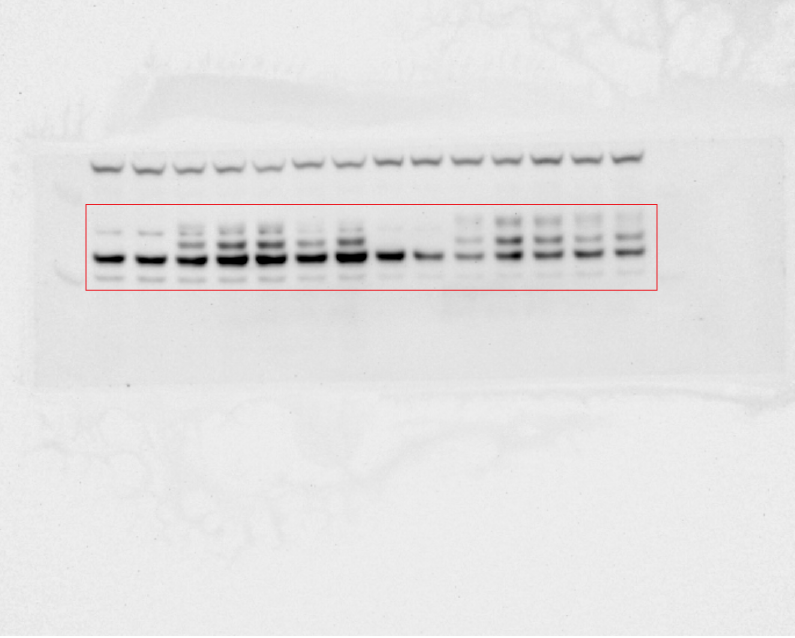

Supplement: Supplementary file 5 — Source data Fig. 3 [file 44319_2024_181_MOESM5_ESM.zip › Figure 3/Figure 3B/FLAG lysate.png]

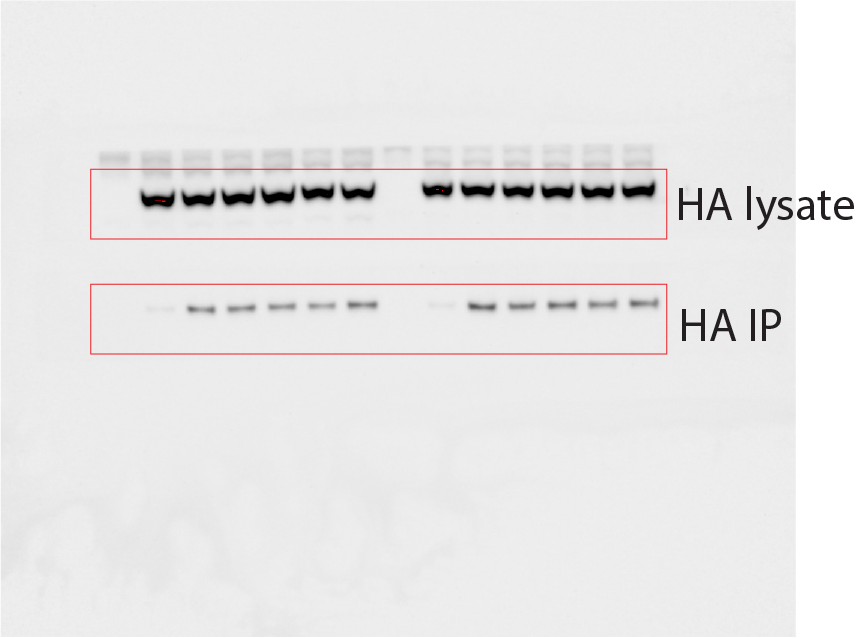

Supplement: Supplementary file 5 — Source data Fig. 3 [file 44319_2024_181_MOESM5_ESM.zip › Figure 3/Figure 3B/HA.png]

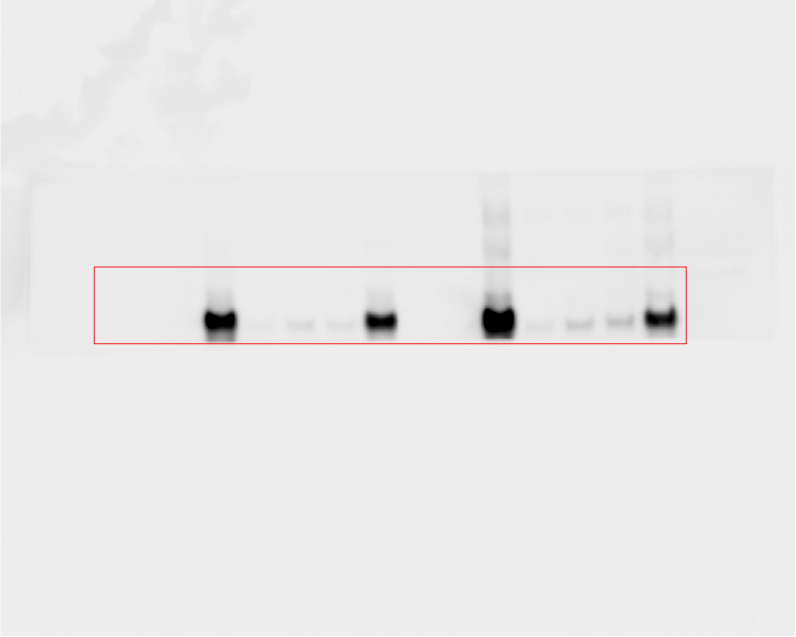

Supplement: Supplementary file 5 — Source data Fig. 3 [file 44319_2024_181_MOESM5_ESM.zip › Figure 3/Figure 3B/NIX IP.png]

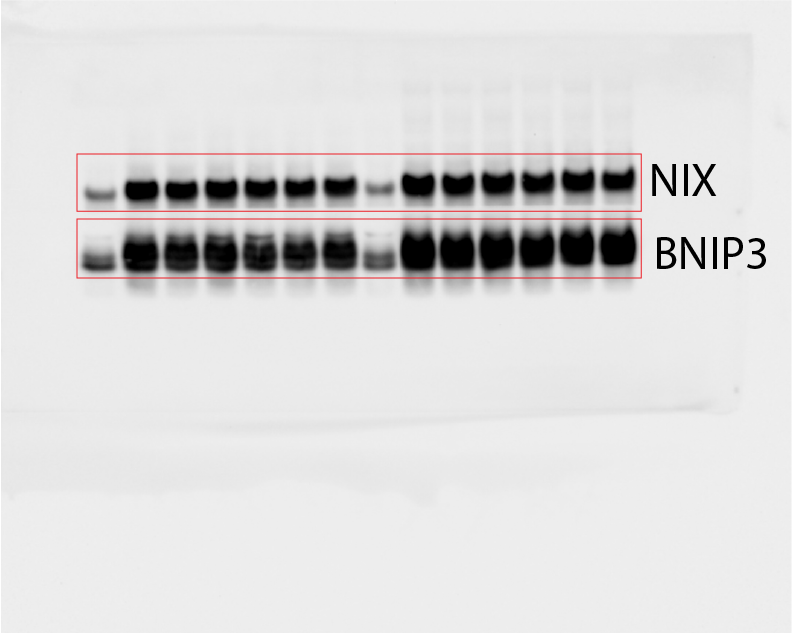

Supplement: Supplementary file 5 — Source data Fig. 3 [file 44319_2024_181_MOESM5_ESM.zip › Figure 3/Figure 3B/NIX, BNIP3 lysate.png]

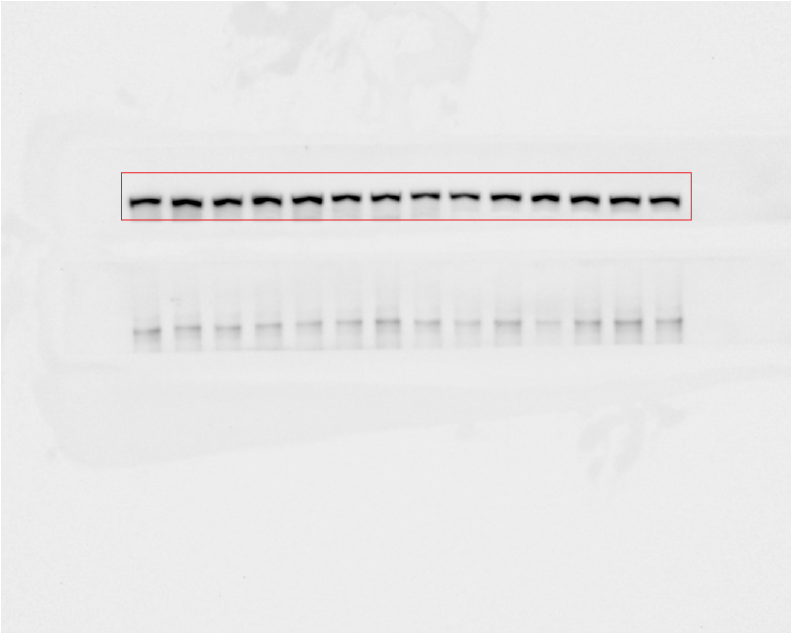

Supplement: Supplementary file 5 — Source data Fig. 3 [file 44319_2024_181_MOESM5_ESM.zip › Figure 3/Figure 3B/Vinculin (FLAG, HA blot).png]

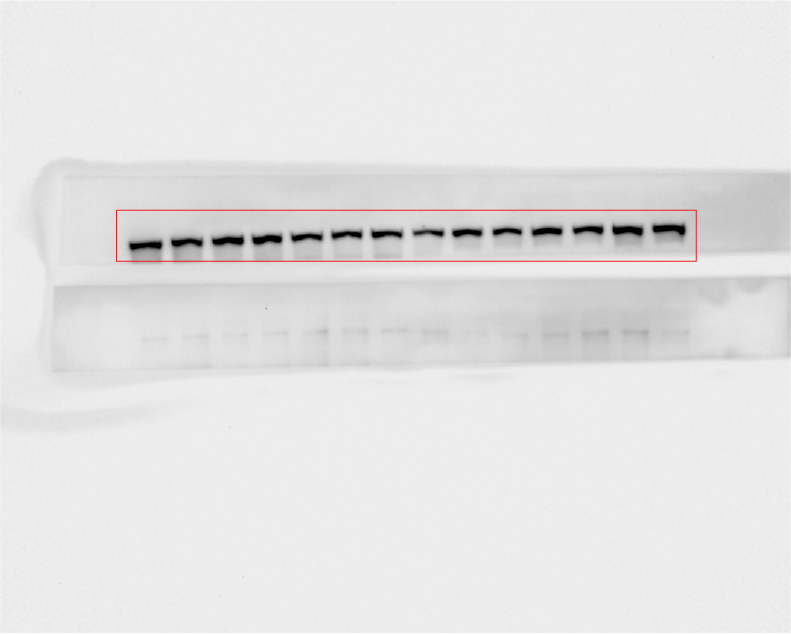

Supplement: Supplementary file 5 — Source data Fig. 3 [file 44319_2024_181_MOESM5_ESM.zip › Figure 3/Figure 3B/Vinculin (NIX, BNIP3 blot).png]

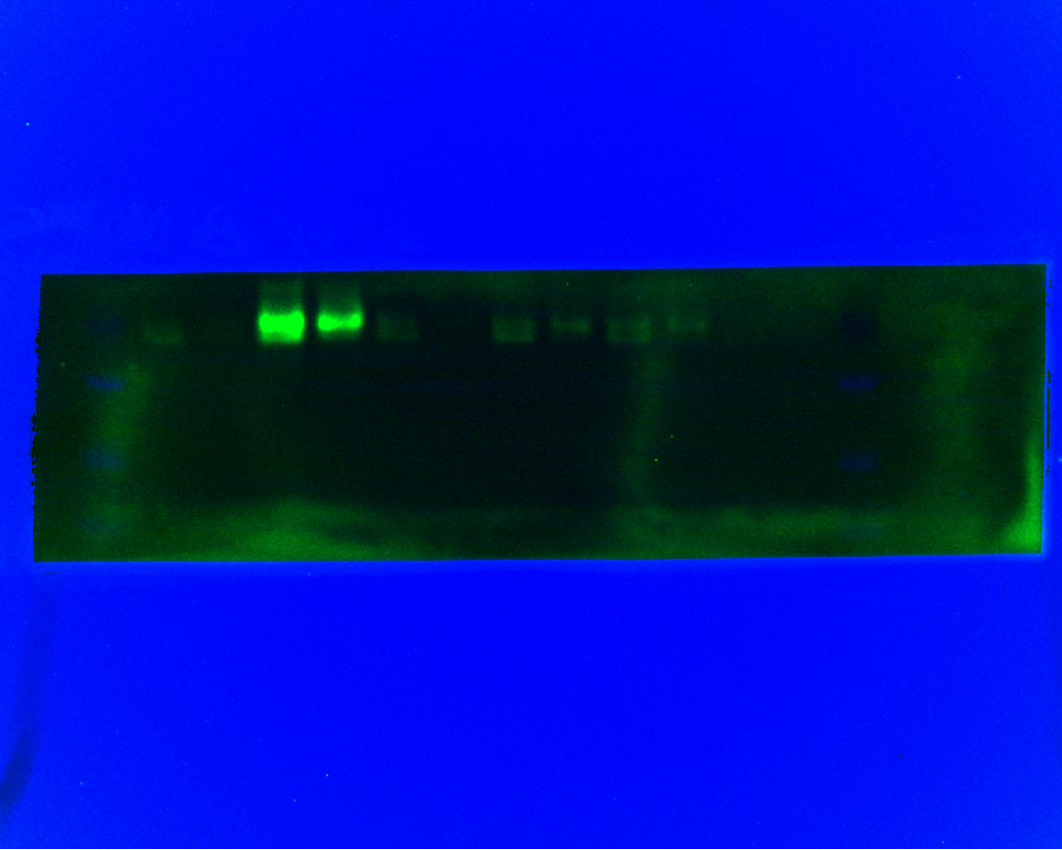

Supplement: Supplementary file 5 — Source data Fig. 3 [file 44319_2024_181_MOESM5_ESM.zip › Figure 3/Figure 3C/(BNIP3)(Composite).tif]
