# Supplementary material for: PPTC7 antagonizes mitophagy by promoting BNIP3 and NIX degradation via SCFFBXL4
Source: EMBO Rep. 2024 Jul 11;25(8):3324–47. doi: 10.1038/s44319-024-00181-y (PMC11316107; doi:10.1038/s44319-024-00181-y)
Supplement: Supplementary file 4 — Source data Fig. 2 [file 44319_2024_181_MOESM4_ESM.zip › Figure 2/Figure 2E/Annotations Figure 2E.pdf]

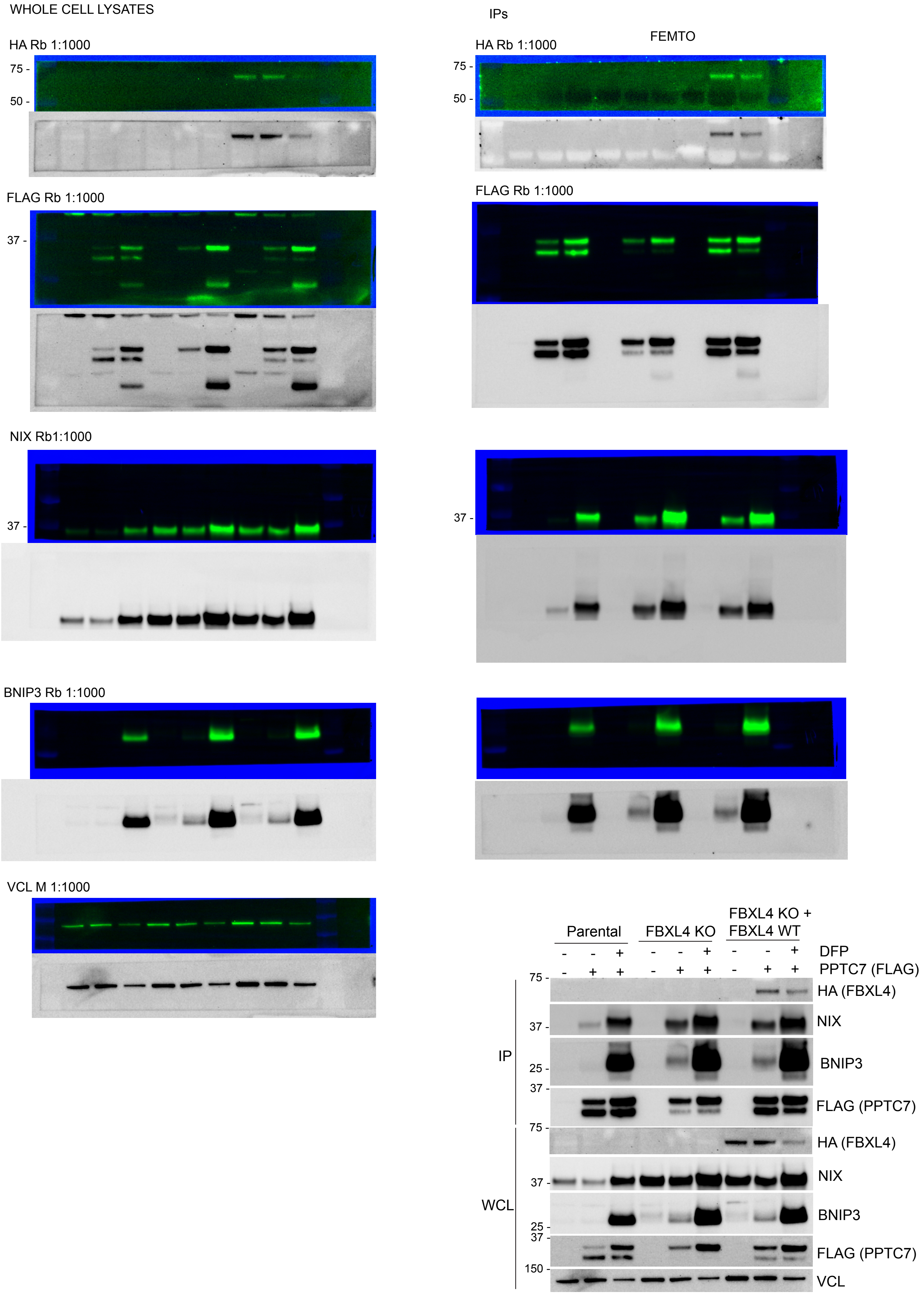
WHOLE CELL LYSATES
HA Rb 1:1000
75 -
50 -
FLAG Rb 1:1000
37 -
NIX Rb1:1000
37 -
BNIP3 Rb 1:1000
VCL M 1:1000
IPs
FEMTO
HA Rb 1:1000
75 -
50 -
FLAG Rb 1:1000
37 -
Parental
FBXL4 KO
FBXL4 KO +
FBXL4 WT
DFP
PPTC7 (FLAG)
75 -
37 -
25 -
37 -
75 -
37 -
25 -
37 -
150 -
IP
WCL
HA (FBXL4)
NIX
BNIP3
FLAG (PPTC7)
HA (FBXL4)
NIX
BNIP3
FLAG (PPTC7)
VCL
