# Supplementary figures and images for: PPTC7 antagonizes mitophagy by promoting BNIP3 and NIX degradation via SCFFBXL4 (part 2 of 2)
Source: EMBO Rep. 2024 Jul 11;25(8):3324–47. doi: 10.1038/s44319-024-00181-y (PMC11316107; doi:10.1038/s44319-024-00181-y)

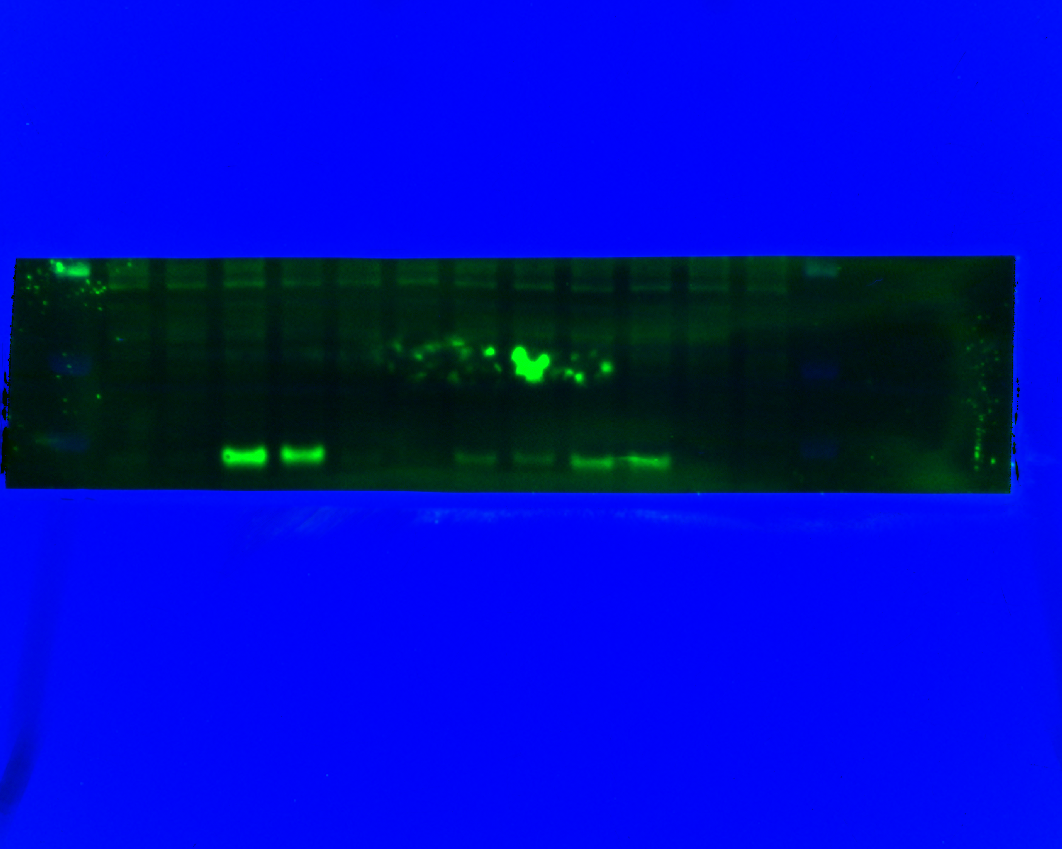

Supplement: Supplementary file 5 — Source data Fig. 3 [file 44319_2024_181_MOESM5_ESM.zip › Figure 3/Figure 3C/(NIX)(Composite).tif]

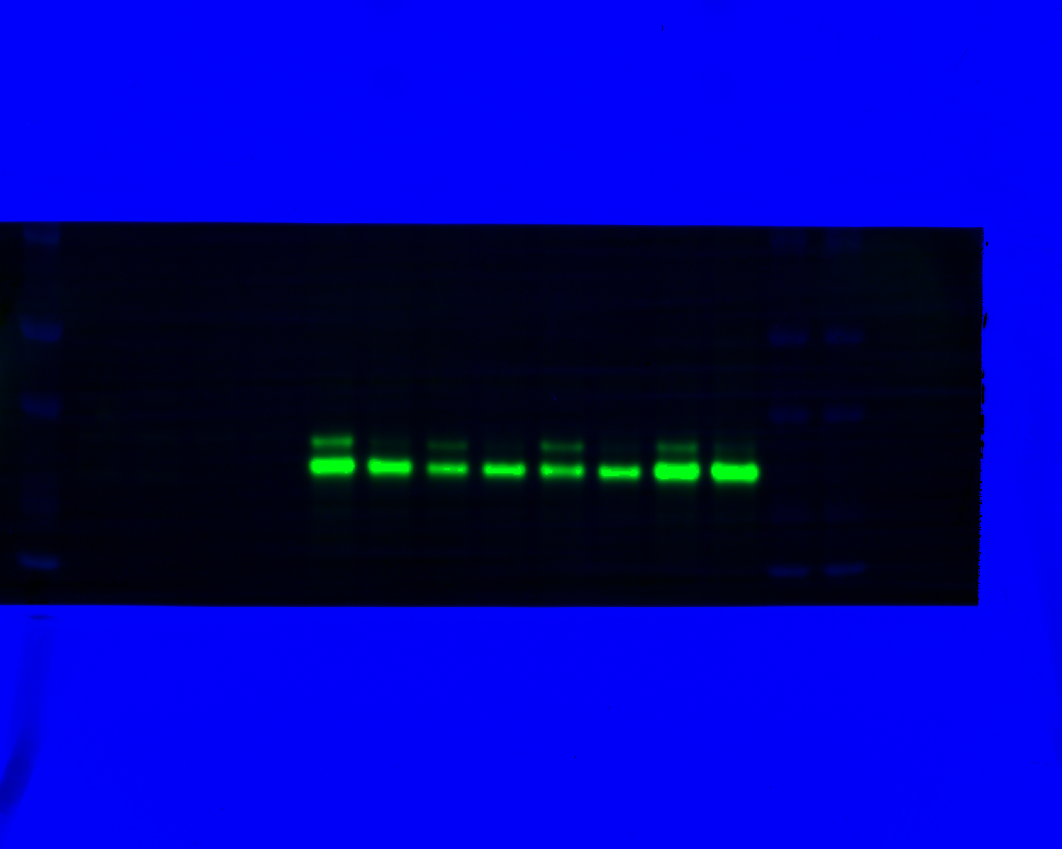

Supplement: Supplementary file 5 — Source data Fig. 3 [file 44319_2024_181_MOESM5_ESM.zip › Figure 3/Figure 3C/(PPTC7)(Composite).tif]

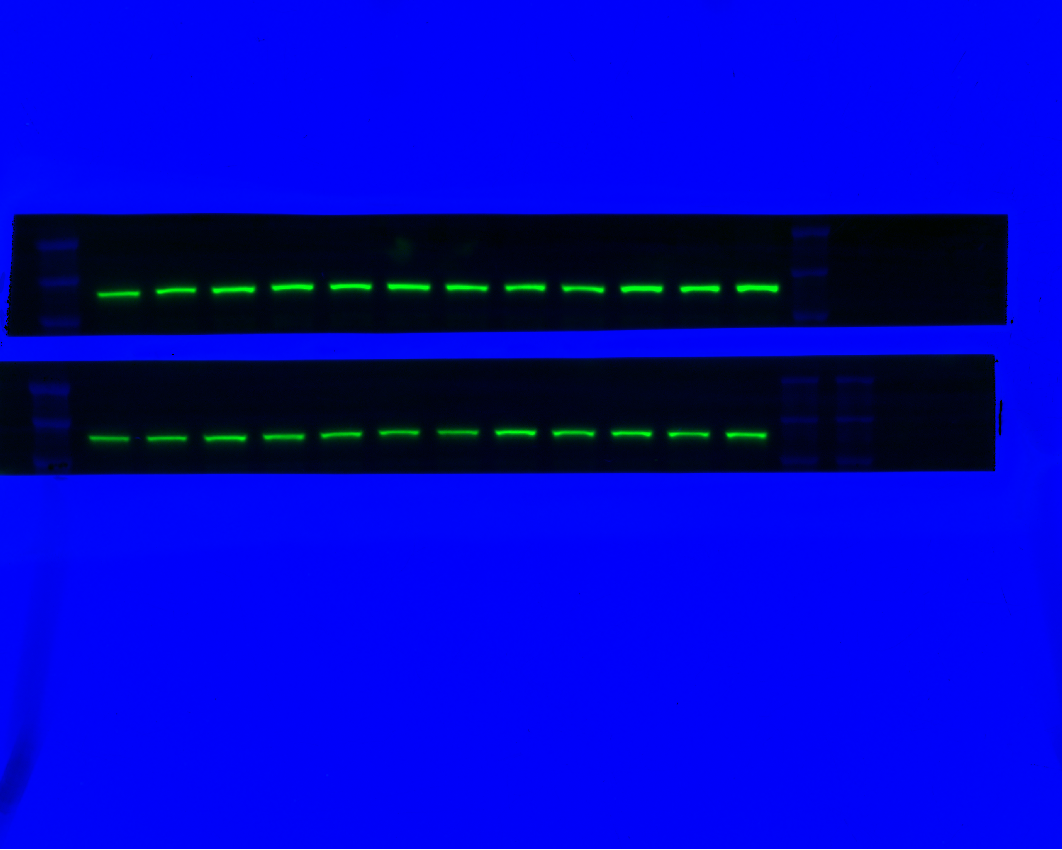

Supplement: Supplementary file 5 — Source data Fig. 3 [file 44319_2024_181_MOESM5_ESM.zip › Figure 3/Figure 3C/(VCL)(Composite).tif]

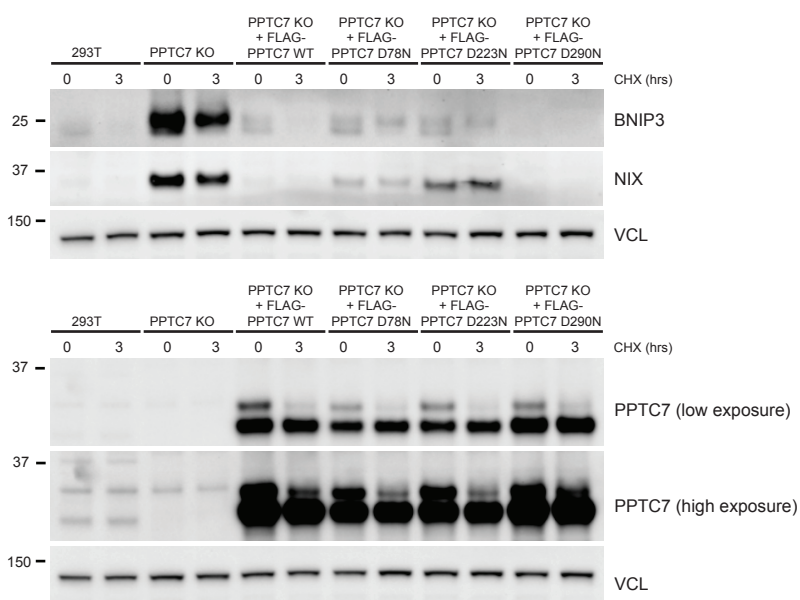

## NIX

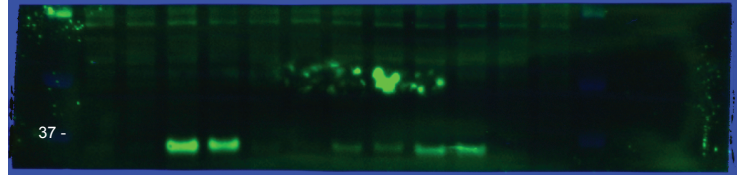

## PPTC7

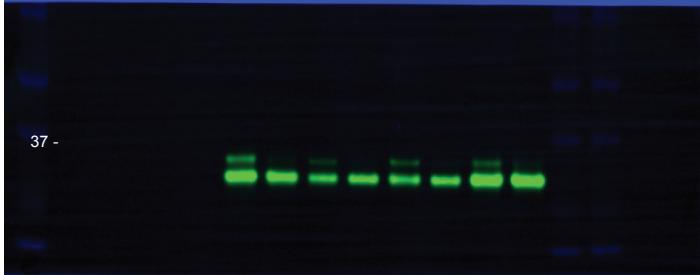

## BNIP3

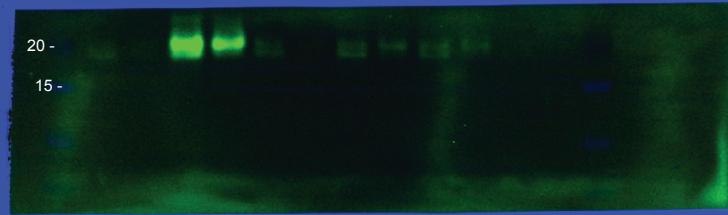

## VCL

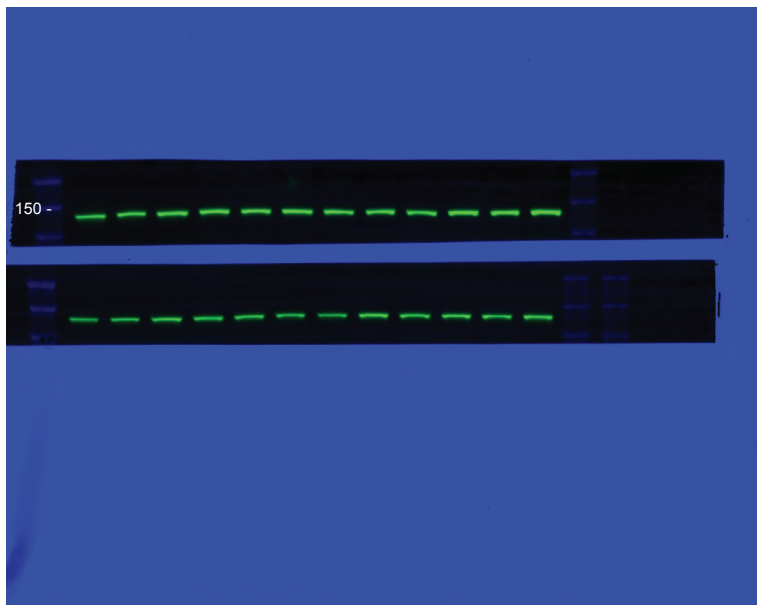

Supplement: Supplementary file 5 — Source data Fig. 3 [file 44319_2024_181_MOESM5_ESM.zip › Figure 3/Figure 3C/Annotations Figure 3C.pdf]

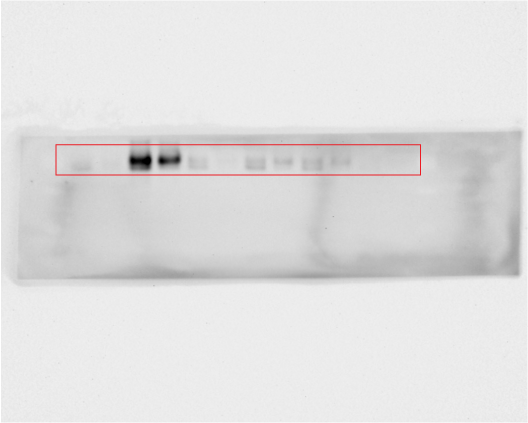

Supplement: Supplementary file 5 — Source data Fig. 3 [file 44319_2024_181_MOESM5_ESM.zip › Figure 3/Figure 3C/BNIP3.png]

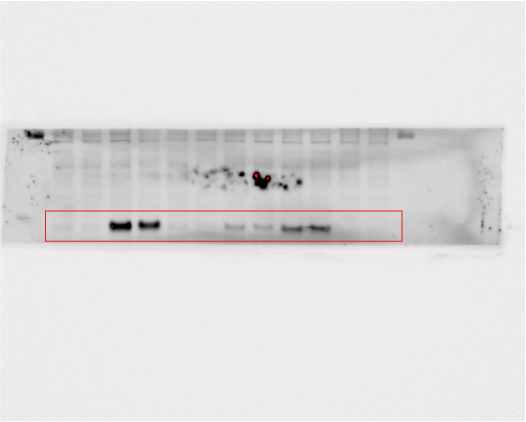

Supplement: Supplementary file 5 — Source data Fig. 3 [file 44319_2024_181_MOESM5_ESM.zip › Figure 3/Figure 3C/NIX.png]

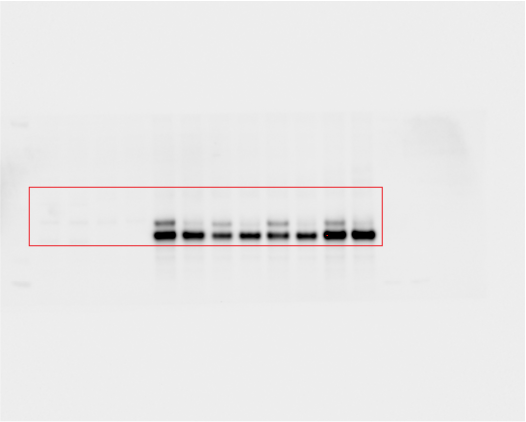

Supplement: Supplementary file 5 — Source data Fig. 3 [file 44319_2024_181_MOESM5_ESM.zip › Figure 3/Figure 3C/PPTC7.png]

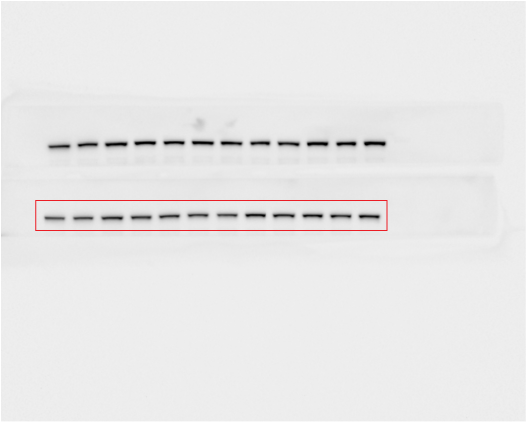

Supplement: Supplementary file 5 — Source data Fig. 3 [file 44319_2024_181_MOESM5_ESM.zip › Figure 3/Figure 3C/Vinculin.png]

Scale bar = 20uM

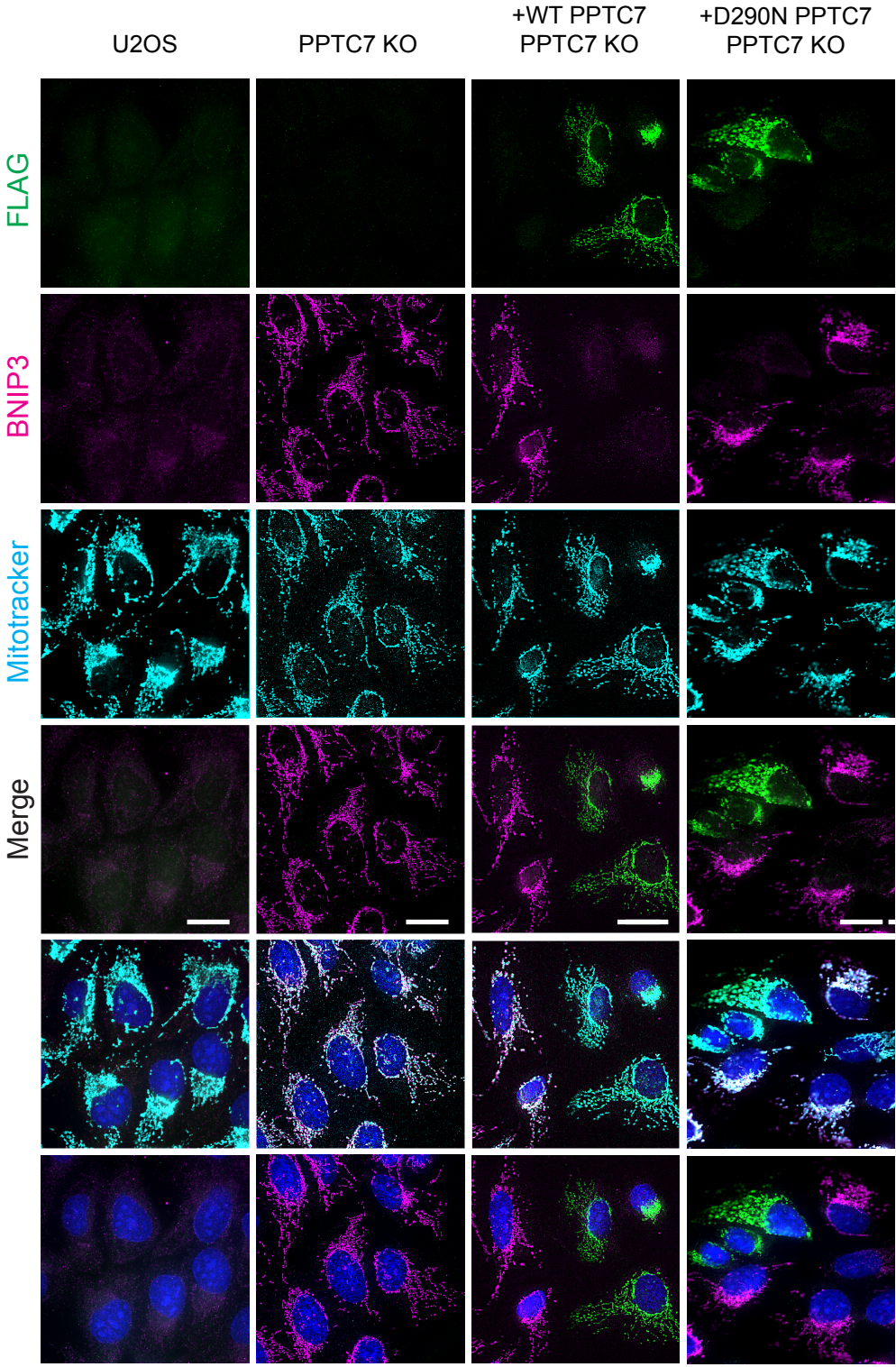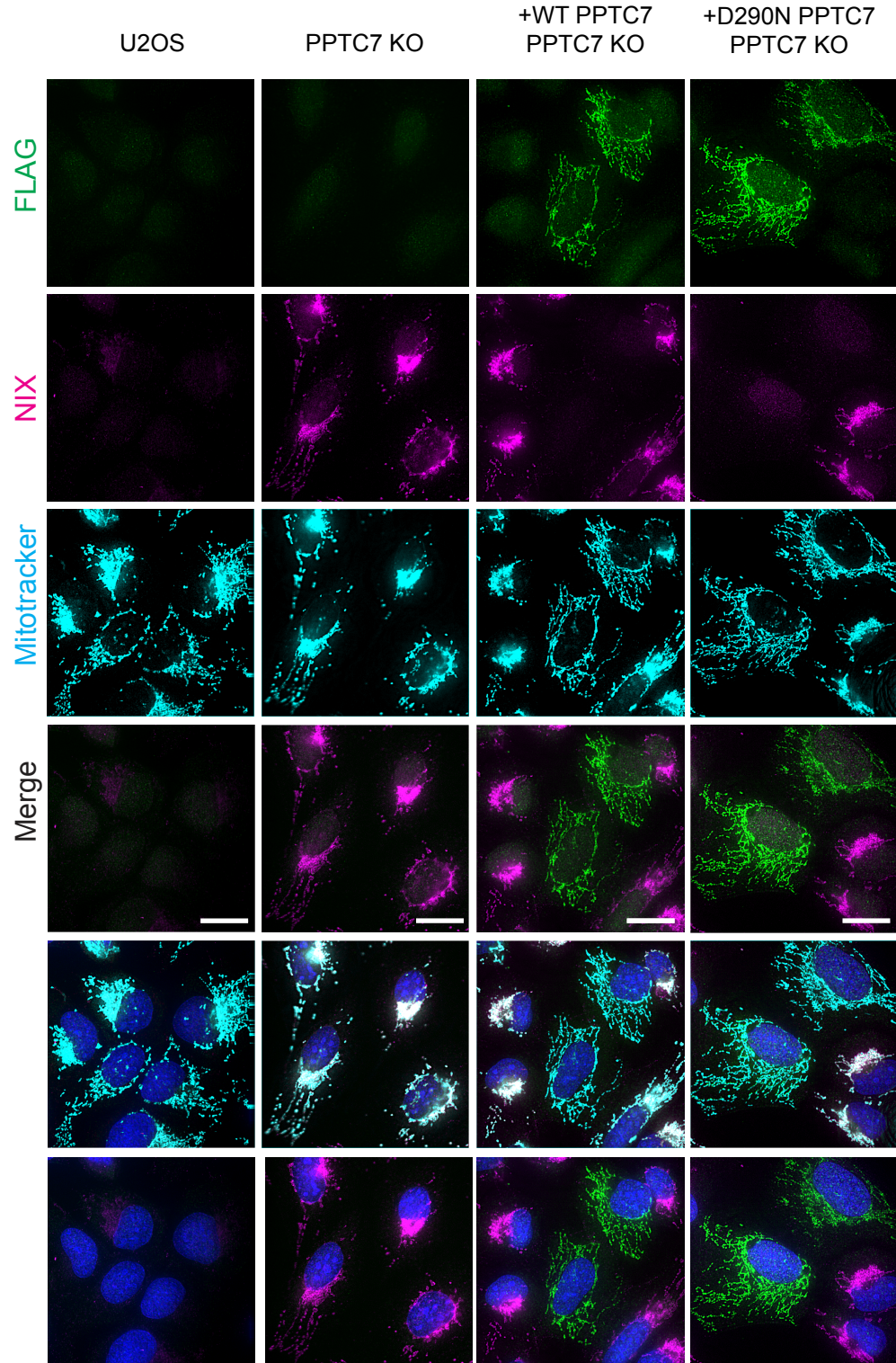

Supplement: Supplementary file 5 — Source data Fig. 3 [file 44319_2024_181_MOESM5_ESM.zip › Figure 3/Figure 3D/Annotations Figure 3D.pdf]

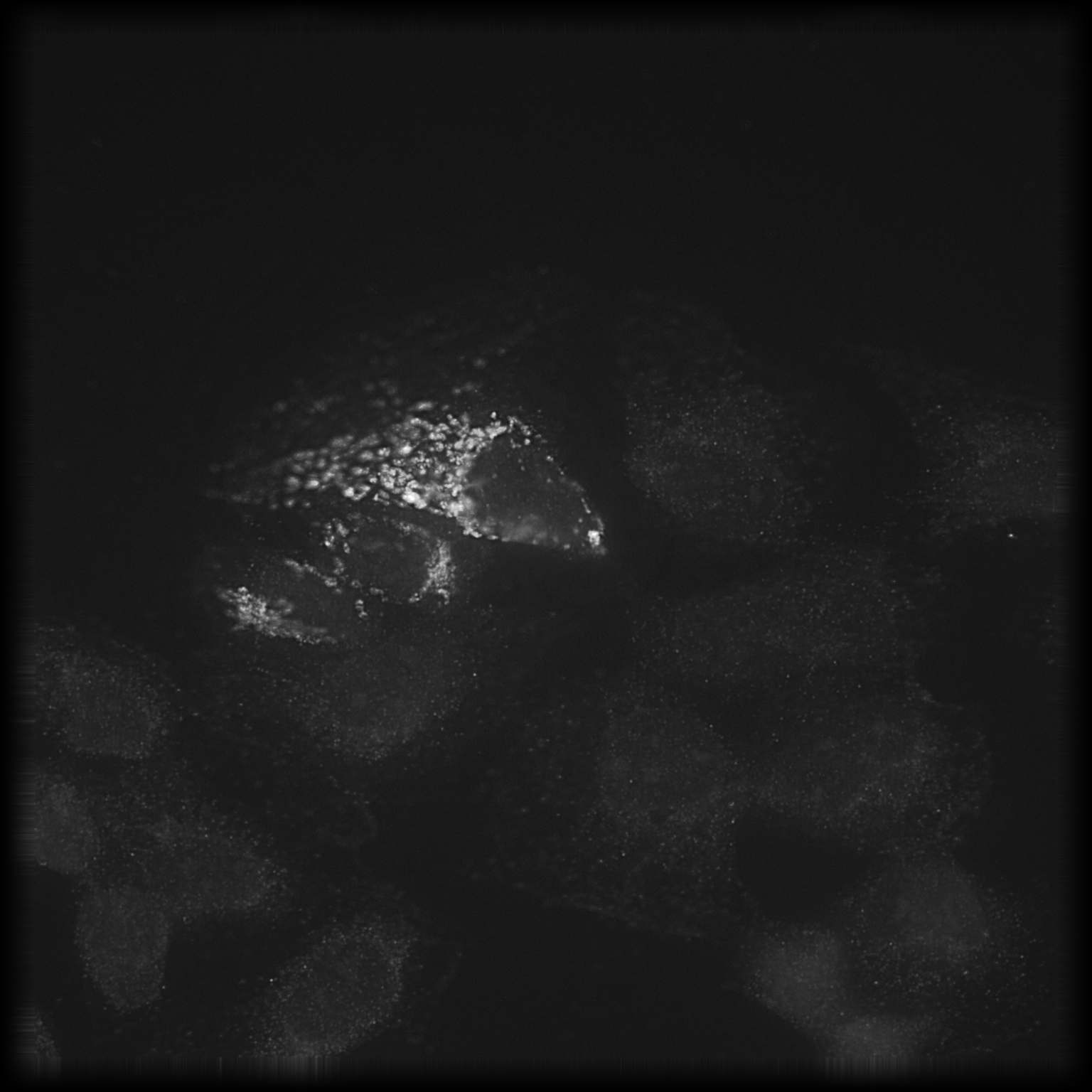

Supplement: Supplementary file 5 — Source data Fig. 3 [file 44319_2024_181_MOESM5_ESM.zip › Figure 3/Figure 3D/D290N_BNIP3.tif]

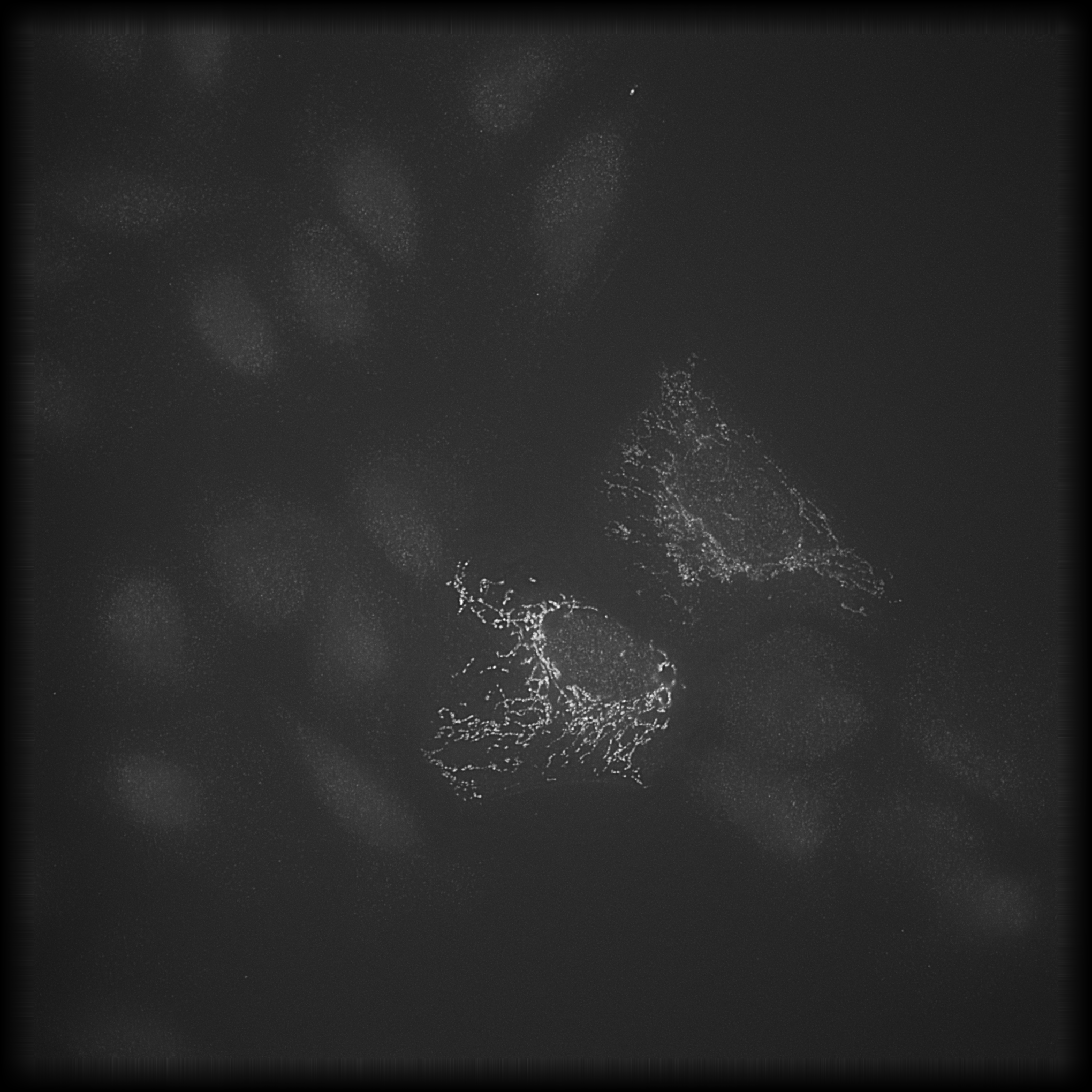

Supplement: Supplementary file 5 — Source data Fig. 3 [file 44319_2024_181_MOESM5_ESM.zip › Figure 3/Figure 3D/D290N_NIX.tif]

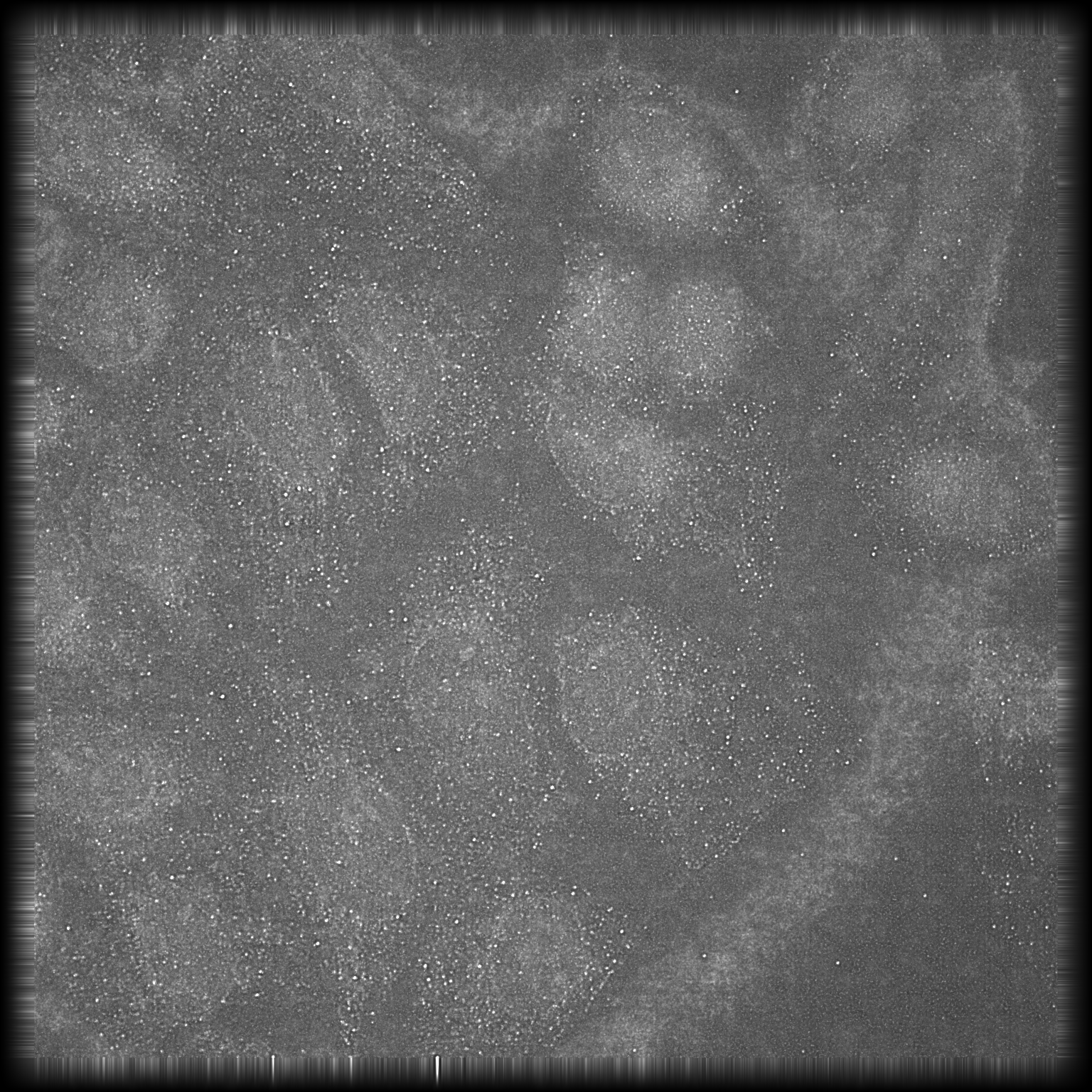

Supplement: Supplementary file 5 — Source data Fig. 3 [file 44319_2024_181_MOESM5_ESM.zip › Figure 3/Figure 3D/PPTC7KO_BNIP3.tif]

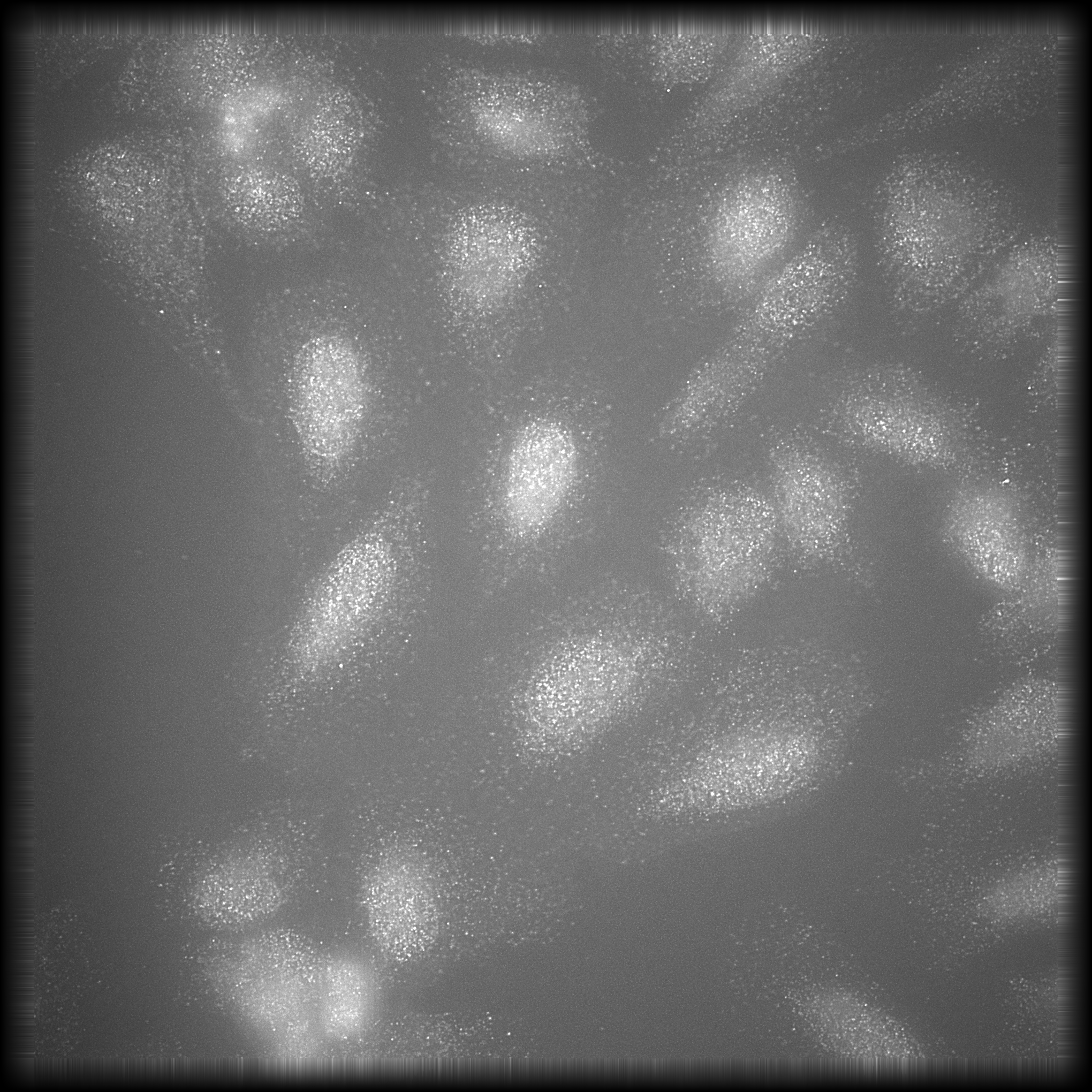

Supplement: Supplementary file 5 — Source data Fig. 3 [file 44319_2024_181_MOESM5_ESM.zip › Figure 3/Figure 3D/PPTC7KO_NIX.tif]

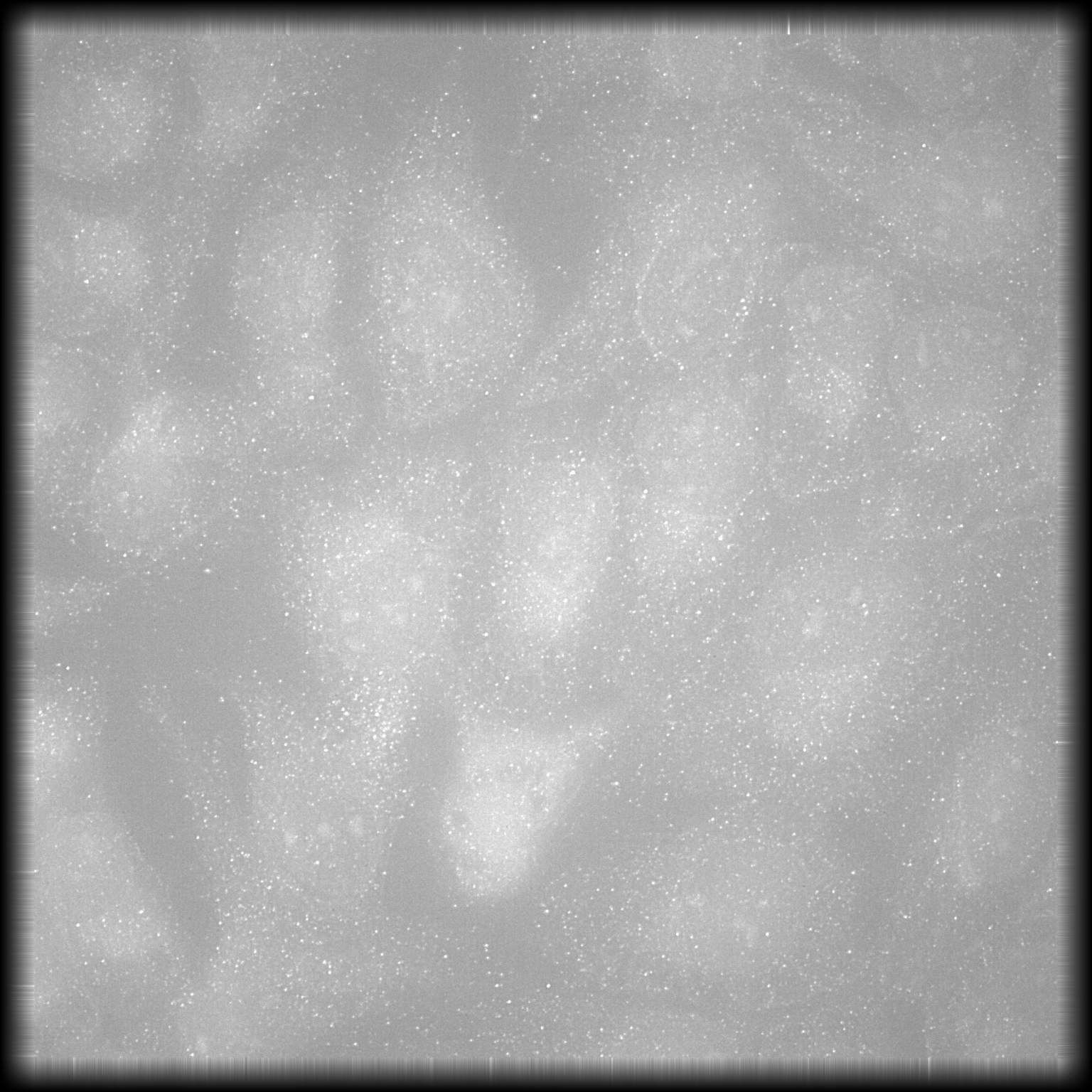

Supplement: Supplementary file 5 — Source data Fig. 3 [file 44319_2024_181_MOESM5_ESM.zip › Figure 3/Figure 3D/U2OS_BNIP3.tif]

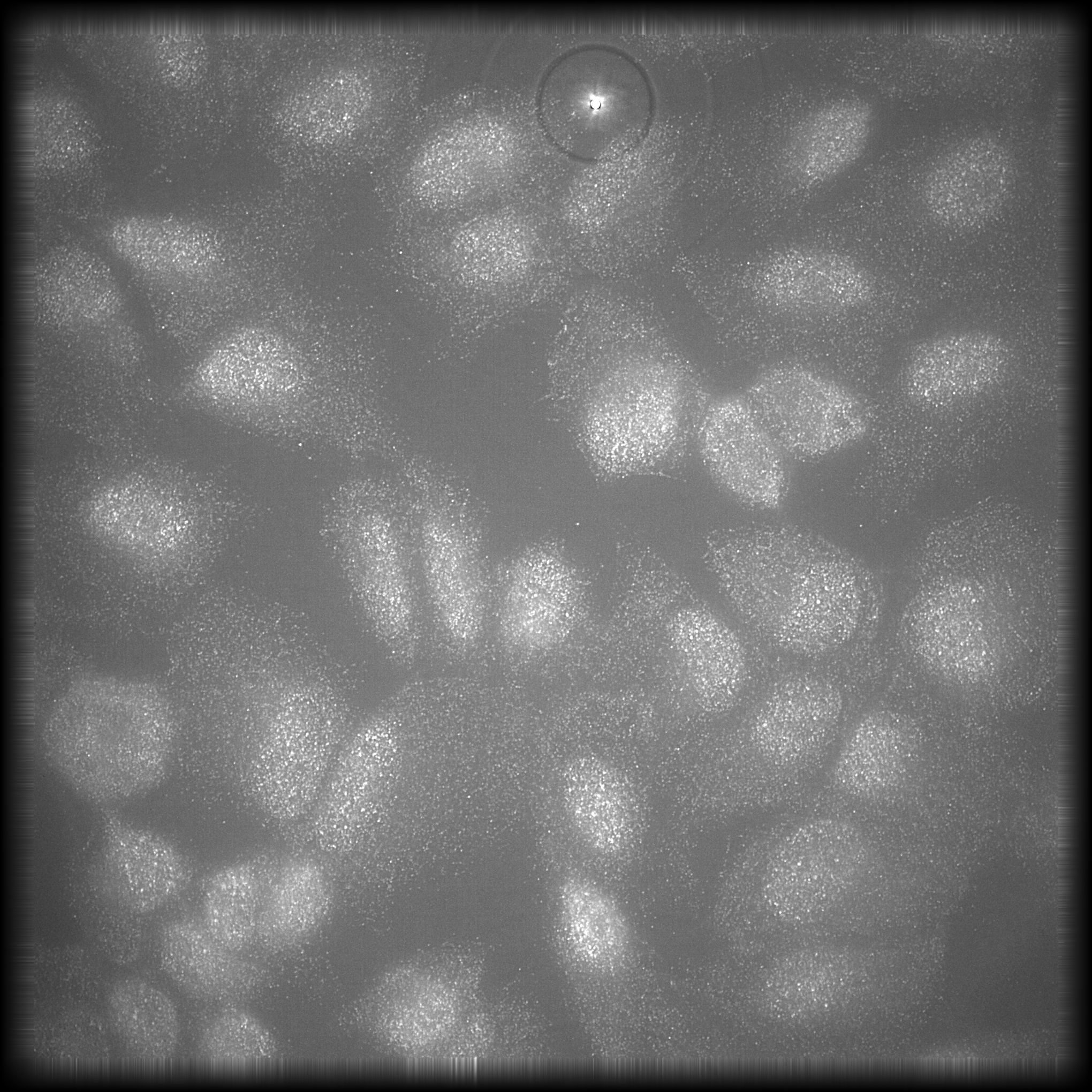

Supplement: Supplementary file 5 — Source data Fig. 3 [file 44319_2024_181_MOESM5_ESM.zip › Figure 3/Figure 3D/U2OS_NIX.tif]

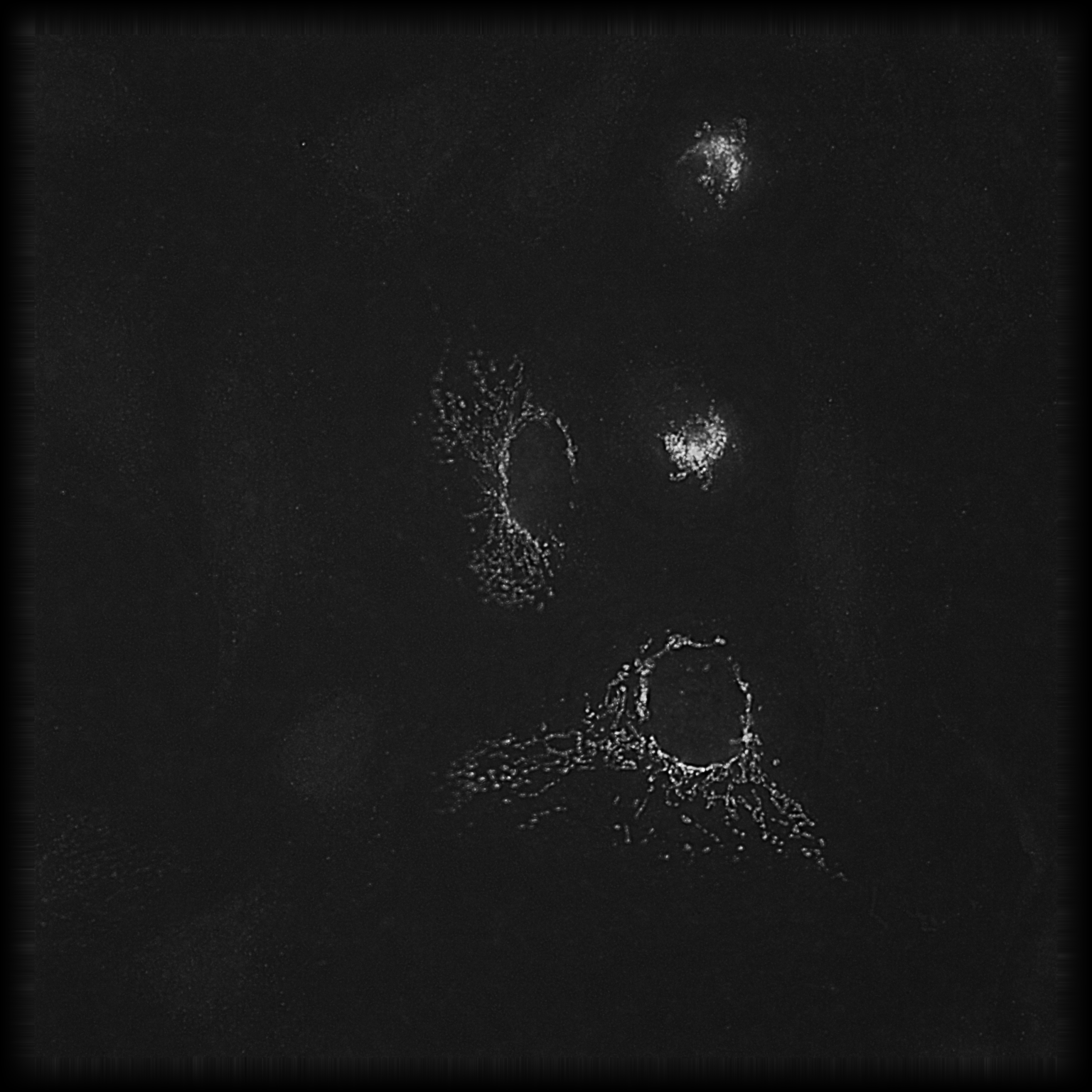

Supplement: Supplementary file 5 — Source data Fig. 3 [file 44319_2024_181_MOESM5_ESM.zip › Figure 3/Figure 3D/WT_BNIP3.tif]

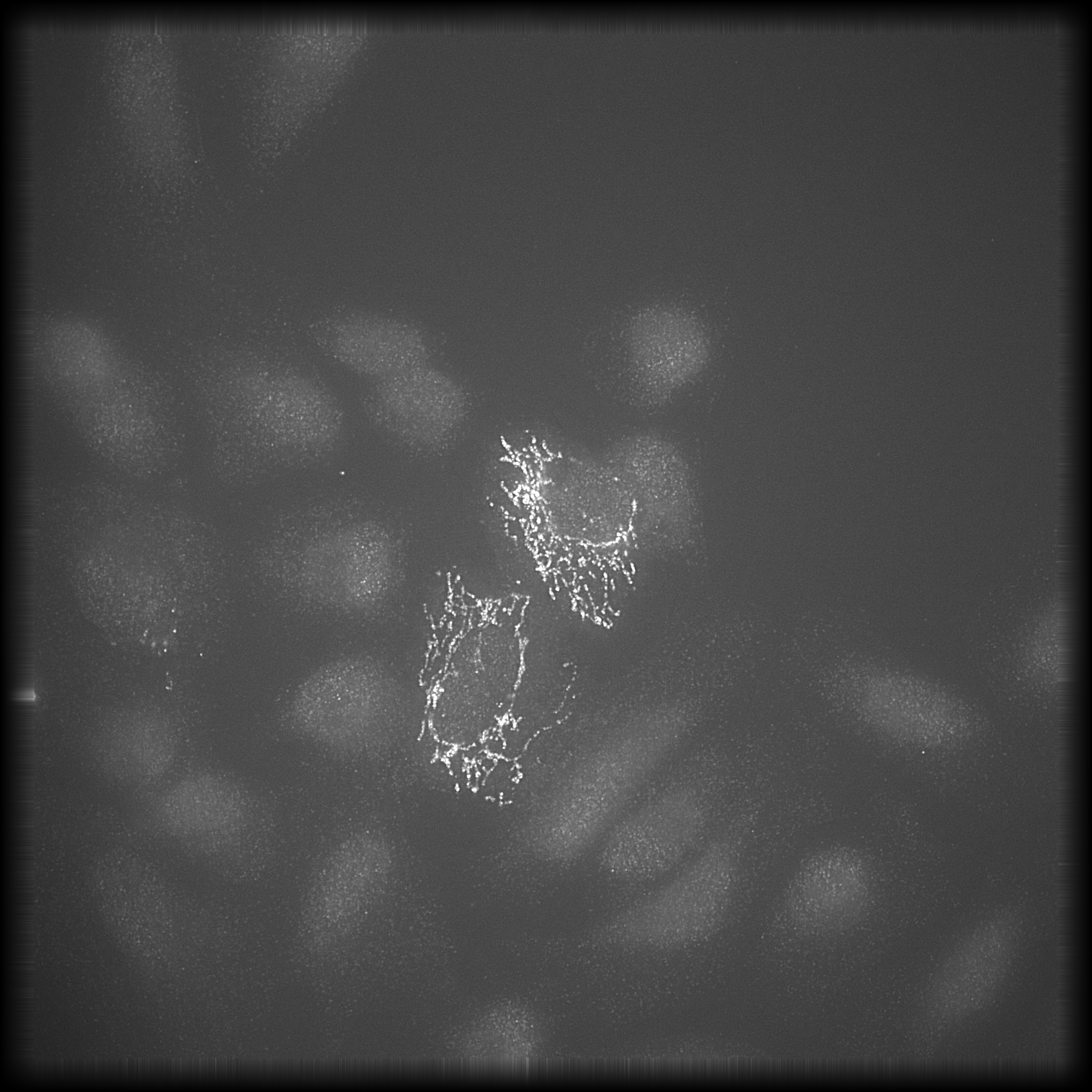

Supplement: Supplementary file 5 — Source data Fig. 3 [file 44319_2024_181_MOESM5_ESM.zip › Figure 3/Figure 3D/WT_NIX.tif]

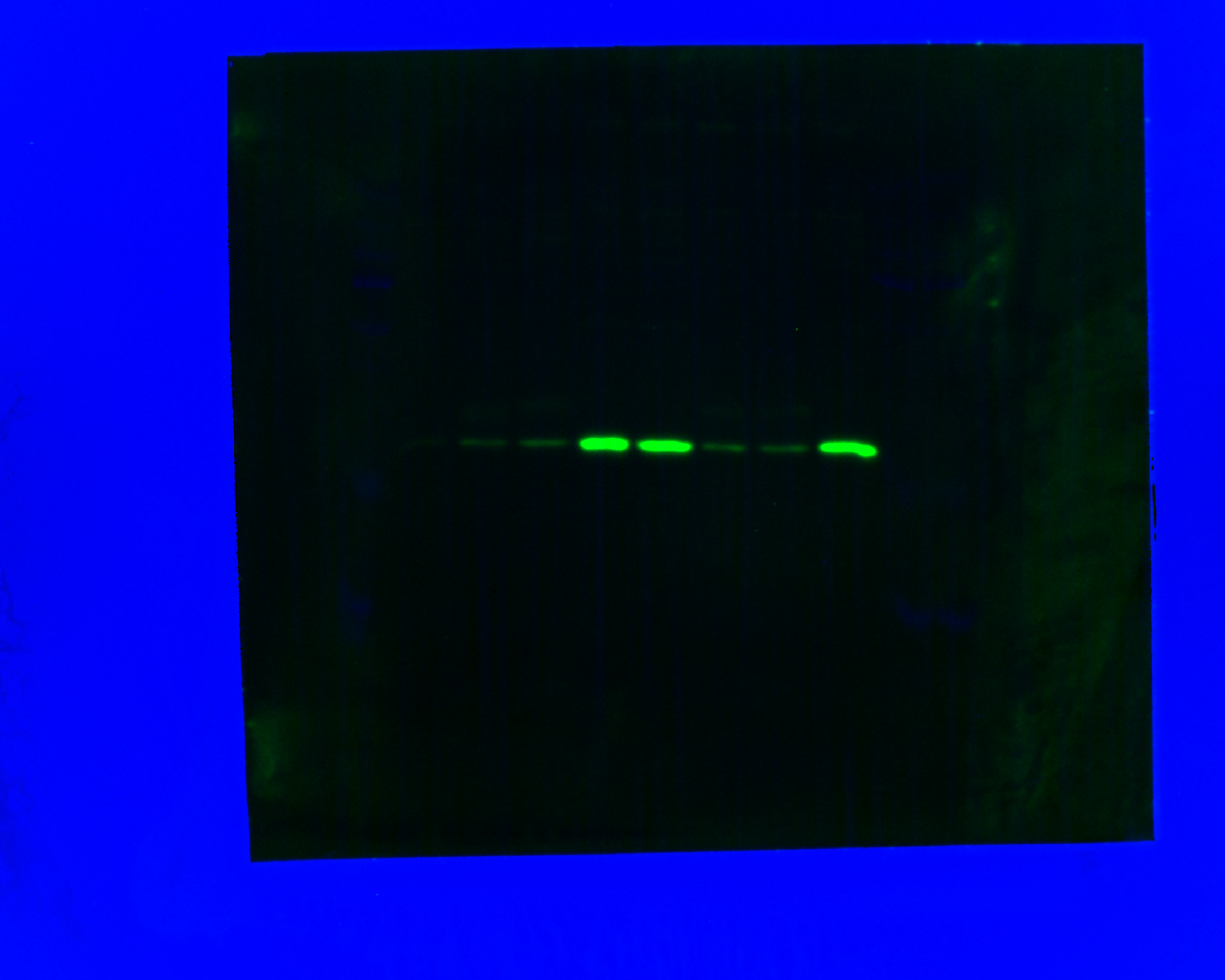

Supplement: Supplementary file 5 — Source data Fig. 3 [file 44319_2024_181_MOESM5_ESM.zip › Figure 3/Figure 3E/(BNIP3) (Composite).tif]

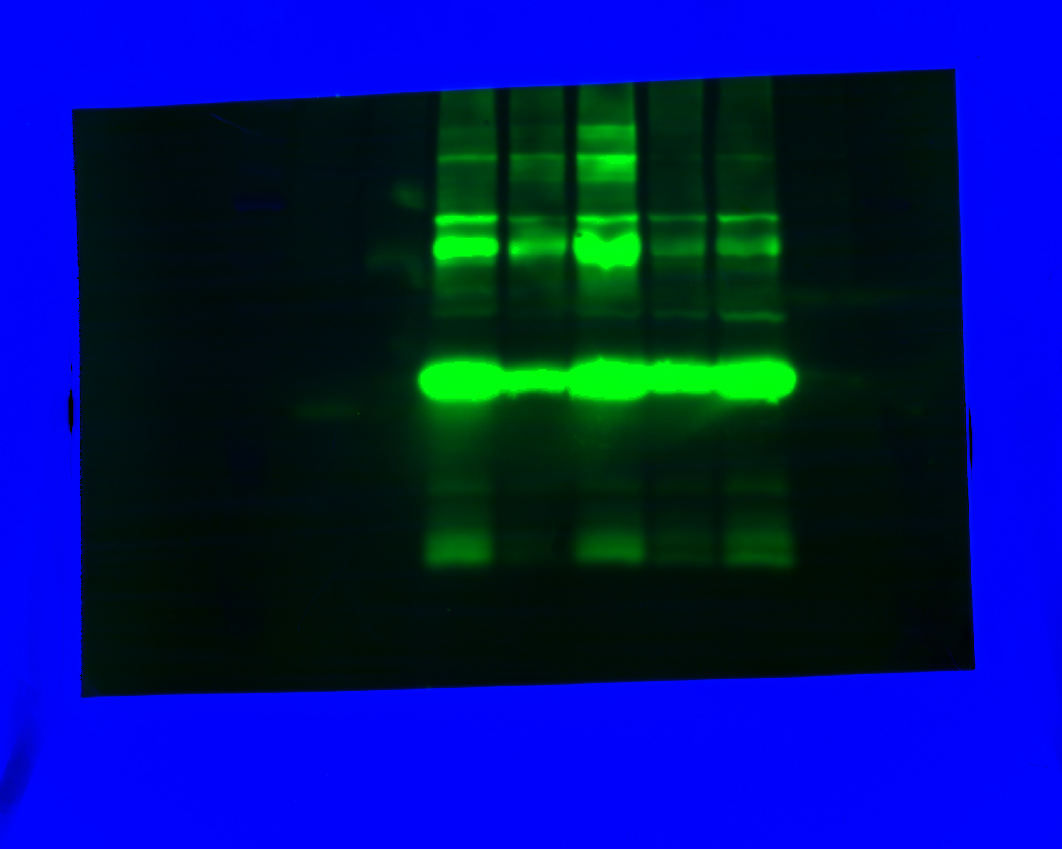

Supplement: Supplementary file 5 — Source data Fig. 3 [file 44319_2024_181_MOESM5_ESM.zip › Figure 3/Figure 3E/(PPTC7) (Composite).tif]

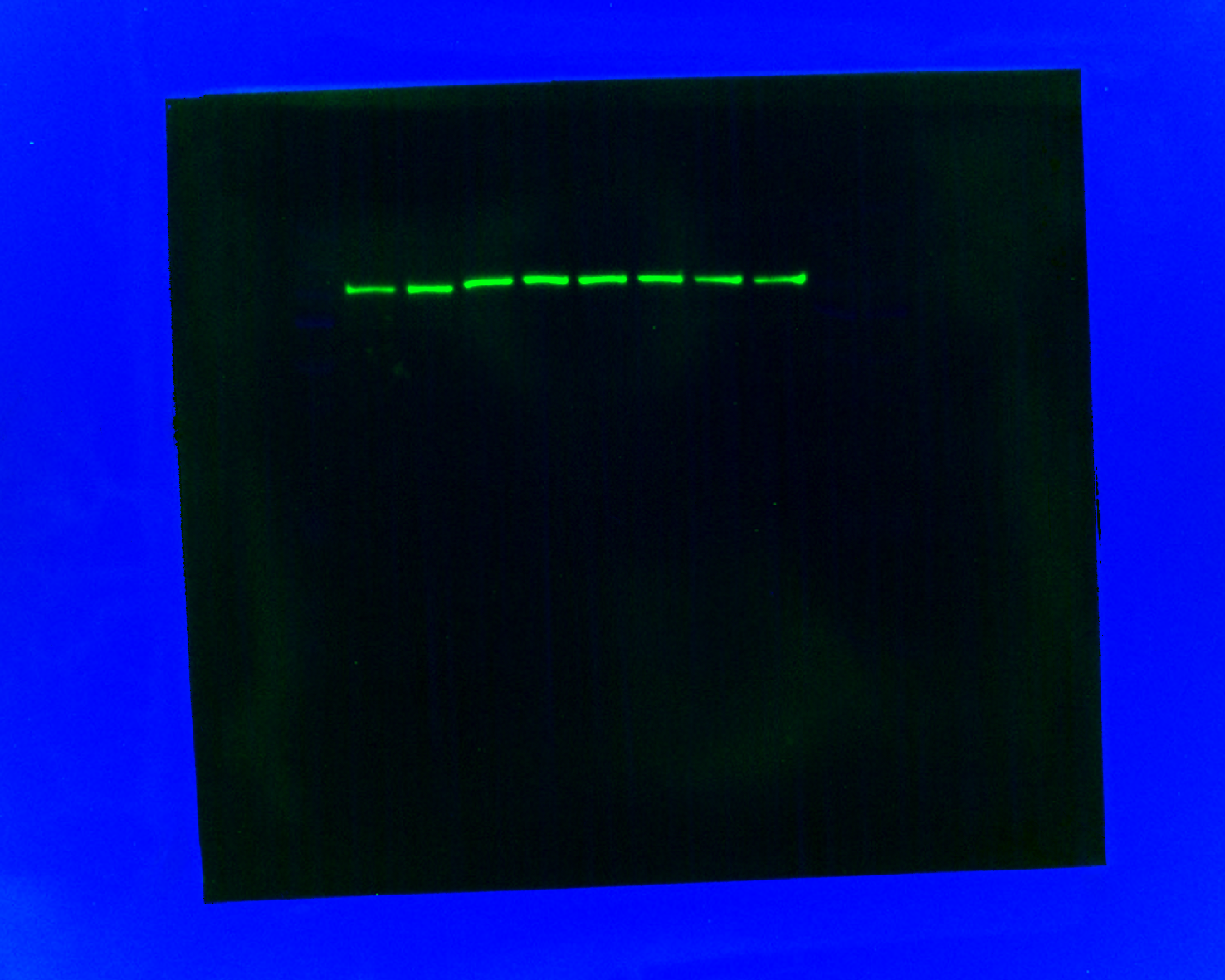

Supplement: Supplementary file 5 — Source data Fig. 3 [file 44319_2024_181_MOESM5_ESM.zip › Figure 3/Figure 3E/(VCL)(Composite).tif]

PHOSTAG GEL

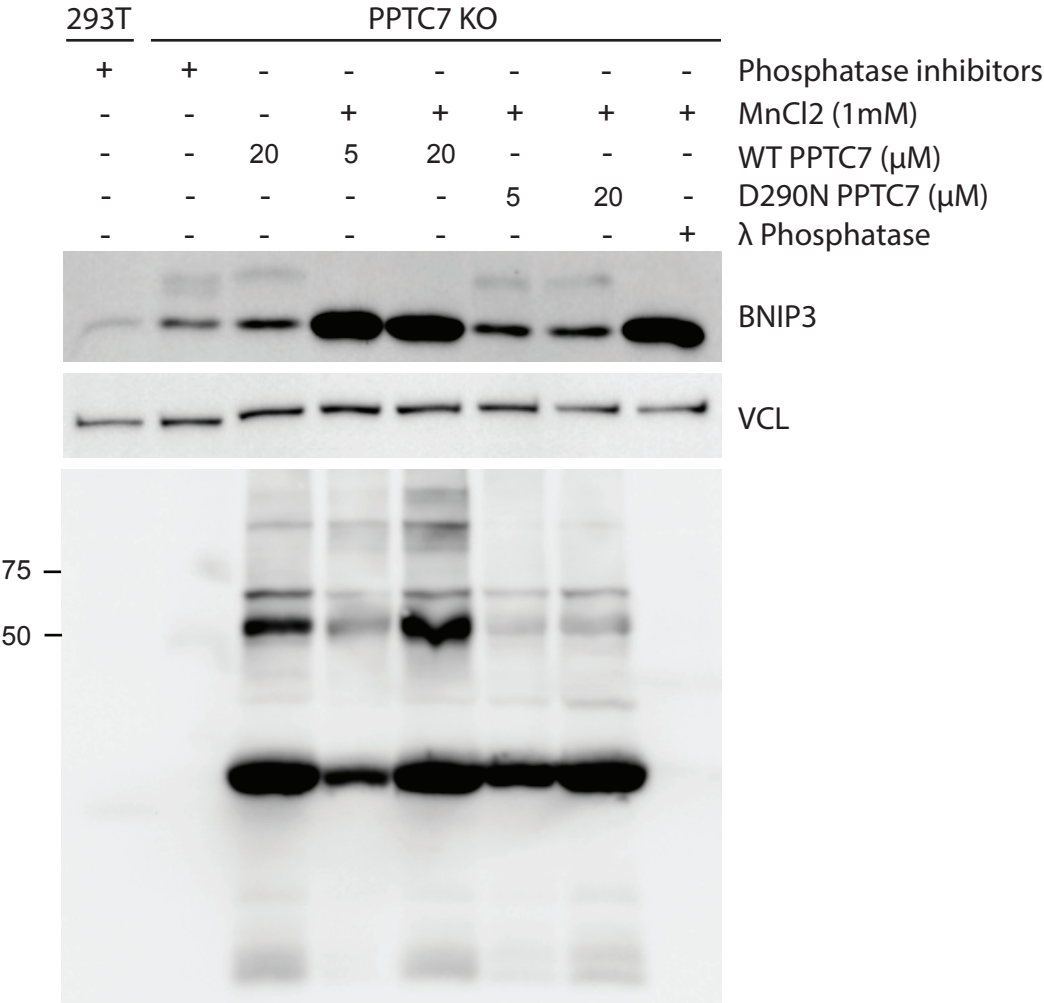

Supplement: Supplementary file 5 — Source data Fig. 3 [file 44319_2024_181_MOESM5_ESM.zip › Figure 3/Figure 3E/Annotations Figure 3E.pdf]

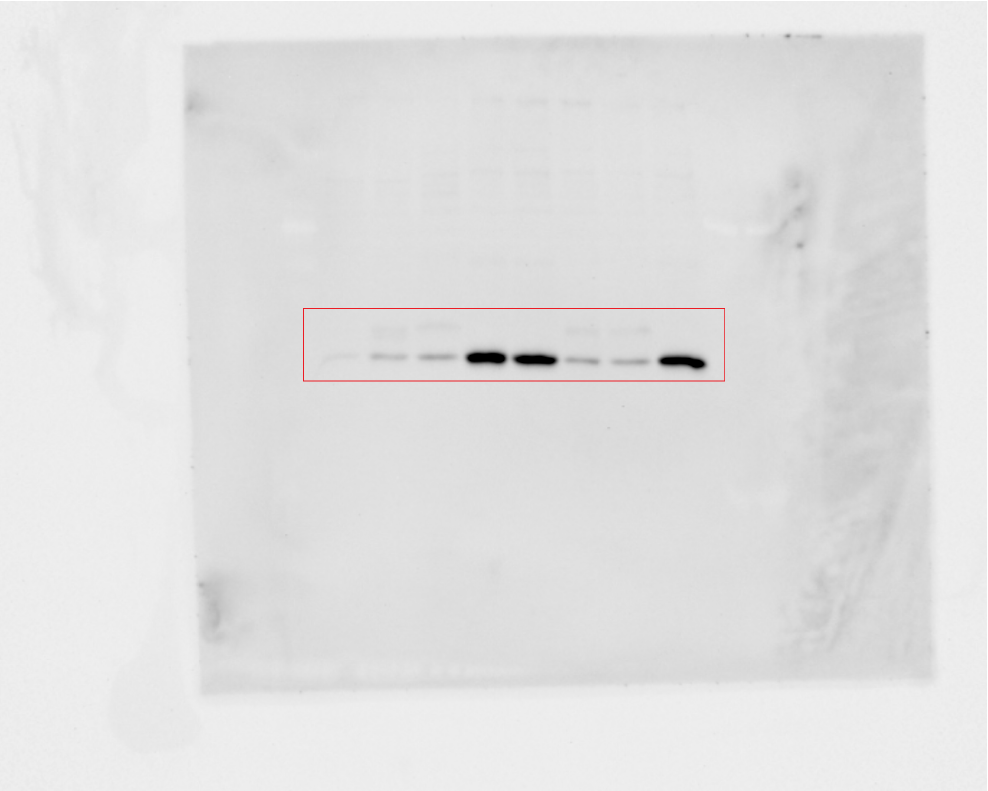

Supplement: Supplementary file 5 — Source data Fig. 3 [file 44319_2024_181_MOESM5_ESM.zip › Figure 3/Figure 3E/BNIP3.png]

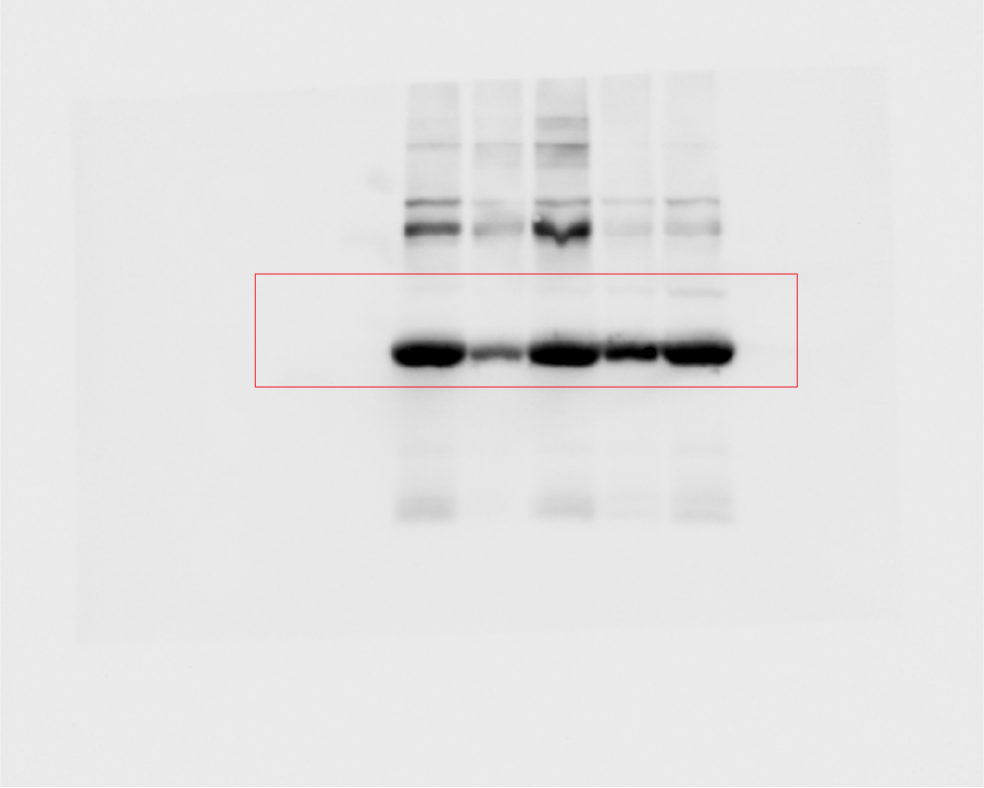

Supplement: Supplementary file 5 — Source data Fig. 3 [file 44319_2024_181_MOESM5_ESM.zip › Figure 3/Figure 3E/PPTC7.png]

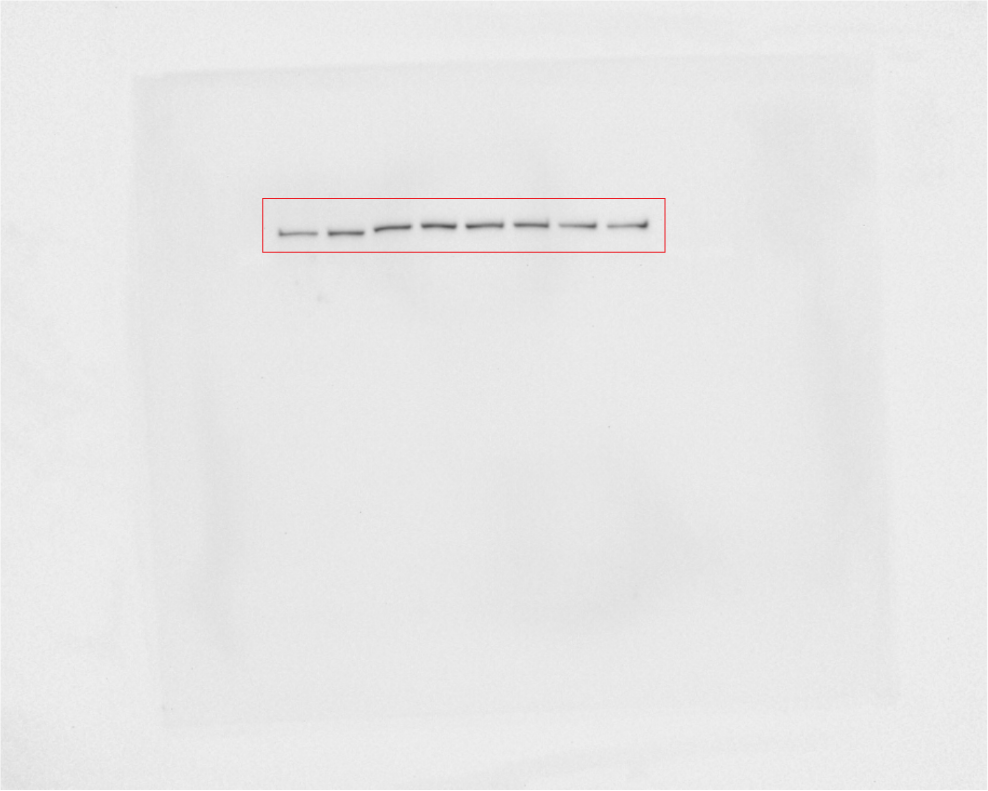

Supplement: Supplementary file 5 — Source data Fig. 3 [file 44319_2024_181_MOESM5_ESM.zip › Figure 3/Figure 3E/Vinculin.png]

## Immunoprecipitations

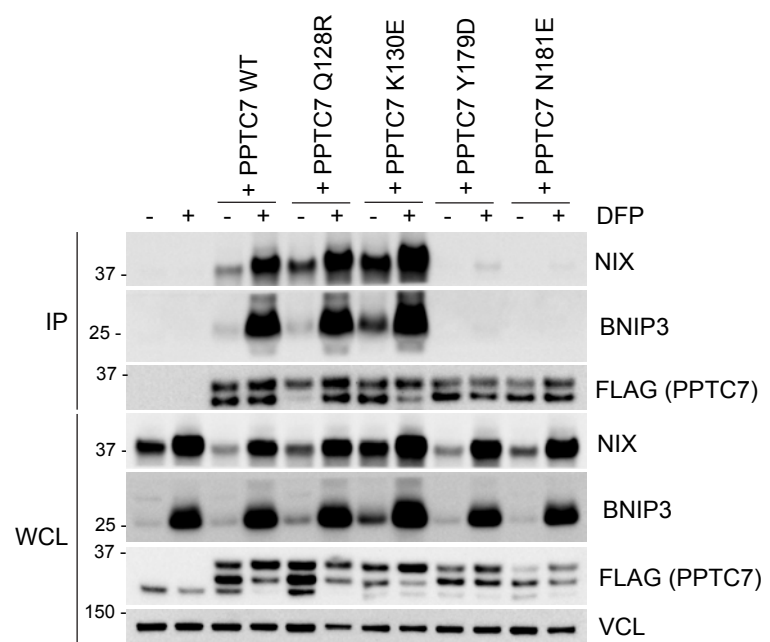

Supplement: Supplementary file 6 — Source data Fig. 4 [file 44319_2024_181_MOESM6_ESM.zip › Figure 4/Figure 4B/Annotation Figure 4B.pdf]

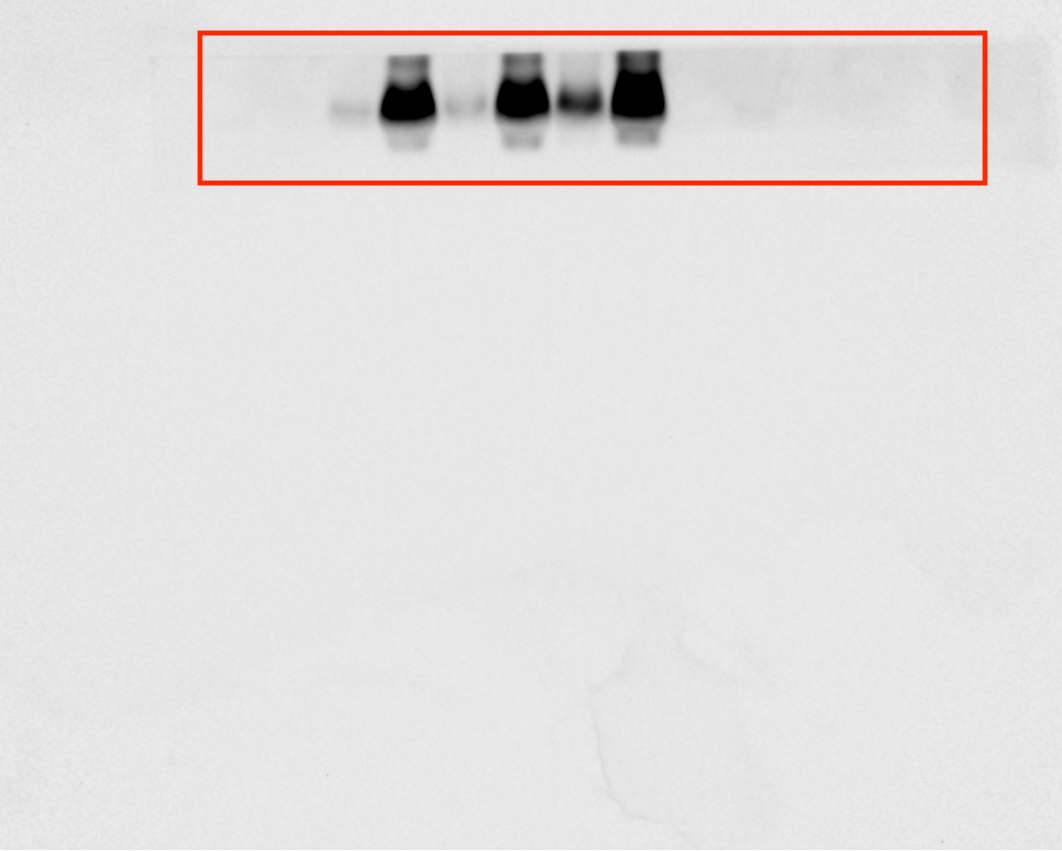

Supplement: Supplementary file 6 — Source data Fig. 4 [file 44319_2024_181_MOESM6_ESM.zip › Figure 4/Figure 4B/ip bnip3.tif]

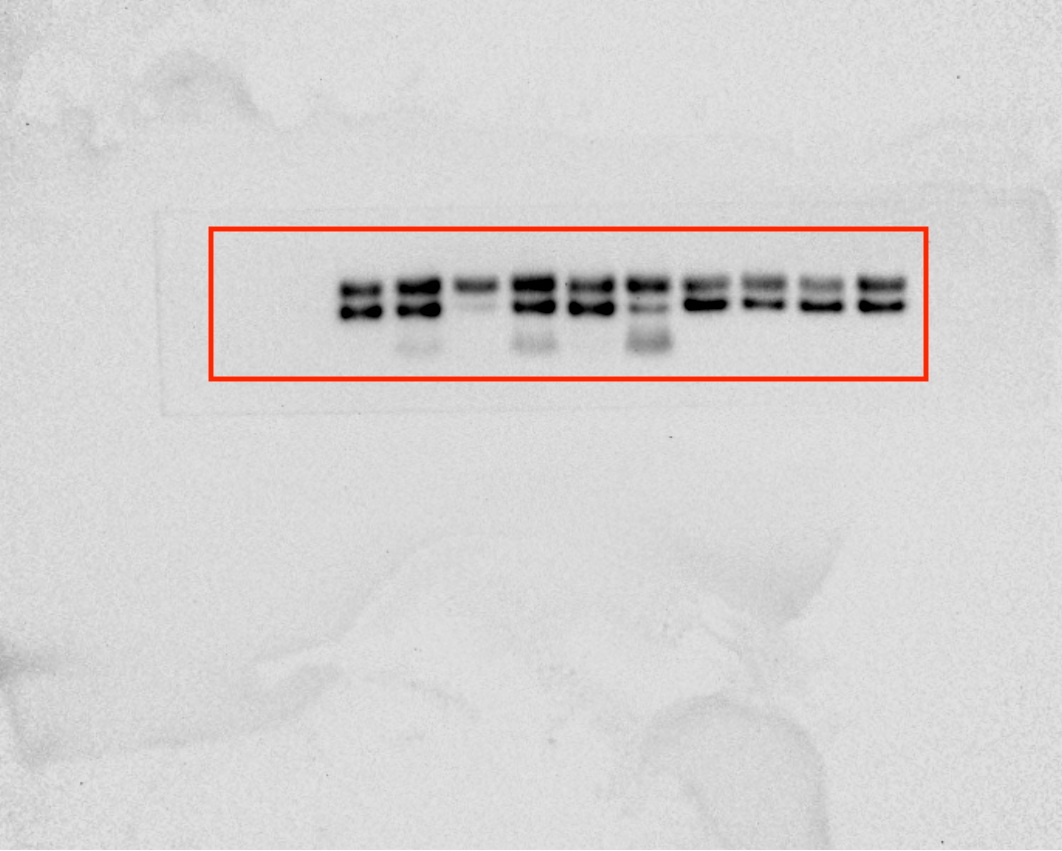

Supplement: Supplementary file 6 — Source data Fig. 4 [file 44319_2024_181_MOESM6_ESM.zip › Figure 4/Figure 4B/ip flag.tif]

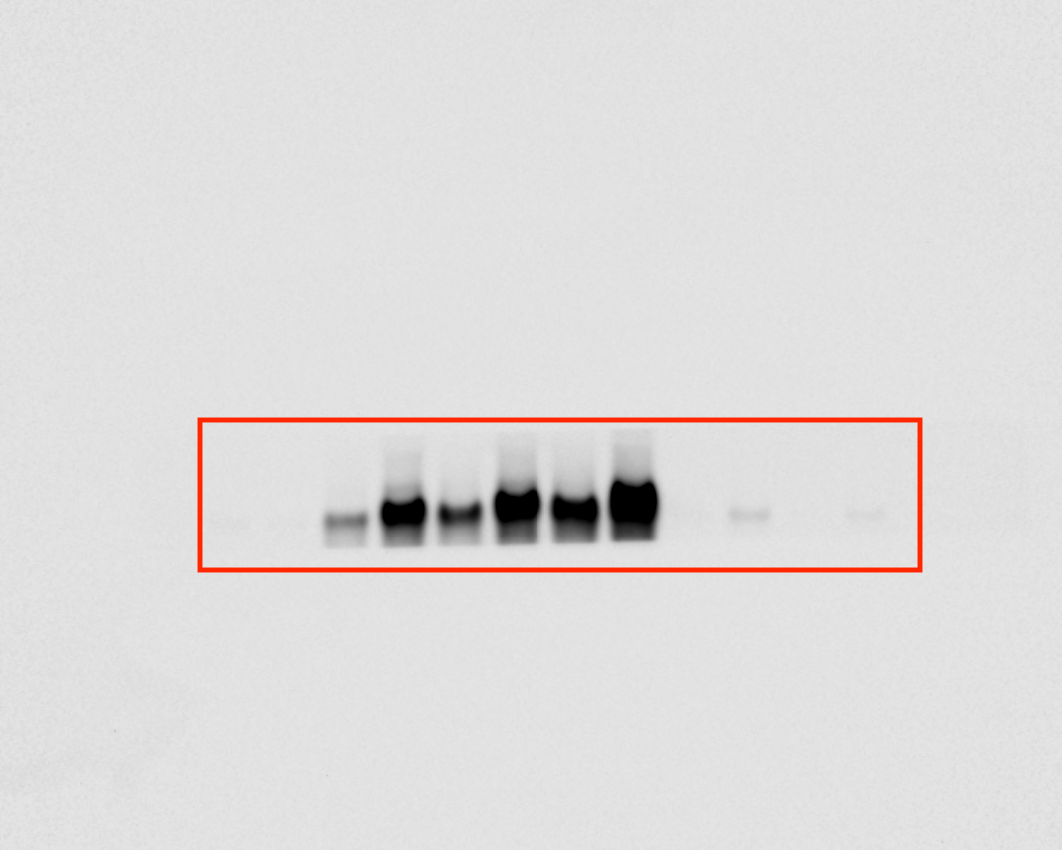

Supplement: Supplementary file 6 — Source data Fig. 4 [file 44319_2024_181_MOESM6_ESM.zip › Figure 4/Figure 4B/ip nix.tif]

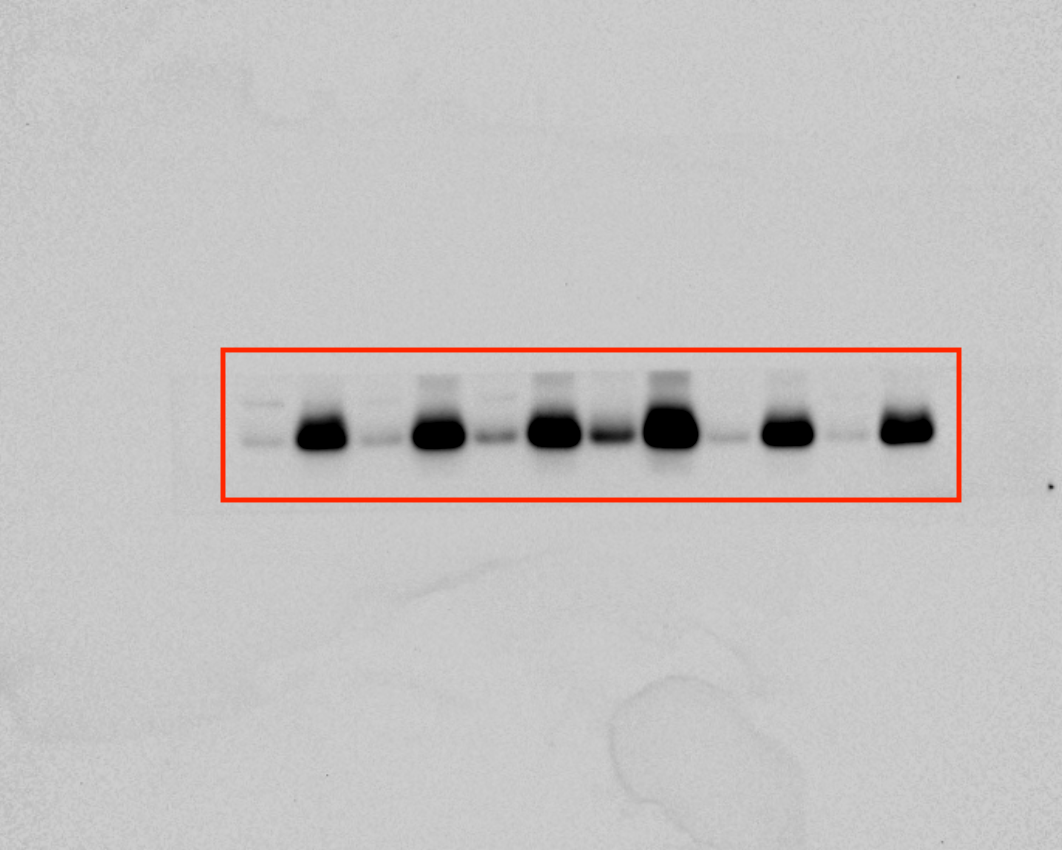

Supplement: Supplementary file 6 — Source data Fig. 4 [file 44319_2024_181_MOESM6_ESM.zip › Figure 4/Figure 4B/wcl bnip3.tif]

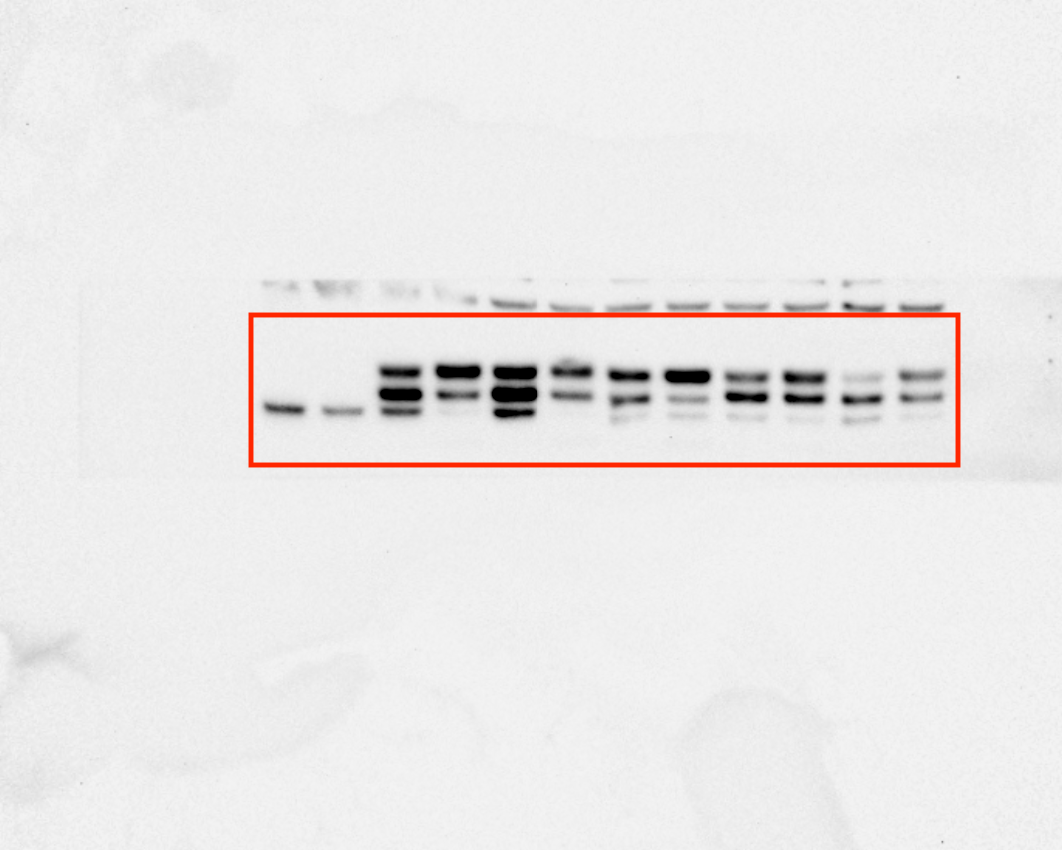

Supplement: Supplementary file 6 — Source data Fig. 4 [file 44319_2024_181_MOESM6_ESM.zip › Figure 4/Figure 4B/wcl flag.tif]

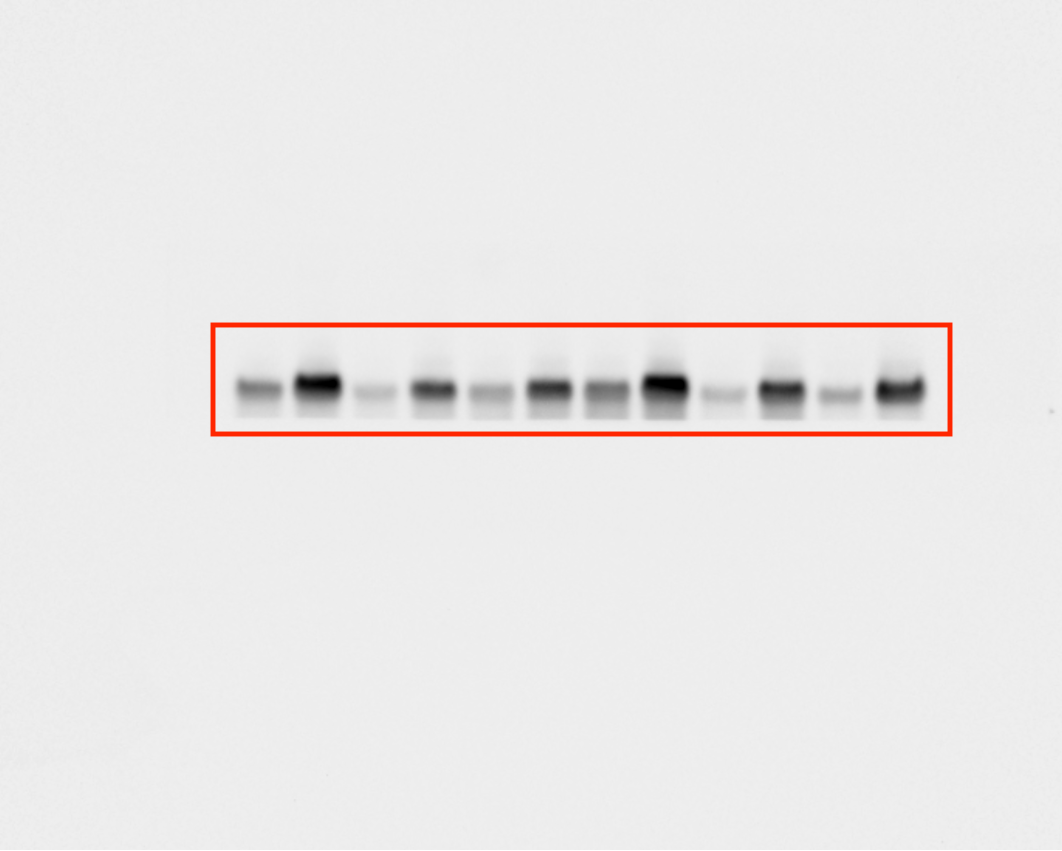

Supplement: Supplementary file 6 — Source data Fig. 4 [file 44319_2024_181_MOESM6_ESM.zip › Figure 4/Figure 4B/wcl nix.tif]

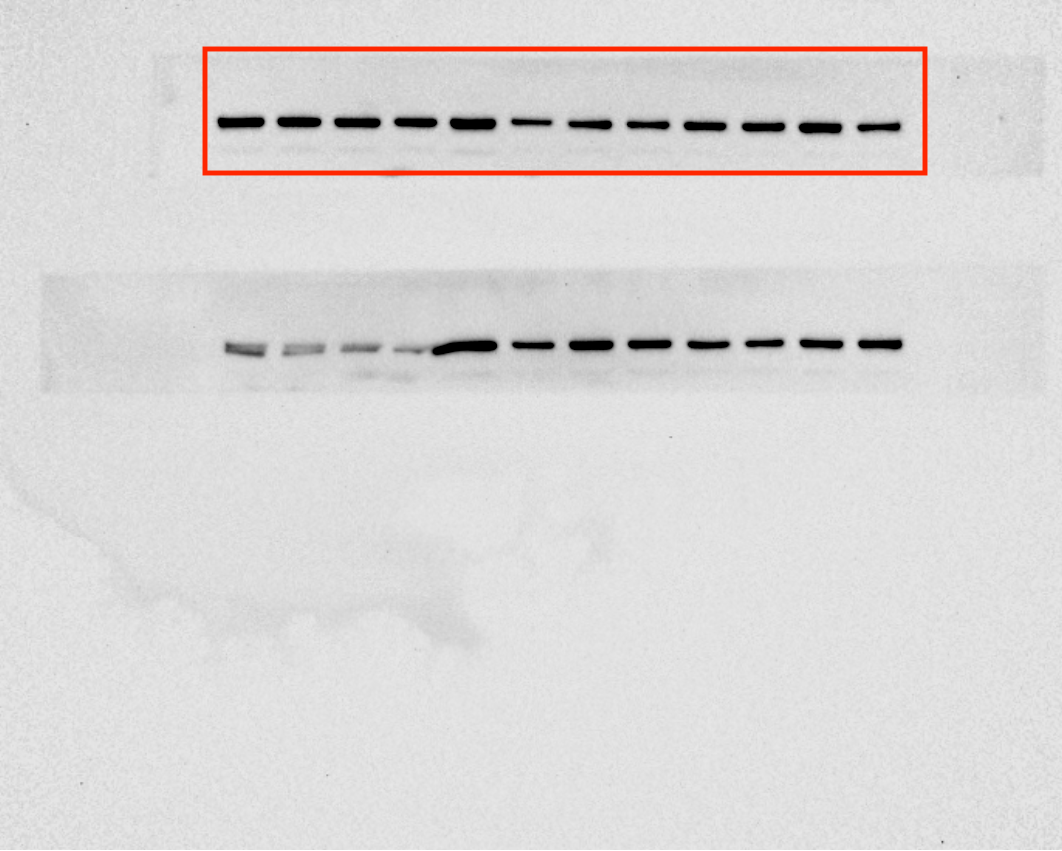

Supplement: Supplementary file 6 — Source data Fig. 4 [file 44319_2024_181_MOESM6_ESM.zip › Figure 4/Figure 4B/wcl vcl.tif]

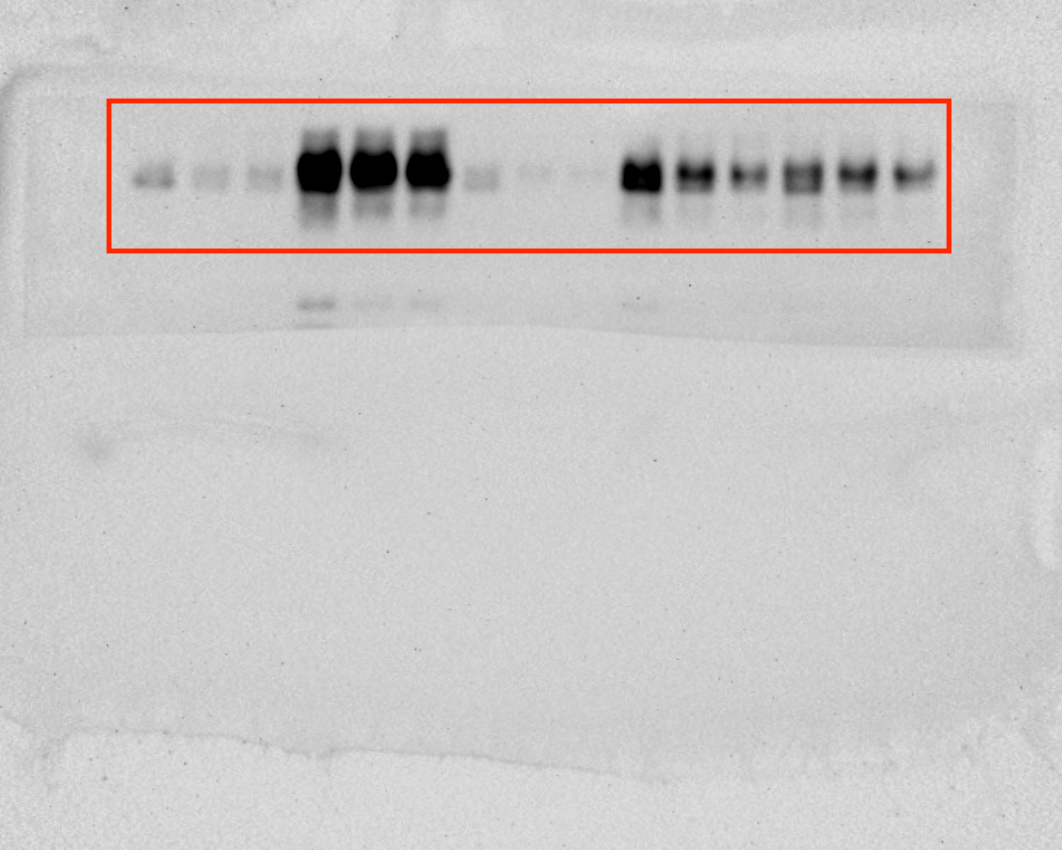

Supplement: Supplementary file 6 — Source data Fig. 4 [file 44319_2024_181_MOESM6_ESM.zip › Figure 4/Figure 4C/BNIP3.tif]

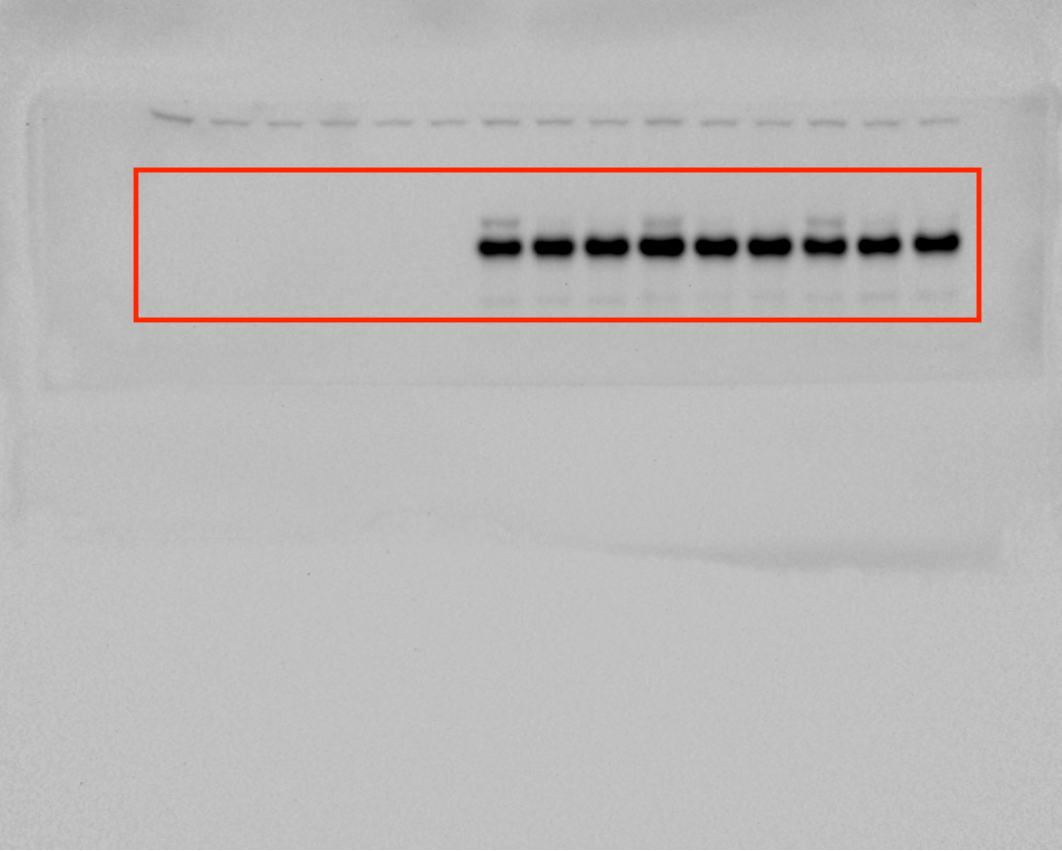

Supplement: Supplementary file 6 — Source data Fig. 4 [file 44319_2024_181_MOESM6_ESM.zip › Figure 4/Figure 4C/HA-PPTC7.tif]

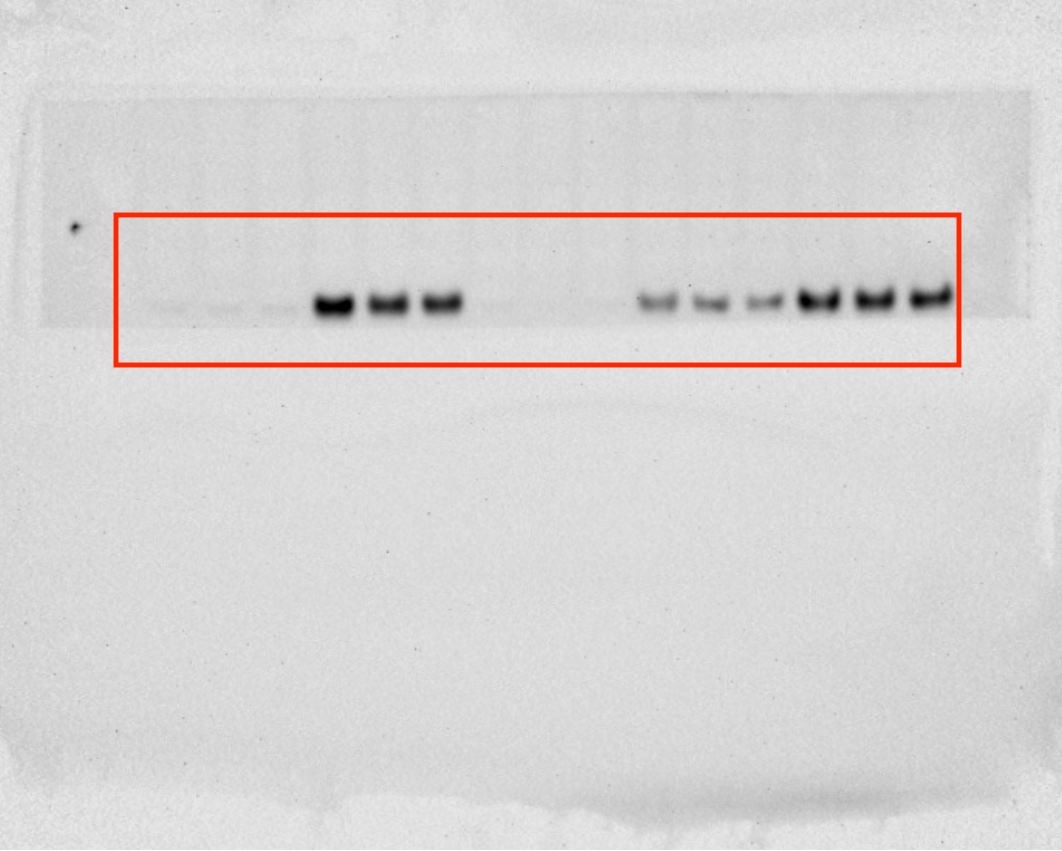

Supplement: Supplementary file 6 — Source data Fig. 4 [file 44319_2024_181_MOESM6_ESM.zip › Figure 4/Figure 4C/NIX.tif]

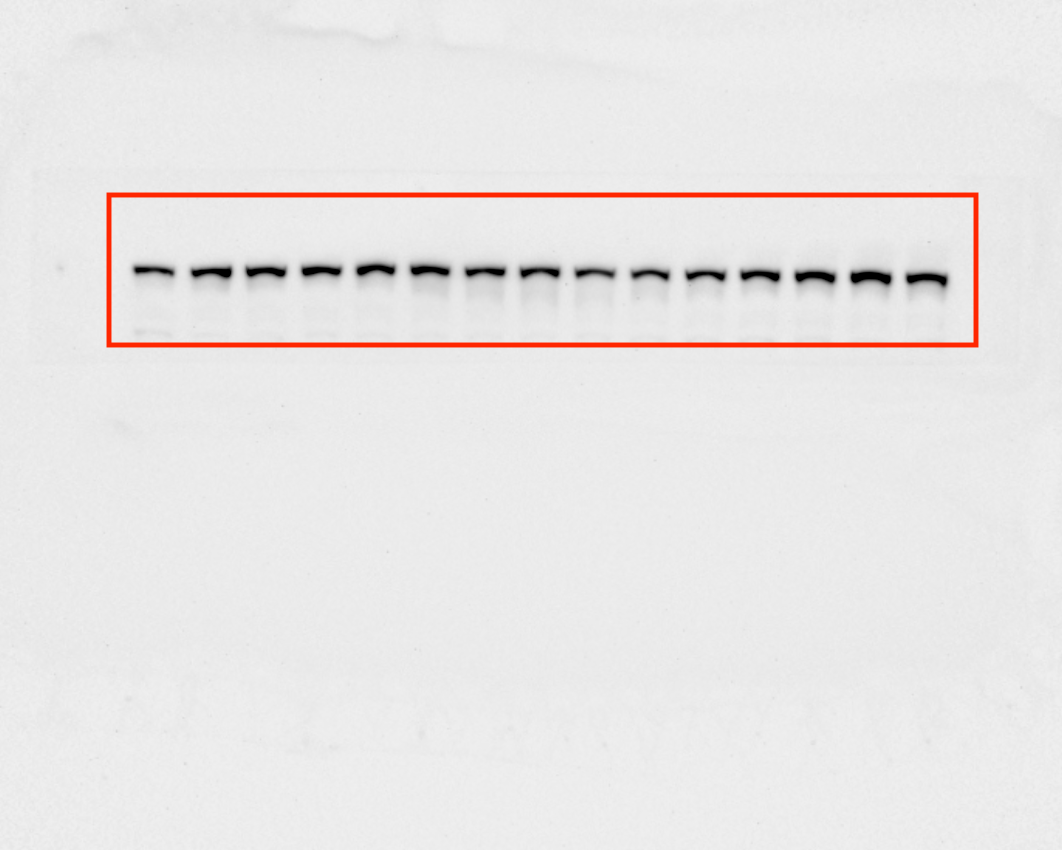

Supplement: Supplementary file 6 — Source data Fig. 4 [file 44319_2024_181_MOESM6_ESM.zip › Figure 4/Figure 4C/VCL.tif]

FLAG-IP

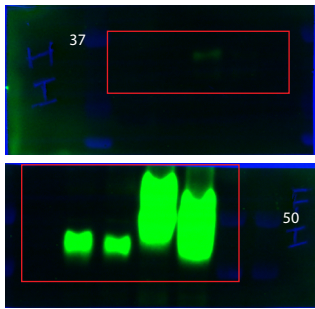

WCL

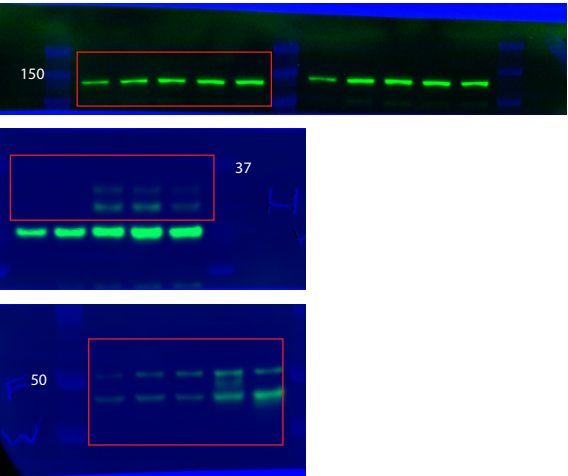

HeLa Flp-in BNIP3/NIX KO

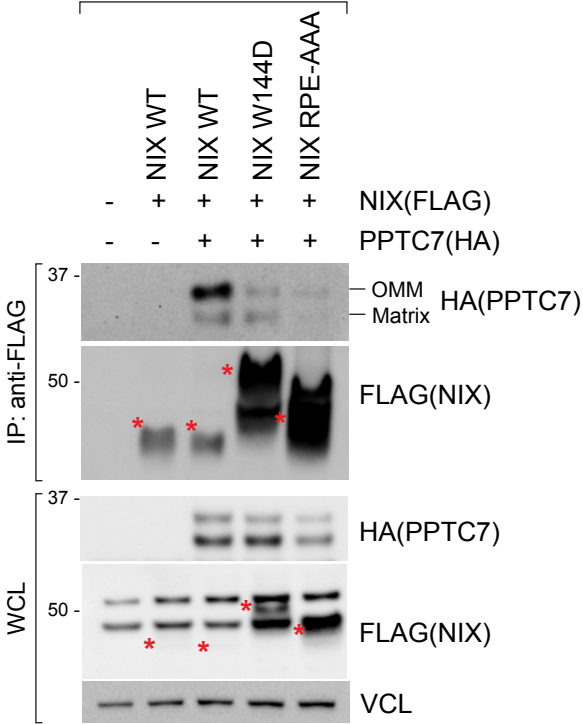

Supplement: Supplementary file 6 — Source data Fig. 4 [file 44319_2024_181_MOESM6_ESM.zip › Figure 4/Figure 4E/Annotation Figure 4E.pdf]

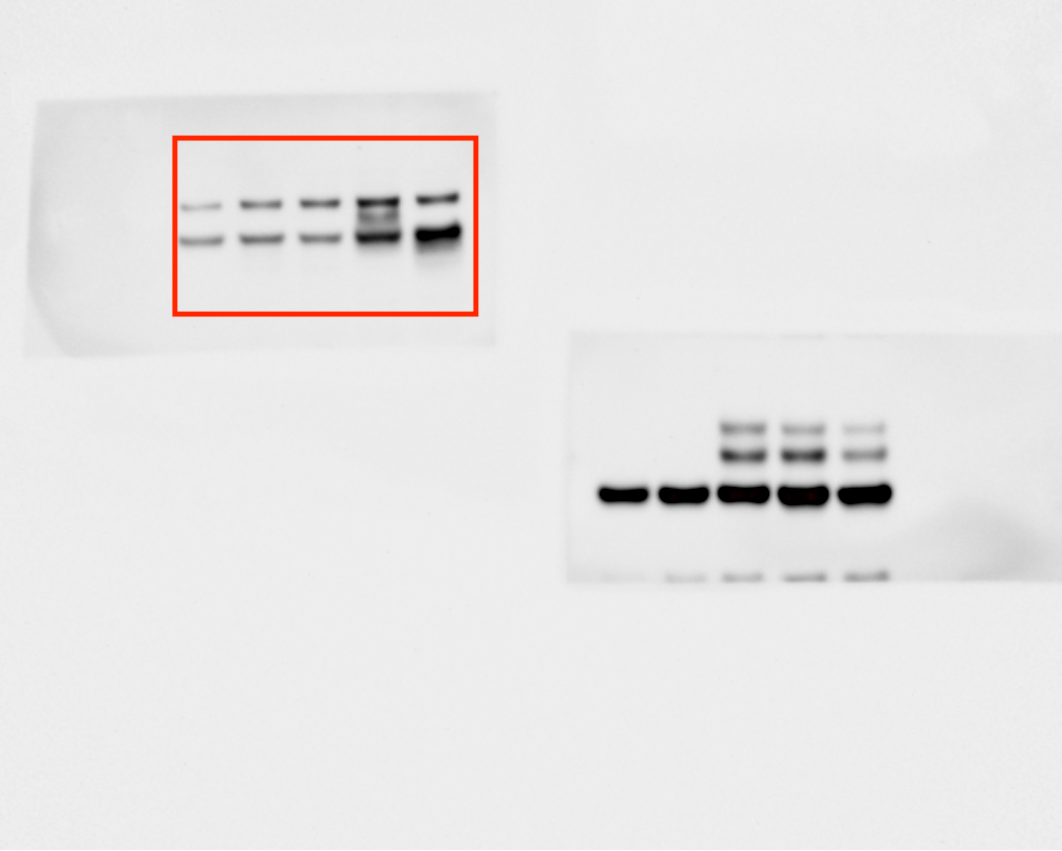

Supplement: Supplementary file 6 — Source data Fig. 4 [file 44319_2024_181_MOESM6_ESM.zip › Figure 4/Figure 4E/FLAG.tif]

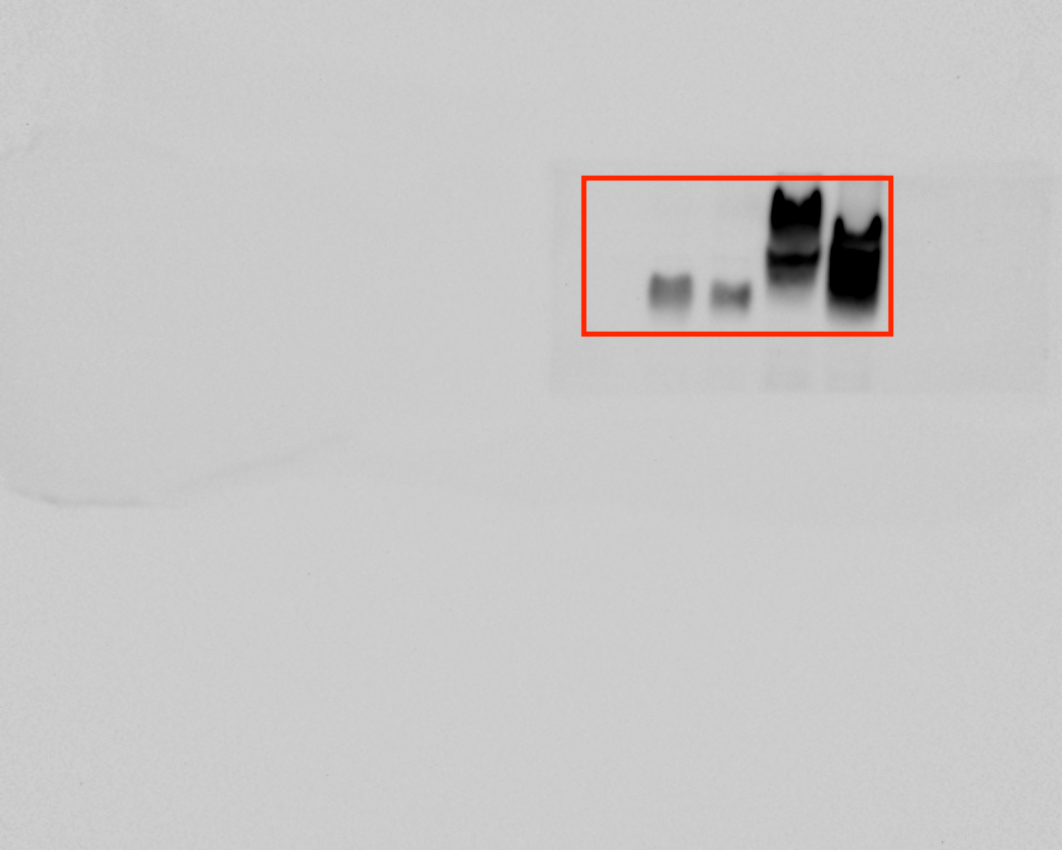

Supplement: Supplementary file 6 — Source data Fig. 4 [file 44319_2024_181_MOESM6_ESM.zip › Figure 4/Figure 4E/IP_FLAG.tif]

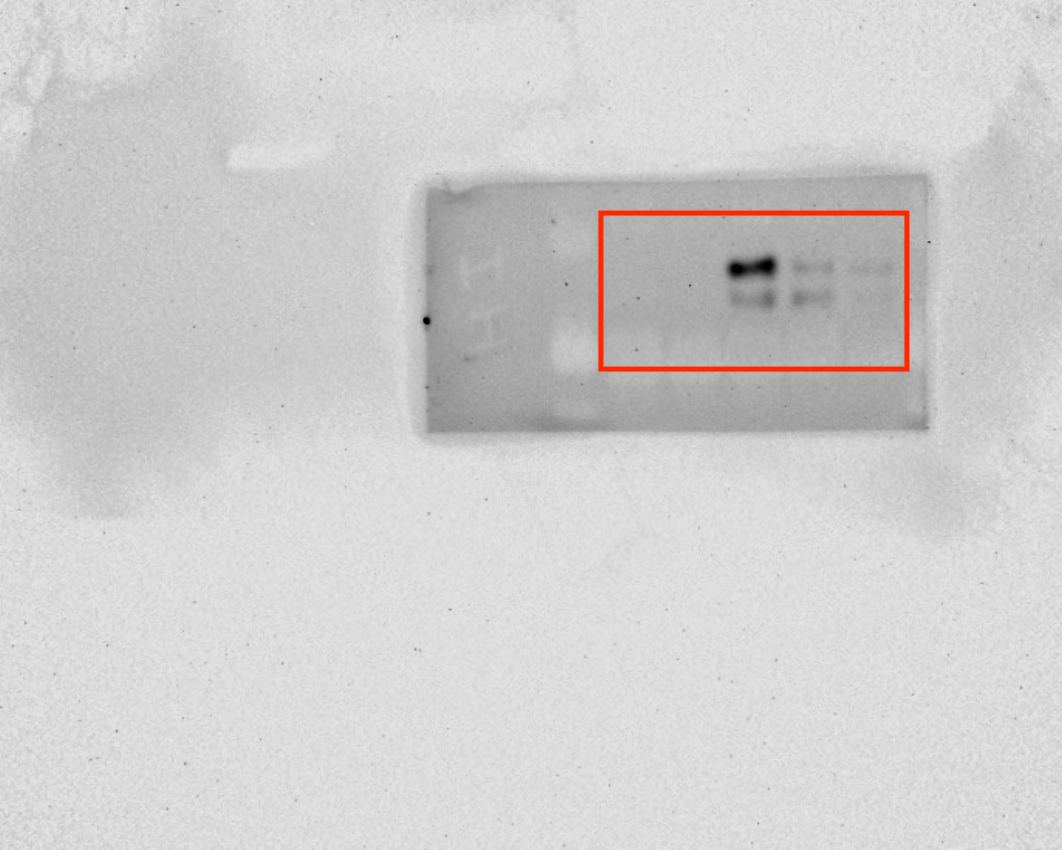

Supplement: Supplementary file 6 — Source data Fig. 4 [file 44319_2024_181_MOESM6_ESM.zip › Figure 4/Figure 4E/IP_HA.tif]

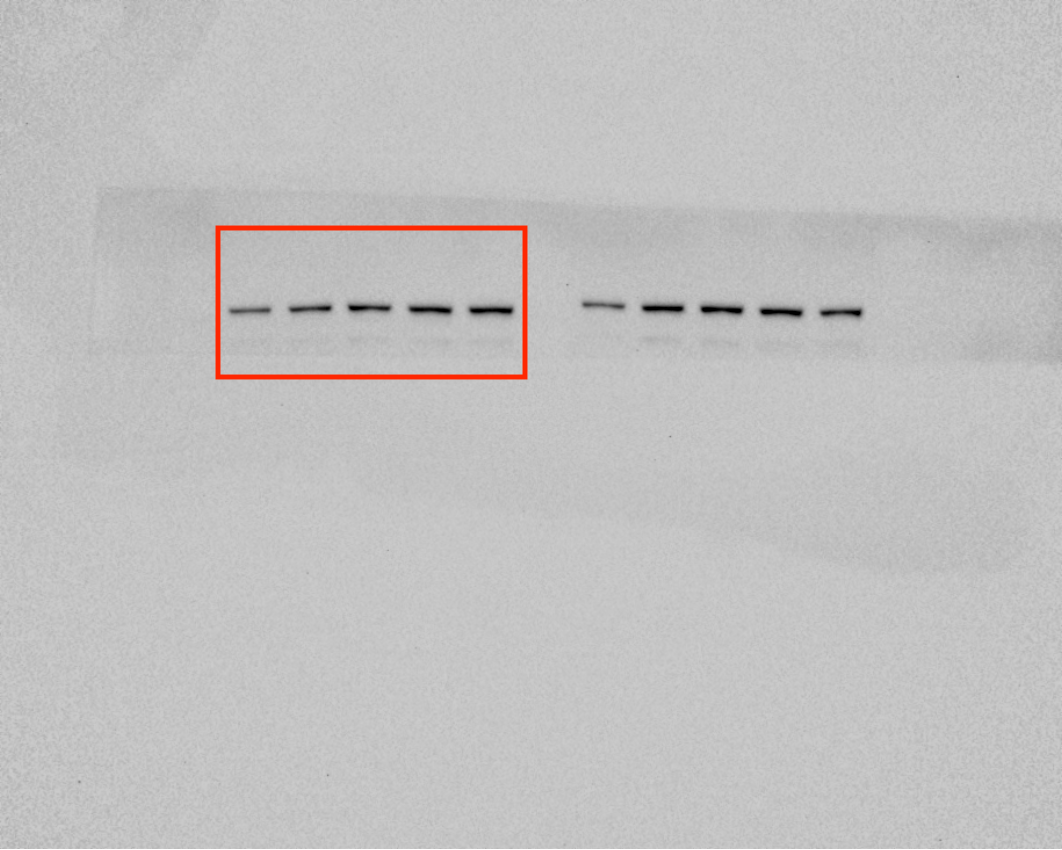

Supplement: Supplementary file 6 — Source data Fig. 4 [file 44319_2024_181_MOESM6_ESM.zip › Figure 4/Figure 4E/VCL.tif]

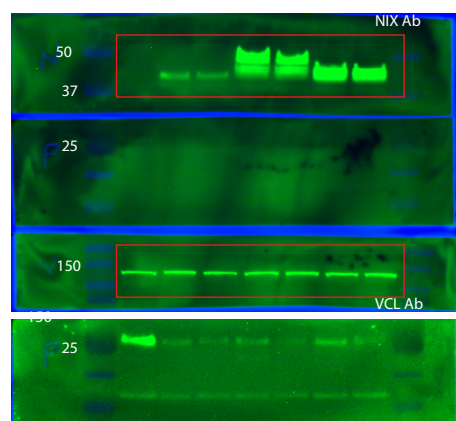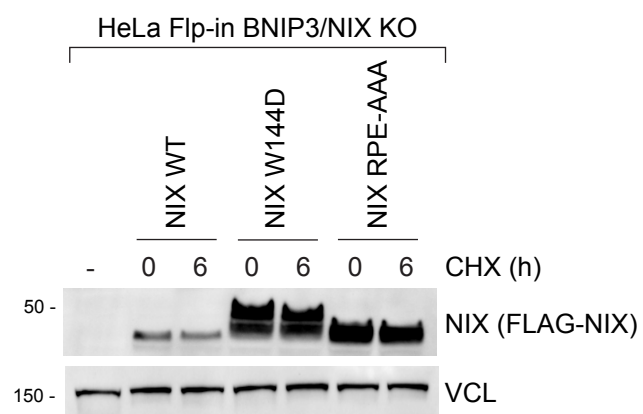

Supplement: Supplementary file 6 — Source data Fig. 4 [file 44319_2024_181_MOESM6_ESM.zip › Figure 4/Figure 4F/Annotation Figure 4F.pdf]

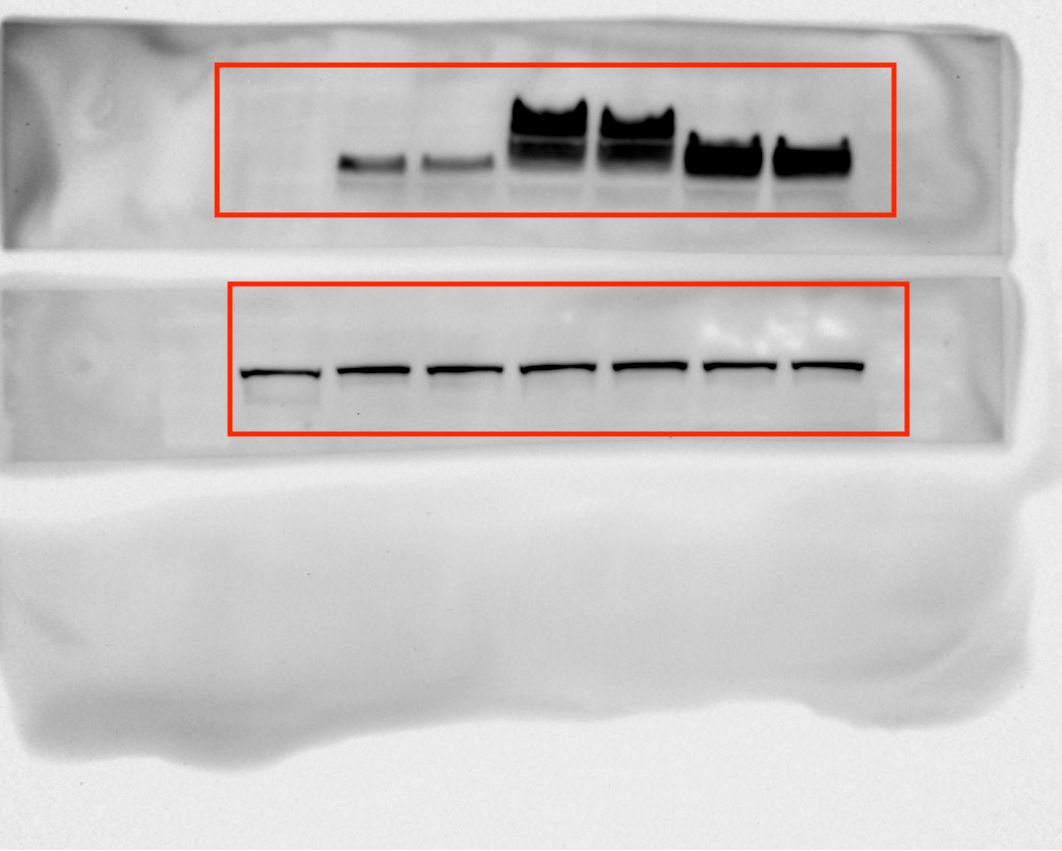

Supplement: Supplementary file 6 — Source data Fig. 4 [file 44319_2024_181_MOESM6_ESM.zip › Figure 4/Figure 4F/NIX-top_VCL-bottom.tif]

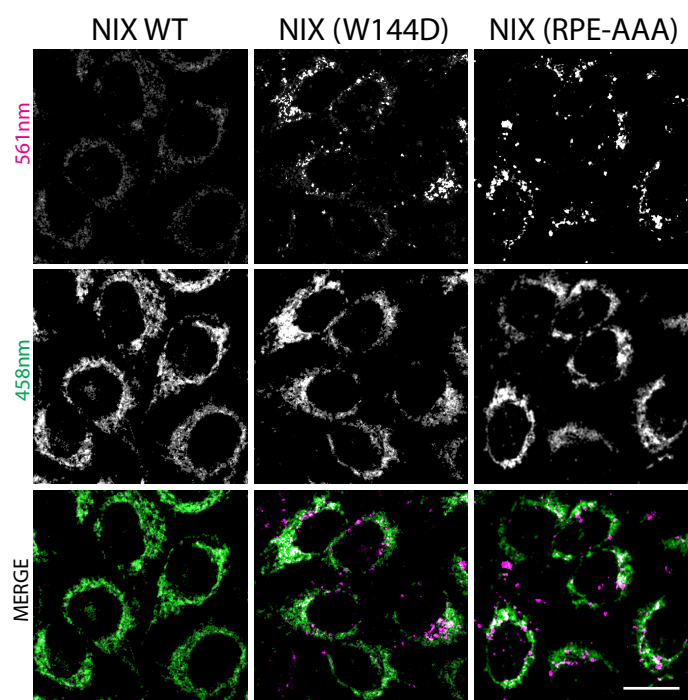

Supplement: Supplementary file 6 — Source data Fig. 4 [file 44319_2024_181_MOESM6_ESM.zip › Figure 4/Figure 4G/Annotation 4G.pdf]

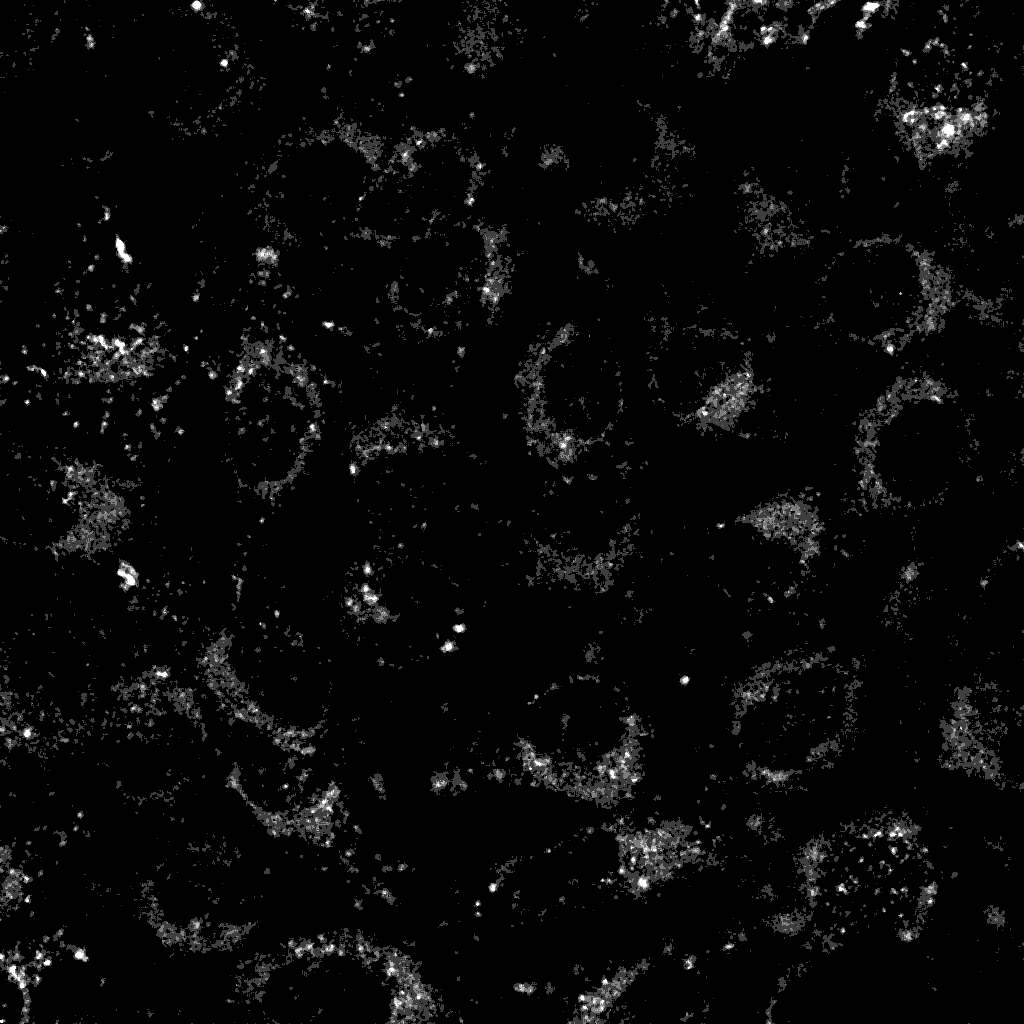

Supplement: Supplementary file 6 — Source data Fig. 4 [file 44319_2024_181_MOESM6_ESM.zip › Figure 4/Figure 4G/Hela-Flp-In_DKO-3C9+ FLAG-NIX-RPE-AAA.tif]

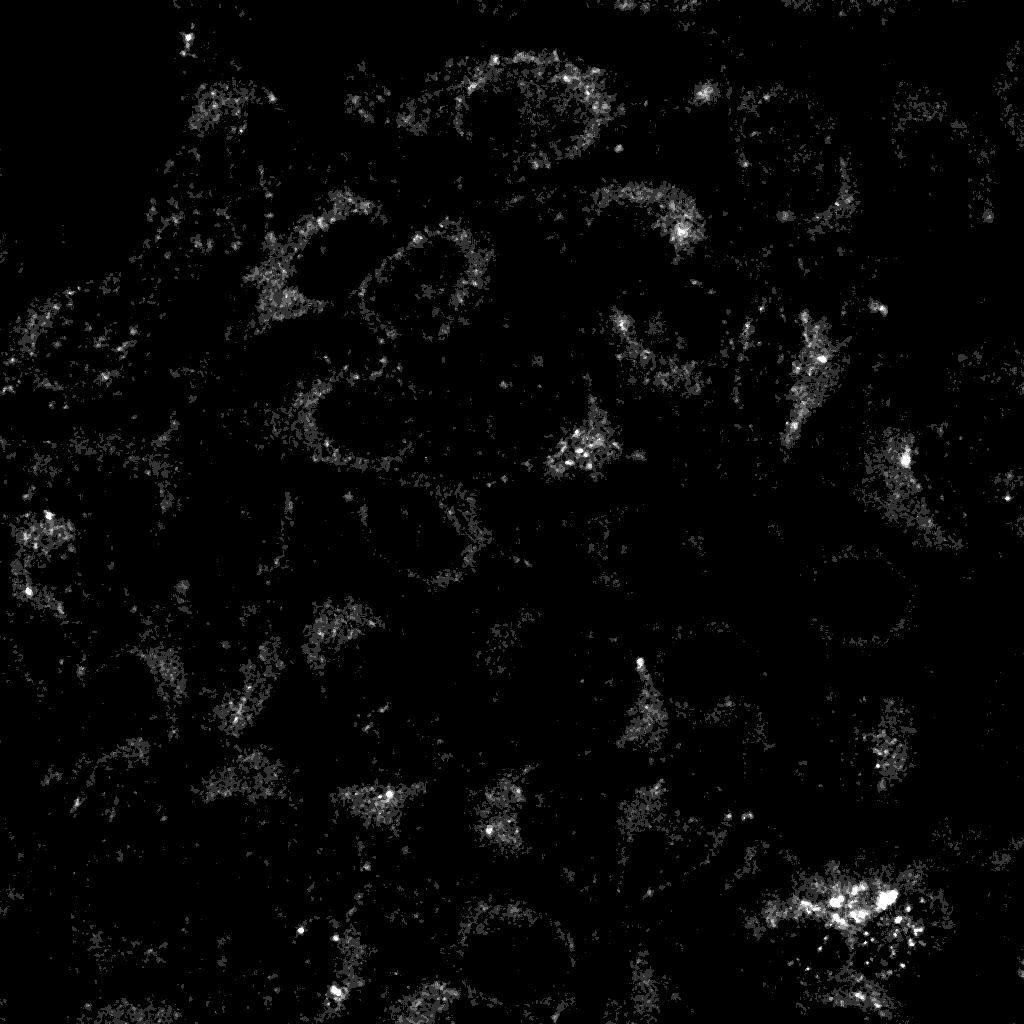

Supplement: Supplementary file 6 — Source data Fig. 4 [file 44319_2024_181_MOESM6_ESM.zip › Figure 4/Figure 4G/Hela-Flp-In_DKO-3C9+ FLAG-NIX-W144D.tif]

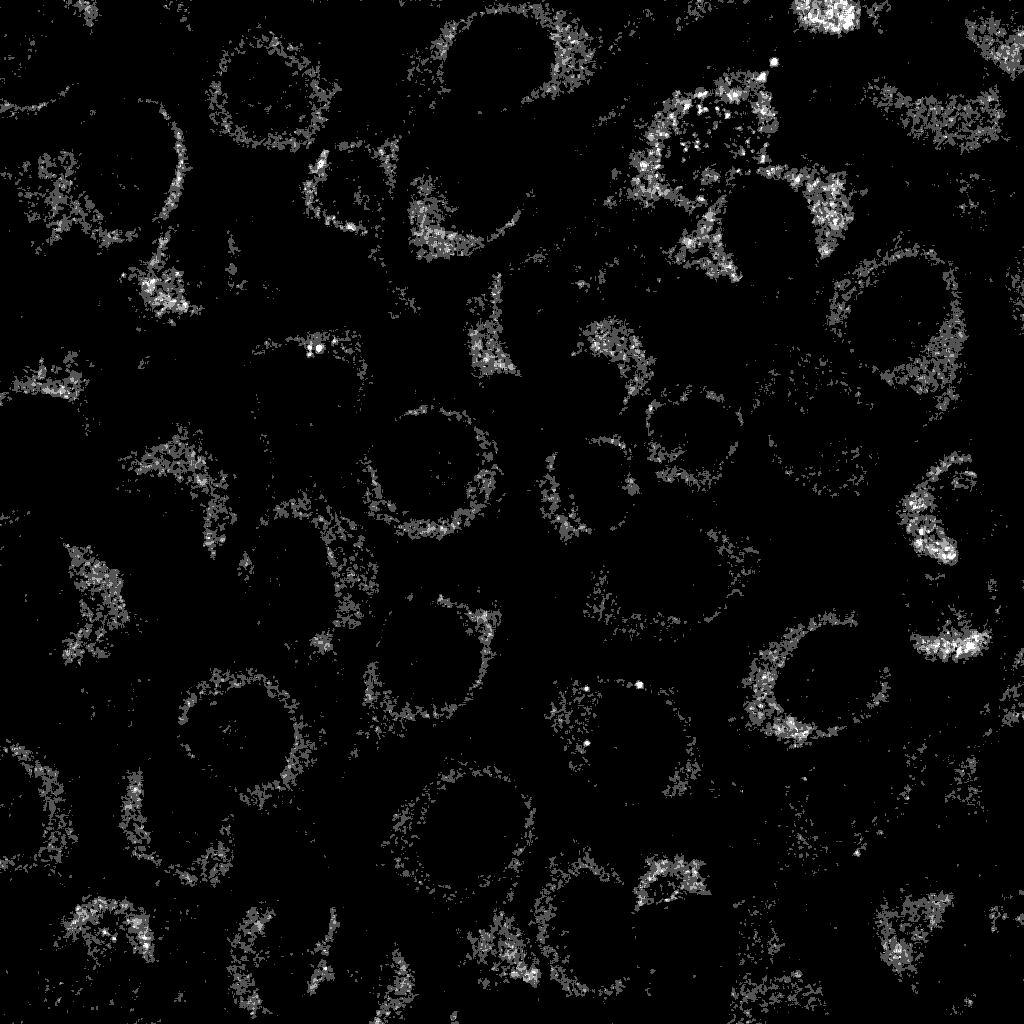

Supplement: Supplementary file 6 — Source data Fig. 4 [file 44319_2024_181_MOESM6_ESM.zip › Figure 4/Figure 4G/Hela-Flp-In_DKO-3C9+ FLAG-NIX-WT.tif]

Whole Cell Lysates

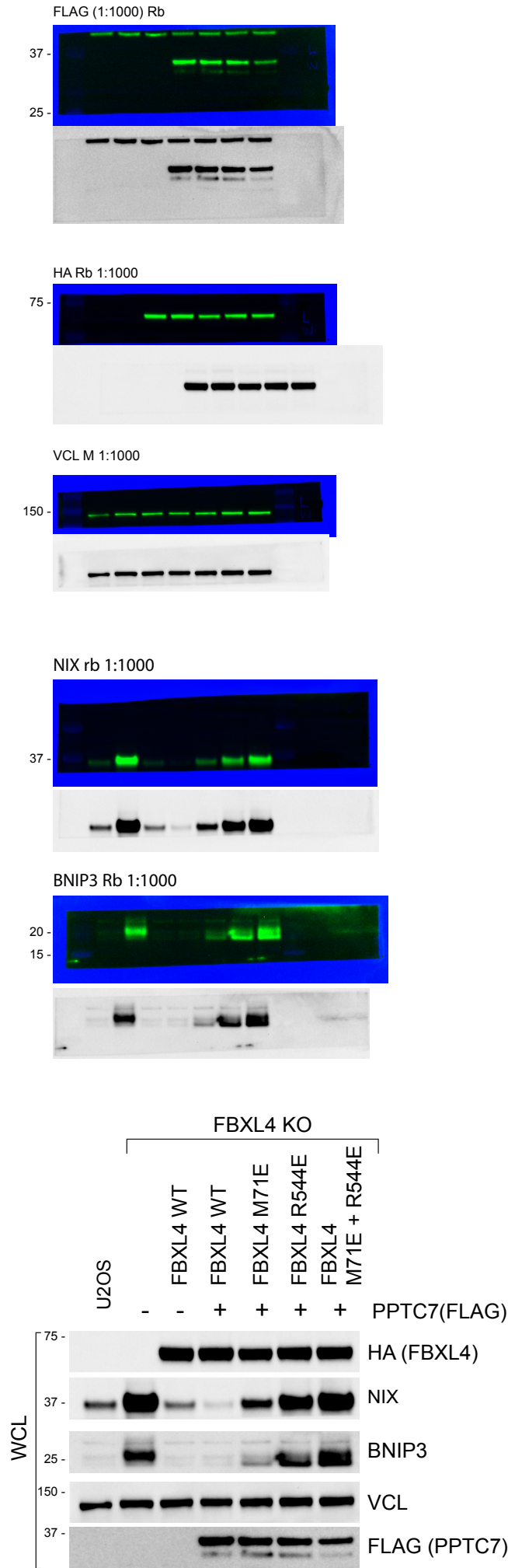

Immunoprecipitates

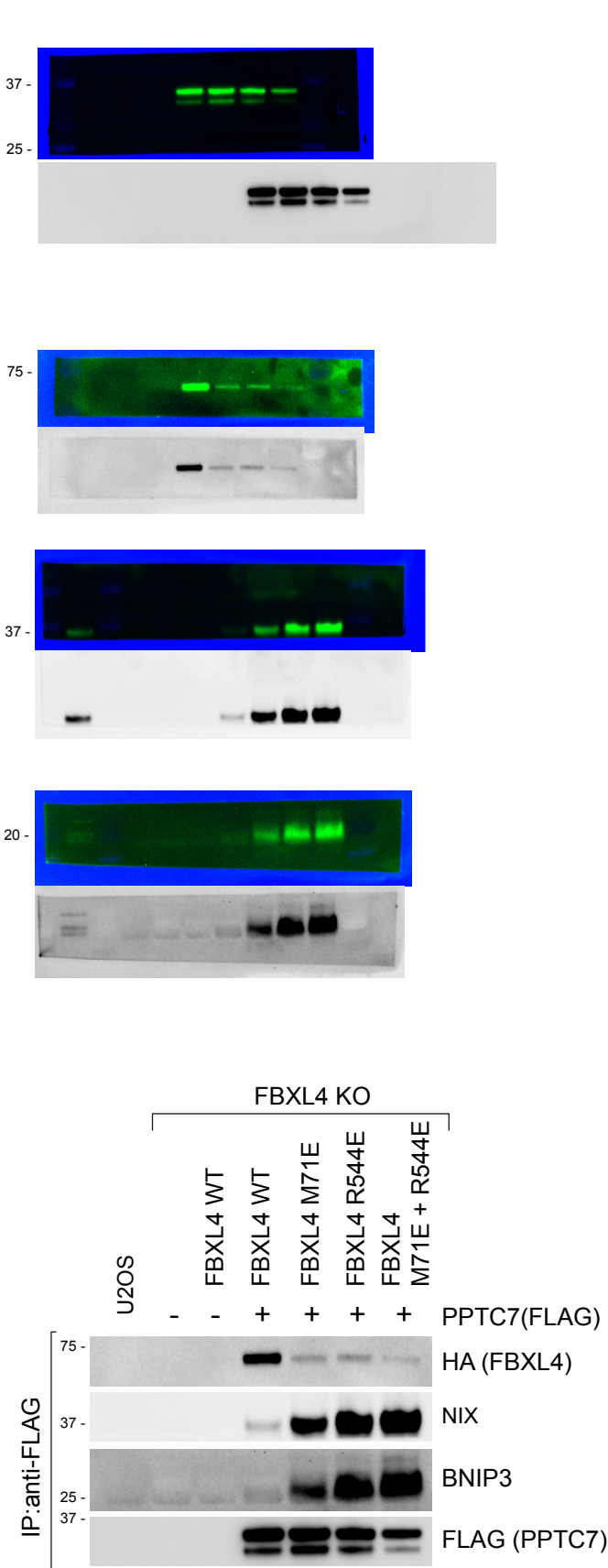

Supplement: Supplementary file 7 — Source data Fig. 5 [file 44319_2024_181_MOESM7_ESM.zip › Figure 5/Figure 5B/Annotation Figure 5B.pdf]

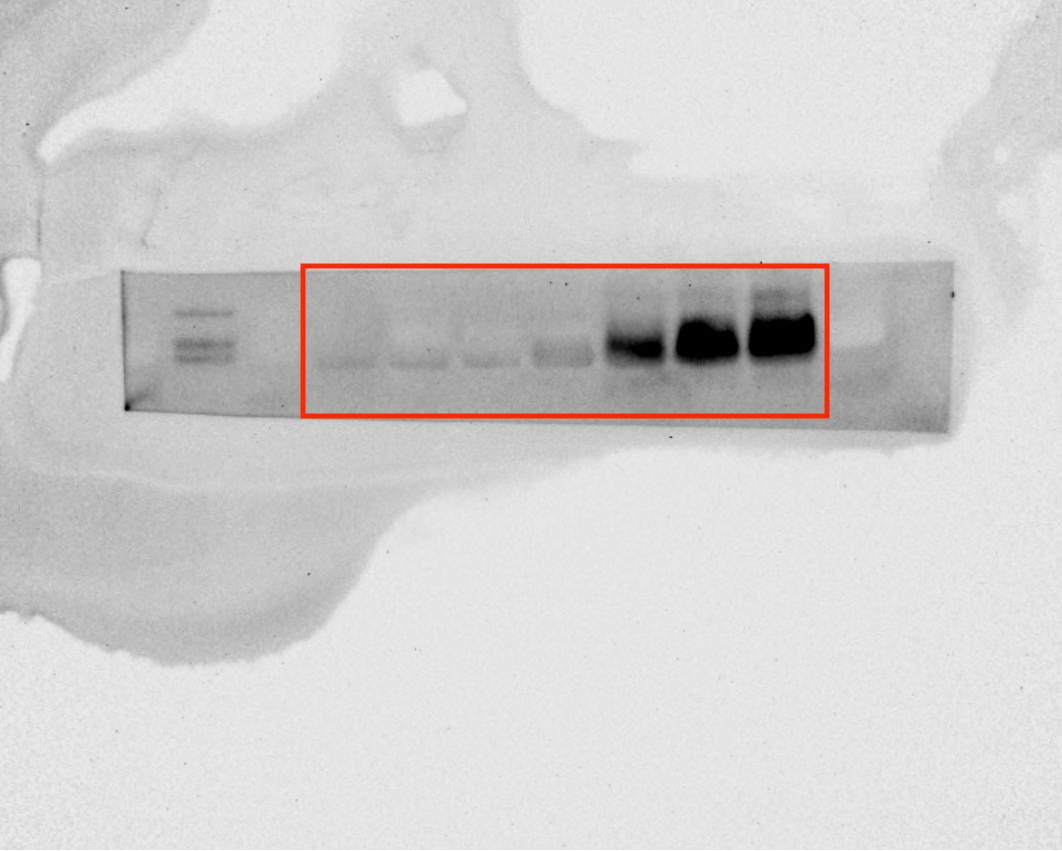

Supplement: Supplementary file 7 — Source data Fig. 5 [file 44319_2024_181_MOESM7_ESM.zip › Figure 5/Figure 5B/ip bnip3.tif]

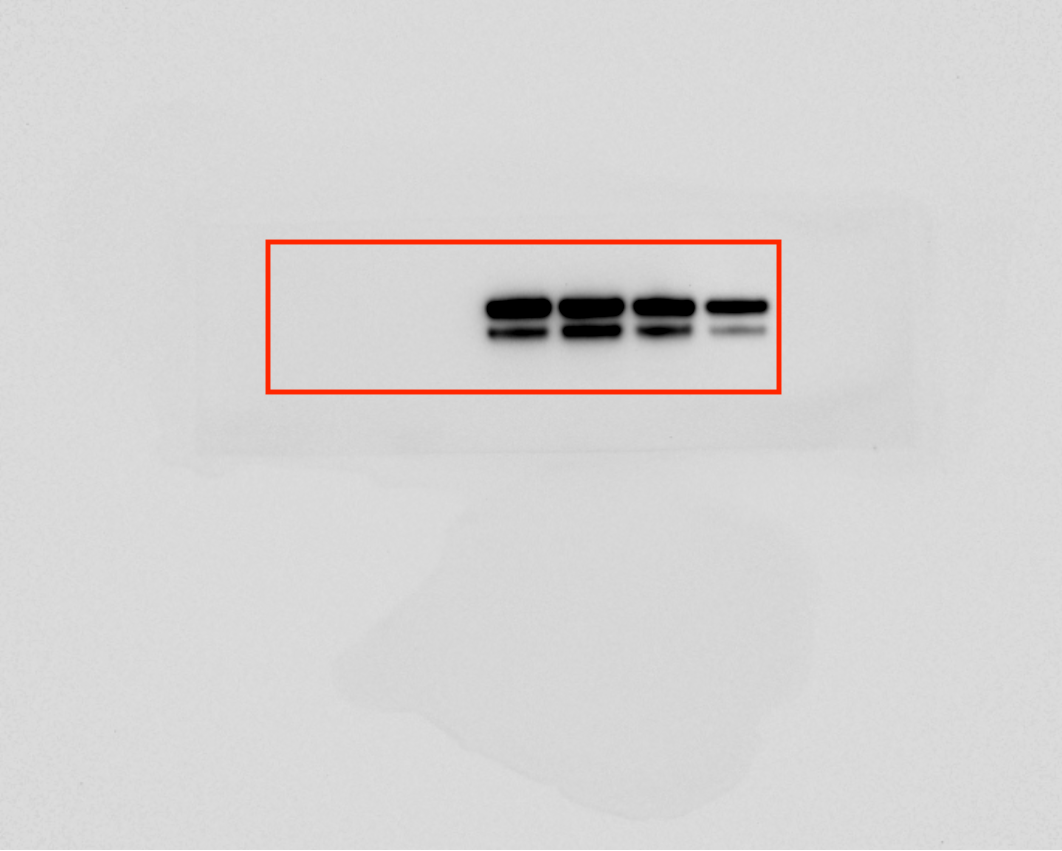

Supplement: Supplementary file 7 — Source data Fig. 5 [file 44319_2024_181_MOESM7_ESM.zip › Figure 5/Figure 5B/ip flag (PPTC7).tif]

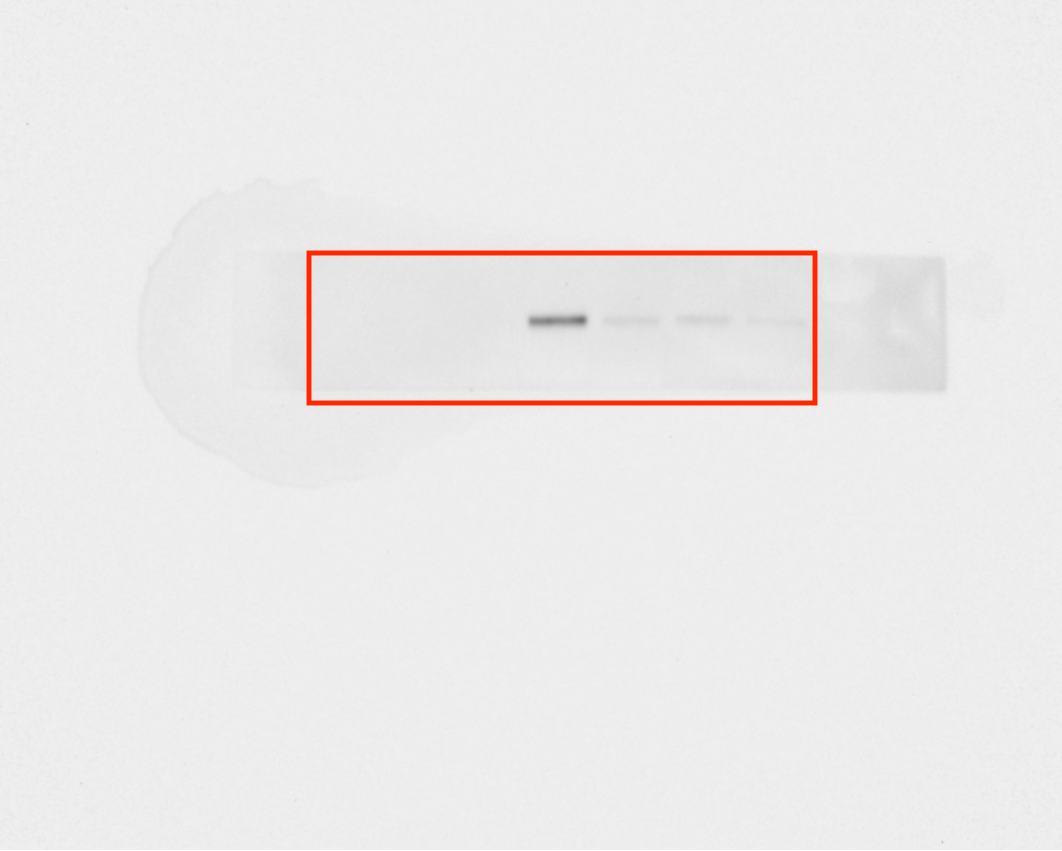

Supplement: Supplementary file 7 — Source data Fig. 5 [file 44319_2024_181_MOESM7_ESM.zip › Figure 5/Figure 5B/ip ha (l4) ha.tif]

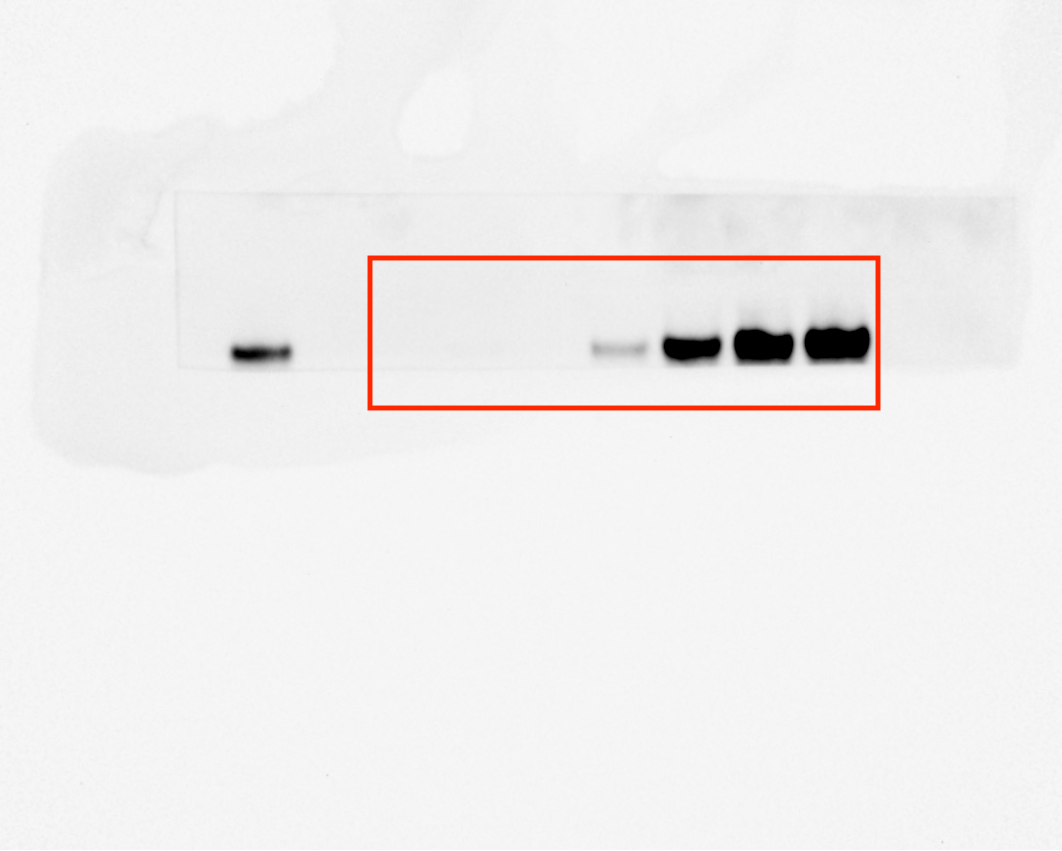

Supplement: Supplementary file 7 — Source data Fig. 5 [file 44319_2024_181_MOESM7_ESM.zip › Figure 5/Figure 5B/ip nix.tif]

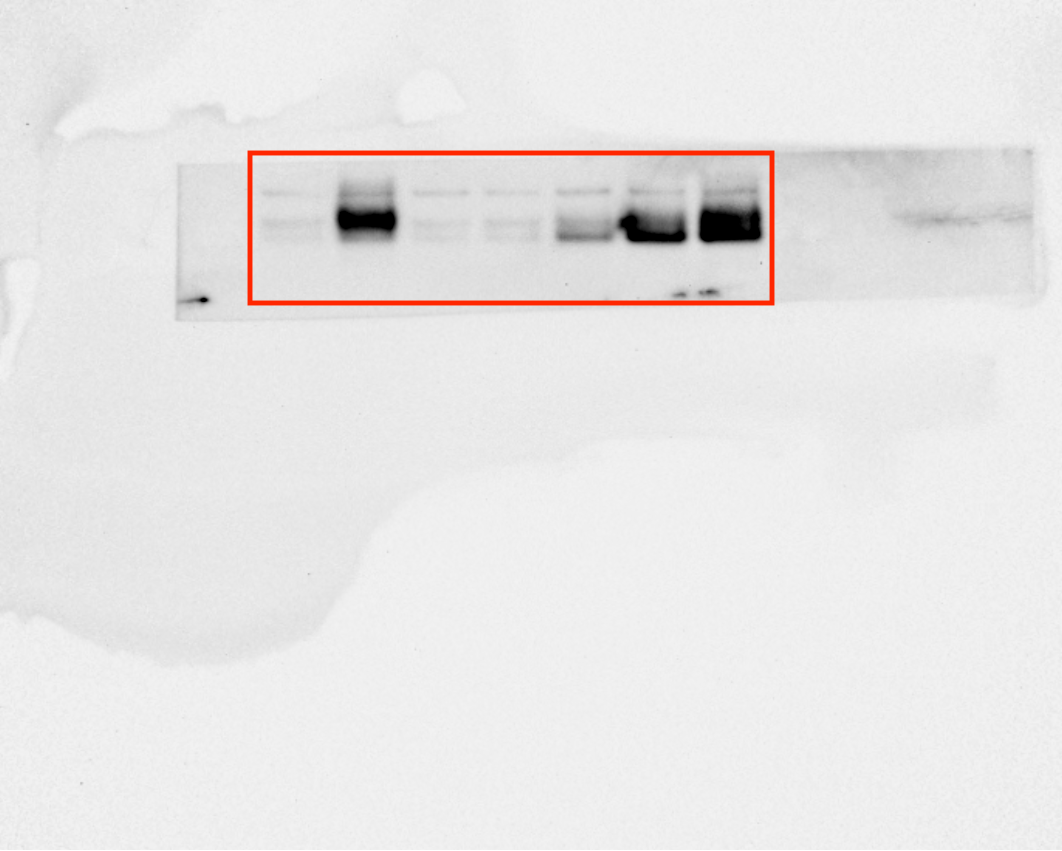

Supplement: Supplementary file 7 — Source data Fig. 5 [file 44319_2024_181_MOESM7_ESM.zip › Figure 5/Figure 5B/wcl bnip3.tif]

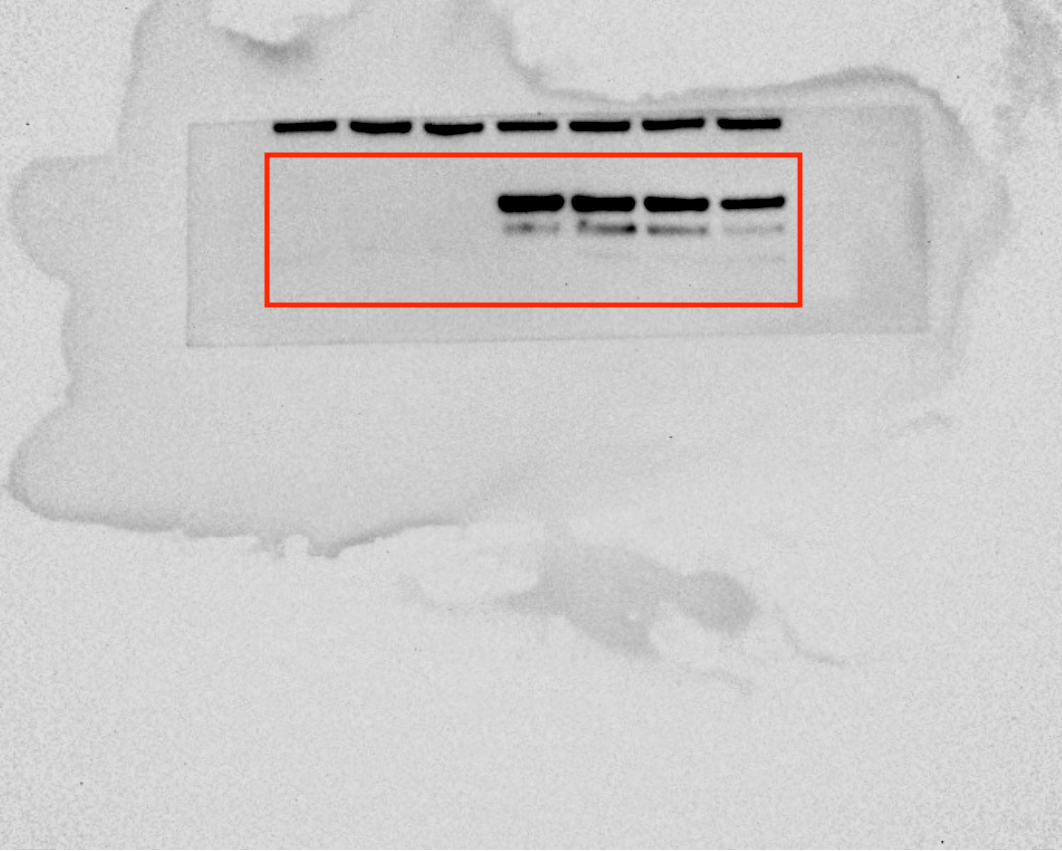

Supplement: Supplementary file 7 — Source data Fig. 5 [file 44319_2024_181_MOESM7_ESM.zip › Figure 5/Figure 5B/wcl flag.tif]

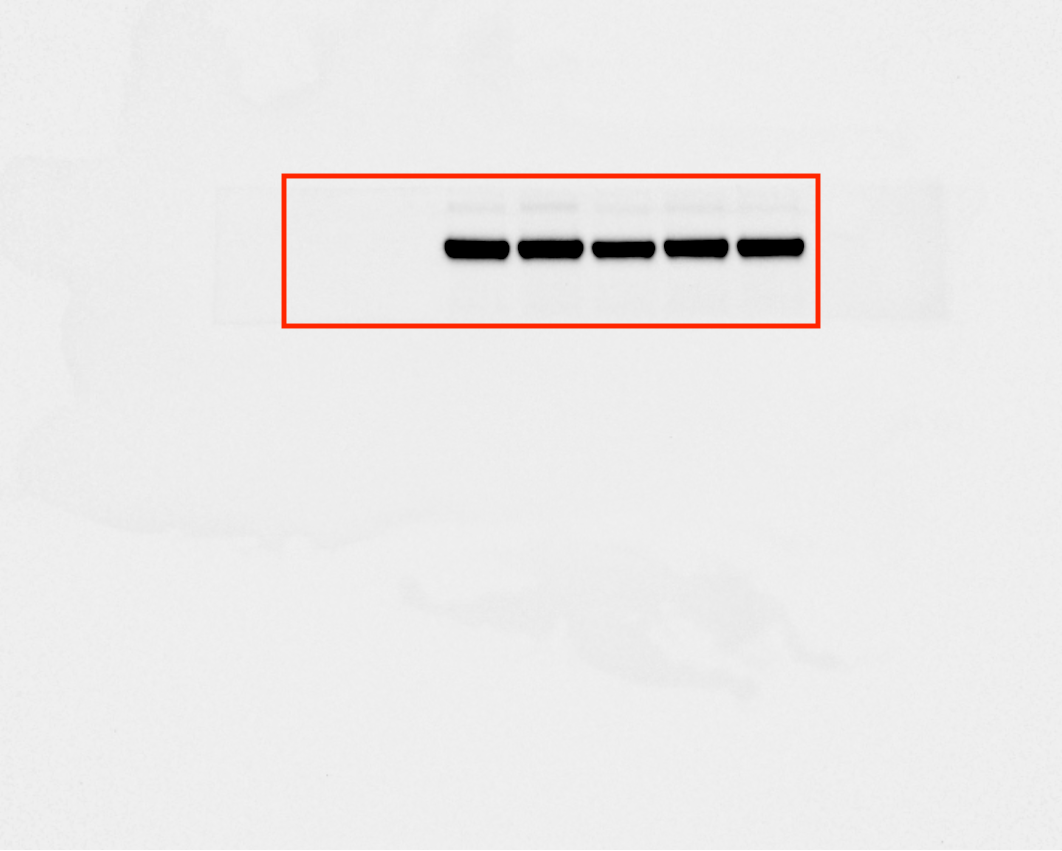

Supplement: Supplementary file 7 — Source data Fig. 5 [file 44319_2024_181_MOESM7_ESM.zip › Figure 5/Figure 5B/wcl ha.tif]

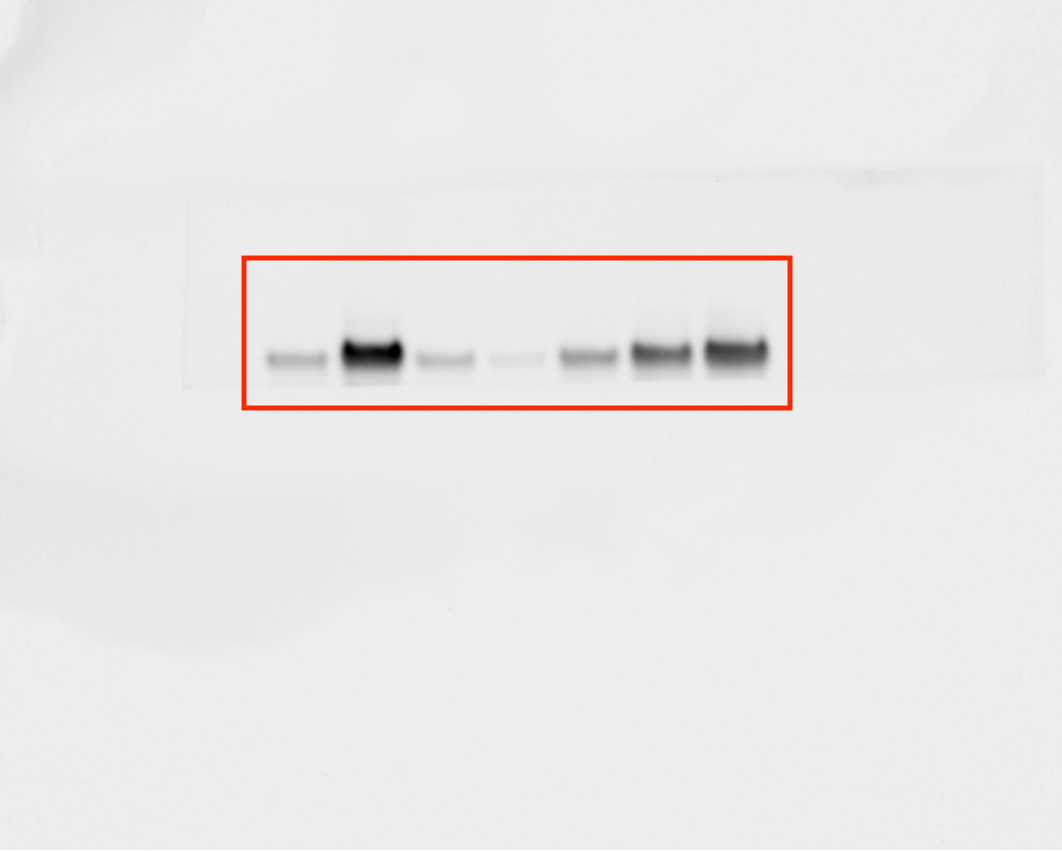

Supplement: Supplementary file 7 — Source data Fig. 5 [file 44319_2024_181_MOESM7_ESM.zip › Figure 5/Figure 5B/wcl nix.tif]

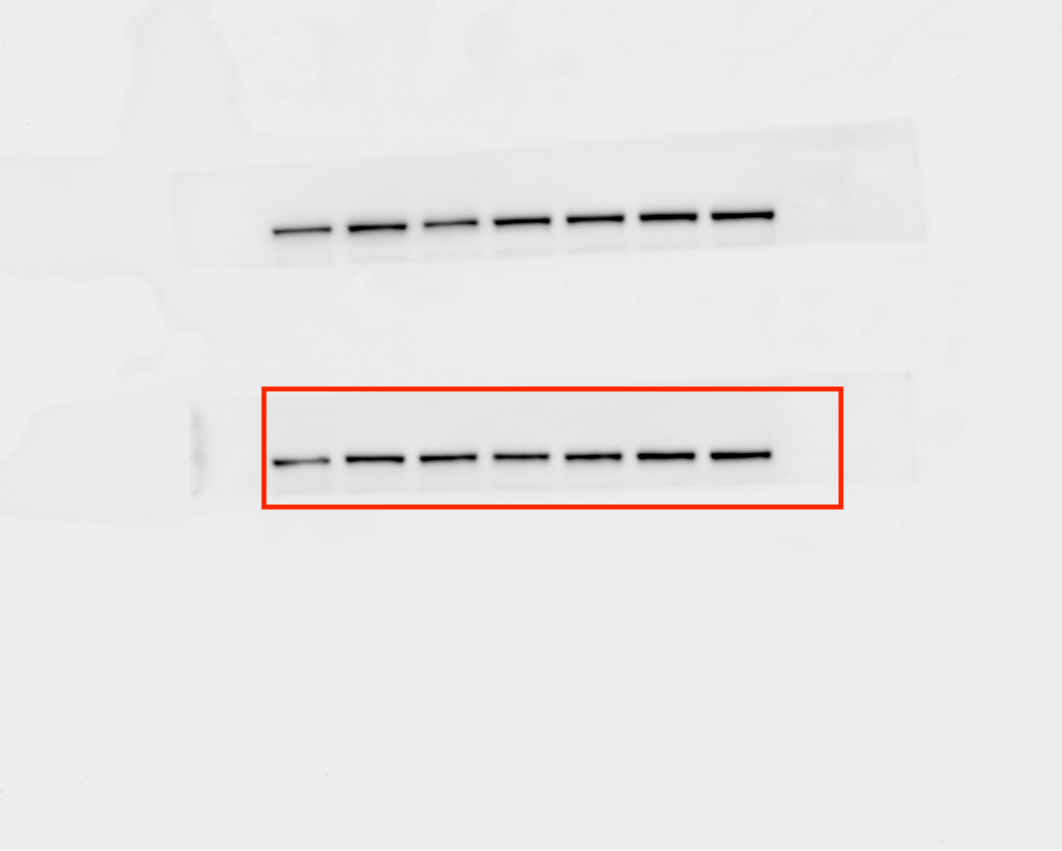

Supplement: Supplementary file 7 — Source data Fig. 5 [file 44319_2024_181_MOESM7_ESM.zip › Figure 5/Figure 5B/wcl vcl.tif]

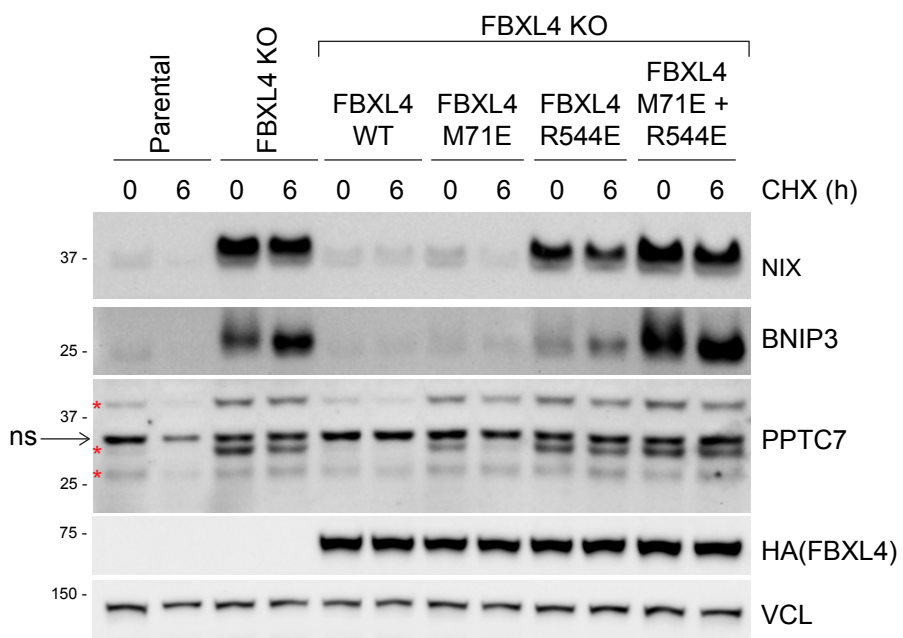

Supplement: Supplementary file 7 — Source data Fig. 5 [file 44319_2024_181_MOESM7_ESM.zip › Figure 5/Figure 5C/Annotations Figure 5C.pdf]

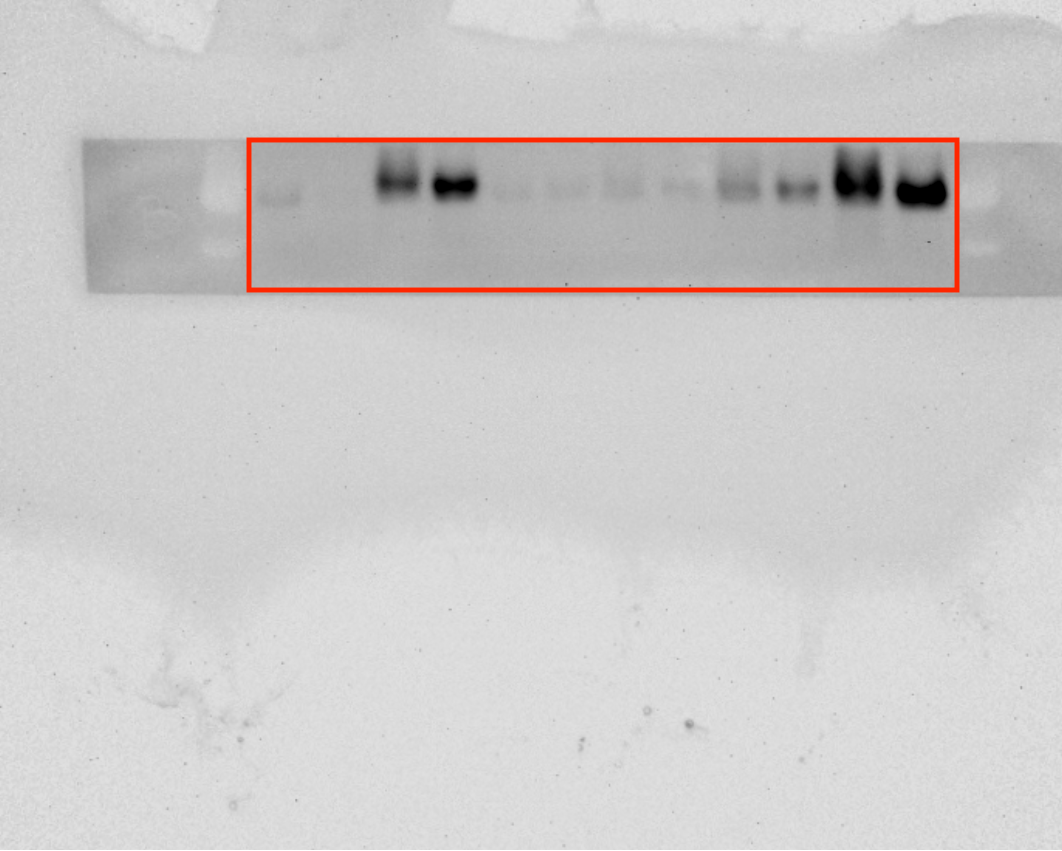

Supplement: Supplementary file 7 — Source data Fig. 5 [file 44319_2024_181_MOESM7_ESM.zip › Figure 5/Figure 5C/BNIP3.tif]

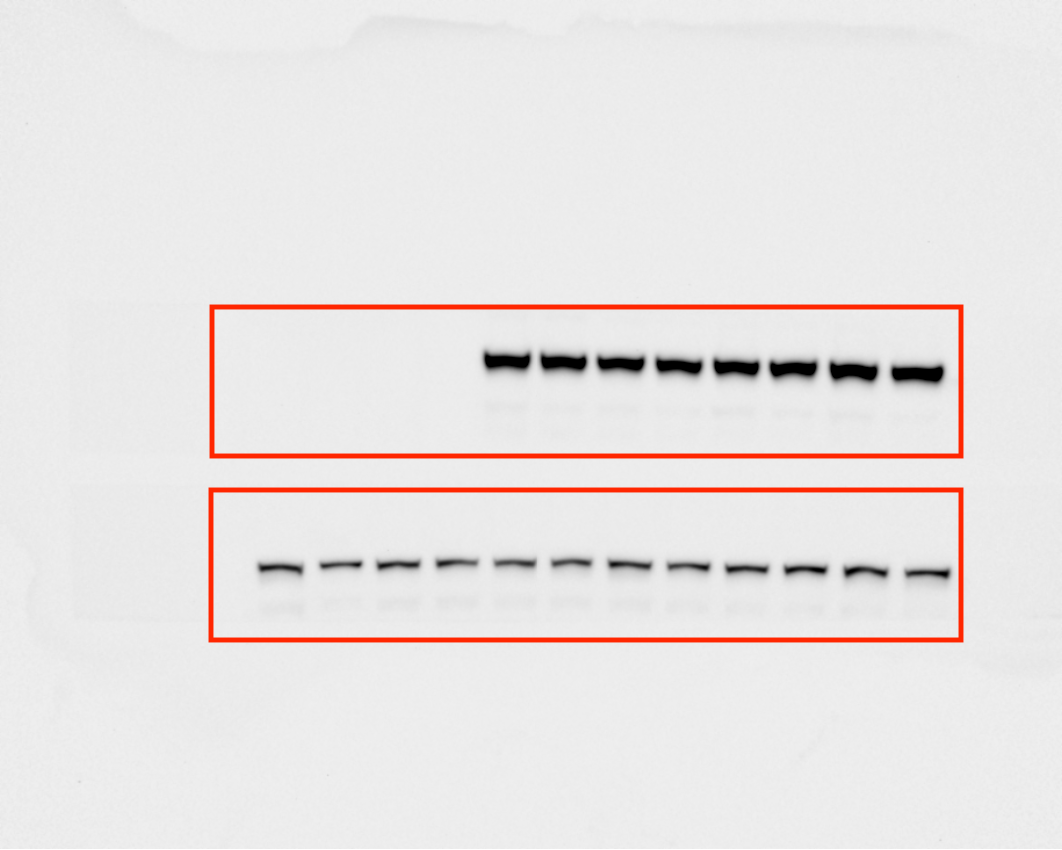

Supplement: Supplementary file 7 — Source data Fig. 5 [file 44319_2024_181_MOESM7_ESM.zip › Figure 5/Figure 5C/HA-FBXL4-top_VCL-bottom.tif]

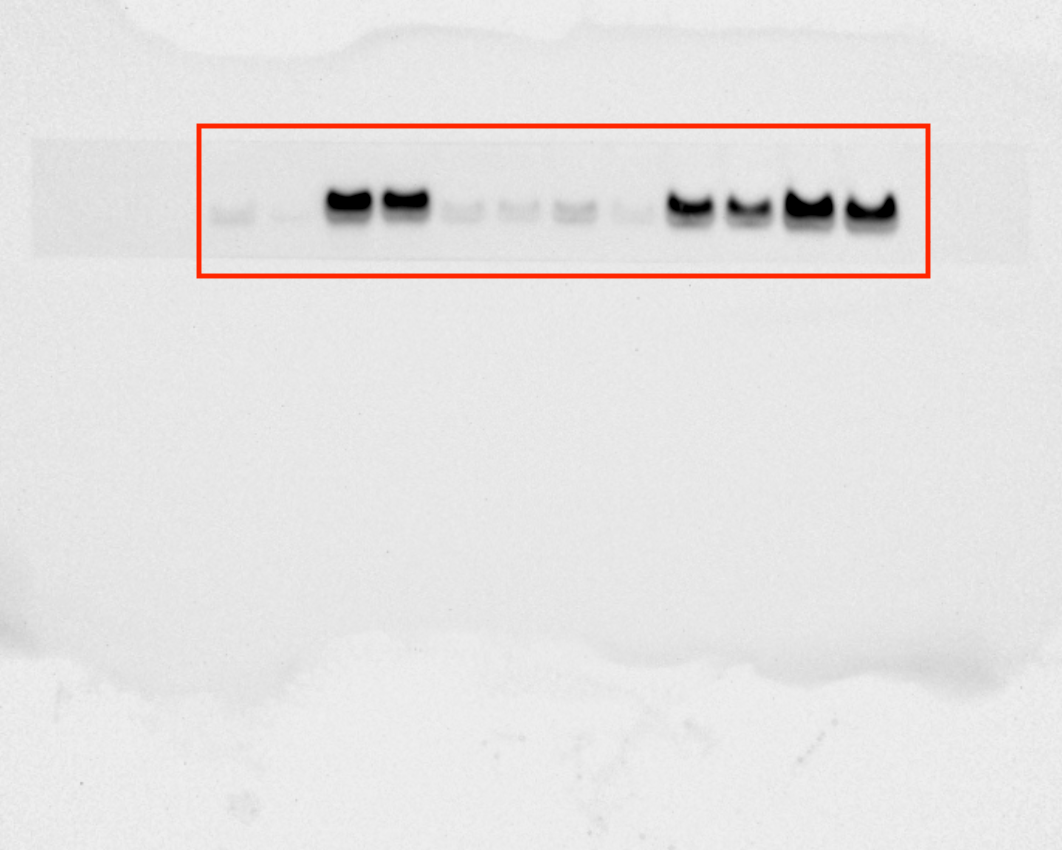

Supplement: Supplementary file 7 — Source data Fig. 5 [file 44319_2024_181_MOESM7_ESM.zip › Figure 5/Figure 5C/NIX.tif]

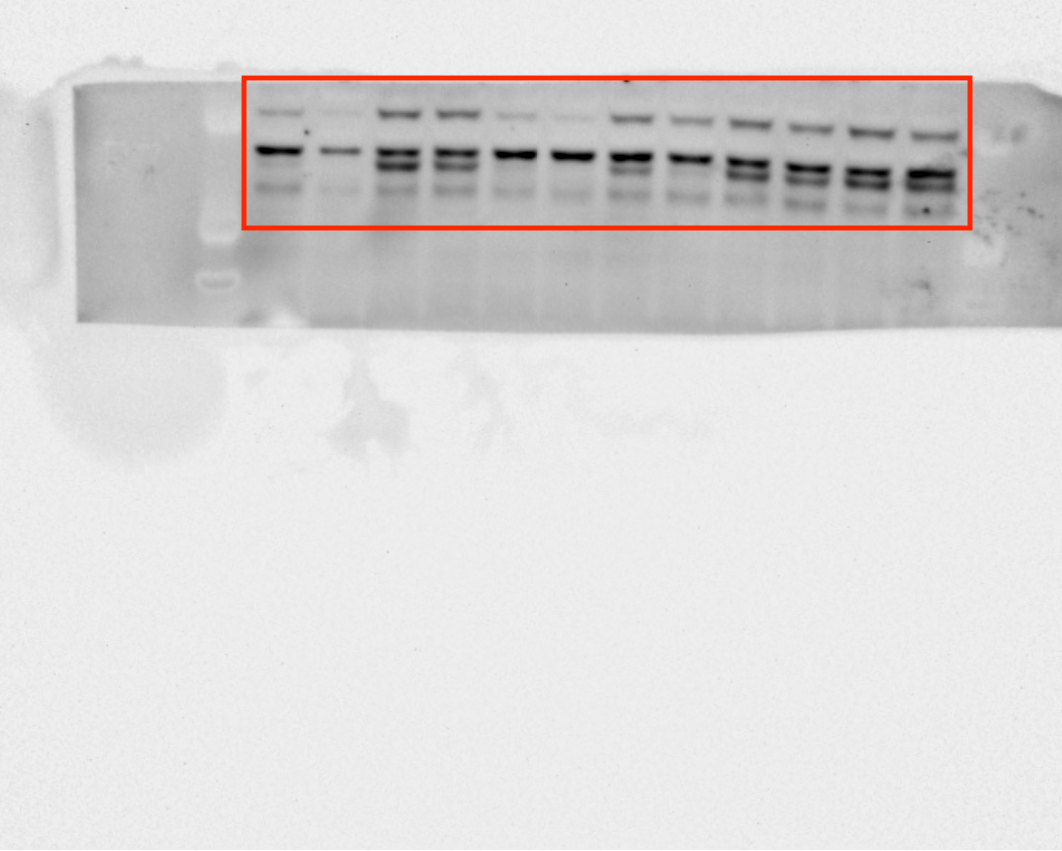

Supplement: Supplementary file 7 — Source data Fig. 5 [file 44319_2024_181_MOESM7_ESM.zip › Figure 5/Figure 5C/PPTC7.tif]

FBXL4 KO

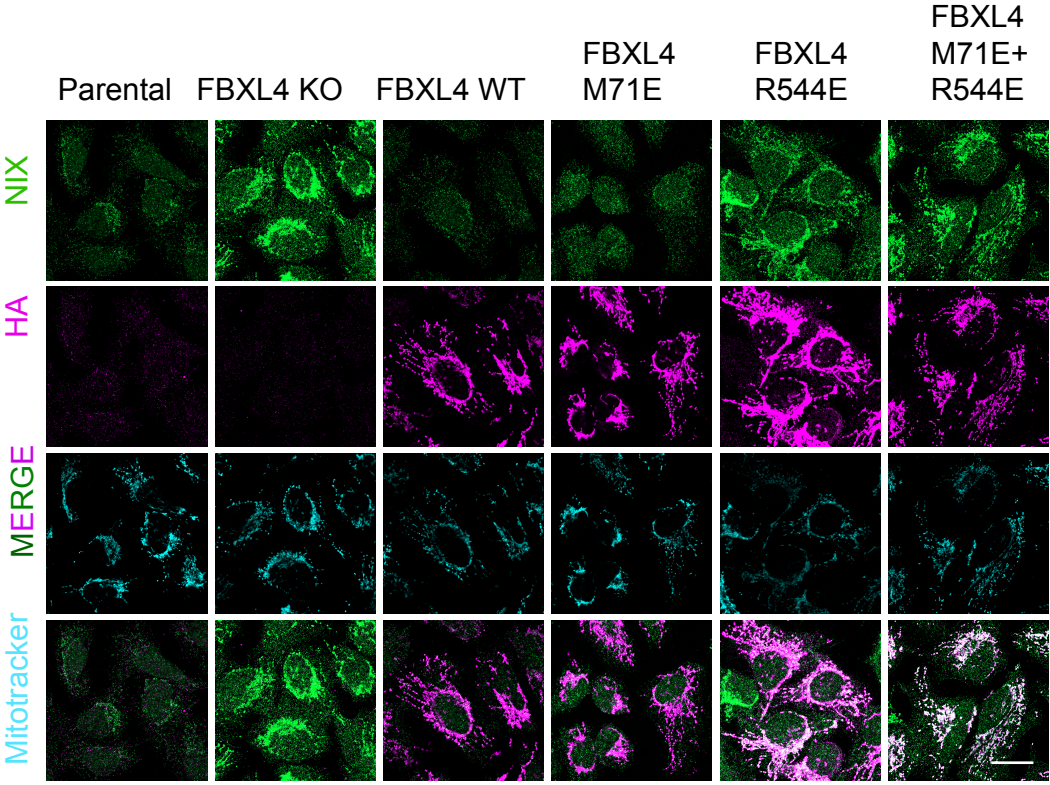

Supplement: Supplementary file 7 — Source data Fig. 5 [file 44319_2024_181_MOESM7_ESM.zip › Figure 5/Figure 5D/Annotation Figure 5D.pdf]

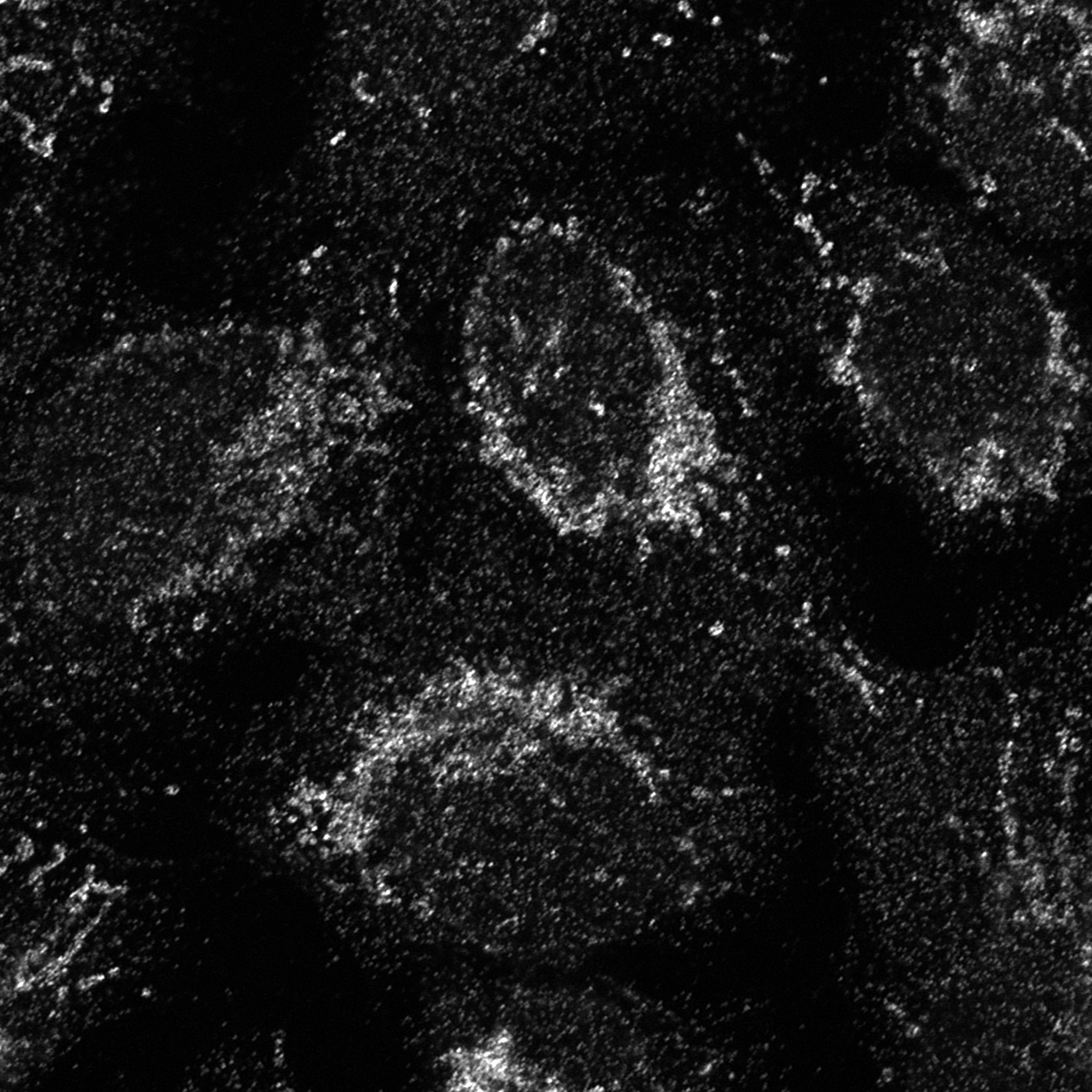

Supplement: Supplementary file 7 — Source data Fig. 5 [file 44319_2024_181_MOESM7_ESM.zip › Figure 5/Figure 5D/FBXL4 KO.tif]

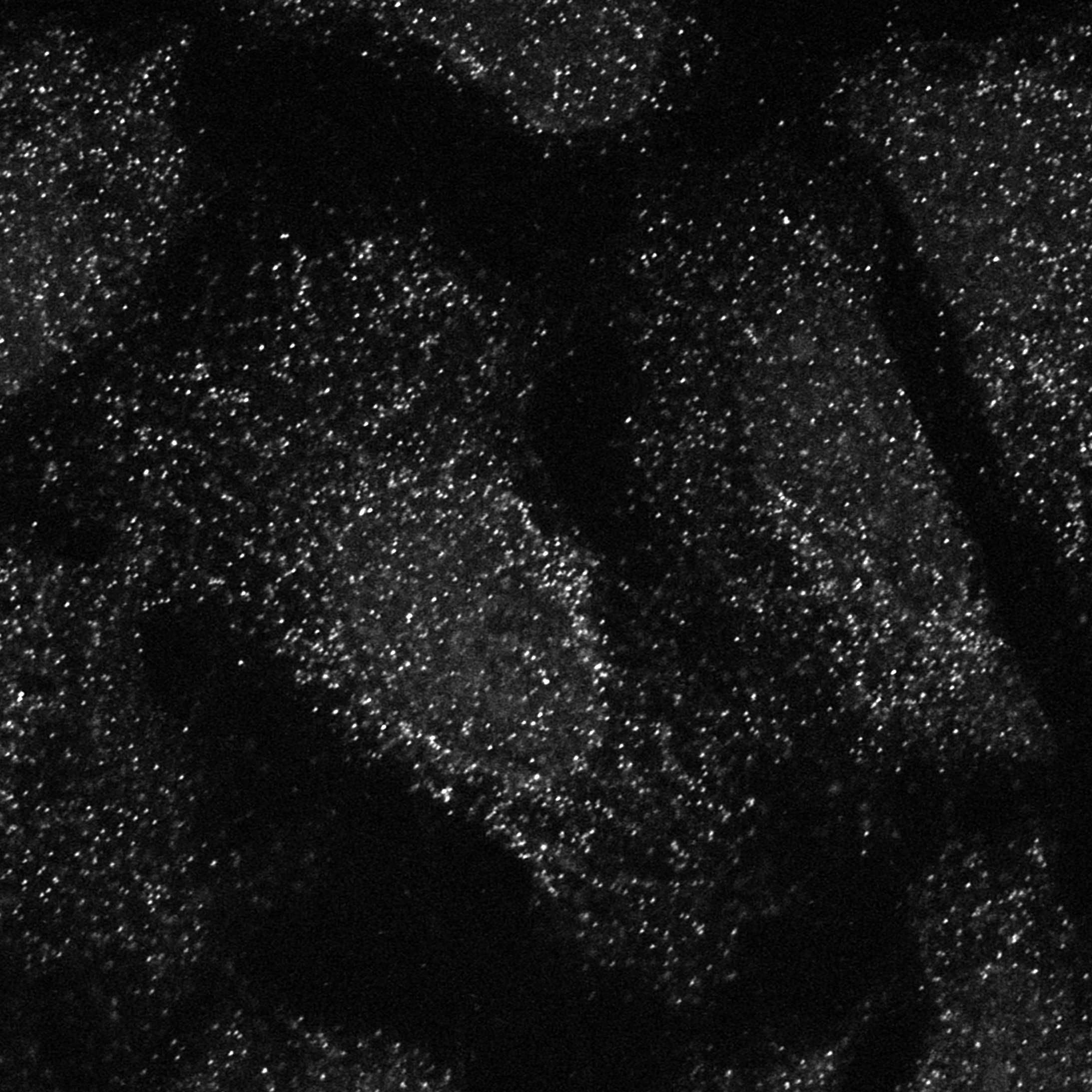

Supplement: Supplementary file 7 — Source data Fig. 5 [file 44319_2024_181_MOESM7_ESM.zip › Figure 5/Figure 5D/FBXL4 WT.tif]

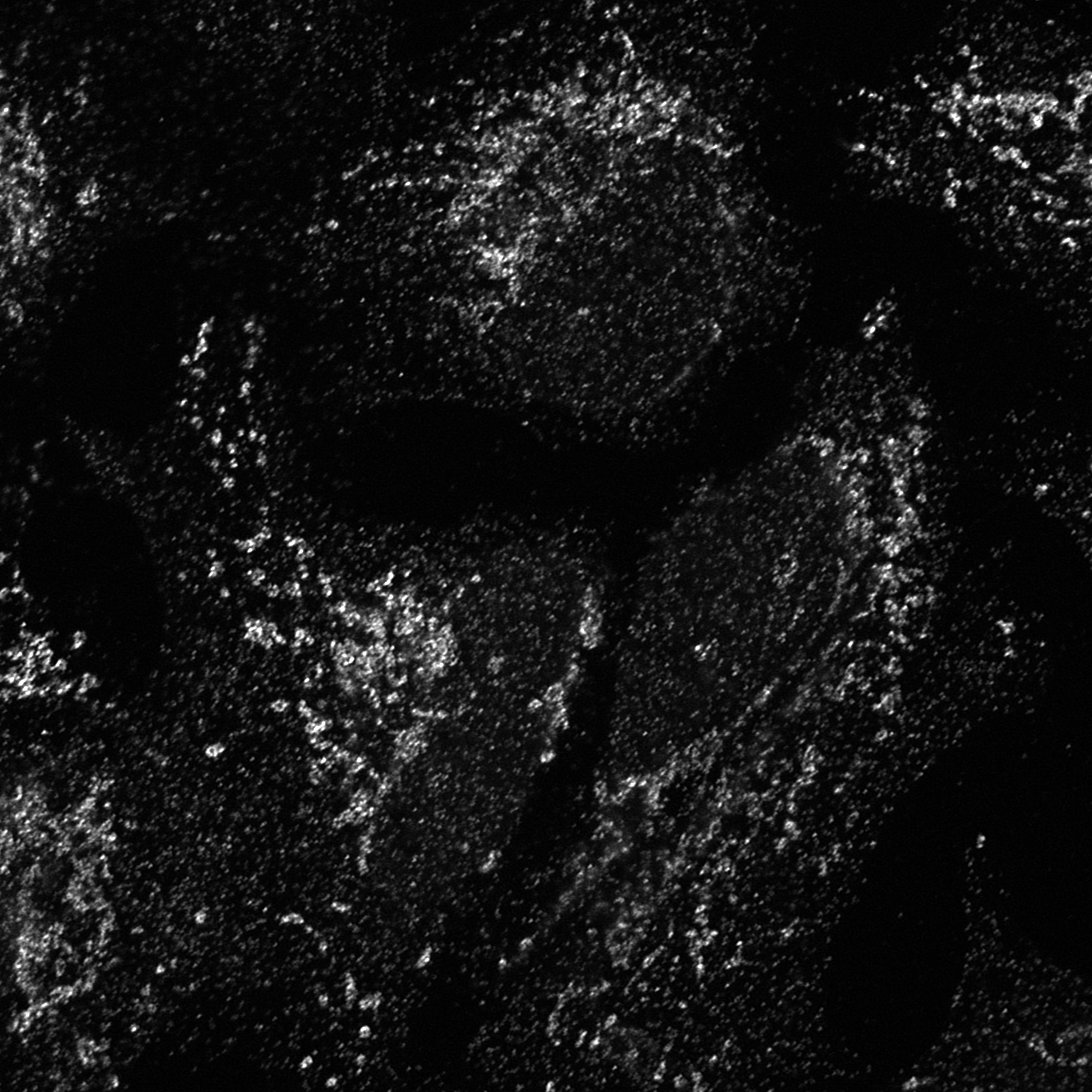

Supplement: Supplementary file 7 — Source data Fig. 5 [file 44319_2024_181_MOESM7_ESM.zip › Figure 5/Figure 5D/M71+R544.tif]

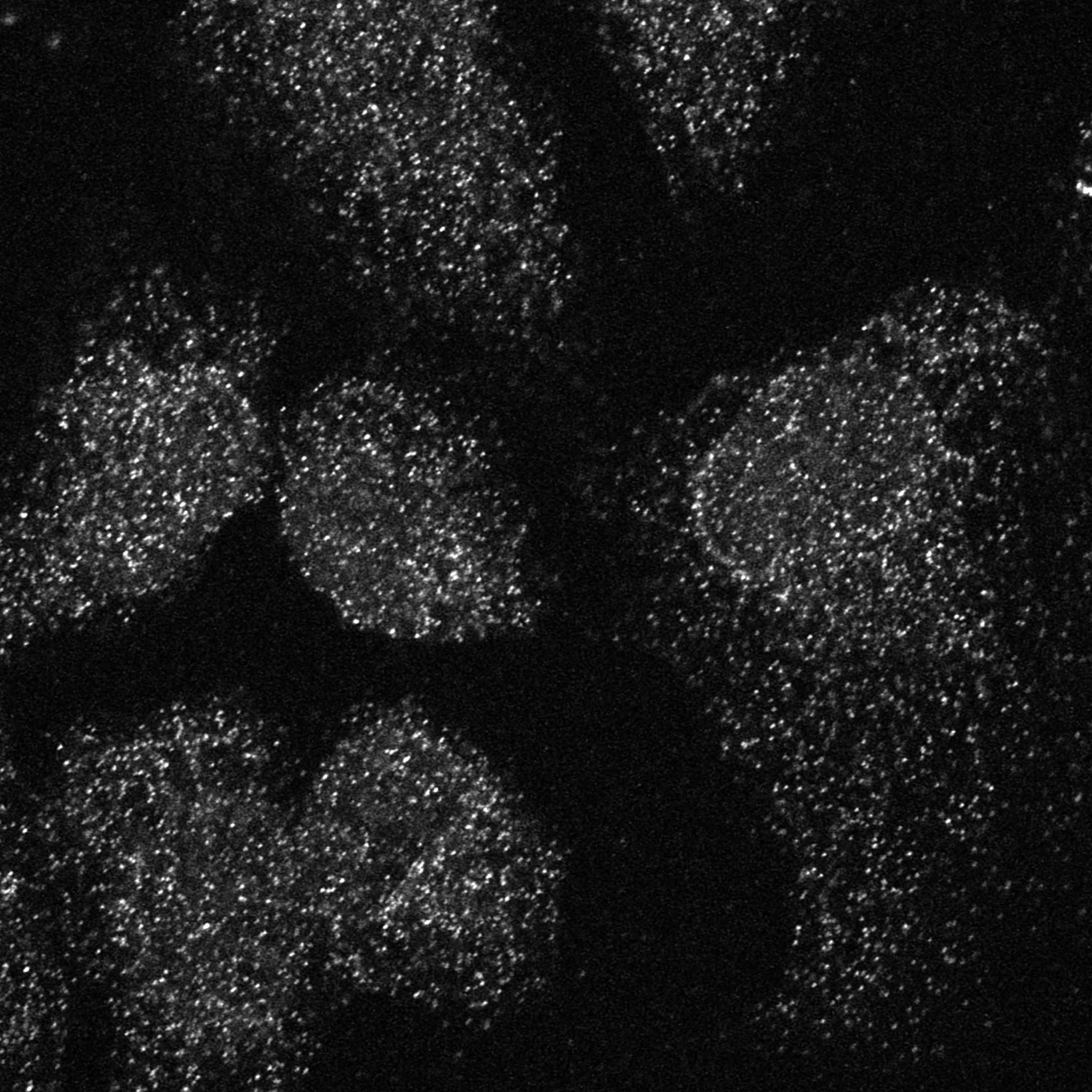

Supplement: Supplementary file 7 — Source data Fig. 5 [file 44319_2024_181_MOESM7_ESM.zip › Figure 5/Figure 5D/M71E.tif]

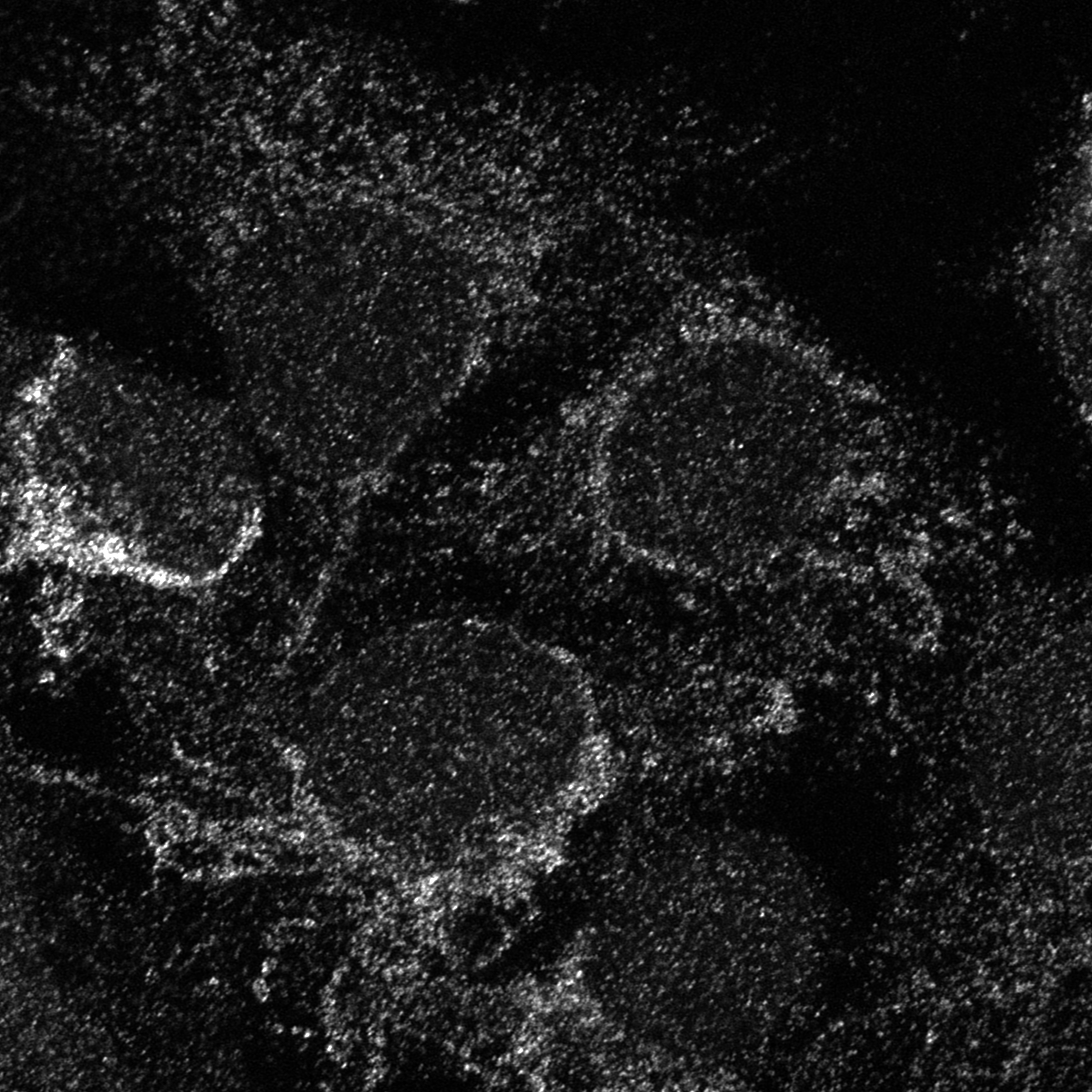

Supplement: Supplementary file 7 — Source data Fig. 5 [file 44319_2024_181_MOESM7_ESM.zip › Figure 5/Figure 5D/R544.tif]

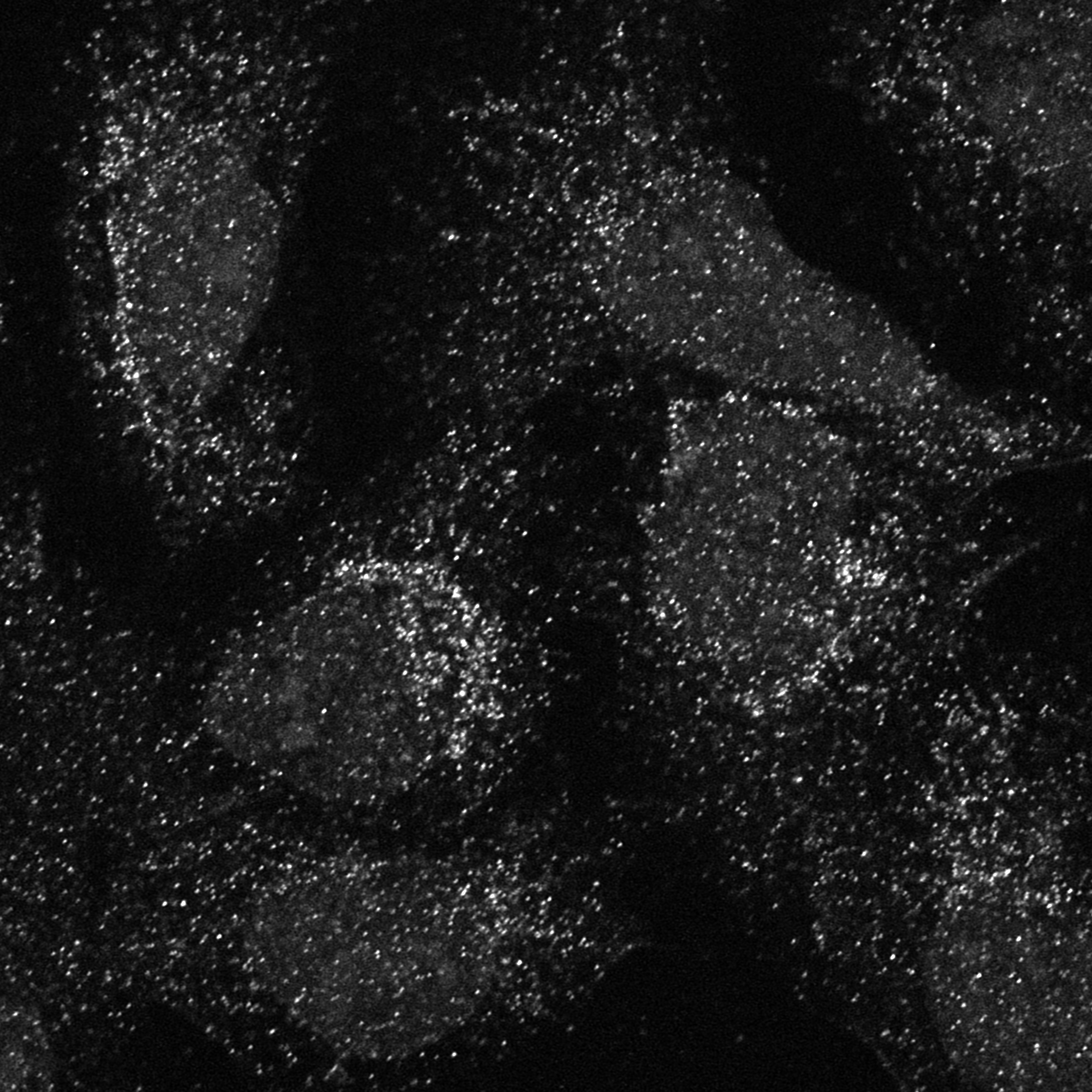

Supplement: Supplementary file 7 — Source data Fig. 5 [file 44319_2024_181_MOESM7_ESM.zip › Figure 5/Figure 5D/U2OS.tif]

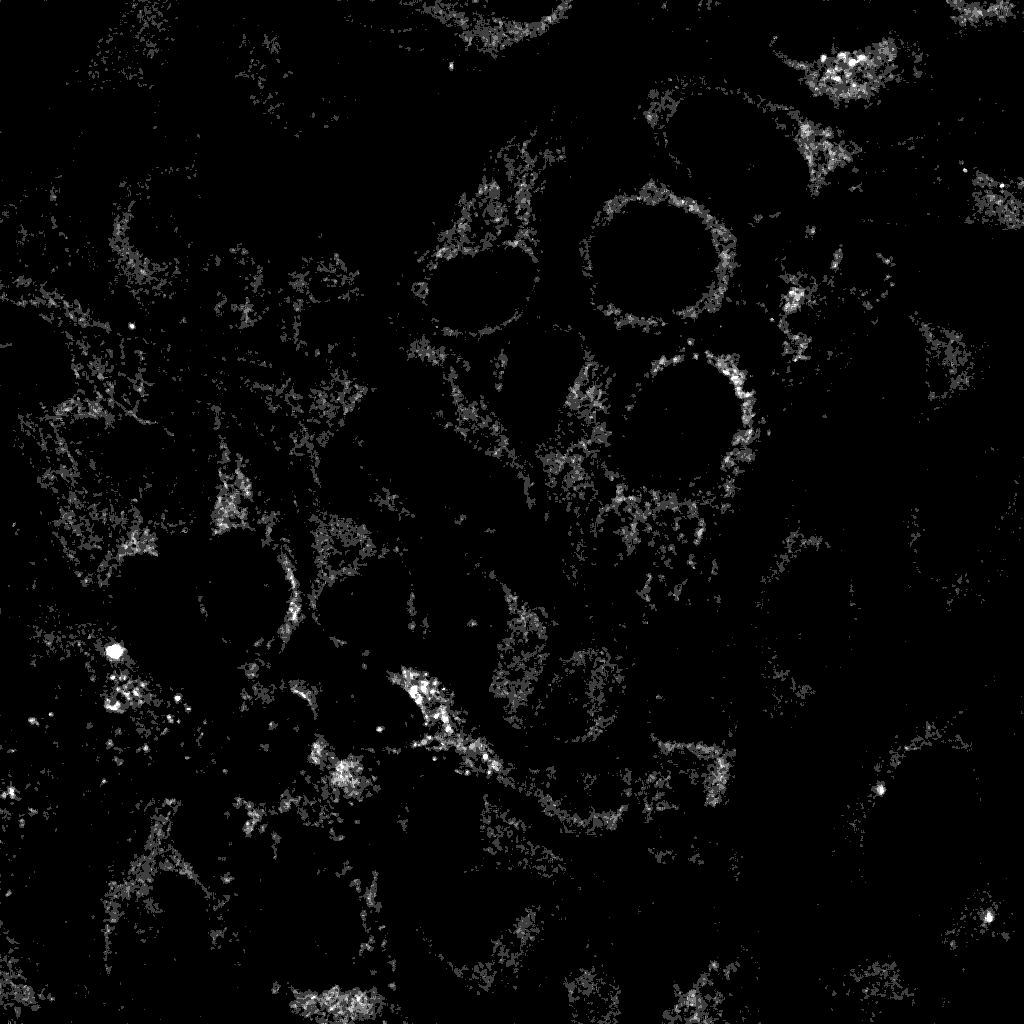

Supplement: Supplementary file 7 — Source data Fig. 5 [file 44319_2024_181_MOESM7_ESM.zip › Figure 5/Figure 5E/U2OS-FBXL4KO+FBXL4-WT.tif]

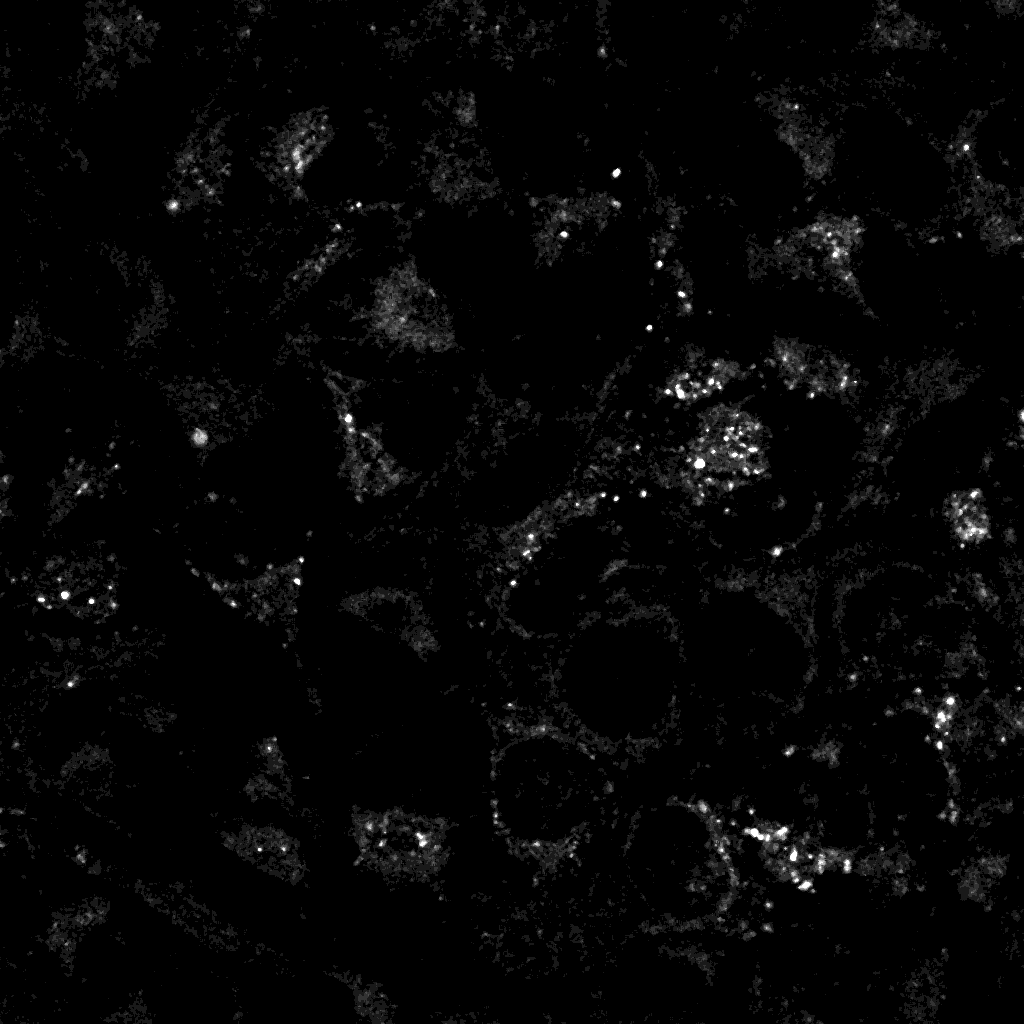

Supplement: Supplementary file 7 — Source data Fig. 5 [file 44319_2024_181_MOESM7_ESM.zip › Figure 5/Figure 5E/U2OS-FBXL4KO+FBXL4M71:R544.tif]

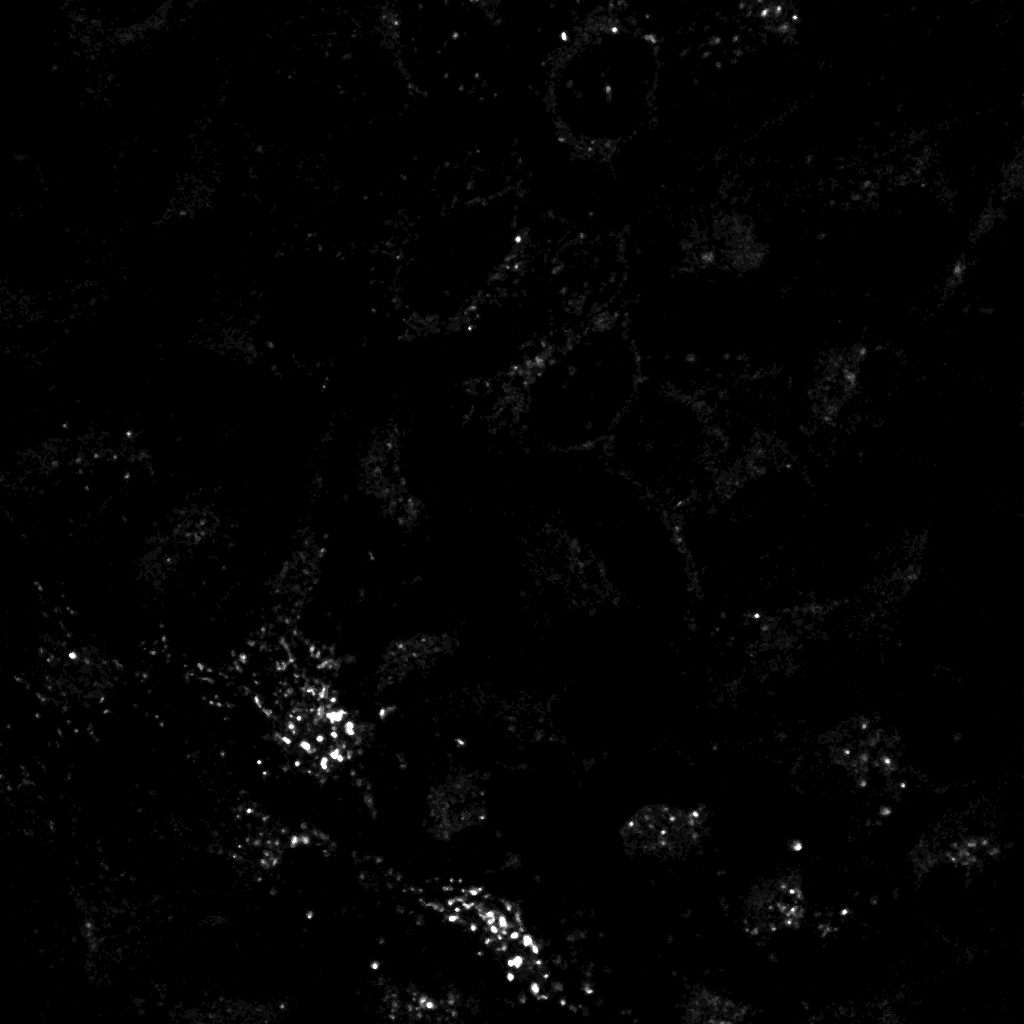

Supplement: Supplementary file 7 — Source data Fig. 5 [file 44319_2024_181_MOESM7_ESM.zip › Figure 5/Figure 5E/U2OS-FBXL4KO.tif]

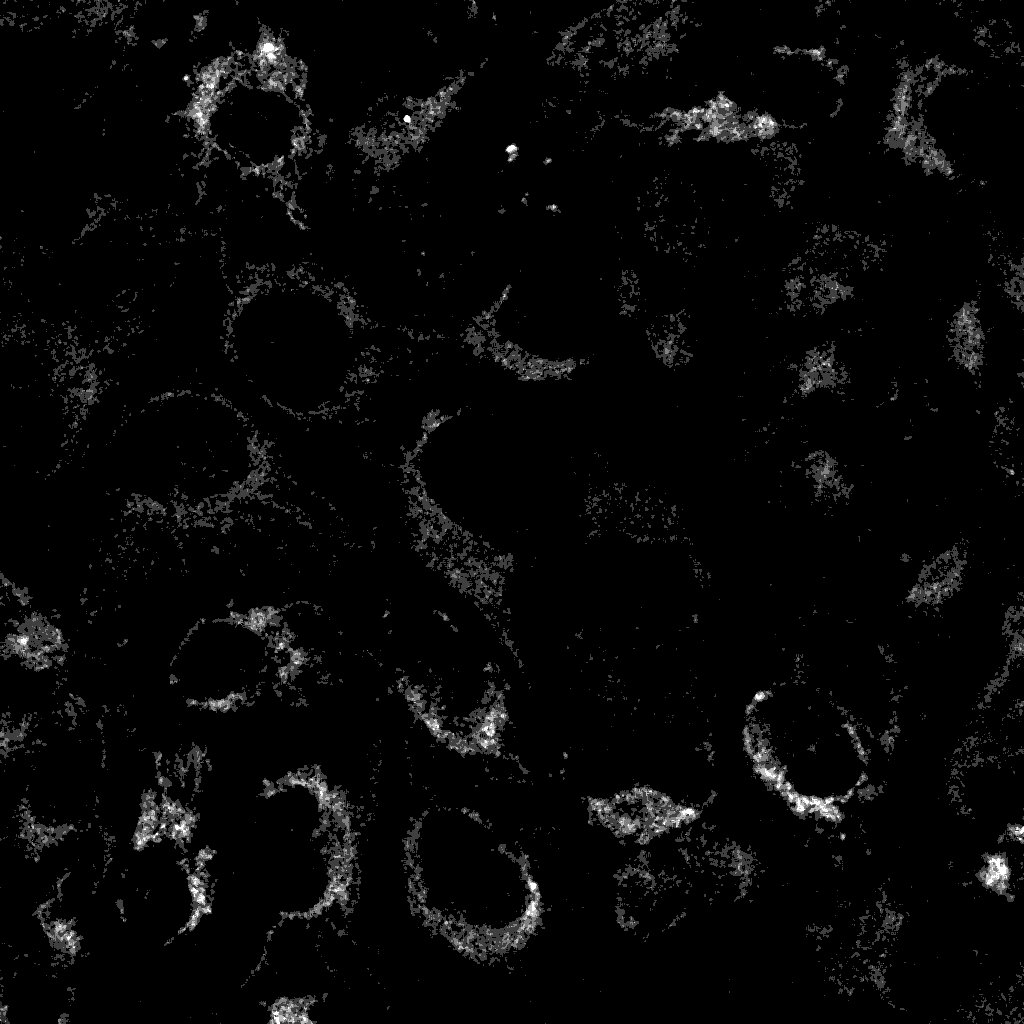

Supplement: Supplementary file 7 — Source data Fig. 5 [file 44319_2024_181_MOESM7_ESM.zip › Figure 5/Figure 5E/U2OS.tif]
