# Supplementary material for: PPTC7 antagonizes mitophagy by promoting BNIP3 and NIX degradation via SCFFBXL4
Source: EMBO Rep. 2024 Jul 11;25(8):3324–47. doi: 10.1038/s44319-024-00181-y (PMC11316107; doi:10.1038/s44319-024-00181-y)
Supplement: Supplementary file 8 — Expanded View Figures [file 44319_2024_181_MOESM8_ESM.pdf]

# Expanded View Figures

**Figure EV1. PPTC7 is required for the FBXL4-mediated destabilization of NIX/BNIP3.**

(**A**) Distinct guide RNAs targeting PPTC7 result in BNIP3 and NIX upregulation. Cells were transfected PPTC7 guide 1 (G1) or guide 2 (G2). (**B**) BNIP3 and NIX are stabilised in PPTC7-deficient 293T cells. Cells were treated with cycloheximide for the indicated times before immunoblotting as indicated. (**C**) Inhibition of HIF1α with echinomycin does not prevent the accumulation of BNIP3 and NIX in PPTC7-deficient cells. U2OS cells or PPTC7 KO cells were treated with echinomycin for 24 h. Echinomycin completely prevented the DFP-induced upregulation of BNIP3 and NIX, but only partially prevented the accumulation of BNIP3 and NIX in PPTC7 KO cells. DFP induces the 32 kDa form of PPTC7. (**D**) FBXL4 requires PPTC7 for its ability to promote BNIP3 and NIX turnover. FBXL4-HA was expressed in parental, FBXL4 KO, PPTC7 KO, and FBXL4/PPTC7 dKO cells. BNIP3 and NIX protein levels were monitored by Western blotting in response to FBXL4 expression. (**E**) PPTC7-mediated downregulation of BNIP3 and NIX does not occur in FBXL4-deficient cells. PPTC7-FLAG was transfected into either PPTC7 KO or FBXL4 KO cells. Cells were fixed and stained for FLAG(PPTC7) (green) and either NIX or BNIP3 (magenta). The orange dotted line surrounds the cells that have been transfected with PPTC7. (**F**) PPTC7(HA) overexpression causes the downregulation of BNIP3 and NIX in U2OS and 293T cells in basal conditions and after DFP treatment. PPTC7(HA) was transduced into U2OS or 293T cells and the levels of BNIP3 and NIX were monitored by immunoblotting. (**G**) PPTC7 overexpression results in the downregulation of BNIP3 and NIX in basal conditions as well as after DFP treatment. U2OS cells or U2OS cells stably transfected with PPTC7(HA) were treated with DFP for 24 h. Cells were subjected to cycloheximide chase. s.e. = shorter exposure. (**H**) PPTC7 overexpression suppresses DFP-induced mitophagy. Mitophagy was assessed U2OS mt-Keima cells or U2OS mt-Keima cells overexpressing PPTC7(HA) in the presence or absence of DFP. Emission signals at neutral pH were obtained after excitation with the 458 nm laser (green), and emission signals at acidic pH were obtained after excitation with the 458 nm laser 561 nm laser (magenta). Mitophagy is represented as the ratio of mt-Keima 561 nm fluorescence intensity divided by mt-Keima 458 nm fluorescence intensity for individual cells normalised to the mean of the untreated U2OS cells. Translucent grey dots represent measurements from individual cells. Coloured circles represent the mean ratio from independent experiments. The centre lines and bars represent the mean of the independent replicates +/− standard deviation. *P* values were calculated based on the mean values using a one-way ANOVA. ****$P<0.0001$. $n = 3$ independent experiments. Data Information: (**E**, **H**) Scale bar = 20 microns. (**B**, **C**) The red asterisks indicate PPTC7-specific bands and 28 kDa, 32 kDa, and 40 kDa. Arrow and ns=non-specific band at ~36 kDa.

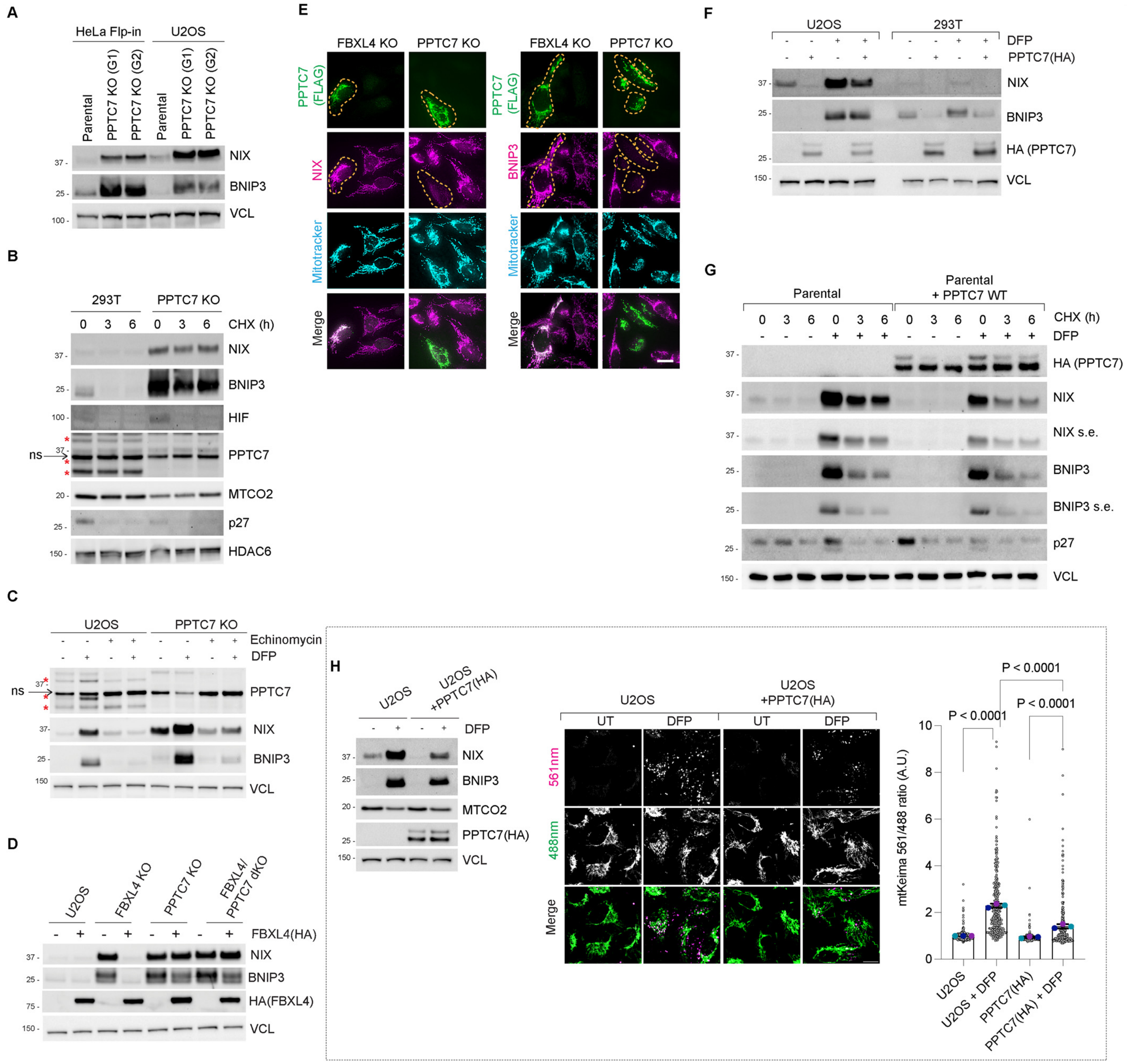
A
HeLa Flp-in
U2OS
Parental
PPTC7 KO (G1)
PPTC7 KO (G2)
NIX
BNIP3
VCL
B
293T
PPTC7 KO
CHX (h)
HIF
PPTC7
MTCO2
p27
HDAC6
ns
C
Echinomycin
DFP
D
FBXL4 KO
PPTC7 KO
FBXL4/PPTC7 dKO
FBXL4(HA)
HA(FBXL4)
E
PPTC7 (FLAG)
Mitotracker
Merge
F
293T
PPTC7(HA)
HA (PPTC7)
G
Parental
Parental + PPTC7 WT
NIX s.e.
BNIP3 s.e.
H
U2OS +PPTC7(HA)
UT
561nm
488nm
mtKeima 561/488 ratio (A.U.)
P < 0.0001
U2OS + DFP
PPTC7(HA) + DFP

 © The Author(s)

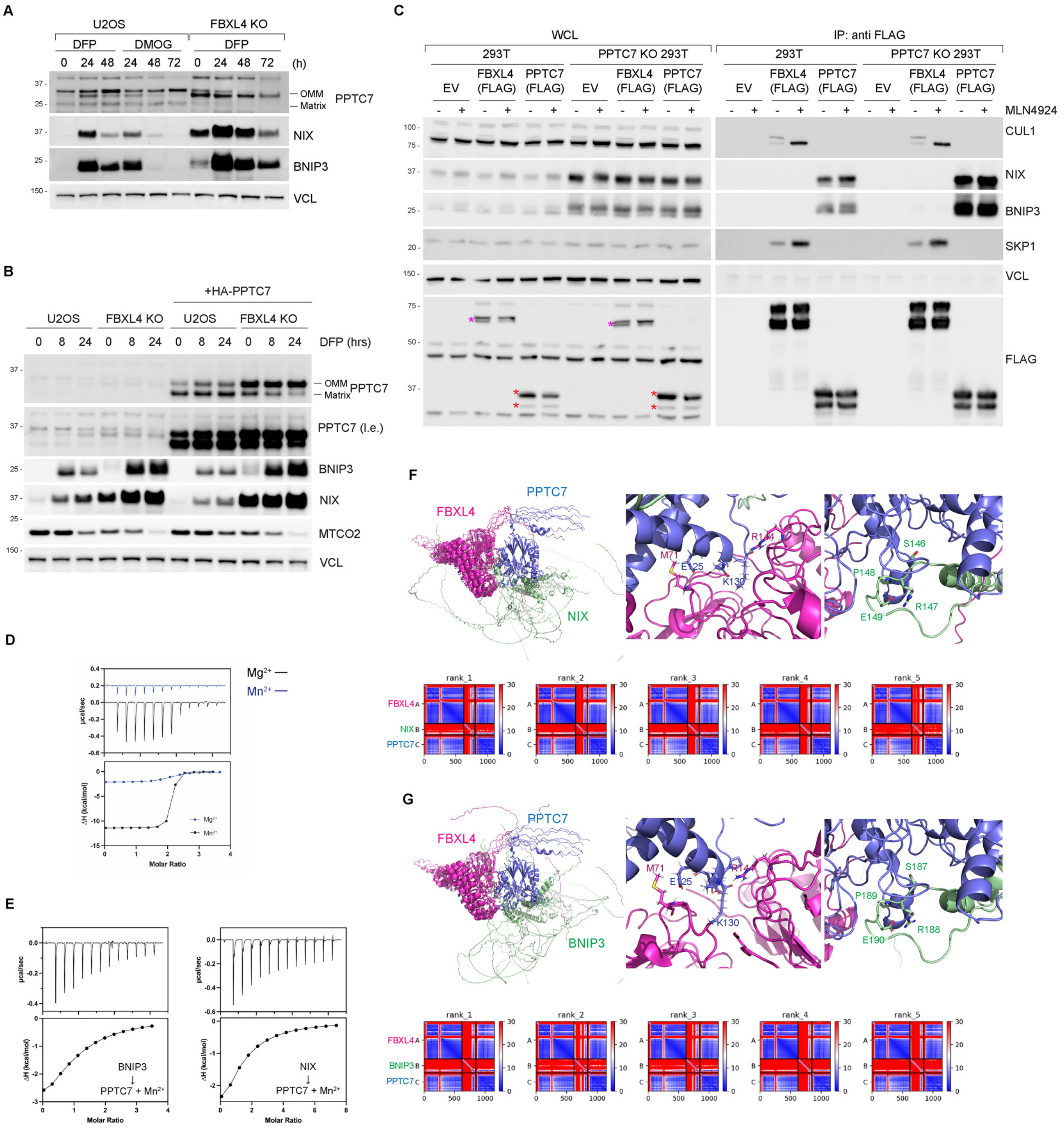

**Figure EV2. PPTC7 interacts with NIX/BNIP3 and FBXL4 and is not required for FBXL4 to interact with CUL1 and SKP1.**

(**A**) Analysis of the stability of the outer membrane form of PPTC7 in response to DFP treatment, DMOG treatment, and/or FBXL4 deficiency. (**B**) Analysis of the stability of the outer membrane form of endogenous PPTC7 and exogenous PPTC7(HA) in response to DFP treatment. (**C**) PPTC7 is not required for FBXL4 to interact with CUL1 or SKP1. 293T or 293T PPTC7 KO cells were transfected with either FLAG-FBXL4 or FLAG-PPTC7. Cells were treated with MLN4924 for 24 h where indicated. Cell lysates were immunoprecipitated with anti-FLAG beads, and the immuno-precipitates were analysed by immunoblotting as indicated. FBXL4 binds to CUL1 and SKP1, whereas PPTC7 binds to BNIP3 and NIX. WCL = whole-cell lysates. Red asterisks mark the PPTC7 transfected product and magenta asterisks mark the FBXL4 transfected product. (**D**) ITC comparison of PPTC7 wild-type binding to $Mg^{2+}$ and $Mn^{2+}$. The binding affinities were 6.21 nM for $Mn^{2+}$ and 237 nM for $Mg^{2+}$. (**E**) ITC comparison of PPTC7 wild-type binding to BNIP3 and NIX in the presence of $Mn^{2+}$. The binding affinities were 20.1 μM for BNIP3 and 37.5 μM for NIX. (**F**) Overlay of the top 5 AlphaFold2 models of FBXL4, PPTC7, NIX with predicted aligned error (PAE) plots. (**G**) Overlay of the top 5 AlphaFold2 models of FBXL4, PPTC7, BNIP3 with PAE plots.

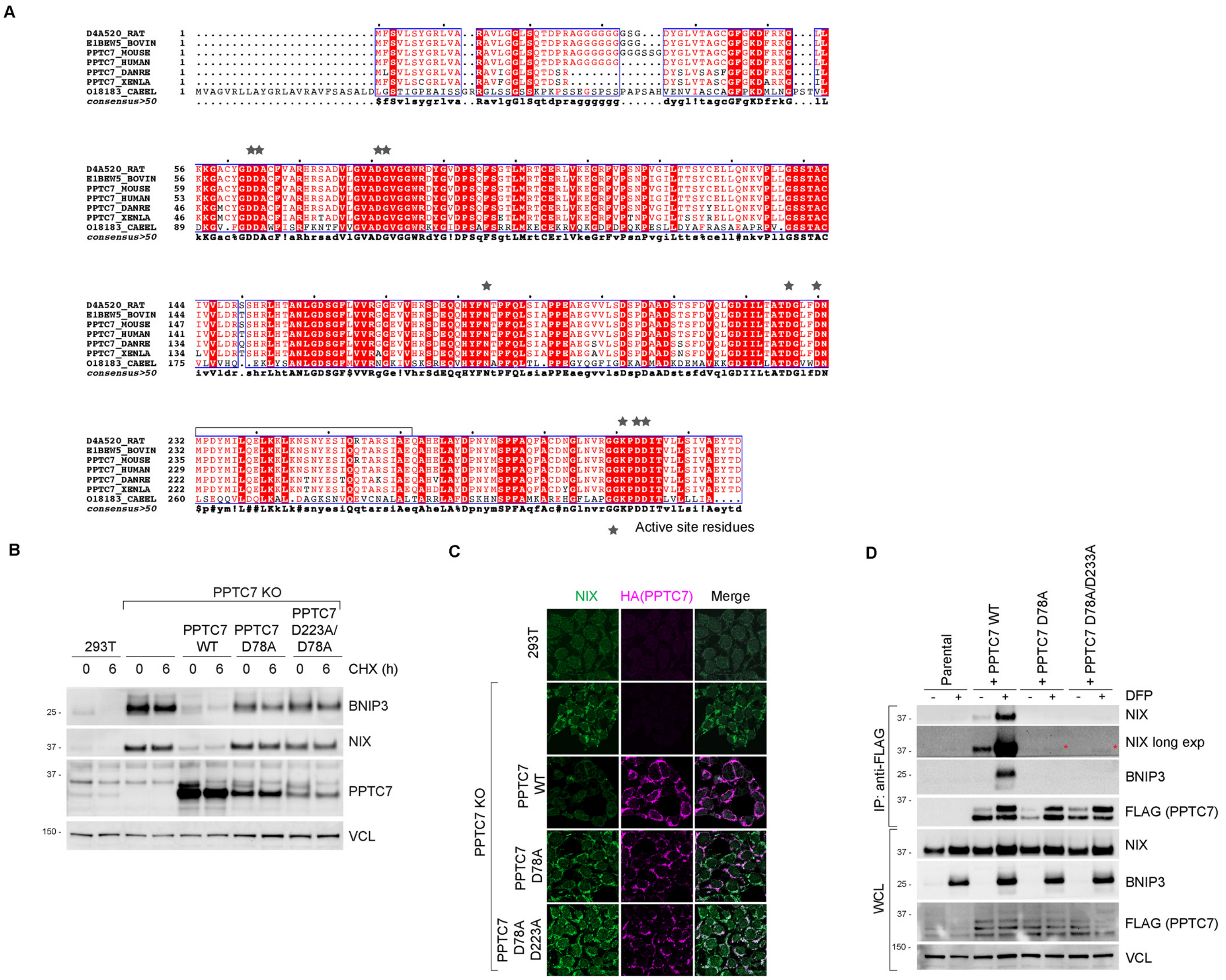


**Figure EV3. Radical disruption of PPTC7's catalytic site interferes with its binding to BNIP3 and NIX.**

(**A**) Sequence alignment of PPTC7 orthologues with active site residues from Fig. 3A indicated. (**B**) Disruption of the PPTC7's active site residues from aspartate to alanine compromises its ability to downregulate BNIP3 and NIX. PPTC7 KO cells were transduced with PPTC7 wild-type, PPTC7-D78A, or PPTC7-D223A/D78A. Wild-type PPTC7 rescued the turnover of BNIP3 and NIX, however, the D78A and D223A/D78A variants did not. (**C**) Aspartate to alanine mutations in PPTC7's active cannot rescue the downregulation of NIX by PPTC7. PPTC7 KO cells were complemented with PPTC7 or active site mutants and NIX levels were analysed by immunofluorescence microscopy. Scale bar = 20 microns. (**D**) Disruption of the PPTC7's active site residues from aspartate to alanine interferes with its ability to bind to BNIP3 and NIX. Cell lysates expressing PPTC7(FLAG) and mutants were immunoprecipitated with anti-FLAG beads, and the immuno-precipitates were analysed by immunoblotting as shown. Unlike the PPTC7-D78N mutant in Fig. 3B, the D78A mutant is unable to bind to BNIP3 or NIX.

 © The Author(s)

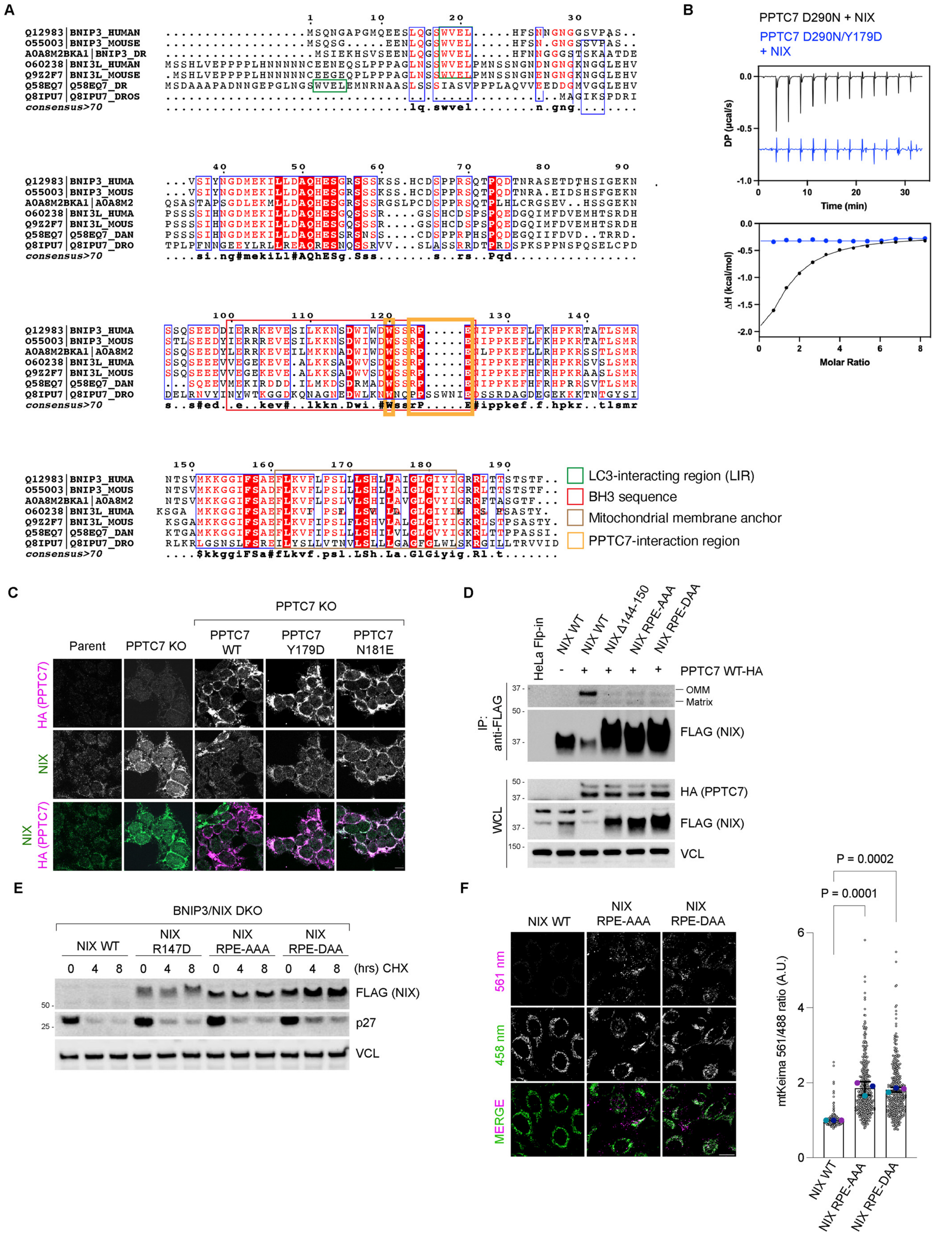
A
Q12983|BNIP3_HUMAN
O55003|BNIP3_MOUSE
A0A8M2BKA1|BNIP3_DR
O60238|BNI3L_HUMAN
Q9Z2F7|BNI3L_MOUSE
Q58EQ7|Q58EQ7_DR
Q8IPU7|Q8IPU7_DROS
consensus>70
LC3-interacting region (LIR)
BH3 sequence
Mitochondrial membrane anchor
PPTC7-interaction region
B
PPTC7 D290N + NIX
PPTC7 D290N/Y179D + NIX
DP (µcal/s)
Time (min)
ΔH (kcal/mol)
Molar Ratio
C
PPTC7 KO
Parent
PPTC7 KO
PPTC7 WT
PPTC7 Y179D
PPTC7 N181E
HA (PPTC7)
NIX
NIX HA (PPTC7)
D
HeLa Flp-in
NIX WT
NIX WT
NIX Δ144-150
NIX RPE-AAA
NIX RPE-DAA
PPTC7 WT-HA
OMM
Matrix
IP: anti-FLAG
FLAG (NIX)
WCL
HA (PPTC7)
FLAG (NIX)
VCL
E
BNIP3/NIX DKO
NIX WT
NIX R147D
NIX RPE-AAA
NIX RPE-DAA
(hrs) CHX
FLAG (NIX)
p27
VCL
F
NIX WT
NIX RPE-AAA
NIX RPE-DAA
561 nm
458 nm
MERGE
P = 0.0002
P = 0.0001
mtKeima 561/488 ratio (A.U.)
NIX WT
NIX RPE-AAA
NIX RPE-DAA

**Figure EV4. The NIX-PPTC7 interaction is critical for NIX turnover and mitophagy suppression.**

(**A**) Sequence alignment of BNIP3 and NIX orthologues. Functionally relevant motifs or domains are indicated. BNIP3 accession Q12983 (194 aa) replaces the previous BNIP3 accession EAW49143.1 (259 aa) characterised previously (Nguyen-Dien et al, 2023). (**B**) ITC comparison of PPTC7-D290N and PPTC7-D290N/Y179D binding to NIX peptide. The binding affinities were 35.9 ± 1.09 for PPTC7-D290N and non-binding for PPTC7-D290N/Y179D. (**C**) PPTC7-Y179D and PPTC7-N181E variants localise to mitochondria. Wild-type PPTC7 can reduce NIX levels when expressed in PPTC7 KO cells, but PPTC7-Y179D and PPTC7-N181E cannot. (**D**) Arg147 in NIX is critical for binding to PPTC7. PPTC7(HA) was transduced into cell lines expressing inducible NIX mutants. NIX expression was induced with doxycycline for 24 h. Cell lysates were immunoprecipitated with anti-FLAG beads, and the immuno-precipitates were analysed by immunoblotting. (**E**) Arg147 in NIX is critical for its turnover. HeLa Flp-in BNIP3/NIX double KO cells expressing FLAG-tagged NIX-WT or NIX binding mutants (FLAG-tagged NIX Δ144-150, NIX-RPE-AAA and NIX-RPE-DAA) were subjected to a cycloheximide chase. (**F**) Expression of NIX-RPE/AAA and NIX-RPE/DAA in NIX leads to an increase in basal levels of mitophagy compared with NIX-wildtype. Hela Flp-In NIX knockout/ Hela Flp-In BNIP3/NIX double knockout Keima cells stably expressing NIX mutants were treated with doxycycline for 48 h and mitophagy was evaluated using live-cell confocal fluorescence microscopy. Translucent grey dots represent measurements from individual cells. Coloured circles represent the mean ratio from independent experiments. The centre lines and bars represent the mean of the independent replicates +/− standard deviation. *P* values were calculated based on the mean values using a one-way ANOVA. Data Information: (**C**, **F**) Scale bars = 20 microns.

 © The Author(s)
