# Supplementary material for: PPTC7 antagonizes mitophagy by promoting BNIP3 and NIX degradation via SCFFBXL4
Source: EMBO Rep. 2024 Jul 11;25(8):3324–47. doi: 10.1038/s44319-024-00181-y (PMC11316107; doi:10.1038/s44319-024-00181-y)
Supplement: Supplementary file 3 — Source data Fig. 1 [file Annotation Figure 1C.pdf › Figure 1/Figure 1C/Annotation Figure 1C.pdf]

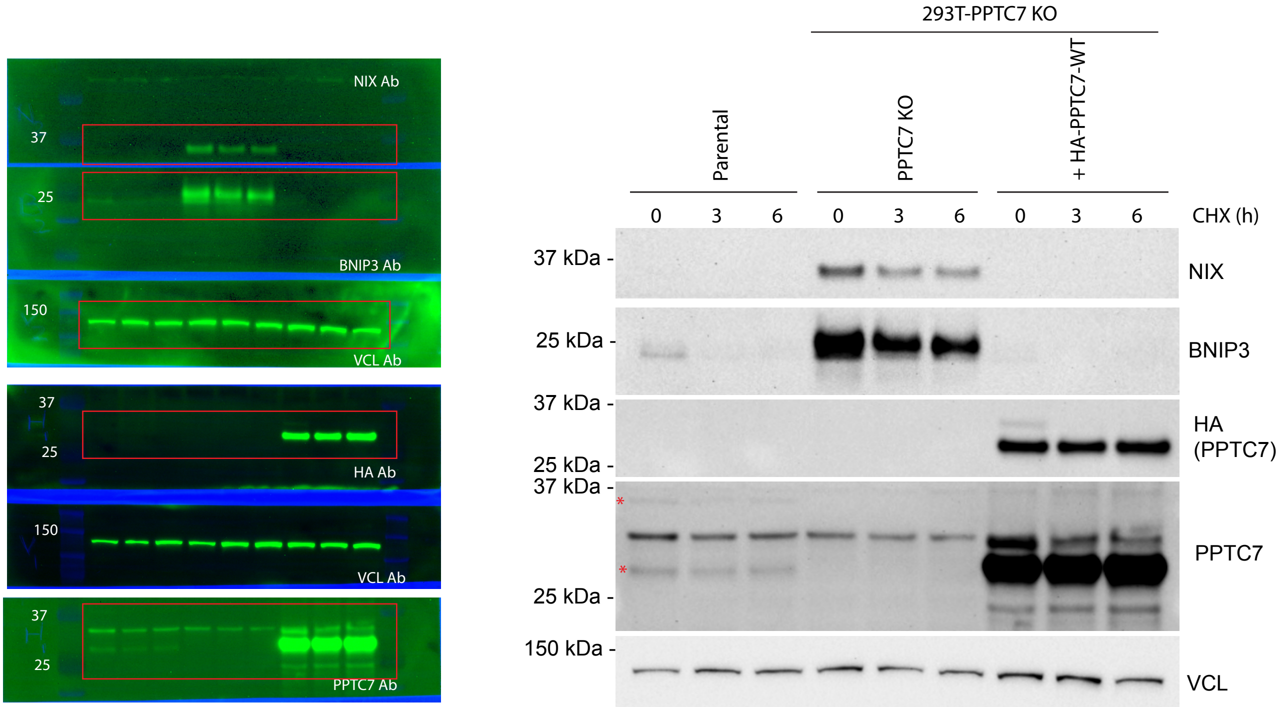
NIX Ab
37
25
BNIP3 Ab
150
VCL Ab
37
25
HA Ab
150
VCL Ab
37
25
PPTC7 Ab
293T-PPTC7 KO
Parental
PPTC7 KO
+ HA-PPTC7-WT
0 3 6 0 3 6 0 3 6
CHX (h)
37 kDa -
NIX
25 kDa -
BNIP3
37 kDa -
25 kDa -
HA
(PPTC7)
37 kDa -
*
*
25 kDa -
PPTC7
150 kDa -
VCL
