# Supplementary material for: PPTC7 antagonizes mitophagy by promoting BNIP3 and NIX degradation via SCFFBXL4
Source: EMBO Rep. 2024 Jul 11;25(8):3324–47. doi: 10.1038/s44319-024-00181-y (PMC11316107; doi:10.1038/s44319-024-00181-y)
Supplement: Supplementary file 4 — Source data Fig. 2 [file Annotation Figure 2B.pdf › Figure 2/Figure 2B/Annotation Figure 2B.pdf]

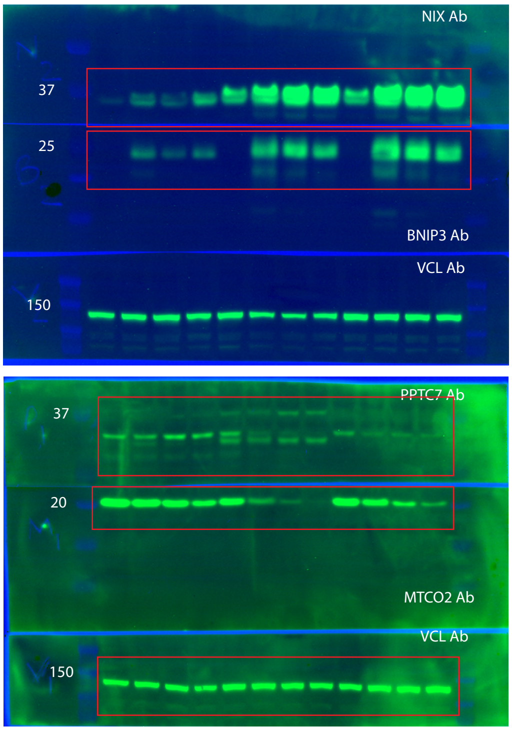
NIX Ab
37
25
BNIP3 Ab
VCL Ab
150
PPTC7 Ab
37
20
MTCO2 Ab
VCL Ab
150

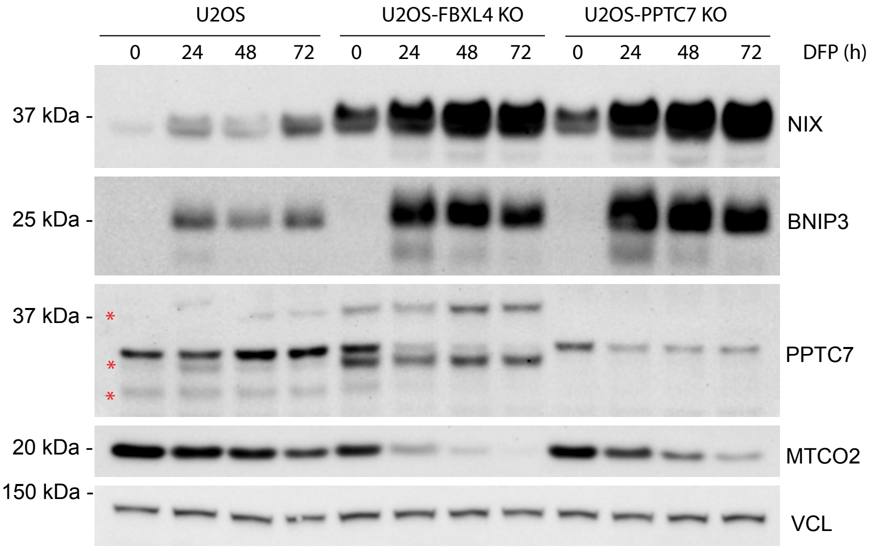
U2OS
U2OS-FBXL4 KO
U2OS-PPTC7 KO
0
24
48
72
0
24
48
72
0
24
48
72
DFP (h)
37 kDa -
NIX
25 kDa -
BNIP3
37 kDa -
*
*
*
PPTC7
20 kDa -
MTCO2
150 kDa -
VCL
