# Supplementary material for: PPTC7 antagonizes mitophagy by promoting BNIP3 and NIX degradation via SCFFBXL4
Source: EMBO Rep. 2024 Jul 11;25(8):3324–47. doi: 10.1038/s44319-024-00181-y (PMC11316107; doi:10.1038/s44319-024-00181-y)
Supplement: Supplementary file 6 — Source data Fig. 4 [file Annotation Figure 4C.pdf › Figure 4/Figure 4C/Annotation Figure 4C.pdf]

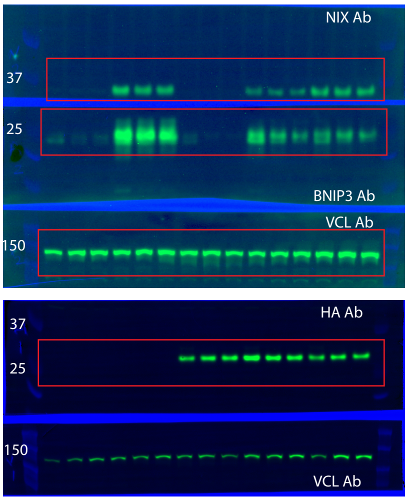
NIX Ab
37
25
BNIP3 Ab
VCL Ab
150
HA Ab
37
25
150
VCL Ab

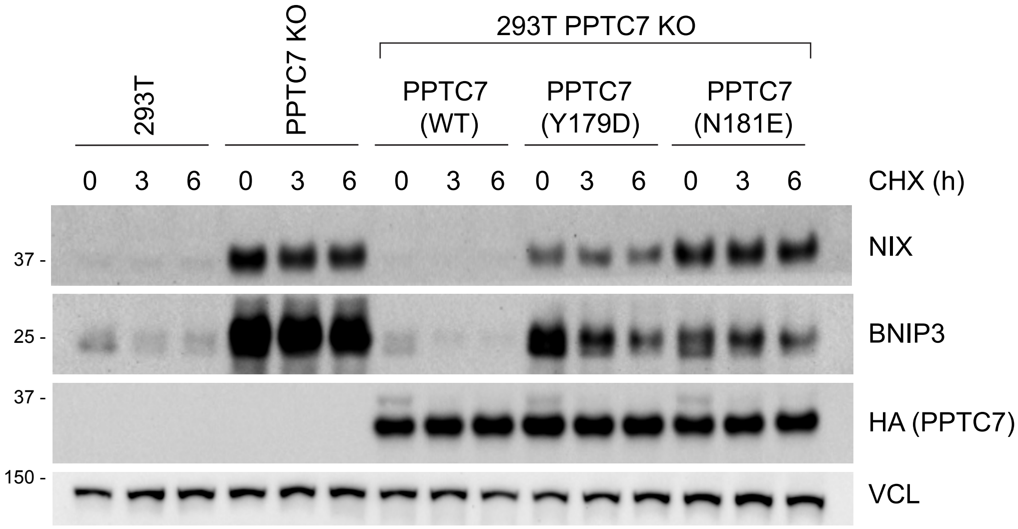
293T PPTC7 KO
293T
PPTC7 KO
PPTC7 (WT)
PPTC7 (Y179D)
PPTC7 (N181E)
0 3 6 0 3 6 0 3 6 0 3 6 0 3 6
CHX (h)
37 -
NIX
25 -
BNIP3
37 -
HA (PPTC7)
150 -
VCL
