# Supplementary material for: PPTC7 antagonizes mitophagy by promoting BNIP3 and NIX degradation via SCFFBXL4
Source: EMBO Rep. 2024 Jul 11;25(8):3324–47. doi: 10.1038/s44319-024-00181-y (PMC11316107; doi:10.1038/s44319-024-00181-y)
Supplement: Supplementary file 7 — Source data Fig. 5 [file Annotation Figure 5E.pdf › Figure 5/Figure 5E/Annotation Figure 5E.pdf]

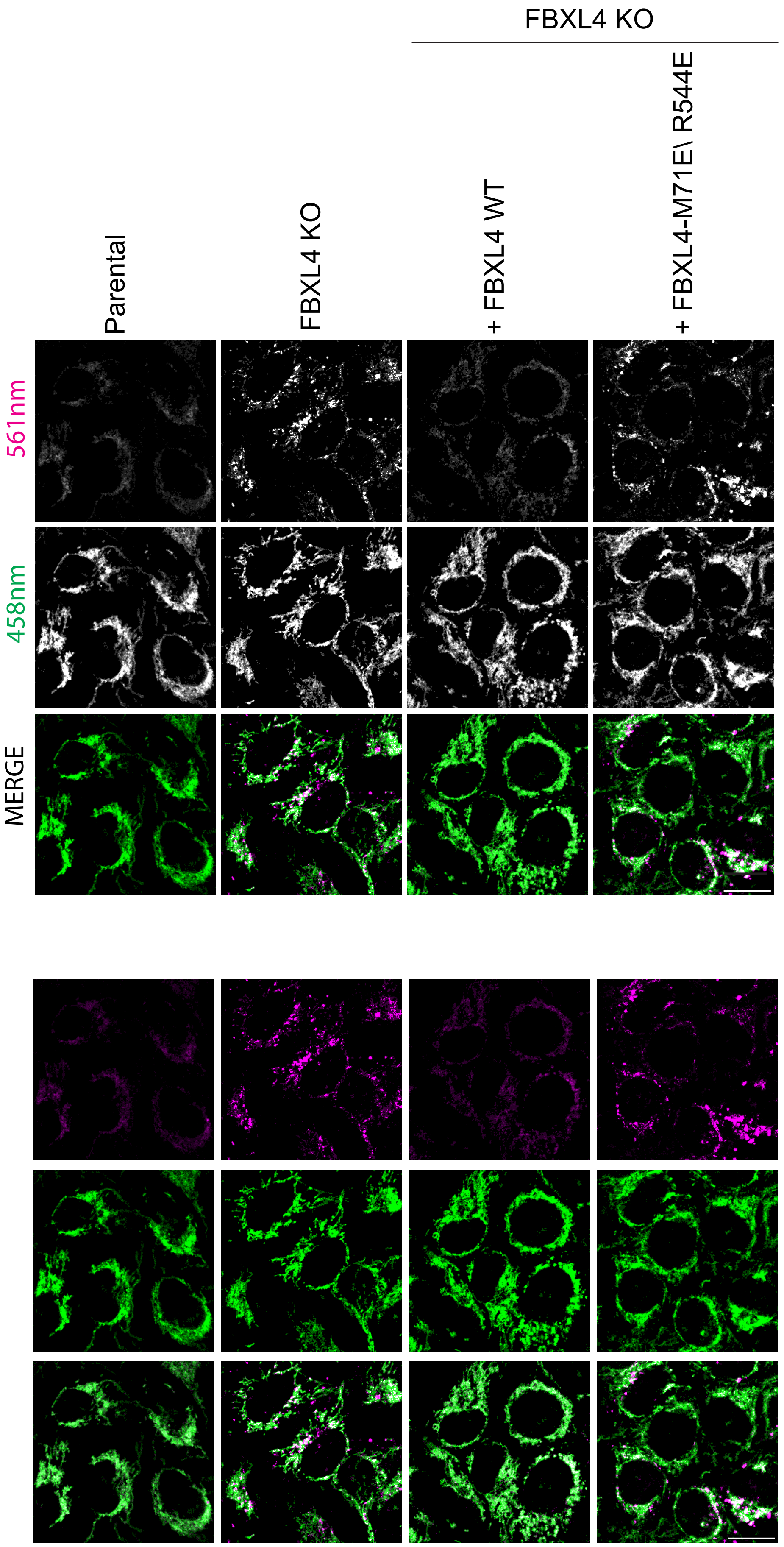

FBXL4 KO
Parental
FBXL4 KO
+ FBXL4 WT
+ FBXL4-M71E\ R544E
561nm
458nm
MERGE
